# Acupuncture and moxibustion for chronic fatigue syndrome: A systematic review and network meta-analysis

**DOI:** 10.1097/MD.0000000000029310

**Published:** 2022-08-05

**Authors:** Yang Fang, Bo-Wen Yue, Han-Bo Ma, Yi-Peng Yuan

**Affiliations:** a School of Acupuncture-Moxibustion and Tuina, Beijing University of Chinese Medicine, Beijing, China; b Shenzhen Bao’an Traditional Chinese Medicine Hospital, Guangzhou University of Chinese Medicine, Shenzhen, Guangdong Province, China.

**Keywords:** acupuncture, chronic fatigue syndrome, moxibustion, network meta-analysis, traditional Chinese medicine

## Abstract

**Background:**

Research into acupuncture and moxibustion and their application for chronic fatigue syndrome (CFS) has been growing, but the findings have been inconsistent.

**Objective:**

To evaluate the existing randomized clinical trials (RCTs), compare the efficacy of acupuncture, moxibustion and other traditional Chinese medicine (TCM) treatments.

**Data sources:**

Three English-language databases (PubMed, Embase, Web of Science, and The Cochrane Library) and 4 Chinese-language biomedical databases (Chinese Biomedical Literature Database, VIP Database for Chinese Technical Periodicals, China National Knowledge Infrastructure, and Wanfang) were searched for RCTs published from database inception through August 2021.

**Study selection:**

RCTs include acupuncture, moxibustion, traditional Chinese herbal medicine, western medicine and no control.

**Data extraction and synthesis:**

Data were screened and extracted independently using predesigned forms. The quality of RCTs was appraised with the Cochrane Collaboration risk of bias tool. We conducted a random-effects network meta-analysis within a frequentist framework. We assessed the certainty of evidence contributing to network estimates of the main outcomes with the Grading of Recommendations Assessment, Development and Evaluation (GRADE) framework.

**Main outcomes and measures:**

The primary outcomes were the overall response rate and FS-14 scale.

**Results:**

A total of 51 randomized controlled trials involving 3473 patients with CFS were included in this review. Forty one studies indicate low risk or unknown risk, and the GRADE scores of the combined results show low levels. Among the main indicators, traditional Chinese medicine therapies have excellent performance. However, the overall response rate is slightly different from the results obtained from the Fatigue Scale-14 total score. Moxibustion and traditional Chinese medicine (Odds ratios 48, 95% CrI 15–150) perform better in the total effective rate, while moxibustion plus acupuncture (MD 4.5, 95% CrI 3.0–5.9) is better in the FS-14 total score.

**Conclusions:**

The effect of acupuncture and moxibustion in the treatment of CFS was significantly higher than that of other treatments. Traditional Chinese medicine should be used more widely in the treatment of CFS.

## 1. Introduction

The chronic fatigue syndrome (CFS) is characterized by severe and disabling fatigue.^[1]^ In addition to fatigue, individuals with CFS also report a variety of other symptoms including muscu-loskeletal pain, sleep disturbance, impairment in short term memory and concentration, sore throat, and headaches of new type, pattern and severity. In nearly all cases there is an exacerbation of these symptoms, but particularly the fatigue by any form of physical, mental and sometimes emotional exertion. Symptom severity may also fluctuate on a daily or weekly basis without obvious cause. At present, there are 3 main hypotheses regarding the pathology and physiology of CFS: acute viral or nonviral pathogens and other persistent infections, continuous immune response leading to chronic cytokine production mediating long-term symptoms (immune disorders), and genetic factors. Most researchers argue that Chronic fatigue syndrome is closely related to neuroinflammation after infection.^[2,3]^ The cytokines produced in the process of neuroinflammation could affect the hypothalamus-pituitary-adrenal axis, cause mild cortisol hypoxia, increase negative feedback and slow response, and that leads to the occurrence of CFS.^[4]^

CFS is associated with a variety of diseases, such as brucellosis, coronavirus infection, depression, cancer, etc,^[5,6]^ but because only fatigue symptoms appear in the early onset of CFS, it is hardly to cause enough attention from patients. Moreover, the diagnosis method of CFS is relatively old, and nearly 80% of patients are difficult to diagnose in time, which delays their treatment.^[7]^ CFS affects 0.1% to 0.5% of the population in the world.^[8]^ In a recent review of the CFS literature reported by the Institute of Medicine of the United States (US), it was estimated that between 836,000 and 2.5 million Americans suffer from CFS. The most common age group of the disease is between 25 and 40 years old, causing an unemployment rate of 35% to 69%,^[9]^ which brings approximately $20,000 in economic losses to each family.^[10]^ The incidence of CFS is slightly higher in developing countries than in developed countries,^[11]^ and the worldwide prevalence of COVID-19 since late 2019 has increased the number of CFS patients,^[12]^ making the global economic recovery worse.^[13]^

The current guidelines for the treatment of chronic fatigue syndrome are only cognitive behavioral therapy, graded exercise programs and symptomatic treatment.^[9]^ It is not always possible to obtain satisfactory and expected curative effects in clinical application. Therefore, we urgently need to find more safe and effective therapies to supplement. Traditional Chinese Medicine (TCM) is a therapy widely used in China. Its treatment methods include acupuncture, moxibustion, cupping, and Traditional Herbal Chinese Medicine (THM). Among these therapies, acupuncture and moxibustion are simple, convenient, and relatively safe, and they are now accepted by a growing number of people in Western countries. Acupuncture and moxibustion of chronic fatigue syndrome efficacy and safety has been assessed by many clinical trials.^[14,15]^ Therefore, in this study, we performed a systematic review and network meta-analysis mesh to compare efficacy of acupuncture, moxibustion and other therapies.

### 1.1. Protocol registration

This system review program will strictly follow the system review and meta-analysis program (PRISMA-P) preferred report items for reporting. The system review program has been registered on the PROSPERO website (the registration number is CRD42020210391). If there are any adjustments during the entire study period, we will promptly revise and update the detailed information in the final report.^[16]^

## 2. Method

### 2.1. Search strategy and selection criteria

Eight English and Chinese medical databases, including PubMed, The Cochrane Library, Web of Science, EMBASE, the China National Knowledge Infrastructure (CNKI [Chinese version]), Wanfang Data (Chinese version), CQVIP (Chinese version), and SionMed (Chinese version) were electronically searched from inception to August 2021. Only studies published in Chinese or English were included in this systematic review and meta-analysis. According to the “PICOS” principle to formulate the search strategy, we used search terms including: “acupuncture,” “acupuncture points,” “acupuncture therapy,” “acupuncture, ear,” “dry needling,” “trigger points,” “moxibustion,” “meridians,” “acupoint injection,” “auricular acupuncture,” “auricular plaster,” “body acupuncture,” “coiling dragon needling,” “dermal needle,” “ear acupuncture,” “ear seed pressure,” “electro-acupuncture,” “Embedding,” “embedding therapy,” “fire needle,” “panlongci,” “percusso-puncture,” “point injection,” “pricking blood,” “scalp acupuncture,” “fatigue syndrome, chronic,” “chronic fatigue syndrome,” “chronic fatigue,” “fatigue syndrome,” “myalgic encephalopathy.” The combination of subject words and free words was used in the retrieval, and the references that had been included in the literature were supplemented. In addition to searching electronic databases, we also conducted manual searches on published, unpublished or ongoing RCT studies in the U.S. National Library of Medicine Clinical Trial Registry and the Chinese Clinical Trial Registry.

We included randomized controlled trials (RCT) and observational trials. The diagnostic criteria of the included trials should be consistent with the “CFS diagnostic criteria revised by the Centers for Disease Control and Prevention in 1994 (Fukuda’s 1994)^[17]^: unexplained sustained or repeated episodes of severe fatigue lasted for 6 months or more. The symptoms did not relieve after adequate rest, and the activity level decreased by 50% compared with healthy conditions.” The control group received placebo or no treatment, while the treatment group received TCM treatment based on conventional treatment, including acupuncture with moxibustion, only acupuncture, only moxibustion, acupuncture with THM, moxibustion with THM, only THM, Western medicine (Western medicine includes vitamin C, oryzanol, etc. THM includes Chinese medicine decoction, Chinese patent medicine, etc.). The treatment process, dosage and operation are not considered. The primary outcome indicator should include at least one of the overall response rates of CFS^[18]^ treatment or Chalder’s Fatigue Scale (FS-14).^[19]^

Two pairs of researchers (Yang Fang, Bowen Yue, Yipeng Yuan, Hanbo Ma) independently carried out literature screening, data extraction and literature bias evaluation. We extracted data double-track entry to check in case of differences or disputes by discussion or consultation third-party solutions. Data extraction includes author, year, random method, random hiding method, sample size, age, course of the disease, intervention measures, course of treatment, overall response rate, FS-14 scale, follow-up time, etc. We assessed the studies’ risk of bias in accordance with the Cochrane Handbook for Systematic Reviews of Interventions. Additionally, we assessed the certainty of evidence contributing to network estimates of the main outcomes with the Grading of Recommendations Assessment, Development and Evaluation (GRADE) framework.^[20]^

### 2.2. Data synthesis and statistical analysis

We conducted a random-effects network meta-analysis (NMA) within a frequentist framework using STATA (version 16.0) and R (version 4.0.3) software.^[21]^ Direct and indirect (and mixed) comparisons were accomplished through the self-programmed routines of STATA^[22,23]^ and the net-meta package of R.^[24]^ Further details of the methodology for NMA are described elsewhere.^[25,26]^ The effect estimation was in odds ratios (OR) for dichotomous variables and mean difference (MD) for continuous variables, both with 95% confidential intervals. When median (interquartile range) was presented for continuous variables of interest, it was converted to mean (standard deviation) by calculation.^[27,28]^ A 2-sided *P* value of less than .05 was regarded as statistically significant.

For the inconsistency test, based on the direct comparison data, the difference between the direct result and the indirect result is calculated, and the *Z* test is performed to detect the inconsistency. In each closed loop, the absolute difference between direct evidence and indirect evidence can be calculated, which can be expressed by an inconsistency factor. If the inconsistency factor value is close to 0 or the RoR value is close to 1, it means that the direct comparison evidence and the indirect comparison evidence are very consistent. A funnel plot was used to evaluate the small sample effect. The rank of effect estimation for each treatment was investigated using the surface under the cumulative rank curve of the P rank score of STATA.^[29]^ If there is enough data, we will consider subgroup analysis. The sensitivity of our conclusions was evaluated by analyzing the dataset with the following restrictions: studies with reported overall response rate, studies with unpublished data, multi-center studies, and head-to-head studies.

## 3. Results

### 3.1. Selected studies

**Figure 1.**
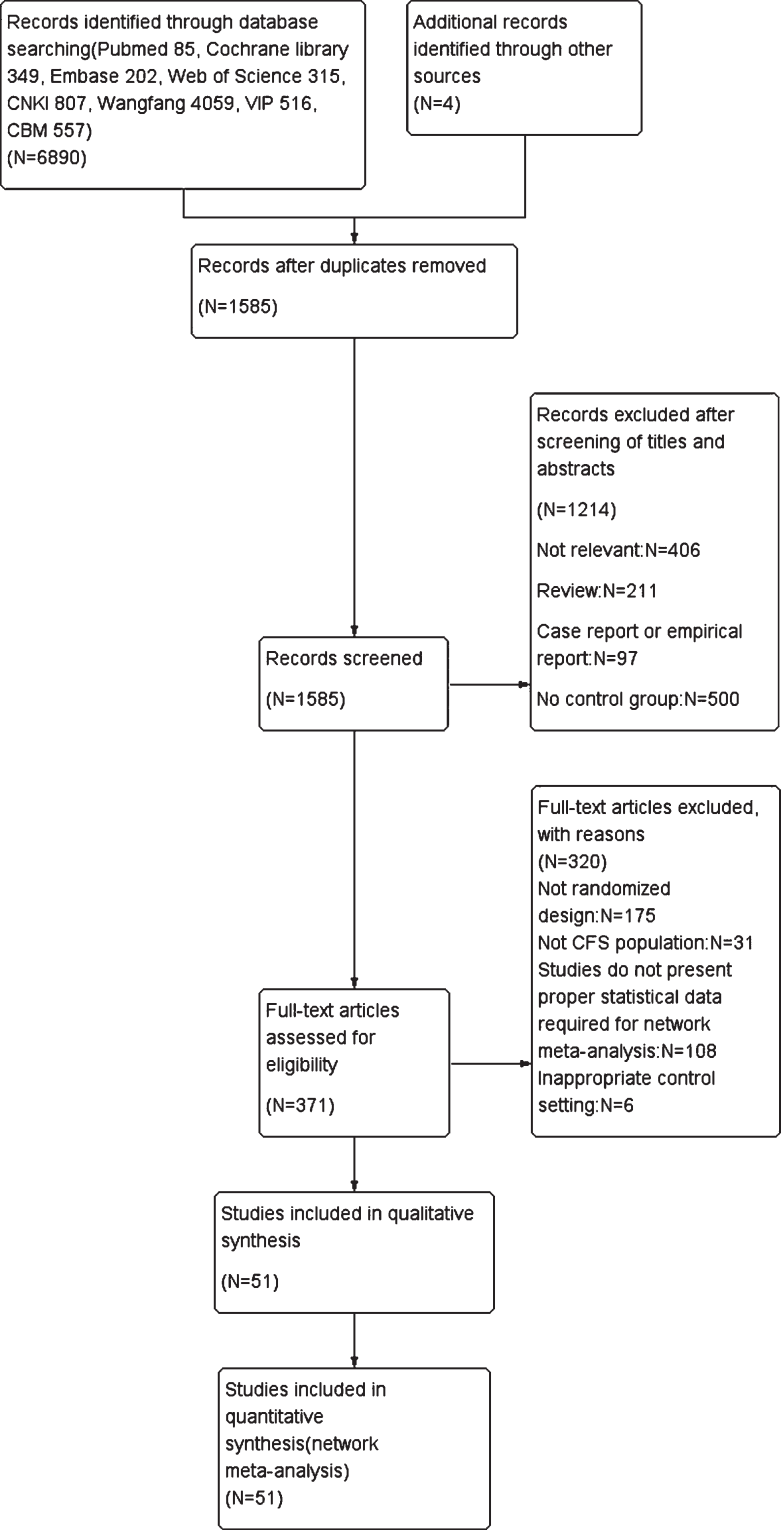
The preferred reporting items for systematic reviews and network meta-analyses (PRISMA) flow diagram.

The initial search identified 6890 articles. These studies were assessed for inclusion using the prespecified inclusion and exclusion criteria described in methods. Title and abstract of 1585 articles were assessed, and 371 studies were found suitable for full-text review. After excluding 320 studies, 51 RCTs studies and observational trials were finally included in our NMA. Among the included studies, 11 studies compared acupuncture + moxibustion with acupuncture,^[30–40]^ 8 studies compared acupuncture with moxibustion,^[41–48]^ 1 study compared acupuncture + moxibustion with moxibustion,^[49]^ 1 study compared acupuncture + traditional Chinese herbal medicine with Western medicine,^[50]^ 6 studies compared acupuncture with traditional Chinese herbal medicine,^[51–56]^ 1 study compared acupuncture with Western medicine,^[57]^ 4 studies compared acupuncture + traditional Chinese herbal medicine with traditional Chinese herbal medicine,^[50,58–60]^ 1 study Comparing acupuncture and moxibustion + traditional Chinese herbal medicine,^[61]^ 4 studies comparing moxibustion with western medicine,^[57,62–64]^ 2 study comparing moxibustion with moxibustion + traditional Chinese herbal medicine,^[65,66]^ 2 studies comparing moxibustion with traditional Chinese herbal medicine,^[67,68]^ 2 studies Comparing traditional Chinese herbal medicine with acupuncture plus moxibustion,^[69,70]^ 10 studies compared acupuncture with no control group,^[31,71–79]^ and 1 study compared acupuncture + moxibustion with no control group.^[80]^ A flow diagram of study inclusion is illustrated in Figure 1.

### 3.2. Study characteristics

A total of 3473 CFS patients were included. Most of these studies were single-center studies, only 3 were multi-center studies.^[52,60,76]^ Of these studies, 20 were conducted in northern China, 29 were conducted in southern China, 1 was conducted in Sydney, Australia,^[57]^ and 1 was conducted in Singapore.^[52]^ These studies were published from 2005 to 2020. The diagnostic criteria of the included studies follow the diagnostic criteria revised by the Centers for Disease Control and Prevention in 1994 (Fukuda’s 1994). The mean study sample size was 68 participants.

In the total sample size, there are 1445 males (41.6%) and 2028 females (58.4%), with an average age of 38.44 years and an average duration of 26.94 months. The average age and average disease course of each group are shown in the table (Supplemental Digital Content S1, http://links.lww.com/MD2/B16). The median duration of the CFS treatment was 4 weeks (IQR = 2). Of the 51 studies, 46 reported the overall response rate of treatment, 31 reported the change in the total score of FS-14 before and after treatment, and 13 studies respectively reported the physical and mental score of FS-14 before and after treatment Variety. We also counted the selection of acupoints in the trial (Supplemental Digital Content S1, http://links.lww.com/MD2/B16). Background characteristics and reference list of included studies are presented in appendix, http://links.lww.com/MD2/B21 (Supplemental Digital Content S2, http://links.lww.com/MD2/B17). The results were assessed according to The Cochrane Risk of Bias Assessment Tool (Kappa score = 0.895), and related results are summarized and graphically displayed with the support of RevMan software. The RoB in included studies was generally low to moderate (Supplemental Digital Content S3, http://links.lww.com/MD2/B18).

### 3.3. Results of meta-analysis: overall response rate

**Figure 2.**
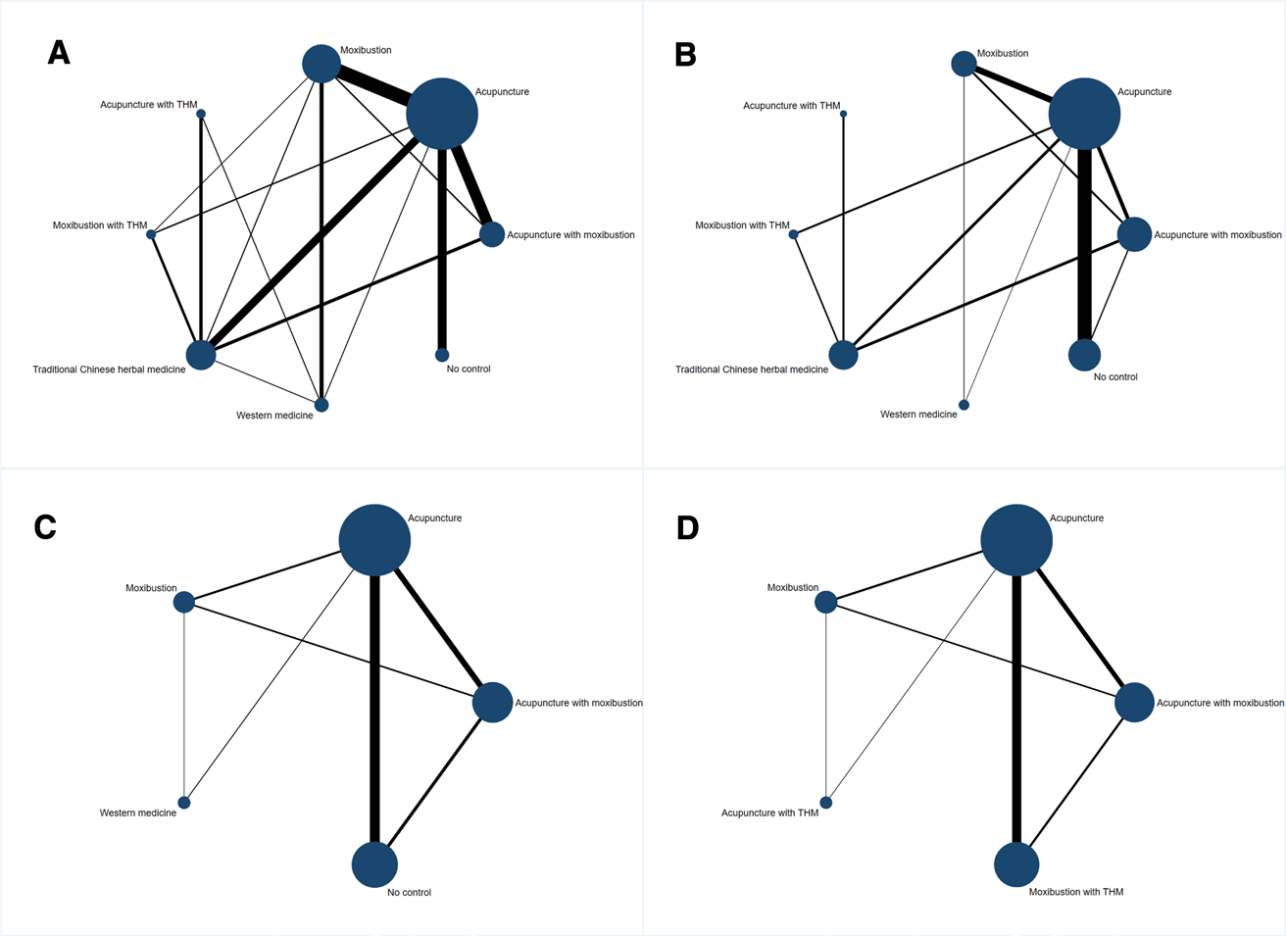
Network meta-analysis of eligible comparisons for overall response rate (A), FS-14 total score (B), FS-14 physical score (C) and FS-14 mental score (D). Width of the lines is proportional to the number of trials comparing every pair of treatments. Size of every circle is proportional to the number of randomly assigned participants (i.e., sample size).

**Figure 3.**
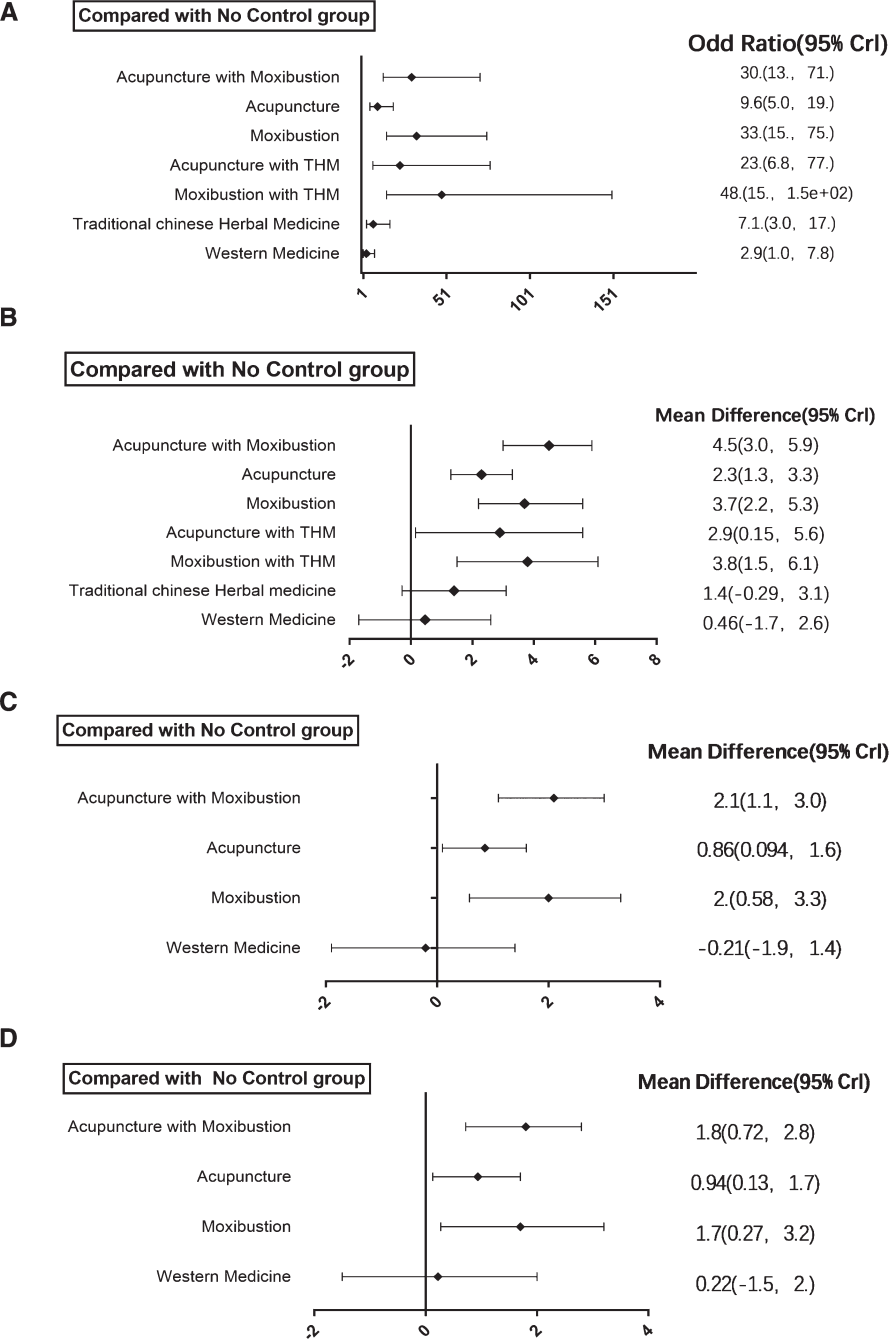
Forest plots of network meta-analysis of all trials for overall response rate (A), FS-14 total score (B), FS-14 physical score (C) and FS-14 mental score (D).

Forty six articles reported the overall response rate after CFS treatment. Figure 2A shows the qualified comparison network of CFS overall response rate. Except for the acupuncture + traditional Chinese herbal medicine group, all treatment measures have at least 1 comparison with the acupuncture + moxibustion group. Figure 3A shows the results of the network meta-analysis. In terms of overall response rate (46 studies, including 3083 participants), in addition to western medicine (OR 2.9, 95% Credible interval [CrI] 1.0–7.8), acupuncture + moxibustion (OR 30, 95% CrI 13–71), acupuncture (OR 9.6, 95% CrI 5.0–19), moxibustion (OR 33, 95% CrI 15–75), acupuncture + traditional Chinese herbal medicine (OR 23, 95% CrI 6.8–77), moxibustion + traditional Chinese herbal medicine (OR 48, 95% CrI 15–150), traditional Chinese herbal medicine (OR 7.1, 95% CrI 3.0–17) are more effective than the no control group. In the analysis of the overall response rate, none of the loops showed inconsistency (0 of 11 loops; *P* value of the design by treatment test was 0.3267), and the overall level of heterogeneity was low (Loop_Heterog_tau^2^ range: 0.000–0.287).

We have also synthesized head-to-head studies to assess the differences between the various therapies. Table 1A shows the main results of these data. Acupuncture + moxibustion, moxibustion + traditional Chinese herbal medicine, moxibustion, acupuncture + traditional Chinese herbal medicine are more effective than other therapies (MDs between 3.19 and 47.67), while western medicine and no control group are less effective than other therapies (MDs between 2.47 and 2.89). The table (Supplemental Digital Content S4, http://links.lww.com/MD2/B19) shows the GRADE judgment. Only the evidence of acupuncture plus moxibustion compared with moxibustion showed low certainty, and the evidence of moxibustion compared with moxibustion + traditional Chinses herbal medicine showed very low certainty. The results of rank probability analysis, from low to high, were as follows: no control group (0.0), western medicine (0.0), traditional Chinses herbal medicine (0.0), acupuncture (0.0), moxibustion (2.1), acupuncture + traditional Chinses herbal medicine (5.8), acupuncture + moxibustion (22.2), moxibustion + traditional Chinses herbal medicine (69.9) (Supplemental Digital Content S5, http://links.lww.com/MD2/B20).

**Table 1:** Head-to-head comparisons for overall response rate (A), FS-14 total score (B), FS-14 physical score (C) and FS-14 mental score (D) of the Traditional Chinese medicine therapy.

a							
Acupuncture with moxibustion							
3.11 (1.86, 5.28)	**Acupuncture**						
0.9 (0.46, 1.77)	0.29 (0.18, 0.46)	**Moxibustion**					
1.31 (0.42, 4.11)	0.42 (0.15, 1.18)	1.46 (0.48, 4.31)	**Acupuncture with THM**				
0.63 (0.22, 1.88)	0.2 (0.08, 0.52)	0.7 (0.27, 1.83)	0.48 (0.13, 1.81)	**Moxibustion with THM**			
4.2 (2.15, 8.68)	1.35 (0.8, 2.37)	4.68 (2.5, 9.05)	3.19 (1.3, 8.36)	6.68 (2.64, 17.21)	**Traditional Chinses herbal medicine**		
10.38 (4.25, 26.26)	3.33 (1.55, 7.29)	11.47 (5.48, 24.89)	7.91 (2.65, 25.2)	16.58 (5.3, 50.45)	2.47 (1.09, 5.51)	**Western medicine**	
29.89 (13.09, 71.88)	9.63 (5.03, 19.02)	33.19 (15.17, 75.48)	22.76 (6.71, 79.94)	47.67 (14.93, 147.62)	7.12 (3.02, 16.66)	2.89 (1.03, 7.91)	**No control**
**b**							
**Acupuncture with moxibustion**							
9.04 (2.88, 27.73)	**Acupuncture**						
2.2 (0.5, 9.5)	0.24 (0.07, 0.82)	**Moxibustion**					
5 (0.38, 65.46)	0.56 (0.04, 7.35)	2.3 (0.14, 38.67)	**Acupuncture with THM**				
1.98 (0.22, 17.3)	0.22 (0.03, 1.72)	0.9 (0.09, 9.59)	0.39 (0.02, 7.03)	**Moxibustion with THM**			
22.08 (5.28, 91.55)	2.45 (0.61, 9.78)	9.96 (1.76, 59.3)	4.36 (0.5, 37.92)	11 (1.77, 71.79)	**Traditional Chinses herbal medicine**		
54.61 (6.33, 445.72)	6.08 (0.89, 41.21)	24.88 (4.12, 151.54)	10.91 (0.43, 256.78)	27.79 (1.61, 456.6)	2.49 (0.24, 24.35)	**Western medicine**	
86.56 (20.2, 364.67)	9.58 (3.62, 26.23)	39.42 (8.57, 189.2)	16.96 (1.11, 265.34)	43.5 (4.61, 441.68)	3.91 (0.76, 21.41)	1.57 (0.19, 13.94)	**No control**
**c**							
**Acupuncture with moxibustion**							
3.37 (1.41, 7.98)	**Acupuncture**						
1.12 (0.3, 4.31)	0.33 (0.1, 1.08)		**Moxibustion**				
9.87 (1.85, 51.66)	2.95 (0.69, 12.73)		8.84 (2, 37.9)	**Western medicine**			
7.91 (2.92, 20.94)	2.35 (1.09, 4.98)		7.05 (1.79, 27.04)	0.8 (0.15, 4.06)		**No control**	
**d**							
**Acupuncture with moxibustion**							
2.32 (0.92, 5.81)	**Acupuncture**						
1.03 (0.25, 4.26)	0.44 (0.13, 1.57)		**Moxibustion**				
4.67 (0.8, 27.85)	2.01 (0.42, 9.99)		4.6 (0.97, 21.55)	**Western medicine**			
5.88 (2.03, 17.24)	2.54 (1.13, 5.79)		5.77 (1.32, 25.25)	1.26 (0.21, 7.36)		**No control**	

### 3.4. Results of meta-analysis: FS-14 total score of CFS patients

Thirty one articles reported the FS-14 total score after CFS treatment. Figure 2B shows the qualified comparison network of FS-14 total score. Except for the acupuncture + traditional Chinese herbal medicine group and western medicine group, all treatment measures have at least one comparison with the acupuncture + moxibustion group.

Figure 3B shows the results of the network meta-analysis. In terms of FS-14 total score (31 studies, including 2060 participants), except for western medicine (MD 0.46, 95% CrI −1.7–2.6) and traditional Chinese herbal medicine (MD 1.4, 95% CrI −0.29–3.1), acupuncture + moxibustion (MD 4.5, 95% CrI 3.0–5.9), acupuncture (MD 2.3, 95% CrI 1.3–3.3), moxibustion (MD 3.7, 95% CrI 2.2–5.3), acupuncture + traditional Chinese herbal medicine (MD 2.9, 95% CrI 0.15–5.6), acupuncture + moxibustion (MD 3.8, 95% CrI 1.5–6.1) are more effective than no control group.

In the analysis of the FS-14 total score, none of the loops showed inconsistency (0 of 5 loops; *P* value of the design by treatment test was 0.5026), and the overall level of heterogeneity was low to moderate (Loop_Heterog_tau^2^ range: 0.694–5.810). We have also synthesized head-to-head studies to assess the differences between the various therapies. Table 1B shows the main results of these data. There are 3 therapies (Acupuncture + moxibustion, moxibustion + traditional Chinese herbal medicine, moxibustion) more effective than others (ORs between 9.04 and 86.56), while traditional Chinese herbal medicine, western medicine and no control group are less effective than other therapies (ORs between 1.57 and 3.91). The table (Supplemental Digital Content S4, http://links.lww.com/MD2/B19) shows the GRADE judgment. Only the evidence of acupuncture compared with moxibustion, acupuncture compared with moxibustion + traditional Chinese herbal medicine, acupuncture compared with western medicine showed low certainty. The results of rank probability analysis, from low to high, were as follows: no control group (0.0), western medicine (0.0), traditional Chinese herbal medicine (0.0), acupuncture (0.0), acupuncture + traditional Chinese herbal medicine (6.9), moxibustion (8.7), moxibustion + traditional Chinese herbal medicine (21.8), acupuncture + moxibustion (62.6) (Supplemental Digital Content S5, http://links.lww.com/MD2/B20).

### 3.5. Results of meta-analysis: FS-14 physical score of CFS patients

Thirteen articles reported the FS-14 physical score after CFS treatment. Due to the influence of the quantity and quality of the literature, we only included 5 interventions: acupuncture + moxibustion, acupuncture, moxibustion, western medicine, and baseline blank. Figure 2C shows the qualified comparison network of FS-14 physical score. All treatment measures have at least one comparison with the acupuncture + moxibustion group.

Figure 3C shows the results of the network meta-analysis. In terms of FS-14 physical score (13 studies, including 978 participants), except for western medicine (MD −0.21, 95% CrI −1.9–1.4), acupuncture + moxibustion (MD 2.1, 95% CrI 1.1–3.0), acupuncture (MD 0.86, 95% CrI 0.094–1.6), moxibustion (MD 2, 95% CrI 0.58–3.3) are more effective than no control group.

In the analysis of FS-14 physical score, 66.7% of the loops were inconsistent (2 of 3 loops; *P* value of the design by treatment test was 0.0004), and the overall level of heterogeneity was low to moderate (Loop_Heterog_tau^2^ range: 0.000–0.838). The loops showing inconsistency are acupuncture + moxibustion ~ acupuncture ~ no control group (*P* = .013, 95% CrI 0.37–2.94), acupuncture~ moxibustion~ western medicine (*P* = .001, 95% CrI 0.36–1.43). We found that the inconsistency of acupuncture + moxibustion ~ acupuncture ~ no control group closed-loop originated from the study,^[30]^ and the inconsistency of the closed-loop decreased after exclusion (*P* = .076, 95% CrI 0.00–3.12). Through further analysis, we speculate that the inconsistency is most likely due to the higher FS-14 scores of patients in this article before treatment. The inconsistency of the acupuncture ~ moxibustion ~ western medicine closed loop was derived from the study,^[80]^ and the inconsistency of the closed-loop decreased after being excluded (*P* = .342, 95% CrI 0.00–2.71). Through further analysis, we speculate that the inconsistency is most likely caused by the large difference between the acupuncture points used in the acupuncture group in this thesis and those included in other studies. We have also synthesized head-to-head studies to assess the differences between the various therapies. Table 1C shows the main results of these data. Acupuncture + moxibustion and moxibustion are more effective than other therapies (MDs between 3.37 and 9.87), while western medicine is less effective than other therapies (MDs 0.8, 95% CrI 0.15–4.06). The table (Supplemental Digital Content S4, http://links.lww.com/MD2/B19) shows the GRADE judgment, the quality of the evidence in the studies showed low or very low levels. The results of rank probability analysis, from low to high, were as follows: no control group (0.0), western medicine (0.0), acupuncture (0.0), moxibustion (43.1), acupuncture + moxibustion (56.9) (Supplemental Digital Content S5, http://links.lww.com/MD2/B20).

### 3.6. Results of meta-analysis: FS-14 mental score of CFS patients

Thirteen articles reported the FS-14 mental score after CFS treatment. Due to the influence of the quantity and quality of the literature, we only included 5 interventions: acupuncture + moxibustion, acupuncture, moxibustion, western medicine, and baseline blank. Figure 2D shows the qualified comparison network of FS-14 mental score. All treatment measures have at least 1 comparison with the acupuncture + moxibustion group.

Figure 3D shows the results of the network meta-analysis. In terms of FS-14 mental score (13 studies, including 978 participants), except for western medicine (MD −0.22, 95% CrI −1.5–2.0), acupuncture + moxibustion (MD 1.8, 95% CrI 0.72–2.8), acupuncture (MD 0.94, 95% CrI 0.13–1.7), moxibustion (MD 1.7, 95% CrI 0.27–3.2) are more effective than no control group.

In the analysis of FS-14 mental score, none of the loops were inconsistent (0 of 3 loops; *P* value of the design by treatment test was .1187), and the overall level of heterogeneity was low to moderate (Loop_Heterog_tau^2^ range: 0.000–1.403). We have also synthesized head-to-head studies to assess the differences between the various therapies. Table 1D shows the main results of these data. The results show that there is no distinction between the various therapies. The table (Supplemental Digital Content S4, http://links.lww.com/MD2/B19) shows the GRADE judgment. The quality of the evidence in the studies showed low or very low levels. The results of rank probability analysis, from low to high, were as follows: no control group (0.0), acupuncture (0.3), western medicine (0.4), moxibustion (47.5), acupuncture + moxibustion (51.8) (Supplemental Digital Content S5, http://links.lww.com/MD2/B20).

### 3.7. Subgroup analysis and sensitivity analysis

We conducted a subgroup analysis of the main results by age, gender, course of the disease, duration of treatment, and the principle of point selection. The results showed that these indicators had no significant impact on the efficacy and the change of FS-14 score. According to the review plan, we also conducted a sensitivity analysis to evaluate the stability and reliability of the comprehensive results of the meta-analysis, and to evaluate whether a single study excessively affected the comprehensive results. The process was carried out using STATA. After excluding individual studies one by one, most of the results did not change substantially, indicating that the results of the meta-analysis were relatively stable.

### 3.8. Adverse reaction and safety

A total of 21 articles reported the occurrence of adverse reactions. Three studies^[37,47,69]^ reported a total of 3 cases of minor scalds in the moxibustion group, and 2 studies^[47,69]^ reported 2 cases of hematoma and 2 cases of fainting during acupuncture in the acupuncture group. All events were resolved on their own within a week, and the participants continued to receive treatment. No adverse reactions were observed in the remaining 18 studies. The incidence of acupuncture-related adverse reactions in 21 articles was 0.55% (n = 722), and the incidence of moxibustion-related adverse reactions was 0.69% (n = 432).

## 4. Discussion

This study is based on 51 RCT studies involving 3473 patients. Based on baseline treatment, they were randomly assigned to different TCM intervention groups, western medicine groups, and no control group. Compared with the previous similar meta-analysis, the analysis of this project is more comprehensive, because we include direct and indirect comparisons between 8 different therapies, and the evidence base is about twice that of the previous study (about 3500 vs 1350). This enables us to investigate more important results (such as the detailed comparison of the improvement of patients’ mental and physical levels after treatment) as well as many methodological issues (such as acupoint selection, research accuracy, novelty).

We found that all TCM methods are more effective than the no control group in overall response rate, physical score and mental score. In terms of the total score of FS-14, the intervention groups other than traditional Chinese herbal medicine and western medicine were more effective than the no control group. From the overall assessment, it is difficult to distinguish the gaps between the various TCM methods. The head-to-head analysis results of each group in terms of overall response rate, FS-14 total score, physical score, and mental score have more diversity compared with meta-analysis.

It can be seen from the results that moxibustion and the combination therapy containing moxibustion perform more prominently. The combination of moxibustion and traditional Chinese herbal medicine is among the best in the total effective rate. At the same time, the combination of moxibustion and acupuncture is outstanding in the FS-14 total score and physical score. After further comparing the efficacy evaluation with the details of the FS-14 score, we found that the efficacy evaluation focused on the improvement of the degree of fatigue syndrome. The FS-14 score included a more comprehensive evaluation of mental and physical. We inferred that this may be the cause of the difference in results.

At the same time, we found in the mental evaluation that the various TCM methods are superior to western medicine and no control group, but there is no obvious difference in efficacy between the TCM methods. We conducted frequency statistics on commonly used acupoints. The efficacy of these commonly used acupoints mainly regulates internal organ function and improves the symptoms of tissues and muscles around the acupoints. It does not significantly improve the memory, attention, logical ability and language organization ability contained in the FS-14 table. This may be the treatment in the mental evaluation reasons why measures cannot produce differences in efficacy.

We also conducted a subgroup analysis of age, gender, course of the disease, duration of treatment, and principle of acupoint selection. Different treatment courses, gender, course of the disease, and treatment duration have little effect on the efficacy. According to the relatively high effect size, TCM acupoint selection based on syndrome differentiation seems to be more effective than fixed acupuncture points. People aged ≤40 years old seem to be more effective after treatment.

Nowadays, there is a lack of clinical-specific treatments for CFS. Most patients in Asia tend to seek the help of TCM therapy. Acupuncture and moxibustion are popular among patients because of their simplicity, convenience and safety.

Neuroinflammation and immune dysfunction are found involved in the pathogenesis of CFS. Environmental, neurological, viral infection or stress triggers activate microglia to release more inflammatory factors, leading to HPA axis and mitochondrial dysfunction.^[81–83]^ Studies have shown that acupuncture stimulation can inhibit the activation of microglia and reduce neuroinflammation.^[84]^ SARS-Cov-2 infection can lead to resemblant clinical symptoms. Similar neuro pathobiology may be shared in CFS.^[85]^ Notably, acupuncture, which is effective in the treatment of CFS, may also shine in the treatment of COVID-19 sequelae.^[86]^

Moxibustion may affect CFS by up-regulating the expression of Progranulin (PGRN) in the hippocampus.^[87]^ The role of PGRN in the hippocampus includes neurotrophic effects,^[88]^ regulation of neurogenesis, protection of nerves^[89]^ and inhibition of inflammation.^[90]^ PGRN is a key regulator of inflammation. PGRN can inhibit the expression of pro-inflammatory cytokines (IL-1β, IL-6 and TNF-α) in glial cells, so it is an endogenous anti-apoptotic and anti-inflammatory nerve Protective agent. Moxibustion up-regulates the expression of PGRN has a positive effect on CFS. Considering that hippocampal neurogenesis can regulate the HPA axis,^[91,92]^ up-regulating the expression of PGRN may have a positive effect on CFS. Therefore, the results of this review show that acupuncture and moxibustion may play a positive role in the treatment of CFS. Studies have shown that CFS may be associated with gut microbiota. The efficacy of moxibustion on CFS may also be related to the regulation of intestinal flora balance.^[93]^

In the previous statistics, we found that BL13, BL 15, BL 18, BL 20, BL 23, ST36, SP6, CV4, CV6 are the most frequently selected acupuncture points, which are the same as the results obtained in the previous study,^[14]^ which can provide useful information for clinical operations.

Acupuncture and moxibustion can be used not only for pain relief, but also for diseases of the digestive system, respiratory system, and circulatory system.^[94,95]^ In addition, acupuncture and moxibustion also contribute to health care. With the improvement of medical devices, the operation of acupuncture and moxibustion has become easier and safer. For example, tube needles and moxibustion boxes can also be used without professional training. However, how to select acupoints for different diseases requires corresponding theoretical knowledge. Besides, to reduce infection, the concept of sterility is also necessary.

There are still some limitations in this study. First of all, only 2 studies were conducted outside of China, and the vast majority of patients in the studies were yellow races, so there is a lack of efficacy evaluation studies for different races. None of the studies have stratified or grouped patients with different baseline levels. Combining data from patients with higher baseline levels may cause significant bias in the experimental results. At the same time, we also found that the included literature has deficiencies in experimental design, random allocation concealment, blind implementation, safety reporting, etc., which makes the certainty of the level of evidence limited. Taking into account the above-mentioned problems and limitations, it is necessary to conduct further research in the future in combination with larger samples and a more standardized design of randomized controlled experiment data.

Despite these limitations, the findings of this network meta-analysis still represent the most comprehensive evidence base currently available to guide the initial selection of complementary therapies for chronic fatigue syndrome. Combining safety, effectiveness and difficulty of operation, moxibustion and acupuncture are the most recommended therapies. Choosing acupuncture treatment can avoid the side effects of anti-anxiety agents and antidepressants and the abuse of analgesics. Also acupuncture and moxibustion is more easily accepted by patients. Before using acupuncture and moxibustion to attempt pain relief, medical personnel only need simple operation training.

## 5. Conclusion

CFS is a disabling fatigue disease that is not easy to attract enough attention from patients. It has a wide range of diseases and is one of the complications of many acute infectious diseases (such as COVID-19). Since 2019, COVID-19 has spread worldwide. In the foreseeable future, CFS will become new trouble in the post epidemic era. Acupuncture and moxibustion has great potential in the treatment of CFS, but it has not received much attention nowadays. Our network meta-analysis results of existing clinical trials show that acupuncture and moxibustion are particularly effective and safe. However, considering the quality of the research, our results should be treated with caution.

## Acknowledgments

We are also grateful to Prof. Xun Li and Prof. Li-ping Wang for their technical guidance and support.

## Author contributions

Conceived and designed the study: Yang Fang, Bo-Wen Yue.

Data curation: Yang Fang, Bo-Wen Yue, Han-Bo Ma, Yi-Peng Yuan.

Literature search: Yang Fang.

Data analysis: Yang Fang.

Writing-original draft: Yang Fang, Bo-Wen Yue.

Writing-review & editing: Yang Fang, Bo-Wen Yue.

Conceptualization: Bowen Yue, Yang Fang

Data curation: Bowen Yue, Yang Fang

Formal analysis: Bowen Yue, Yang Fang

Funding acquisition: Bowen Yue, Yang Fang

Investigation: Bowen Yue, Yang Fang

Methodology: Han-bo Ma, Yang Fang, Yi-Peng Yuan

Project administration: Han-bo Ma, Yang Fang, Yi-Peng Yuan

Resources: Han-bo Ma, Yang Fang

Software: Yang Fang

Supervision: Yang Fang

Validation: Yang Fang

Visualization: Yang Fang

Writing – original draft: Bowen Yue, Yang Fang

Writing – review & editing: Bowen Yue, Yang Fang

## Supplementary Material


