# Supplementary material for: Acupuncture and moxibustion for chronic fatigue syndrome: A systematic review and network meta-analysis
Source: Medicine (Baltimore). 2022 Aug 5;101(31):e29310. doi: 10.1097/MD.0000000000029310 (PMC9351926; doi:10.1097/MD.0000000000029310)
Supplement: Supplementary file 1 [file medi-101-e29310-s001.docx]

**see Table, Supplemental Content 1, which illustrates the general information of each therapy**

|  | Average age | Average course of disease |
| --- | --- | --- |
| Acupuncture + Moxibustion | **37.94 years** | **22.34 months** |
| Acupuncture | **38.78 years** | **21.96 months** |
| Moxibustion | **40.22 years** | **14.14 months** |
| Acupuncture + THM | **36.95 years** | **31.28 months** |
| Moxibustion + THM | **40.36 years** | **58.32 months** |
| Traditional Chinses herbal medicine | **38.02 years** | **23.76 months** |
| Western medicine | **39.54 years** | **17.29 months** |
| No control | **35.67 years** | **26.44 months** |
