# Supplementary material for: Acupuncture and moxibustion for chronic fatigue syndrome: A systematic review and network meta-analysis
Source: Medicine (Baltimore). 2022 Aug 5;101(31):e29310. doi: 10.1097/MD.0000000000029310 (PMC9351926; doi:10.1097/MD.0000000000029310)
Supplement: Supplementary file 2 [file medi-101-e29310-s002.docx]

**see Table, Supplemental Content 2, which illustrates the characteristics of Included studies (RCTs)**

| First author(year) | Diagnosis | Participants | | | Interventions | | Outcome measures |
| --- | --- | --- | --- | --- | --- | --- | --- |
|  |  | Number(male/female) | Age(mean±SD or range) | Disease duration(mean±SD or range | Experimental group(E) | Control group(C) |  |
| Xiao (2014) | **1994CDC** | **80**  **(39/41)** | **E: 40.2±8.2 years**  **C: 40.6±8.5 years** | **E: 0.5~2years**  **C: 0.6~2years** | **Mox: semi-fixed points; mainpoints(bilateral BL13、BL15、BL18、BL20、BL23) and additional points( according to TCM syndrome differentiation); 15min/ session, 30 sessions for 5 weeks** | **WM: *Vitamin B1、Vitamin B6、Oryzanol、Paroxetine*; 5 weeks** | **FS-14；SF-36；adverse reaction；overall response rate** |
| Li  (2018) | **1994CDC** | **60**  **(24/36)** | **E: 38.4±3.1 years**  **C: 38.1±3.2 years** | **E: 6.10±18.30 months**  **C: 6.09±18.17 months** | **Mox: fixed points(bilateral ST36)；15min/session, 20 sessions for 4 weeks** | **THM: *Liuwei Dihuang pill;* one dose daily for 4 weeks** | **FS-14;overall response rate;CFS clinical symptom score** |
| Xing  (2019) | **1994**  **CDC** | **80**  **(28/52)** | **E: 32.23±10.35 years**  **C: 36.98±11.38 years** | **Not reported** | **MT: *Shenqi Zhencao decoction* combined with moxibustion(CV4);moxibustion 30min/session, THM one dose daily; 20 sessions for 4 weeks** | **Mox: fixed point(CV4); 30min/session,20 sessions for 4 weeks** | **FASA;overall response rate** |
| Qi  (2017) | **1994**  **CDC** | **60**  **(36/24)** | **E: 38±9 years**  **C: 37±11 years** | **E: 27.7±5.8 months**  **C: 26.5±4.5 months** | **AT: *Chaihu Longgu Muli Decoction* combined with acupuncture(semi-fixed points; main points (bilateral BL13、BL15、BL18、BL20、BL23) and additional points( according to TCM syndrome differentiation); de qi required; acupuncture 30min/session, THM one dose daily; 36 sessions for 5 weeks** | **THM: *Chaihu Longgu Muli Decoction;* one dose daily for 5 weeks** | **FS-14、SAS** |
| Huang  (2017) | **1994**  **CDC** | **60**  **(29/31)** | **E: 39.70±9.86 years**  **C: 41.73±9.33 years** | **E: 14.13±3.12 months**  **C: 13.97±3.23 months** | **Acu: fixed points(GV20、GV29; bilateral HT7、KI3、LR3、SP6、ST36); de qi required; 40min/session, 16 sessions for 2 months** | **WM: *Vitamin B1、Oryzanol, once daily for 2 months*** | **FAI;FS-14;SAS;SDS;overall response rate** |
| Hu  (2013) | **1994 CDC** | **60**  **(25/35)** | **E: 37.36±8.55 years**  **C: 37.20±6.93 years** | **E: 38.27±8.73 months**  **C: 39.30±7.55 months** | **AT: acupuncture combined with THM; fixed points(GV20、CV4、CV6;bilateral BL15、BL17、BL18、BL20、PC6、LI4、ST36、LR3); de qi required; 30min/session, 12 sessions for 4 weeks;**  ***Liuwei Dihuang pill*; three times daily for 4 weeks** | **THM: *Liuwei Dihuang pill*; three times daily for 4 weeks** | **FAI;WHOQOL-BREF;overall response rate; CFS clinical symptom score** |
| Xiong  (2005) | **1994**  **CDC** | **87**  **(25/62)** | **E: 18~50years**  **C: 18~50years** | **E: 6~12 months**  **C: 6~12months** | **Acu: fixed points(bilateral BL13、BL15、BL18、BL20、BL23); de qi required; 30min/session, 20 session for 4 weeks** | **THM: *Liuwei Dihuang pill*; twice daily for 4 weeks** | **Overall response rate;FS-14;SCL-90; adverse reaction** |
| Xu  (2014) | **1994**  **CDC** | **95**  **(51/44)** | **E:**  **AT 20~50 years**  **C：**  **THM 20~50 years**  **WM 20~50 years** | **E: AT 0.5~3 years**  **C: THM 0.5~3 years**  **WM 0.5~3 years** | **AT: fixed points(GV14、BL43、GV4、CV6、ST36); de qi required; 15min/session, 20 sessions for 4 weeks; *Qingshu Yiqi decoction*; once daily for 4 weeks** | **THM: *Qingshu Yiqi decoction*; once daily for 4 weeks**  **WM: *oryzanol*、*diazepam*; symptomatic treatment** | **Overall response rate** |
| Hou  (2017) | **1994**  **CDC** | **54**  **(30/24)** | **E: 43.04±9.31 years**  **C: 45.62±9.92 years** | **E: 52.54±15.23 days**  **C: 48.35 ±16.93 days** | **Mox: fixed points(GV14~GV2); 15min/session,6 sessions for 3 months** | **Acu: semi-fixed points(GV29、GV20; bilateral KI3、HT7、SP6、LR3) and additional points( according to TCM syndrome differentiation); 30min/session, 48 sessions for 3 months** | **FS-14; CFS clinical symptom score;overall response rate; adverse reaction** |
| Xu  (2019) | **1994**  **CDC** | **94**  **(56/38)** | **E: 41.5±5.3 years**  **C: 42.5±3.6 years** | **Not reported** | **Mox: fixed points(GV14~GV2); 15min/session,6 sessions for 3 months** | **Acu: semi-fixed points; main points(GV29; bilateral KI3、HT7、SP6、LR3) and additional points( according to TCM syndrome differentiation); 30min/session, 48 sessions for 3 months** | **Overall response rate;FS-14; CFS clinical symptom score** |
| Liu  (2018) | **1994 CDC** | **88**  **(19/69)** | **E: AM 37.39±8.47 years**  **C: Mox 37.10±7.36 years**  **Acu 36.60±8.07 years** | **E: AM 18.29±5.81 months**  **C: Mox 15.93±5.71 months**  **Acu 16.73±5.74 months** | **AM：Mox fixed points (GV14~GV2); 40min/session,8 sessions for 4 weeks；Acu fixed points(GV20、CV4、BL20、BL18、BL23、ST36、SP6）；de qi required 30min/session, 8 sessions for 4 weeks** | **Mox: fixed points (GV14~GV2); 40min/session,8 sessions for 4 weeks**  **Acu: fixed points(GV20、CV4、BL20、BL18、BL23、ST36、SP6）;de qi required; 30min/session, 8 sessions for 4 weeks** | **Overall response rate;FS-14;FAI** |
| Shi  (2015) | **1994**  **CDC** | **59**  **(17/42)** | **E: 39.00±12.54 years**  **C: 41.62±11.70 years** | **E： 13.00±4.94 months**  **C： 13.42±4.97 months** | **Mox：fixed points（GV14~GV2；BL11~BL26）；30min/session，12 sessions for 6 weeks** | **Acu：semi-fixed points; main points(GV20、PC6、CV4、ST36、KI3、BL20、BL23、GV4) and additional points( according to TCM syndrome differentiation)； de qi required; 30min/session, 18 sessions for 6 weeks** | **FS-14;SAS;overall response rate; adverse reaction** |
| Luo Dai Hong  (2019) | **1994**  **CDC** | **60**  **(31/29)** | **E: 43±4 years**  **C: 42±3 years** | **E: 11.9±4.4 months**  **C: 11.1±3.7 months** | **Mox: fixed points（GV14~GV2；BL11~BL26）；30min/session, 30 sessions for 2 months** | **Acu: fixed points(GV20、CV6、CV4；bilateral BL20、BL23、ST36、PC6); de qi required;30min/session, 30 sessions for 2 months** | **FS-14;overall response rate; adverse reaction** |
| Zhou  (2018) | **1994 CDC** | **96**  **(30/66)** | **E: 35.2±8.5 years**  **C: 34.7±8.3 years** | **E: 1.0±0.4 years**  **C: 1.1±0.4 years** | **AM: Acu fixed points(CV12、CV10、CV6、CV4; bilateral ST24、ST26、SP15、KI13); de qi required; 30min/session,20 sessions for 6 weeks;**  **Mox fixed points(CV6、CV4); 30min/session,20 sessions for 6 weeks** | **Acu: fixed points(CV12、CV10、CV6、CV4; bilateral ST24、ST26、SP15、KI13); de qi required; 30min/session,20 sessions for 6 weeks** | **FS-14;overall response rate** |
| Zhu  (2012) | **1994 CDC** | **60**  **(25/35)** | **E: 26.98±9.54 years**  **C: 28.58±12.67 years** | **E: 18.72±14.00 months**  **C: 20.08±16.11 months** | **Acu: semi-fixed points; main points(CV12、CV10、CV6、CV4、ST25、ST26、ST24、CV9) and additional points( according to TCM syndrome differentiation); 30min/session, 10 sessions for 3 weeks** | **THM: Chinses herb decoctions according to syndrome differentiation; 10 days** | **FSS;HAMA;VAS;HAMD;overall response rate; adverse reaction** |
| Tian  (2015) | **1994 CDC** | **72**  **(40/32)** | **E: 42±9 years**  **C: 42±10 years** | **Not reported** | **Mox: fixed points(BL43、CV6、ST36);30min/session,30 sessions for 1 month** | **Acu: fixed points(BL43、CV6、ST36); de qi required;30min/session,30 sessions for 1 month** | **FAI;overall response rate; adverse reaction** |
| Jiang  (2015) | **1994 CDC** | **60**  **(24/36)** | **E: 37±9 years**  **C: 39±11 years** | **Not reported** | **MT:**  **Mox fixed points(CV12、CV4、CV6、ST36、PC6)；15min/session, 18 sessions for 3 weeks**  **THM *Bazhen decoction*; one dose daily for 3 weeks** | **Acu: fixed points(CV20、CV29、TF4、PC6、LR3、ST36、SP6)；de qi required; 30min/ session, 18 sessions for 3 weeks** | **FS-14;overall response rate** |
| Guo  (2016) | **1994 CDC** | **60**  **(29/31)** | **E: 45.48±8.32 years**  **C：46.31±7.95 years** | **E： 58.32±10.65 months**  **C: 60.58±11.57 months** | **MT:**  **Mox fixed points(CV6; bilateral ST36、BL20)；15min/session, 20 sessions for 1 month**  **THM *Bazhen decoction; one dose daily for 1 month*** | **THM: *Bazhen decoction; one dose daily for 1 month*** | **Overall response rate;FS-14; adverse reaction** |
| Zhao  (2014) | **1994 CDC** | **60**  **(19/41)** | **E: 40.80±6.60 years**  **C：41.07±5.78 years** | **E: 11.57±3.58 months**  **C: 11.13±2.98 months** | **Mox: fixed points(CV4；bilateral BL20、BL23、ST36); 15min/session, 20 sessions for 4 weeks** | **Acu: fixed points(GV20、CV4、BL18、BL20、BL23、ST36、CV17); de qi required; 30min/session, 20 sessions for 4 weeks** | **FS-14;SDS;SAS;overall response rate** |
| Luo Wen  (2019) | **1994 CDC** | **90**  **(32/58)** | **E: 37.58±6.48 years**  **C: 38.25±6.26 years** | **E: 10.42±3.22 months**  **C: 11.06±3.18 months** | **AM:**  **Acu fixed points(CV13、CV12、CV10、CV4、CV6；bilateral ST25、ST36、SP6); de qi required; 30min/session, 28 sessions for 4 weeks;**  **Mox fixed point(CV8)；6min/session, 28 sessions for 4 weeks** | **Acu: fixed points(CV13、CV12、CV10、CV4、CV6；bilateral ST25、ST36、SP6); de qi required; 30min/session, 28 sessions for 4 weeks;** | **FSS;SF-36;overall response rate** |
| Zheng  (2014) | **1994 CDC** | **86**  **(44/42)** | **E: 43.5±13.2 years**  **C: 42.6±12.9 years** | **E: 2.85±0.89 years**  **C: 2.82±0.92 years** | **Mox: fixed points(bilateral ST36、GB39); de qi required; 5min/session, 21 sessions for 4 weeks** | **Acu: semi-fixed points; main points(GV20、GV29、TF4、KI3、LR3、ST36、SP6) and additional points( according to TCM syndrome differentiation); de qi required; 30min/sessions, 21 sessions for 4 weeks** | **Overall response rate; adverse reaction** |
| Wang  (2013) | **1994 CDC** | **80**  **(25/55)** | **E: 39±6 years**  **C: 38±8 years** | **E: 23.6±2.9 months**  **C: 25.1±3.3 months** | **Mox: fixed points(GV20、EX-HN1); 1min/session, 20 sessions for 3 weeks** | **WM: Fluoxetine hydrochloride; 20mg/d for 3 weeks** | **Overall response rate; adverse reaction** |
| Sui  (2015) | **1994 CDC** | **60**  **(27/33)** | **E: 44.2±4.2 years**  **C: 45.3±5.1 years** | **E: 9.7±2.5 months**  **C: 10.1±3.1 months** | **Mox: fixed points(CV4、CV6、GV4； bilateral BL20、BL15、PC6、ST36); 30min/session, 56 sessions for 2 weeks** | **THM: *Guipi pill;* 10 pills/bid for 2 months** | **Overall response rate** |
| Hao  (2013) | **1994 CDC** | **59**  **(24/35)** | **E: 35.07±7.77 years**  **C: 37.07±8.00 years** | **E: 26.53±14.29 months**  **C: 23.79±14.26 months** | **AM:**  **Acu semi-fixed points;main points and additional points( according to TCM syndrome differentiation); de qi required; 20min/session, 24 sessions for 8 weeks**  **Mox fixed point(CV8); 20min/session, 24 sessions for 8 weeks** | **Acu: semi-fixed points;main points(GV20、GV29; bilateral TF4、KI3、LR3、SP6) and additional points( according to TCM syndrome differentiation); de qi required; 30min/session, 24 sessions for 8 weeks** | **FS-14;overall response rate; adverse reaction** |
| Li Wei Wei  (2016) | **1994 CDC** | **60**  **(34/26)** | **E: 32~68 years**  **C: 30~60 years** | **Not reported** | **Acu: semi-fixed points; main points and additional points( according to TCM syndrome differentiation); de qi required; 30min/session, 30 sessions for 1 month;** | **THM: *Buzhong Yiqi decoction* combined with *Liuwei Dihuang pill*; one dose daliy for 1 month** | **FS-14;overall response rate;SDS** |
| Lin  (2010) | **1994 CDC** | **60**  **(19/41)** | **E: 38.27±7.49 years**  **C: 39.77±5.58 years** | **Not reported** | **AM: fixed points(bilateral BL13、BL15、BL18、BL20、BL23);de qi required; 30min/session, 15 sessions for 3 weeks** | **THM: Guipi pill; 6g/tid for 3 weeks** | **FS-14;FSS;overall response rate; adverse reaction** |
| Song  (2016) | **1994 CDC** | **57**  **(20/37)** | **E: 35.06±1.35 years**  **C: 35.50±1.40 years** | **E: 17.56±0.48 months**  **C: 17.30±0.44 months** | **AM: fixed points(BL23、BL18、BL20、ST36、SP6、GV20); de qi required; 30min/session, 30 sessions for 1 month** | **THM: *Yougui pill*; 9g/tid for 1 month** | **FS-14;overall response rate; adverse reaction** |
| Liu You  Bao  (2017) | **1994 CDC** | **70**  **(23/47)** | **E: 40.6±8.5 years**  **C: 40.5±8.4 years** | **E: 14.5±6.7 months**  **C: 14.4±6.7 months** | **Acu: fixed points(BL13、BL15、BL18、BL20、BL23、ST36、SP9、SP6、CV4、CV6、CV12、CV10); de qi required; 30min/session** | **AM: fixed points(BL13、BL15、BL18、BL20、BL23、ST36、SP9、SP6、CV4、CV6、CV12、CV10); de qi required; 30min/session** | **Overall response rate** |
| Zhong  (2014) | **1994 CDC** | **60**  **(21/39)** | **E: 41±9 years**  **C: 41±8 years** | **E: 14.32±6.33 months**  **C: 15.42±6.05 months** | **AM:**  **Acu fixed points(bilateral BL13、BL15、BL18、BL20、BL23、ST36、SP6、SP9; CV12、CV10、CV6、CV4); de qi required;**  **Mox fixed points; 30min/session, 20 sessions for 3 weeks** | **Acu: fixed points(bilateral BL13、BL15、BL18、BL20、BL23、ST36、SP6、SP9; CV12、CV10、CV6、CV4); de qi required; 30min/session, 20 sessions for 3 weeks** | **Overall response rate** |
| Zhou  (2013) | **1994 CDC** | **99**  **(46/53)** | **E: AM 40.83±9.13 years**  **C: Acu 43.86±5.93 years**  **THM 41.32±7.02 years** | **E: AM 13.57±4.51 months**  **C: Acu 12.31±1.03 months**  **THM 14.81±2.78 months** | **AM: semi-fixed points; main points(BL15、BL20、ST36、SP6、GV20) and additional points( according to TCM syndrome differentiation); de qi required; 30min/session, 30 sessions for 6 weeks** | **Acu: semi-fixed points: main points(BL15、BL20、ST36、SP6、GV20) and additional points( according to TCM syndrome differentiation); de qi required; 30min/session, 30 sessions for 6 weeks**  **THM: *Guipi decoction*; one dose daliy for 6 weeks** | **Overall response rate;FS-14;SAS;WHOQOL-BREF; adverse reaction** |
| Liu Chun  (2017) | **1994 CDC** | **60**  **(26/34)** | **E: 37.58±12.36 years**  **C: 42.37±14.45 years** | **E: 11.24±4.07 months**  **C: 10.78±5.12 months** | **Mox: semi-fixed points; main points(GV29、GV20、GV24、GV14、GB20、BL23、KI3、EX-HN5) and additional points( according to TCM syndrome differentiation);30min/session, 48 sessions for 8 weeks** | **WH: *Oryzanol*; 20mg/tid for 8 weeks** | **FS-14;overall response rate;SAS;SDS;PSQI** |
| Zheng  (2012) | **1994 CDC** | **77**  **(31/46)** | **E: 38.73±4.11 years**  **C: 37.08±5.32 years** | **E: 18.41±5.34 months**  **C: 17.12±6.03 months** | **Acu: semi-fixed points;main points and additional points( according to TCM syndrome differentiation); de qi required;30min/session, 20 sessions for 4 weeks** | **NC: Streitberger’s placebo**  **Acupuncture*** | **FS-14;VAS;DSI** |
| An  (2014) | **1994 CDC** | **80**  **(35/45)** | **E: 36.49±4.12 years**  **C: 37.08±4.69 years** | **Not reported** | **Acu: semi-fixed points;main points and additional points( according to TCM syndrome differentiation); de qi required;30min/session, 20 sessions for 4 weeks** | **NC: Streitberger’s placebo**  **Acupuncture*** | **FS-14;VAS;DSI** |
| Sai  (2018) | **1994 CDC** | **62**  **(30/32)** | **E: 38.97±6.98 years**  **C: 37.73±6.26 years** | **E: 15.72±5.20 months**  **C: 15.23±5.40 months** | **Mox: fixed points(BL13~BL28);10 min/session, 24 seesions for 8 weeks** | **Acu: fixed points(BL12~BL28); de qi required; 30min/session, 24 sessions for 8 weeks** | **FS-14;overall response rate** |
| Zheng  (2013) | **1994 CDC** | **59**  **(19/40)** | **E: 42±6 years**  **C: 43±6 years** | **E: 21.72±6.04 months**  **C: 22.48±5.64 months** | **AM:**  **Acu fixed points(BL20、BL23**  **LR13、GB25); de qi required; 30min/session, 20 sessions for 4 weeks;**  **Mox fixed points(GV14~GV2); 60min/session, 4 sessions for 4 weeks** | **Acu: fixed points(BL20、BL23**  **LR13、GB25); de qi required; 30min/session, 20 sessions for 4 weeks** | **FS-14;VAS;DSI;overall response rate** |
| Li Rong Zhen  (2016) | **1994 CDC** | **62**  **(30/32)** | **E: 30.77±6.98 years**  **C: 28.94±6.26 years** | **E: 28.10±9.71 months**  **C: 26.75±8.30 months** | **Acu: semi-fixed points; main points(EX-HN1、GV24、GV29; bilateral PC6、SP6); de qi required; 30min/session, 18 sessions for 6 weeks** | **NC: Usual care** | **FAI;FS-14;SAS;SDS;overall response rate** |
| Yu  (2013) | **1994 CDC** | **60**  **(15/45)** | **E: 30~50 years**  **C: 30~50 years** | **E: 0.5~20 years**  **C: 0.5~20 years** | **Acu: fixed points(BL20、BL18; bilateral LI4、ST36、LR3、SP9); de qi required; 30min/session, 15 sessions for 3 weeks** | **THM: *Xiaoyao decoction*; one dose daily for 3 weeks** | **Overall response rate** |
| Zhang  (2007) | **1994 CDC** | **50**  **(29/21)** | **E: 24~61 years**  **C: 18~57 years** | **E: 1~6 years**  **C: 0.8~5 years** | **Acu: semi-fixed points; main points(BL18、BL15、BL20、BL23) and additional points( according to TCM syndrome differentiation); de qi required;30min/session, 20 sessions for 4 weeks** | **NC: Streitberger’s placebo**  **Acupuncture*** | **FS-14;overall response rate** |
| Wang  (2009) | **1994 CDC** | **64**  **(32/32)** | **E: 35.8±10.7 years**  **C: 38.8±8.8 years** | **Not reported** | **Acu: fixed points(GV20、CV17、CV12、CV4、CV6、LI4、ST36、SP6、LR3、KI3、BL20、BL18、BL23); de qi required; 30min/session, 14 sessions for 6 weeks** | **NC: Needling non-acupuncture points; de qi not required*** | **FS-14; adverse reaction** |
| Chen  (2010) | **1994 CDC** | **53**  **(23/30)** | **E:37.47±12.21 years**  **C: 39.85±13.47 years** | **Not reported** | **Acu: fixed points(ST36、KI3); de qi required; 30min/session, 6 sessions for 2 weeks** | **NC: Streitberger’s placebo**  **Acupuncture*** | **WHOQOL-BREF;FS-14;overall response rate; adverse reaction** |
| Xu  (2016) | **1994 CDC** | **62**  **(35/27)** | **E: 20~55 years**  **C: 20~55 years** | **E: 21.31±2.83 months**  **C: 22.87±2.37 months** | **Acu: semi-fixed points; main points(BL18、BL20、LR3、SP6、GV20、ST36) and additional points( according to TCM syndrome differentiation); de qi required;30min/session, 20 sessions for 6 weeks** | **THM: *Xiaoyao decoction*; one dose daily for 3 weeks** | **FAI;SDS;SF-36;overall response rate** |
| Lu  (2014) | **1994 CDC** | **133**  **(60/73)** | **E: Acu 20~55 years**  **C: AM 19~53 years**  **NC 18~54 years** | **E: Acu 1~5 years**  **C: AM 0.8~6 years**  **NC 1.2~5.3 years** | **Acu: fixed points(GV20、CV17、CV4、CV6; bilateral ST36、LI4、LR3、SP6); de qi required; 30min/session, 20 sessions for 3 weeks** | **AM: Acu fixed points(GV20、CV17、CV4、CV6; bilateral ST36、LI4、LR3、SP6); de qi required;**  **Mox fixed points(GV20、CV4、CV6、ST36); 30min/session, 20 sessions for 3 weeks**  **NC: Needling non-acupuncture points; de qi not required*** | **FS-14;** |
| Wang  (2018) | **1994 CDC** | **48**  **(15/33)** | **E: 37.3±7.5 years**  **C: 36.5±6.9 years** | **E: 18.5±6.2 months**  **C: 19.4±7.6 months** | **AM: Acu fixed points(CV4、CV6; bilateral ST25、ST36、KI3、LR3); de qi required;**  **Mox fixed point(CV4、CV6、CV8);**  **30min/session; 16 sessions for 2 months** | **Acu: Acu fixed points(CV4、CV6; bilateral ST25、ST36、KI3、LR3); de qi required; 30min/session; 16 sessions for 2 months** | **Overall response rate;FAI; adverse reaction** |
| Chen  (2018) | **1994 CDC** | **60**  **(20/40)** | **E: 40.77±11.61 years**  **C: 41.47±12.21 years** | **E: 18.70±9.22 months**  **C: 17.83±9.01 months** | **Acu: fixed points(bilateral BL13、BL15、BL18、BL20、BL23); de qi required; 20min/session, 8 sessions for 4 weeks** | **NC: Needling non-acupuncture points; de qi not required*** | **FS-14;SCL-90;overall response rate; adverse reaction** |
| Ding  (2011) | **1994 CDC** | **60**  **(35/25)** | **E: 18~65 years**  **C: 18~65 years** | **E: 0.5~3 years**  **C: 0.5~3 years** | **Acu: fixed points(bilateral BL13、BL15、BL18、BL20、BL23); de qi required; 30min/session, 18 sessions for 6 weeks** | **NC: Needling non-acupuncture points; de qi not required*** | **FS-14;SAS;SDS;overall response rate** |
| Ye  (2009) | **1994 CDC** | **60**  **(17/43)** | **E: 20~50 years**  **C: 18~50 years** | **E: 0.6~20 years**  **E: 0.6~20 years** | **Acu: fixed points(BL20、BL18、SP9、ST36；bilateral LI4、LR3); de qi required; 30min/session, 15 sessions for 3 weeks** | **THM: *Xiaoyao decoction*; one dose daily for 3 weeks** | **Overall response rate** |
| Yang  (2019) | **1994 CDC** | **57**  **(22/35)** | **E: 37.42±9.21 years**  **C: 35.79±10.03 years** | **E: 38.13±14.04 months**  **C: 37.29±15.12 months** | **AT: Acu fixed points(CV12; bilateral BL18、BL20、ST36、SP9、SP6、LR3、HT7); de qi required;30min/session, 20 sessions for 4 weeks**  **THM *Xiaoyao pill*; 8 pills/bid for 4 weeks** | **THM: *Xiaoyao pill*; 8 pills/bid for 4 weeks** | **FS-14;SF-36;overall response rate; adverse reaction** |
| Yang  (2017) | **1994 CDC** | **15**  **(0/15)** | **E: 24.75±3.77 years**  **C: 23.57±3.10 years** | **Not reported** | **Acu: fixed points(BL18、BL20、BL23、ST36、GV20、HT7); de qi required; 30min/session, 16 sessions for 4 weeks** | **NC: Streitberger’s placebo**  **Acupuncture*** | **FS-14;SPHERE; adverse reaction** |
| Liang  (2016) | **1994 CDC** | **59**  **(20/39)** | **E: 31.61±7.60 years**  **C: 30.54±6.87 years** | **E: 27.39±7.18 months**  **C: 28.57±7.45 months** | **AM: Acu fixed points(CV4、CV6、CV12; bilateral ST25、ST36、LI4、SP6); de qi required; 30min/session**  **Mox fixed points(CV4、CV6); 10min/session**  **16 sessions for 4 weeks** | **NC: Usual care** | **FAI;FS-14;SAS;PSQI;overall response rate; adverse reaction** |
| Guan  (2017) | **1994 CDC** | **66**  **(21/45)** | **E: 40.6±9.2 years**  **C: 41.4±9.6 years** | **E: 14.2±6.2 months**  **C: 14.6±5.8 months** | **AM: fixed points(CV4、CV6; bilateral BL13、BL15、BL20、BL23、CV12、CV10、GB34、ST36、SP6); de qi required; 30min/session, 10 sessions for 3 weeks** | **Acu: fixed points(CV4、CV6; bilateral BL13、BL15、BL20、BL23、CV12、CV10、GB34、ST36、SP6); de qi required; 30min/session, 10 sessions for 3 weeks** | **Overall response rate** |
| Wu  (2015) | **1994 CDC** | **100**  **(38/62)** | **E: 40.7±6.0 years**  **C: 40.5±5.0 years** | **E: 5.25±1.34 years**  **C: 5.75±1.50 years** | **AM: fixed points(bilateral BL13、BL15、BL18、BL20、BL23、ST36、SP6、SP9、CV12、CV10、CV6、CV4); de qi required; 30min/session, 10 sessions for 3 weeks** | **Acu: fixed points(bilateral BL13、BL15、BL18、BL20、BL23、ST36、SP6、SP9、CV12、CV10、CV6、CV4); de qi required; 30min/session, 10 sessions for 3 weeks** | **Overall response rate; adverse reaction** |

*The points and treatment course in the NC group were same as in the acupuncture group.

Acronyms: Acu, acupuncture; Mox, moxibustion; AM, acupuncture combined with moxibustion; AT, acupuncture combined with Chinses herbal medicine; MT, moxibustion combined with Chinses herbal medicine; THM, Traditional chinses herbal medicine; WM, western medicine; NC, no control; CDC, Centres for Disease Control and Prevention; FS-14, Fatigue Scale-14; DSI, Depression Status Inventory; FSS, Fatigue Severity Scale; SAS, Self Rating Anxiety Scale; SF-12, SF-20, SF-36: 12, 20, 36-item short-form health survey; VAS, Visual Analogue Scale; WHOQOL-BREF, WHO Quality of Life Assessment Instrument brief version; SDS, Self-Rating Depression Scale; FAI, fatigue assessment instrument; PSQI, Pittsburgh sleep quality index; SPHERE, Somatic and Psychological health report; SCL-90, SymptomChecklist-90; HAMD, Hamilton Depression Scale; HAMA, Hamilton Anxiety Scale.
