# Supplementary material for: Acupuncture and moxibustion for chronic fatigue syndrome: A systematic review and network meta-analysis
Source: Medicine (Baltimore). 2022 Aug 5;101(31):e29310. doi: 10.1097/MD.0000000000029310 (PMC9351926; doi:10.1097/MD.0000000000029310)
Supplement: Supplementary file 3 [file medi-101-e29310-s003.docx]

**see Table, Supplemental Content 3, which illustrates the risk of bias table of included studies.**

The risk of bias graph


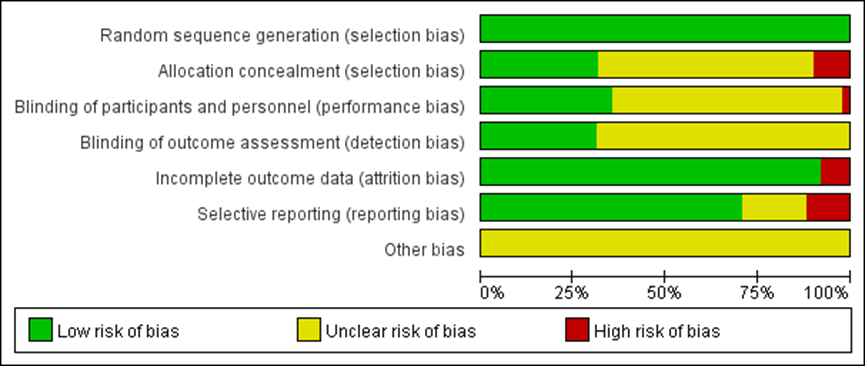


Risk of bias summary

| **Study ID** | **Random sequence generation** | **Allocation concealment** | **Blinding participant and personnel** | **Blinding of outcome assessment** | **Incomplete outcome data** | **Selective reporting** | **Other bias** |
| --- | --- | --- | --- | --- | --- | --- | --- |
| **1.Ding 2011** | **Low risk of bias** | **High risk of bias** | **Unclear risk of bias** | **Unclear risk of bias** | **Low risk of bias** | **Low risk of bias** | **Unclear risk of bias** |
| **2.Yu 2013** | **Low risk of bias** | **Unclear risk of bias** | **Unclear risk of bias** | **Unclear risk of bias** | **Low risk of bias** | **Low risk of bias** | **Unclear risk of bias** |
| **3.Hou 2017** | **Low risk of bias** | **Unclear risk of bias** | **Unclear risk of bias** | **Unclear risk of bias** | **High risk of bias** | **High risk of bias** | **Unclear risk of bias** |
| **4.Liu 2017** | **Low risk of bias** | **Unclear risk of bias** | **Unclear risk of bias** | **Unclear risk of bias** | **Low risk of bias** | **Unclear risk of bias** | **Unclear risk of bias** |
| **5.Liu 2017** | **Low risk of bias** | **Low risk of bias** | **Low risk of bias** | **Low risk of bias** | **Low risk of bias** | **Low risk of bias** | **Unclear risk of bias** |
| **6.Liu 2018** | **Low risk of bias** | **Unclear risk of bias** | **Low risk of bias** | **Unclear risk of bias** | **Low risk of bias** | **Low risk of bias** | **Unclear risk of bias** |
| **7.Lu 2014** | **Low risk of bias** | **Unclear risk of bias** | **Unclear risk of bias** | **Unclear risk of bias** | **Low risk of bias** | **Unclear risk of bias** | **Unclear risk of bias** |
| **8.Ye 2009** | **Low risk of bias** | **Unclear risk of bias** | **Unclear risk of bias** | **Unclear risk of bias** | **Low risk of bias** | **Low risk of bias** | **Unclear risk of bias** |
| **9.Wu 2015** | **Low risk of bias** | **Low risk of bias** | **Low risk of bias** | **Unclear risk of bias** | **Low risk of bias** | **Low risk of bias** | **Unclear risk of bias** |
| **10.Zhou 2013** | **Low risk of bias** | **Low risk of bias** | **Low risk of bias** | **Low risk of bias** | **Low risk of bias** | **Low risk of bias** | **Unclear risk of bias** |
| **11.Zhou 2018** | **Low risk of bias** | **Unclear risk of bias** | **Unclear risk of bias** | **Unclear risk of bias** | **Low risk of bias** | **Low risk of bias** | **Unclear risk of bias** |
| **12.Jiang 2015** | **Low risk of bias** | **Unclear risk of bias** | **Unclear risk of bias** | **Unclear risk of bias** | **Low risk of bias** | **Low risk of bias** | **Unclear risk of bias** |
| **13.An 2014** | **Low risk of bias** | **Unclear risk of bias** | **Unclear risk of bias** | **Unclear risk of bias** | **High risk of bias** | **High risk of bias** | **Unclear risk of bias** |
| **14.Song 2016** | **Low risk of bias** | **Unclear risk of bias** | **Unclear risk of bias** | **Unclear risk of bias** | **Low risk of bias** | **Low risk of bias** | **Unclear risk of bias** |
| **15.Zhang 2007** | **Low risk of bias** | **Low risk of bias** | **Low risk of bias** | **Low risk of bias** | **Low risk of bias** | **Low risk of bias** | **Unclear risk of bias** |
| **16.Xu 2014** | **Low risk of bias** | **Unclear risk of bias** | **Unclear risk of bias** | **Unclear risk of bias** | **Low risk of bias** | **High risk of bias** | **Unclear risk of bias** |
| **17.Xu 2019** | **Low risk of bias** | **Unclear risk of bias** | **Unclear risk of bias** | **Unclear risk of bias** | **Low risk of bias** | **Unclear risk of bias** | **Unclear risk of bias** |
| **18.Xu 2010** | **Low risk of bias** | **Low risk of bias** | **Low risk of bias** | **Low risk of bias** | **Low risk of bias** | **Low risk of bias** | **Unclear risk of bias** |
| **19.Zhu 2012** | **Low risk of bias** | **Low risk of bias** | **Low risk of bias** | **Low risk of bias** | **Low risk of bias** | **Low risk of bias** | **Unclear risk of bias** |
| **20.Li 2016** | **Low risk of bias** | **Unclear risk of bias** | **Unclear risk of bias** | **Unclear risk of bias** | **Low risk of bias** | **Low risk of bias** | **Unclear risk of bias** |
| **21.Li**  **2018** | **Low risk of bias** | **Unclear risk of bias** | **Unclear risk of bias** | **Unclear risk of bias** | **Low risk of bias** | **Low risk of bias** | **Unclear risk of bias** |
| **22.Li 2016** | **Low risk of bias** | **Low risk of bias** | **Unclear risk of bias** | **Unclear risk of bias** | **High risk of bias** | **High risk of bias** | **Unclear risk of bias** |
| **23.Yang 2019** | **Low risk of bias** | **Unclear risk of bias** | **Unclear risk of bias** | **Unclear risk of bias** | **Low risk of bias** | **Low risk of bias** | **Unclear risk of bias** |
| **24.Yang 2017** | **Low risk of bias** | **Low risk of bias** | **Low risk of bias** | **Low risk of bias** | **Low risk of bias** | **Low risk of bias** | **Unclear risk of bias** |
| **25.Lin 2010** | **Low risk of bias** | **Unclear risk of bias** | **Unclear risk of bias** | **Unclear risk of bias** | **Low risk of bias** | **Low risk of bias** | **Unclear risk of bias** |
| **26.Liang 2016** | **Low risk of bias** | **Low risk of bias** | **Low risk of bias** | **Low risk of bias** | **Low risk of bias** | **Low risk of bias** | **Unclear risk of bias** |
| **27.Wang 2013** | **Low risk of bias** | **Unclear risk of bias** | **Unclear risk of bias** | **Unclear risk of bias** | **Low risk of bias** | **Unclear risk of bias** | **Unclear risk of bias** |
| **28.Xiong 2005** | **Low risk of bias** | **Unclear risk of bias** | **Unclear risk of bias** | **Unclear risk of bias** | **Low risk of bias** | **Low risk of bias** | **Unclear risk of bias** |
| **29.Wang 2009** | **Low risk of bias** | **Low risk of bias** | **Low risk of bias** | **Low risk of bias** | **Low risk of bias** | **Low risk of bias** | **Unclear risk of bias** |
| **30.Wang 2018** | **Low risk of bias** | **High risk of bias** | **Unclear risk of bias** | **Unclear risk of bias** | **Low risk of bias** | **Unclear risk of bias** | **Unclear risk of bias** |
| **31.Tian 2015** | **Low risk of bias** | **Low risk of bias** | **Low risk of bias** | **Low risk of bias** | **Low risk of bias** | **Low risk of bias** | **Unclear risk of bias** |
| **32.Shi 2015** | **Low risk of bias** | **High risk of bias** | **Low risk of bias** | **Unclear risk of bias** | **High risk of bias** | **High risk of bias** | **Unclear risk of bias** |
| **33.Qi 2017** | **Low risk of bias** | **Unclear risk of bias** | **Unclear risk of bias** | **Unclear risk of bias** | **Low risk of bias** | **Low risk of bias** | **Unclear risk of bias** |
| **34.Luo 2019** | **Low risk of bias** | **Unclear risk of bias** | **Unclear risk of bias** | **Unclear risk of bias** | **Low risk of bias** | **Unclear risk of bias** | **Unclear risk of bias** |
| **35.Luo 2019** | **Low risk of bias** | **Low risk of bias** | **Low risk of bias** | **Low risk of bias** | **Low risk of bias** | **Low risk of bias** | **Unclear risk of bias** |
| **36.Xiao 2014** | **Low risk of bias** | **Low risk of bias** | **Low risk of bias** | **Low risk of bias** | **Low risk of bias** | **Low risk of bias** | **Unclear risk of bias** |
| **37.Hu 2013** | **Low risk of bias** | **Unclear risk of bias** | **Unclear risk of bias** | **Unclear risk of bias** | **Low risk of bias** | **Low risk of bias** | **Unclear risk of bias** |
| **38.Guan 2107** | **Low risk of bias** | **Unclear risk of bias** | **Unclear risk of bias** | **Unclear risk of bias** | **Low risk of bias** | **High risk of bias** | **Unclear risk of bias** |
| **39.Sai 2018** | **Low risk of bias** | **High risk of bias** | **High risk of bias** | **Unclear risk of bias** | **Low risk of bias** | **Low risk of bias** | **Unclear risk of bias** |
| **40.Zhao 2014** | **Low risk of bias** | **Unclear risk of bias** | **Unclear risk of bias** | **Unclear risk of bias** | **Low risk of bias** | **Low risk of bias** | **Unclear risk of bias** |
| **41.Xing 2019** | **Low risk of bias** | **Unclear risk of bias** | **Unclear risk of bias** | **Unclear risk of bias** | **Low risk of bias** | **Low risk of bias** | **Unclear risk of bias** |
| **42.Zheng 2014** | **Low risk of bias** | **Unclear risk of bias** | **Unclear risk of bias** | **Unclear risk of bias** | **Low risk of bias** | **Unclear risk of bias** | **Unclear risk of bias** |
| **43.Zheng 2012** | **Low risk of bias** | **Unclear risk of bias** | **Unclear risk of bias** | **Unclear risk of bias** | **Low risk of bias** | **Low risk of bias** | **Unclear risk of bias** |
| **44.Zheng 2013** | **Low risk of bias** | **Unclear risk of bias** | **Unclear risk of bias** | **Unclear risk of bias** | **Low risk of bias** | **Unclear risk of bias** | **Unclear risk of bias** |
| **45.Hao 2013** | **Low risk of bias** | **Low risk of bias** | **Low risk of bias** | **Low risk of bias** | **Low risk of bias** | **Low risk of bias** | **Unclear risk of bias** |
| **46.Guo 2016** | **Low risk of bias** | **Low risk of bias** | **Low risk of bias** | **Low risk of bias** | **Low risk of bias** | **Low risk of bias** | **Unclear risk of bias** |
| **47.Zhong 2014** | **Low risk of bias** | **High risk of bias** | **Unclear risk of bias** | **Unclear risk of bias** | **Low risk of bias** | **Unclear risk of bias** | **Unclear risk of bias** |
| **48.Chen 2018** | **Low risk of bias** | **Low risk of bias** | **Low risk of bias** | **Low risk of bias** | **Low risk of bias** | **Low risk of bias** | **Unclear risk of bias** |
| **49.Chen 2010** | **Low risk of bias** | **Unclear risk of bias** | **Unclear risk of bias** | **Low risk of bias** | **Low risk of bias** | **Low risk of bias** | **Unclear risk of bias** |
| **50.Sui 2015** | **Low risk of bias** | **Unclear risk of bias** | **Unclear risk of bias** | **Unclear risk of bias** | **Low risk of bias** | **Low risk of bias** | **Unclear risk of bias** |
| **51.Huang 2017** | **Low risk of bias** | **Low risk of bias** | **Low risk of bias** | **Low risk of bias** | **Low risk of bias** | **Low risk of bias** | **Unclear risk of bias** |
