# Supplementary material for: Acupuncture and moxibustion for chronic fatigue syndrome: A systematic review and network meta-analysis
Source: Medicine (Baltimore). 2022 Aug 5;101(31):e29310. doi: 10.1097/MD.0000000000029310 (PMC9351926; doi:10.1097/MD.0000000000029310)
Supplement: Supplementary file 4 [file medi-101-e29310-s004.docx]

**see Table, Supplemental Content 4, which illustrates the GRADE for the primary outcomes.**

**1 Overall response rate**

**1.1 Contribution of low or moderate RoB comparisons to each network estimate**

**Based on the assessment of RoB for each comparison and the contribution matrix detailing contribution of each direct comparison to all network estimates, the following bar graphs show the percentage of low or moderate RoB contributions for each network estimate.**

**Each bar corresponds to a NMA relative treatment effect and shows how much information comes from comparisons at low risk of bias [green] or moderate risk of bias [yellow].**

**
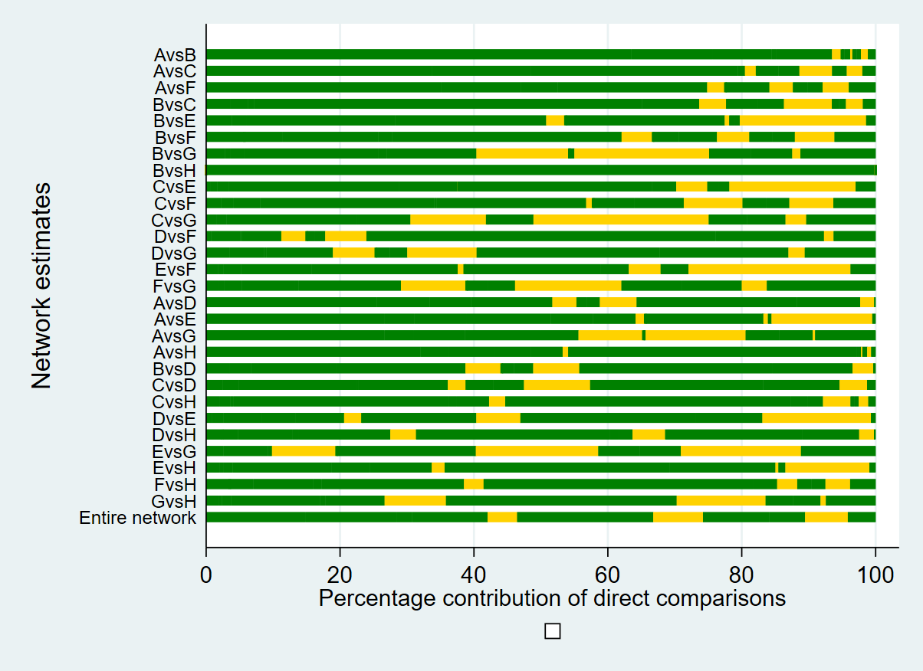
**

|  | P1 | P2 | P3 | P4 | P5 | P6 | P7 | P8 | P9 | P10 | P11 | P12 | P13 | P14 | P15 |
| --- | --- | --- | --- | --- | --- | --- | --- | --- | --- | --- | --- | --- | --- | --- | --- |
| AvsB | 56.2 | 7.3 | 12.1 | 8.9 | 0.7 | 8.4 | 1.5 | 0.0 | 0.4 | 0.8 | 0.3 | 0.6 | 0.6 | 1.0 | 1.2 |
| AvsC | 33.8 | 6.4 | 8.3 | 30.9 | 0.9 | 0.2 | 1.8 | 0.0 | 3.2 | 3.1 | 4.9 | 1.1 | 1.1 | 2.3 | 2.0 |
| AvsF | 30.6 | 4.6 | 11.9 | 5.5 | 1.2 | 21.1 | 2.7 | 0.0 | 2.7 | 3.9 | 3.5 | 2.2 | 2.2 | 3.9 | 4.0 |
| BvsC | 3.6 | 2.7 | 0.9 | 56.0 | 2.0 | 8.5 | 4.2 | 0.0 | 4.5 | 4.0 | 7.2 | 1.1 | 1.1 | 2.5 | 1.9 |
| BvsE | 3.0 | 0.9 | 2.1 | 22.3 | 8.6 | 13.9 | 2.9 | 0.0 | 23.2 | 0.6 | 0.7 | 0.8 | 0.8 | 18.9 | 1.4 |
| BvsF | 5.7 | 0.0 | 5.7 | 14.3 | 2.1 | 34.3 | 4.7 | 0.0 | 3.8 | 5.7 | 4.8 | 3.4 | 3.4 | 5.9 | 6.1 |
| BvsG | 2.8 | 0.8 | 2.1 | 20.1 | 1.2 | 13.4 | 13.9 | 0.0 | 0.0 | 0.7 | 20.1 | 6.2 | 6.2 | 1.2 | 11.2 |
| BvsH | 0.0 | 0.0 | 0.0 | 0.0 | 0.0 | 0.0 | 0.0 | 100.0 | 0.0 | 0.0 | 0.0 | 0.0 | 0.0 | 0.0 | 0.0 |
| CvsE | 0.6 | 1.1 | 1.7 | 17.5 | 8.0 | 8.9 | 0.0 | 0.0 | 28.8 | 3.7 | 4.6 | 1.6 | 1.7 | 18.9 | 3.0 |
| CvsF | 2.3 | 1.8 | 4.0 | 26.0 | 0.4 | 22.3 | 1.0 | 0.0 | 6.2 | 7.3 | 8.8 | 3.5 | 3.5 | 6.6 | 6.3 |
| CvsG | 0.4 | 1.2 | 1.5 | 19.5 | 0.2 | 7.8 | 11.5 | 0.0 | 3.3 | 3.6 | 26.2 | 5.7 | 5.7 | 3.1 | 10.4 |
| DvsF | 0.8 | 0.3 | 1.0 | 3.1 | 0.2 | 5.9 | 3.8 | 0.0 | 1.2 | 1.6 | 6.1 | 52.2 | 16.2 | 1.4 | 6.3 |
| DvsG | 1.3 | 0.5 | 1.7 | 5.2 | 0.3 | 10.0 | 6.4 | 0.0 | 2.1 | 2.6 | 10.4 | 27.3 | 19.2 | 2.4 | 10.6 |
| EvsF | 1.8 | 0.8 | 2.6 | 10.6 | 7.0 | 14.8 | 1.1 | 0.0 | 20.3 | 4.2 | 4.8 | 2.1 | 2.1 | 24.2 | 3.7 |
| FvsG | 1.9 | 0.7 | 2.7 | 7.9 | 0.5 | 15.3 | 9.8 | 0.0 | 3.2 | 4.0 | 15.9 | 9.0 | 9.0 | 3.7 | 16.2 |
| AvsD | 22.1 | 3.4 | 7.9 | 5.4 | 0.7 | 12.1 | 3.8 | 0.0 | 1.3 | 2.0 | 5.5 | 23.9 | 9.5 | 2.1 | 0.2 |
| AvsE | 26.7 | 4.4 | 7.9 | 12.6 | 6.3 | 6.4 | 1.4 | 0.0 | 17.6 | 0.0 | 0.7 | 0.3 | 0.3 | 15.0 | 0.5 |
| AvsG | 26.6 | 4.3 | 7.7 | 10.6 | 0.6 | 5.8 | 9.7 | 0.0 | 0.2 | 0.1 | 15.0 | 5.0 | 5.0 | 0.4 | 9.0 |
| AvsH | 32.0 | 4.2 | 6.9 | 5.0 | 0.4 | 4.8 | 0.8 | 43.1 | 0.2 | 0.5 | 0.2 | 0.4 | 0.4 | 0.6 | 0.7 |
| BvsD | 3.3 | 0.2 | 3.2 | 11.4 | 1.3 | 19.4 | 5.4 | 0.0 | 1.8 | 2.9 | 6.9 | 28.9 | 11.9 | 3.1 | 0.4 |
| CvsD | 1.3 | 1.2 | 2.4 | 17.7 | 0.2 | 13.4 | 2.8 | 0.0 | 3.9 | 4.6 | 9.9 | 25.8 | 11.4 | 4.1 | 1.3 |
| CvsH | 2.1 | 1.5 | 0.5 | 32.1 | 1.1 | 4.9 | 2.4 | 42.6 | 2.6 | 2.3 | 4.1 | 0.6 | 0.6 | 1.4 | 1.1 |
| DvsE | 0.8 | 0.4 | 1.3 | 5.8 | 5.0 | 7.2 | 2.7 | 0.0 | 14.9 | 2.1 | 6.6 | 26.1 | 10.0 | 16.2 | 0.7 |
| DvsH | 2.4 | 0.1 | 2.2 | 8.1 | 0.9 | 13.8 | 3.8 | 29.0 | 1.3 | 2.0 | 4.9 | 20.5 | 8.5 | 2.2 | 0.3 |
| EvsG | 0.2 | 0.1 | 0.1 | 2.2 | 6.6 | 0.6 | 9.7 | 0.0 | 20.7 | 0.1 | 18.3 | 6.2 | 6.2 | 17.9 | 11.1 |
| EvsH | 2.0 | 0.6 | 1.4 | 14.8 | 5.7 | 9.2 | 1.9 | 33.6 | 15.4 | 0.4 | 0.4 | 0.5 | 0.5 | 12.5 | 0.9 |
| FvsH | 3.5 | 0.0 | 3.5 | 8.9 | 1.3 | 21.3 | 2.9 | 37.9 | 2.4 | 3.5 | 3.0 | 2.1 | 2.1 | 3.7 | 3.8 |
| GvsH | 1.9 | 0.5 | 1.4 | 13.3 | 0.8 | 8.9 | 9.2 | 34.0 | 0.0 | 0.5 | 13.3 | 4.1 | 4.1 | 0.8 | 7.4 |

| **Compaison** | **AvsB** | **AvsC** | **AvsF** | **BvsC** | **BvsE** | **BvsF** | **BvsG** | **BvsH** | **CvsE** |
| --- | --- | --- | --- | --- | --- | --- | --- | --- | --- |
| **Moderate percent** | **2.8%** | **9%** | **10.1%** | **13.9%** | **22.5%** | **15.4%** | **35.2%** | **0%** | **23.5%** |
| **CvsF** | **CvsG** | **DvsF** | **DvsG** | **EvsF** | **FvsG** | **AvsD** | **AvsE** | **AvsG** | **AvsH** |
| **16.4%** | **40.8%** | **11.3%** | **19.2%** | **30.1%** | **29.4%** | **11.4%** | **17.1%** | **25.1%** | **1.6%** |
| **BvsD** | **CvsD** | **CvsH** | **DvsE** | **DvsH** | **EvsG** | **EvsH** | **FvsH** | **GvsH** | **Entire network** |
| **15.4%** | **16.8%** | **7.9%** | **25.5%** | **10.9%** | **45.9%** | **14.8%** | **9.6%** | **23.3%** | **18.2** |

**1.2 Table of reasons for downgrading**

**Based on all the above information, we GRADEd each network estimate according to the following criteria.**

1. **Study limitations: We downgraded by one level when the contributions from low RoB comparisons were less than 30% and contributions from moderate RoB comparisons were 70% or greater.**

**(2) Imprecision: We considered a clinically meaningful threshold for OR to be 0.80 or 1.25 and downgraded the estimate if the OR point estimate is 1 or more and the lower limit of its CrI is below 0.80; or if the OR point estimate is less than 1 and the upper limit of its CrI is above 1.25.**

**(3) Inconsistency: We rated two concepts, heterogeneity and incoherence (inconsistency), in this domain.**

**For heterogeneity, we looked at the common tau and found that it is low compared to the expected value as reported in the literature (Turner RM et al (2012) Int J Epidemiol, 41, 818-827) , so we did not downgrade any network estimate for heterogeneity. For inconsistency, we looked at the results of side splitting and we downgraded the comparisons with important inconsistency (p<0.05), where we have not downgraded for imprecision (we did not downgrade the same network estimate for both imprecision and inconsistency).**

**(4) Indirectness: We have assured transitivity in our network by limiting the included studies to chronic fatigue syndrome with the diagnostic criteria of "1994 CDC" .We further ran various subgroup analysis, and assured that they did not violate transitivity of the network.**

**(5) Publication bias: The comparison-adjusted funnel plot did not suggest obvious publication bias. However, we cannot completely rule out the possibility that some studies are still missing. Considering that the field of CFS trials in the past has been prone to publication bias, the review team decided by default to downgrade all the included studies for potential publication bias by one level.**

|  | **Study limitations** | **Imprecision** | **Inconsistency** | **Indirectness** | **Publication bias** | **GRADE** |
| --- | --- | --- | --- | --- | --- | --- |
| **AvsB** | **No downgrade** | **No downgrade** | **No downgrade** | **No downgrade** | **Downgrade** | **MODERATE** |
| **AvsC** | **No downgrade** | **Downgrade**  **Because point estimate >1.0**  **but lower limit<0.80** | **No downgrade** | **No downgrade** | **Downgrade** | **LOW** |
| **AvsF** | **No downgrade** | **No downgrade** | **No downgrade** | **No downgrade** | **Downgrade** | **MODERATE** |
| **BvsC** | **No downgrade** | **No downgrade** | **No downgrade** | **No downgrade** | **Downgrade** | **MODERATE** |
| **BvsE** | **No downgrade** | **No downgrade** | **No downgrade** | **No downgrade** | **Downgrade** | **MODERATE** |
| **BvsF** | **No downgrade** | **No downgrade** | **No downgrade** | **No downgrade** | **Downgrade** | **MODERATE** |
| **BvsG** | **No downgrade** | **No downgrade** | **No downgrade** | **No downgrade** | **Downgrade** | **MODERATE** |
| **BvsH** | **No downgrade** | **No downgrade** | **No downgrade** | **No downgrade** | **Downgrade** | **MODERATE** |
| **CvsE** | **No downgrade** | **Downgrade**  **Because point estimate >1.0**  **but lower limit<0.80** | **Downgrade**  **because sidesplitting**  **p=0.014** | **No downgrade** | **Downgrade** | **VERY LOW** |
| **CvsF** | **No downgrade** | **No downgrade** | **No downgrade** | **No downgrade** | **Downgrade** | **MODERATE** |
| **CvsG** | **No downgrade** | **No downgrade** | **No downgrade** | **No downgrade** | **Downgrade** | **MODERATE** |
| **DvsF** | **No downgrade** | **No downgrade** | **No downgrade** | **No downgrade** | **Downgrade** | **MODERATE** |
| **DvsG** | **No downgrade** | **No downgrade** | **No downgrade** | **No downgrade** | **Downgrade** | **MODERATE** |
| **EvsF** | **No downgrade** | **No downgrade** | **No downgrade** | **No downgrade** | **Downgrade** | **MODERATE** |
| **FvsG** | **No downgrade** | **No downgrade** | **No downgrade** | **No downgrade** | **Downgrade** | **MODERATE** |

**A，acupuncture with moxibustion；B，acupuncture；C，moxibutsion；D，acupuncture with THM；E，moxibustion with THM；F，traditional chinses herbal medicine(THM)；G，western medicine；H，no control group.**

**2 FS-14 total score of CFS patients**

**2.1 Contribution of low or moderate RoB comparisons to each network estimate**

**Based on the assessment of RoB for each comparison and the contribution matrix detailing contribution of each direct comparison to all network estimates, the following bar graphs show the percentage of low or moderate RoB contributions for each network estimate.**

**Each bar corresponds to a NMA relative treatment effect and shows how much information comes from comparisons at low risk of bias [green] or moderate risk of bias [yellow].**

**
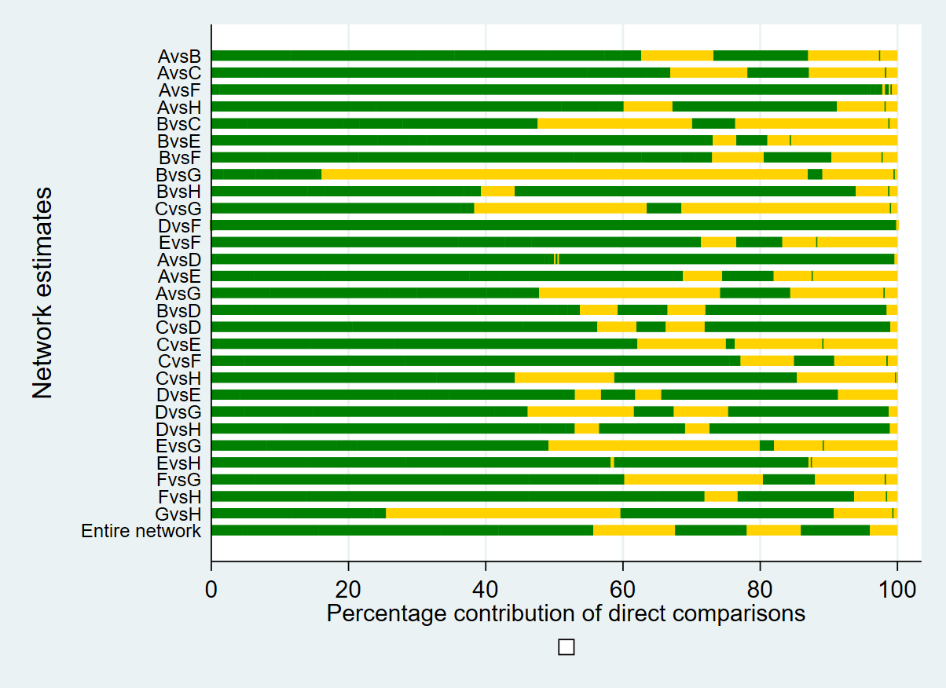
**

|  | P1 | P2 | P3 | P4 | P5 | P6 | P7 | P8 | P9 | P10 | P11 | P12 |
| --- | --- | --- | --- | --- | --- | --- | --- | --- | --- | --- | --- | --- |
| AvsB | 11.5 | 18.6 | 5.4 | 13.8 | 8.1 | 2.5 | 2.9 | 10.5 | 13.8 | 10.5 | 0..0 | 2.5 |
| AvsC | 7.5 | 34.9 | 3.5 | 9.0 | 8.6 | 1.6 | 1.9 | 11.3 | 9.0 | 11.3 | 0.0 | 1.6 |
| AvsF | 0.5 | 0.7 | 94.0 | 0.6 | 0.3 | 0.8 | 0.9 | 0.4 | 0.6 | 0.4 | 0.0 | 0.8 |
| AvsH | 7.8 | 12.6 | 3.6 | 27.0 | 5.5 | 1.7 | 1.9 | 7.1 | 24.0 | 7.1 | 0.0 | 1.7. |
| BvsC | 5.2 | 13.9 | 2.4 | 6.3 | 17.3 | 1.1 | 1.3 | 22.5 | 6.3 | 22.5 | 0.0 | 1.1 |
| BvsE | 3.8 | 6.1 | 14.4 | 4.5 | 2.6 | 40.5 | 1.1 | 3.5 | 4.5 | 3.4 | 0.0 | 15.5 |
| BvsF | 8.2 | 13.3 | 31.3 | 9.9 | 5.8 | 2.1 | 2.4 | 7.5 | 9.9 | 7.5 | 0.0 | 2.1 |
| BvsG | 1.8 | 4.7 | 0.8 | 2.1 | 5.8 | 0.4 | 0.4 | 70.9 | 2.1 | 10.6 | 0.0 | 0.4 |
| BvsH | 5.4 | 8.7 | 2.5 | 16.5 | 3.8 | 1.2 | 1.3 | 4.9 | 49.7 | 4.9 | 0.0 | 1.2 |
| CvsG | 4.2 | 11.2 | 2.0 | 5.1 | 13.9 | 0.9 | 1.0 | 25.1 | 5.1 | 30.6 | 0.0 | 0.9 |
| DvsF | 0.0 | 0.0 | 0.0 | 0.0 | 0.0 | 0.0 | 0.0 | 0.0 | 0.0 | 0.0 | 99.9 | 0.0 |
| EvsF | 5.6 | 9.0 | 21.4 | 6.7 | 3.9 | 23.0 | 1.7 | 5.1 | 6.7 | 5.1 | 0.0 | 11.7 |
| AvsD | 0.2 | 0.4 | 48.0 | 0.3 | 0.2 | 0.4 | 0.5 | 0.2 | 0.3 | 0.2 | 48.9 | 0.4 |
| AvsE | 6.3 | 10.1 | 13.7 | 7.5 | 4.4 | 25.3 | 1.4 | 5.7 | 7.5 | 5.7 | 0.0 | 12.3 |
| AvsG | 8.5 | 17.5 | 4.0 | 10.2 | 3.7 | 1.8 | 2.1 | 26.4 | 10.2 | 13.8 | 0.0 | 1.8 |
| BvsD | 6.2 | 9.8 | 23.1 | 7.3 | 4.2 | 1.6 | 1.8 | 5.5 | 7.3 | 5.5 | 26.4 | 1.6 |
| CvsD | 3.6 | 17.0 | 24.8 | 4.3 | 4.4 | 1.0 | 1.2 | 5.7 | 4.3 | 5.7 | 27.1 | 1.2 |
| CvsE | 1.1 | 13.3 | 10.9 | 1.3 | 9.9 | 25.4 | 0.2 | 12.9 | 1.3 | 12.9 | 0.0 | 10.8 |
| CvsF | 4.9 | 23.3 | 34.1 | 5.9 | 6.0 | 1.4 | 1.6 | 7.8 | 5.9 | 7.8 | 0.0 | 1.4 |
| CvsH | 0.7 | 16.4 | 0.3 | 15.4 | 11.1 | 0.1 | 0.2 | 14.5 | 26.6 | 14.5 | 0.0 | 0.1 |
| DvsE | 4.2 | 6.7 | 15.9 | 5.0 | 2.9 | 17.1 | 1.2 | 3.8 | 5.0 | 3.8 | 25.8 | 8.7 |
| DvsG | 4.9 | 10.0 | 20.7 | 5.8 | 2.1 | 1.3 | 1.5 | 15.5 | 5.8 | 7.9 | 23.4 | 1.3 |
| DvsH | 3.9 | 6.3 | 24.0 | 13.8 | 2.7 | 1.1 | 1.3 | 3.6 | 12.5 | 3.6 | 26.3 | 1.1 |
| EvsG | 1.7 | 6.3 | 10.2 | 2.1 | 0.9 | 27.3 | 0.6 | 30.8 | 2.1 | 7.3 | 0.0 | 10.7 |
| EvsH | 0.6 | 0.6 | 12.4 | 14.0 | 0.4 | 29.8 | 0.0 | 0.5 | 28.3 | 0.5 | 0.0 | 12.4 |
| FvsG | 6.3 | 13.0 | 27.0 | 7.6 | 2.7 | 1.7 | 1.9 | 20.2 | 7.6 | 10.3 | 0.0 | 1.7 |
| FvsH | 5.3 | 8.5 | 32.5 | 18.7 | 3.7 | 1.5 | 1.7 | 4.8 | 17.0 | 4.8 | 0.0 | 1.5 |
| GvsH | 2.5 | 8.1 | 1.2 | 11.8 | 0.6 | 0.6 | 0.6 | 34.2 | 31.1 | 8.7 | 0.0 | 0.6 |

| **Compaison** | **AvsB** | **AvsC** | **AvsF** | **AvsH** | **BvsC** | **BvsE** | **BvsF** | **BvsG** | **BvsH** |
| --- | --- | --- | --- | --- | --- | --- | --- | --- | --- |
| **Moderate percent** | **23.5 %** | **24.2%** | **1.6%** | **15.9%** | **46.1%** | **22.4%** | **17.1%** | **81.9%** | **11%** |
| **CvsG** | **DvsF** | **EvsF** | **AvsD** | **AvsE** | **AvsG** | **BvsD** | **CvsD** | **CvsE** | **CvsF** |
| **56.6%** | **0.0%** | **21.9%** | **0.8%** | **23.7%** | **42%** | **12.6%** | **12.6%** | **36.6%** | **17%** |
| **CvsH** | **DvsE** | **DvsG** | **DvsH** | **EvsG** | **EvsH** | **FvsG** | **FvsH** | **GvsH** | **Entire network** |
| **29.1%** | **16.3%** | **24.7%** | **8.3%** | **48.8%** | **13.4%** | **32.2%** | **11.1%** | **43.5%** | **23.8%** |

**2.2 Table of reasons for downgrading**

**Based on all the above information, we GRADEd each network estimate according to the following criteria.**

1. **Study limitations: We downgraded by one level when the contributions from low RoB comparisons were less than 30% and contributions from moderate RoB comparisons were 70% or greater.**

**(2) Imprecision: We considered a clinically meaningful threshold for OR to be 0.80 or 1.25 and downgraded the estimate if the OR point estimate is 1 or more and the lower limit of its CrI is below 0.80; or if the OR point estimate is less than 1 and the upper limit of its CrI is above 1.25.**

**(3) Inconsistency: We rated two concepts, heterogeneity and incoherence (inconsistency), in this domain.**

**For heterogeneity, we looked at the common tau and found that it is low compared to the expected value as reported in the literature (Turner RM et al (2012) Int J Epidemiol, 41, 818-827) , so we did not downgrade any network estimate for heterogeneity. For inconsistency, we looked at the results of side splitting and we downgraded the comparisons with important inconsistency (p<0.05), where we have not downgraded for imprecision (we did not downgrade the same network estimate for both imprecision and inconsistency).**

**(4) Indirectness: We have assured transitivity in our network by limiting the included studies to chronic fatigue syndrome with the diagnostic criteria of "1994 CDC" .We further ran various subgroup analysis, and assured that they did not violate transitivity of the network.**

**(5) Publication bias: The comparison-adjusted funnel plot did not suggest obvious publication bias. However, we cannot completely rule out the possibility that some studies are still missing. Considering that the field of CFS trials in the past has been prone to publication bias, the review team decided by default to downgrade all the included studies for potential publication bias by one level.**

|  | **Study limitations** | **Imprecision** | **Inconsistency** | **Indirectness** | **Publication bias** | **GRADE** |
| --- | --- | --- | --- | --- | --- | --- |
| **AvsB** | **No downgrade** | **No downgrade** | **No downgrade** | **No downgrade** | **Downgrade** | **MODERATE** |
| **AvsC** | **No downgrade** | **No downgrade** | **No downgrade** | **No downgrade** | **Downgrade** | **MODERATE** |
| **AvsF** | **No downgrade** | **No downgrade** | **No downgrade** | **No downgrade** | **Downgrade** | **MODERATE** |
| **AvsH** | **No downgrade** | **No downgrade** | **No downgrade** | **No downgrade** | **Downgrade** | **MODERATE** |
| **BvsC** | **No downgrade** | **Downgrade**  **Because point estimate >1.0**  **but lower limit<0.80** | **No downgrade** | **No downgrade** | **Downgrade** | **LOW** |
| **BvsE** | **No downgrade** | **Downgrade**  **Because point estimate >1.0**  **but lower limit<0.80** | **No downgrade** | **No downgrade** | **Downgrade** | **LOW** |
| **BvsF** | **No downgrade** | **No downgrade** | **No downgrade** | **No downgrade** | **Downgrade** | **MODERATE** |
| **BvsG** | **Downgrade**  **because >70%**  **contribution**  **from moderate**  **RoB**  **comparisons** | **No downgrade** | **No downgrade** | **No downgrade** | **Downgrade** | **LOW** |
| **BvsH** | **No downgrade** | **No downgrade** | **No downgrade** | **No downgrade** | **Downgrade** | **MODERATE** |
| **CvsG** | **No downgrade** | **No downgrade** | **No downgrade** | **No downgrade** | **Downgrade** | **MODERATE** |
| **DvsF** | **No downgrade** | **No downgrade** | **No downgrade** | **No downgrade** | **Downgrade** | **MODERATE** |
| **EvsF** | **No downgrade** | **No downgrade** | **No downgrade** | **No downgrade** | **Downgrade** | **MODERATE** |

**A，acupuncture with moxibustion；B，acupuncture；C，moxibutsion；D，acupuncture with THM；E，moxibustion with THM；F，traditional chinses herbal medicine(THM)；G，western medicine；H，no control group.**

**3 FS-14 physical score of CFS patients**

**3.1 Contribution of low or moderate RoB comparisons to each network estimate**

**Based on the assessment of RoB for each comparison and the contribution matrix detailing contribution of each direct comparison to all network estimates, the following bar graphs show the percentage of low or moderate RoB contributions for each network estimate.**

**Each bar corresponds to a NMA relative treatment effect and shows how much information comes from comparisons at low risk of bias [green] or moderate risk of bias [yellow].**

**
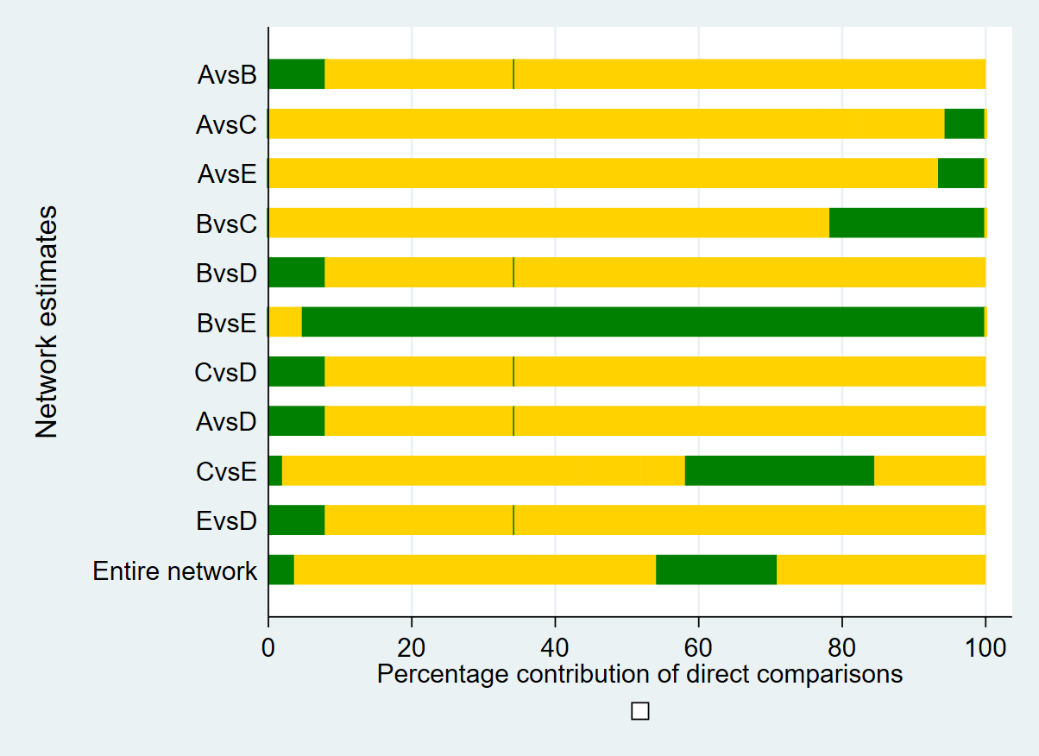
**

|  | P1 | P2 | P3 | P4 | P5 | P6 | P7 |
| --- | --- | --- | --- | --- | --- | --- | --- |
| AvsB | 8.1 | 0.0 | 0.0 | 0.0 | 26.2 | 0.0 | 65.7 |
| AvsC | 0.0 | 77.1 | 5.7 | 11.4 | 0.0 | 5.7 | 0.0 |
| AvsE | 0.0 | 13.3 | 66.9 | 13.3 | 0.0 | 6.6 | 0.0 |
| BvsC | 0.0 | 43.5 | 21.8 | 12.9 | 0.0 | 21.8 | 0.0 |
| BvsD | 8.1 | 0.0 | 0.0 | 0.0 | 26.2 | 0.0 | 65.7 |
| BvsE | 0.0 | 1.9 | 0.9 | 1.9 | 0.0 | 95.3 | 0.0 |
| CvsD | 8.1 | 0.0 | 0.0 | 0.0 | 26.2 | 0.0 | 65.7 |
| AvsD | 8.1 | 0.0 | 0.0 | 0.0 | 26.2 | 0.0 | 65.7 |
| CvsE | 1.9 | 41.6 | 2.8 | 5.6 | 6.2 | 26.4 | 15.5 |
| EvsD | 8.1 | 0.0 | 0.0 | 0.0 | 26.2 | 0.0 | 65.7 |

| **Compaison** | **AvsB** | **AvsC** | | **AvsE** | **BvsC** | **BvsD** | **BvsE** | **CvsD** | **AvsD** | **CvsE** |
| --- | --- | --- | --- | --- | --- | --- | --- | --- | --- | --- |
| **Moderate percent** | **91.9%** | **94.3%** | | **93.4%** | **78.2%** | **91.9%** | **4.7%** | **91.9%** | **91.9%** | **71.7%** |
| **EvsD** | **Entire network** | |  |  |  |  |  |  |  |  |
| **91.9%** | **79.6%** | |  |  |  |  |  |  |  |  |

**3.2 Table of reasons for downgrading**

**Based on all the above information, we GRADEd each network estimate according to the following criteria.**

1. **Study limitations: We downgraded by one level when the contributions from low RoB comparisons were less than 30% and contributions from moderate RoB comparisons were 70% or greater.**

**(2) Imprecision: We considered a clinically meaningful threshold for OR to be 0.80 or 1.25 and downgraded the estimate if the OR point estimate is 1 or more and the lower limit of its CrI is below 0.80; or if the OR point estimate is less than 1 and the upper limit of its CrI is above 1.25.**

**(3) Inconsistency: We rated two concepts, heterogeneity and incoherence (inconsistency), in this domain.**

**For heterogeneity, we looked at the common tau and found that it is low compared to the expected value as reported in the literature (Turner RM et al (2012) Int J Epidemiol, 41, 818-827) , so we did not downgrade any network estimate for heterogeneity. For inconsistency, we looked at the results of side splitting and we downgraded the comparisons with important inconsistency (p<0.05), where we have not downgraded for imprecision (we did not downgrade the same network estimate for both imprecision and inconsistency).**

**(4) Indirectness: We have assured transitivity in our network by limiting the included studies to chronic fatigue syndrome with the diagnostic criteria of "1994 CDC" .We further ran various subgroup analysis, and assured that they did not violate transitivity of the network.**

**(5) Publication bias: The comparison-adjusted funnel plot did not suggest obvious publication bias. However, we cannot completely rule out the possibility that some studies are still missing. Considering that the field of CFS trials in the past has been prone to publication bias, the review team decided by default to downgrade all the included studies for potential publication bias by one level.**

|  | **Study limitations** | **Imprecision** | **Inconsistency** | **Indirectness** | **Publication bias** | **GRADE** |
| --- | --- | --- | --- | --- | --- | --- |
| **AvsB** | **Downgrade**  **because >70%**  **contribution**  **from moderate**  **RoB**  **comparisons** | **No downgrade** | **No downgrade** | **No downgrade** | **Downgrade** | **LOW** |
| **AvsC** | **Downgrade**  **because >70%**  **contribution**  **from moderate**  **RoB**  **comparisons** | **No downgrade** | **No downgrade** | **No downgrade** | **Downgrade** | **LOW** |
| **AvsE** | **Downgrade**  **because >70%**  **contribution**  **from moderate**  **RoB**  **comparisons** | **No downgrade** | **Downgrade**  **because sidesplitting**  **p=0.004** | **No downgrade** | **Downgrade** | **VERY LOW** |
| **BvsC** | **Downgrade**  **because >70%**  **contribution**  **from moderate**  **RoB**  **comparisons** | **Downgrade**  **Because point estimate >1.0**  **but lower limit<0.80** | **No downgrade** | **No downgrade** | **Downgrade** | **VERY LOW** |
| **BvsD** | **Downgrade**  **because >70%**  **contribution**  **from moderate**  **RoB**  **comparisons** | **No downgrade** | **No downgrade** | **No downgrade** | **Downgrade** | **LOW** |
| **BvsE** | **No downgrade** | **No downgrade** | **Downgrade**  **because sidesplitting**  **p=0.001** | **No downgrade** | **Downgrade** | **LOW** |
| **CvsD** | **Downgrade**  **because >70%**  **contribution**  **from moderate**  **RoB**  **comparisons** | **No downgrade** | **No downgrade** | **No downgrade** | **Downgrade** | **LOW** |

**A，acupuncture with moxibustion；B，acupuncture；C，moxibutsion； D，western medicine；E，no control group.**

**4 FS-14 mental score of CFS patients**

**4.1 Contribution of low or moderate RoB comparisons to each network estimate**

**Based on the assessment of RoB for each comparison and the contribution matrix detailing contribution of each direct comparison to all network estimates, the following bar graphs show the percentage of low or moderate RoB contributions for each network estimate.**

**Each bar corresponds to a NMA relative treatment effect and shows how much information comes from comparisons at low risk of bias [green] or moderate risk of bias [yellow].**

**
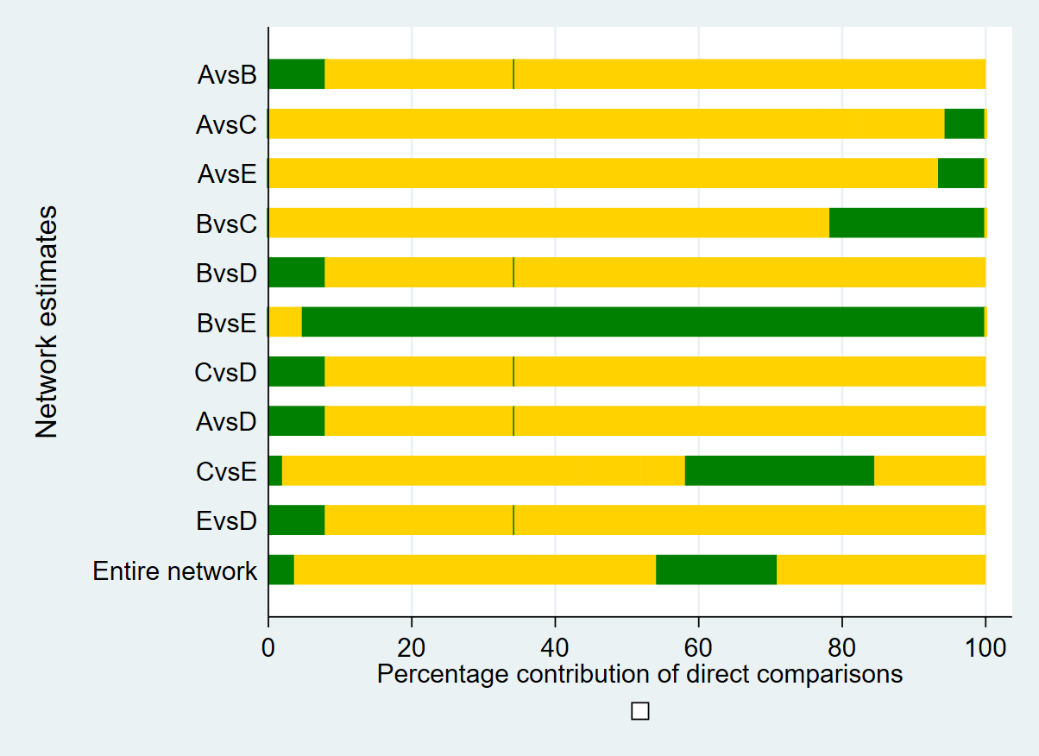
**

|  | P1 | P2 | P3 | P4 | P5 | P6 | P7 |
| --- | --- | --- | --- | --- | --- | --- | --- |
| AvsB | 8.1 | 0.0 | 0.0 | 0.0 | 26.2 | 0.0 | 65.7 |
| AvsC | 0.0 | 77.1 | 5.7 | 11.4 | 0.0 | 5.7 | 0.0 |
| AvsE | 0.0 | 13.3 | 66.9 | 13.3 | 0.0 | 6.6 | 0.0 |
| BvsC | 0.0 | 43.5 | 21.8 | 12.9 | 0.0 | 21.8 | 0.0 |
| BvsD | 8.1 | 0.0 | 0.0 | 0.0 | 26.2 | 0.0 | 65.7 |
| BvsE | 0.0 | 1.9 | 0.9 | 1.9 | 0.0 | 95.3 | 0.0 |
| CvsD | 8.1 | 0.0 | 0.0 | 0.0 | 26.2 | 0.0 | 65.7 |
| AvsD | 8.1 | 0.0 | 0.0 | 0.0 | 26.2 | 0.0 | 65.7 |
| CvsE | 1.9 | 41.6 | 2.8 | 5.6 | 6.2 | 26.4 | 15.5 |
| EvsD | 8.1 | 0.0 | 0.0 | 0.0 | 26.2 | 0.0 | 65.7 |

| **Compaison** | **AvsB** | **AvsC** | | **AvsE** | **BvsC** | **BvsD** | **BvsE** | **CvsD** | **AvsD** | **CvsE** |
| --- | --- | --- | --- | --- | --- | --- | --- | --- | --- | --- |
| **Moderate percent** | **91.9%** | **94.3%** | | **93.4%** | **78.2%** | **91.9%** | **4.7%** | **91.9%** | **91.9%** | **71.7%** |
| **EvsD** | **Entire network** | |  |  |  |  |  |  |  |  |
| **91.9%** | **79.6%** | |  |  |  |  |  |  |  |  |

**4.2 Table of reasons for downgrading**

**Based on all the above information, we GRADEd each network estimate according to the following criteria.**

1. **Study limitations: We downgraded by one level when the contributions from low RoB comparisons were less than 30% and contributions from moderate RoB comparisons were 70% or greater.**

**(2) Imprecision: We considered a clinically meaningful threshold for OR to be 0.80 or 1.25 and downgraded the estimate if the OR point estimate is 1 or more and the lower limit of its CrI is below 0.80; or if the OR point estimate is less than 1 and the upper limit of its CrI is above 1.25.**

**(3) Inconsistency: We rated two concepts, heterogeneity and incoherence (inconsistency), in this domain.**

**For heterogeneity, we looked at the common tau and found that it is low compared to the expected value as reported in the literature (Turner RM et al (2012) Int J Epidemiol, 41, 818-827) , so we did not downgrade any network estimate for heterogeneity. For inconsistency, we looked at the results of side splitting and we downgraded the comparisons with important inconsistency (p<0.05), where we have not downgraded for imprecision (we did not downgrade the same network estimate for both imprecision and inconsistency).**

**(4) Indirectness: We have assured transitivity in our network by limiting the included studies to chronic fatigue syndrome with the diagnostic criteria of "1994 CDC" .We further ran various subgroup analysis, and assured that they did not violate transitivity of the network.**

**(5) Publication bias: The comparison-adjusted funnel plot did not suggest obvious publication bias. However, we cannot completely rule out the possibility that some studies are still missing. Considering that the field of CFS trials in the past has been prone to publication bias, the review team decided by default to downgrade all the included studies for potential publication bias by one level.**

|  | **Study limitations** | **Imprecision** | **Inconsistency** | **Indirectness** | **Publication bias** | **GRADE** |
| --- | --- | --- | --- | --- | --- | --- |
| **AvsB** | **Downgrade**  **because >70%**  **contribution**  **from moderate**  **RoB**  **comparisons** | **No downgrade** | **No downgrade** | **No downgrade** | **Downgrade** | **LOW** |
| **AvsC** | **Downgrade**  **because >70%**  **contribution**  **from moderate**  **RoB**  **comparisons** | **Downgrade**  **because point**  **estimate <1.0**  **but upper limit**  **>1.25** | **No downgrade** | **No downgrade** | **Downgrade** | **VERY LOW** |
| **AvsE** | **Downgrade**  **because >70%**  **contribution**  **from moderate**  **RoB**  **comparisons** | **No downgrade** | **Downgrade**  **because sidesplitting**  **p=0.004** | **No downgrade** | **Downgrade** | **VERY LOW** |
| **BvsC** | **Downgrade**  **because >70%**  **contribution**  **from moderate**  **RoB**  **comparisons** | **Downgrade**  **Because point estimate >1.0**  **but lower limit<0.80** | **No downgrade** | **No downgrade** | **Downgrade** | **VERY LOW** |
| **BvsD** | **Downgrade**  **because >70%**  **contribution**  **from moderate**  **RoB**  **comparisons** | **No downgrade** | **No downgrade** | **No downgrade** | **Downgrade** | **LOW** |
| **BvsE** | **No downgrade** | **No downgrade** | **Downgrade**  **because sidesplitting**  **p=0.004** | **No downgrade** | **Downgrade** | **LOW** |
| **CvsD** | **Downgrade**  **because >70%**  **contribution**  **from moderate**  **RoB**  **comparisons** | **No downgrade** | **No downgrade** | **No downgrade** | **Downgrade** | **LOW** |

**A，acupuncture with moxibustion；B，acupuncture；C，moxibutsion； D，western medicine；E，no control group.**
