# Supplementary material for: Acupuncture and moxibustion for chronic fatigue syndrome: A systematic review and network meta-analysis
Source: Medicine (Baltimore). 2022 Aug 5;101(31):e29310. doi: 10.1097/MD.0000000000029310 (PMC9351926; doi:10.1097/MD.0000000000029310)
Supplement: Supplementary file 5 [file medi-101-e29310-s005.docx]

**see Figure, Supplemental Content 5, which illustrates the SUCRA diagram.**

A Overall response rate

a.Ranking probability diagram


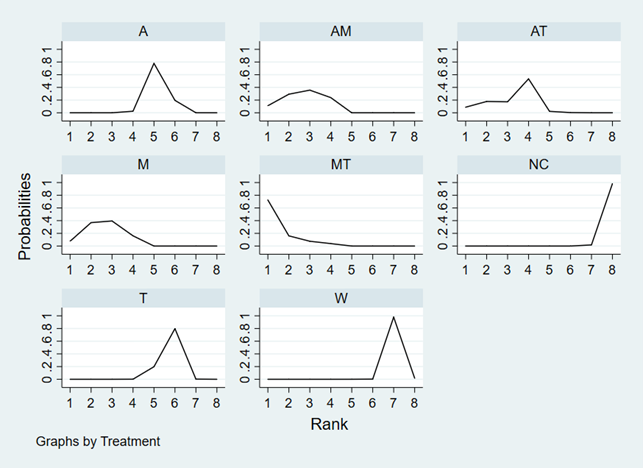


b.Cumulative probability ranking diagram


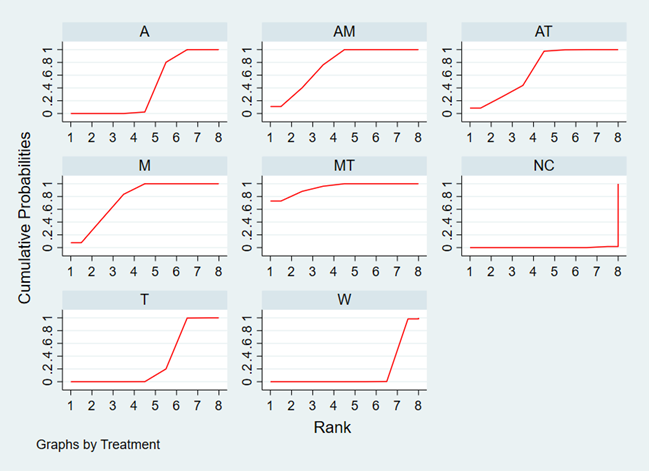


AM-Acupuncture with moxibustion; A-Acupuncture; M-Moxibustion; AT-Acupuncture with THM; MT-Moxibustion with THM; T-Traditional Chinses herbal medicine; W-Western medicine; NC-No control.

B FS-14 total score

a.Ranking probability diagram


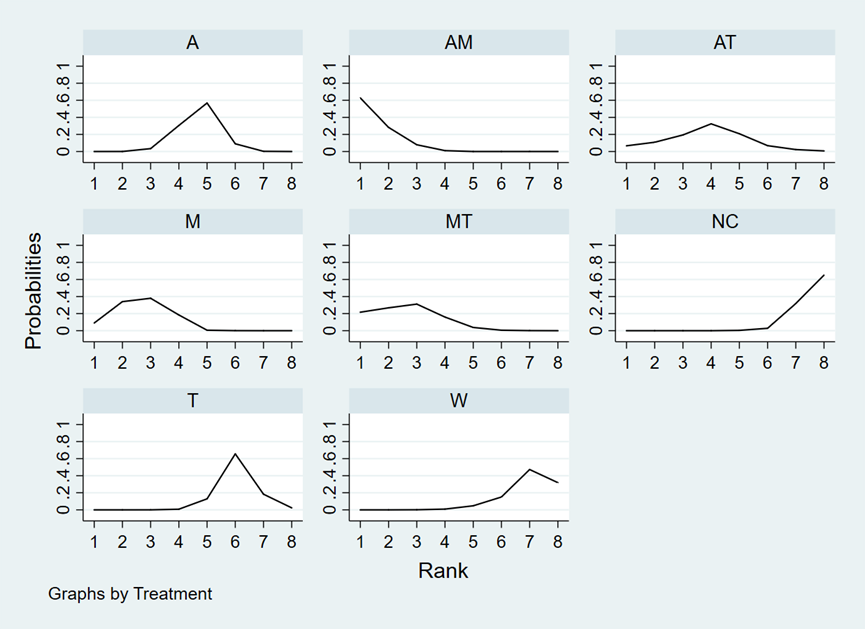


b.Cumulative probability ranking diagram


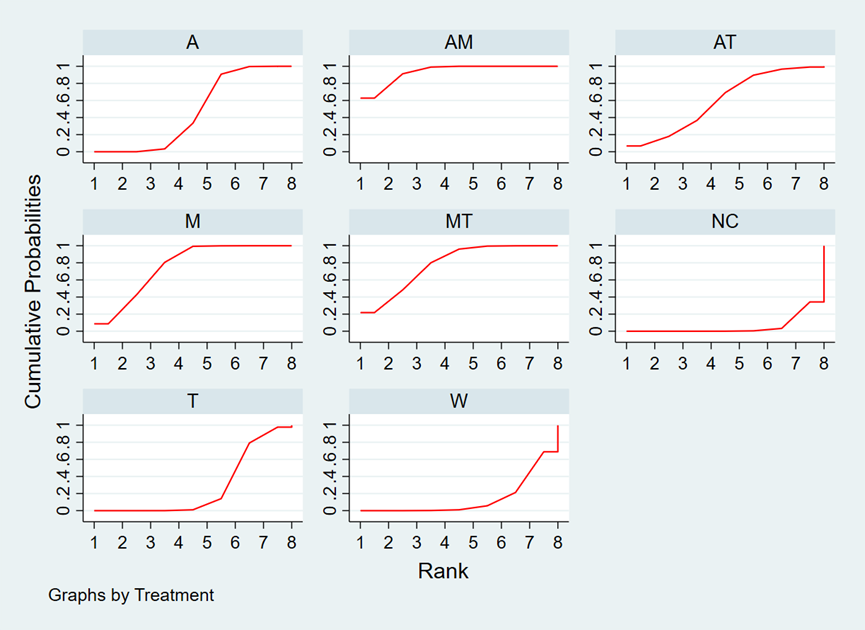


AM-Acupuncture with moxibustion; A-Acupuncture; M-Moxibustion; AT-Acupuncture with THM; MT-Moxibustion with THM; T-Traditional Chinses herbal medicine; W-Western medicine; NC-No control.

C FS-14 physical score

a.Ranking probability diagram


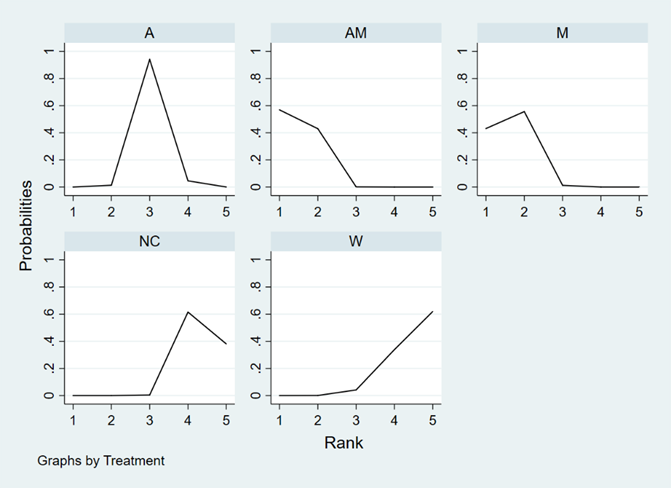


b.Cumulative probability ranking diagram


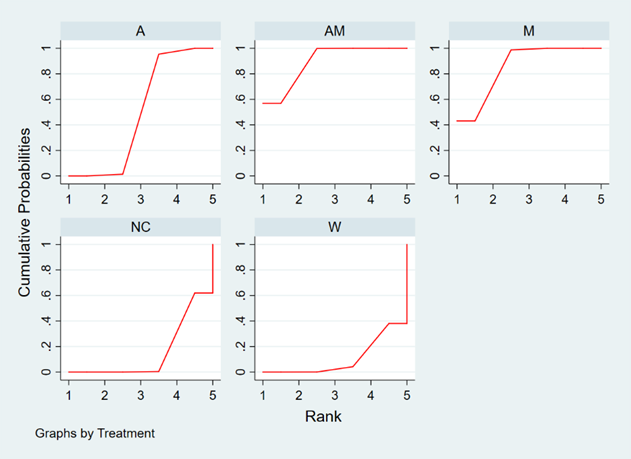


AM-Acupuncture with moxibustion; A-Acupuncture; M-Moxibustion; W-Western medicine; NC-No control.

D FS-14 mental score

a.Ranking probability diagram


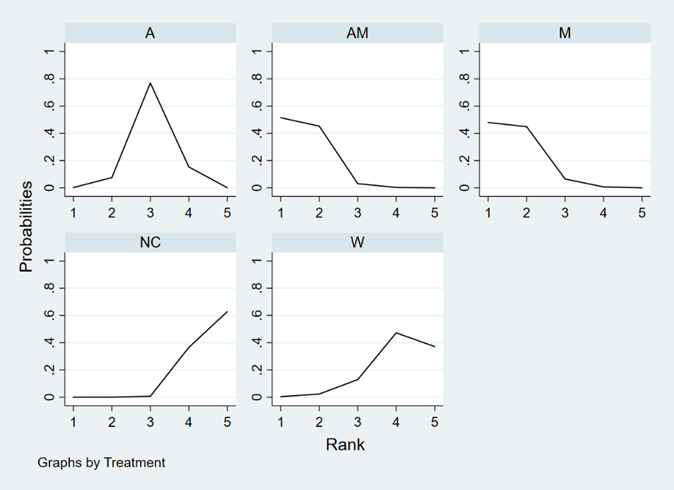


b.Cumulative probability ranking diagram


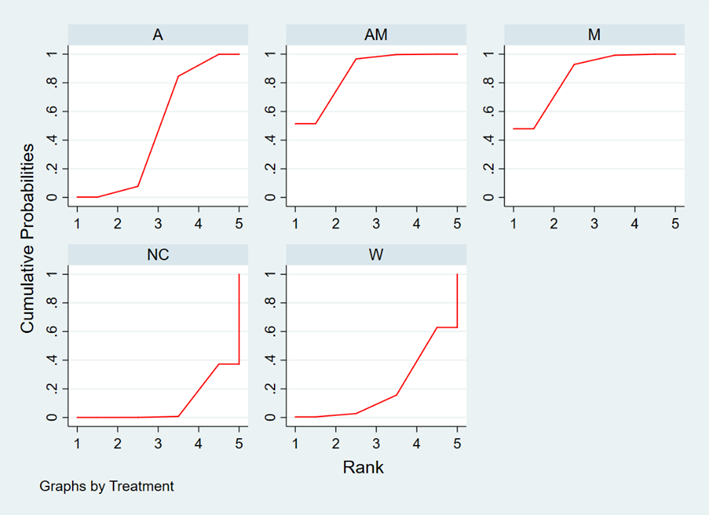


AM-Acupuncture with moxibustion; A-Acupuncture; M-Moxibustion; W-Western medicine; NC-No control.
