# Supplementary material for: Acupuncture and moxibustion for chronic fatigue syndrome: A systematic review and network meta-analysis
Source: Medicine (Baltimore). 2022 Aug 5;101(31):e29310. doi: 10.1097/MD.0000000000029310 (PMC9351926; doi:10.1097/MD.0000000000029310)
Supplement: Supplementary file 6 [file medi-101-e29310-s006.pdf]

Efficacy of Traditional Chinese Medicine Therapy in the Treatment of Chronic Fatigue Syndrome: A Network Meta-Analysis of Randomized Clinical Trials

SUPPLEMENTARY APPENDIX

目录

1 PRISMA diagram 1
2 Characteristics of Included studies 3
3 Risk of bias table of included studies 22
4 Analyses of the all trials network 26
5 GRADE for the primary outcomes 80

1 PRISMA diagram

1.1 PRISMA diagram showing selection of articles for pairwise

and network meta-analysis

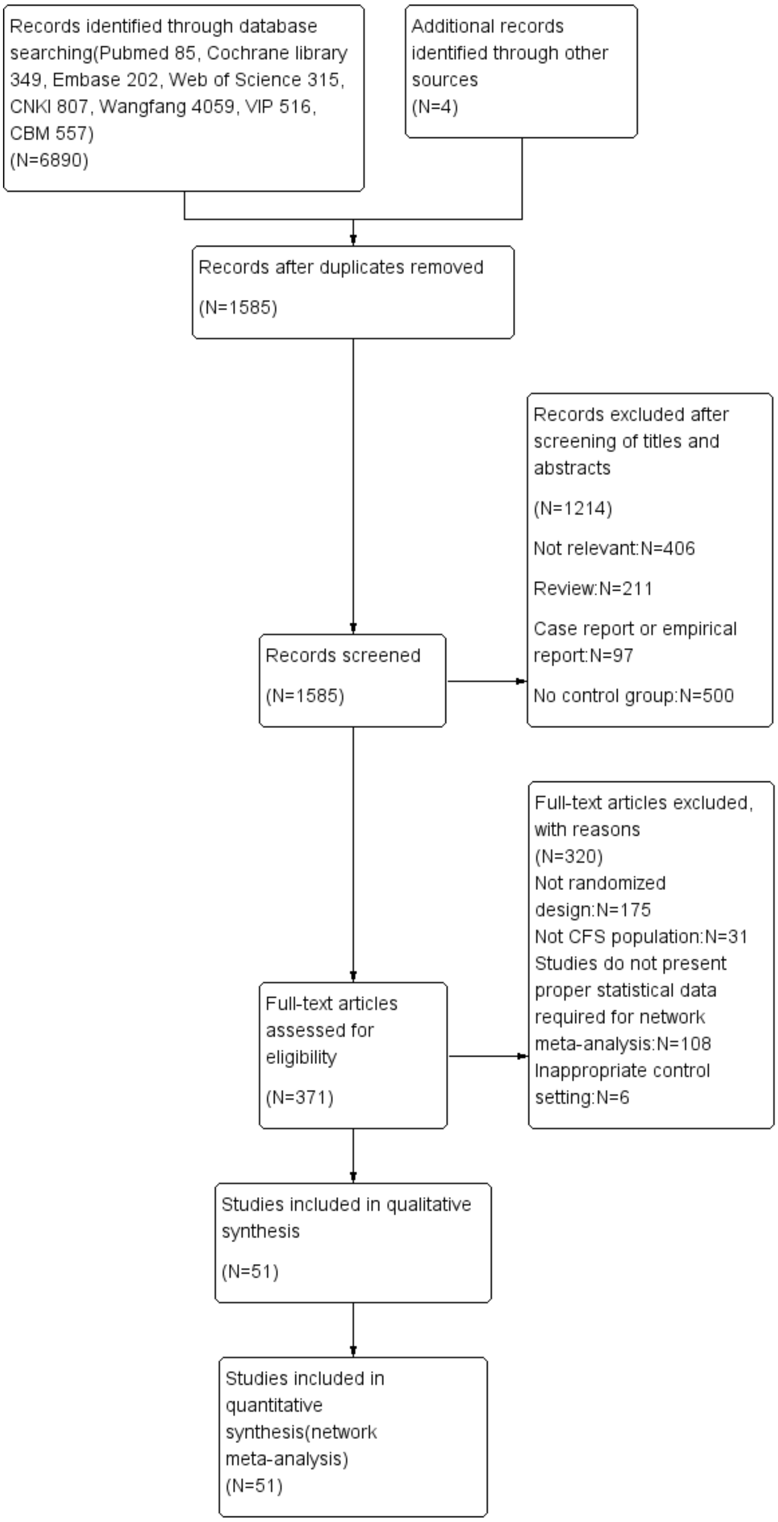

2 Characteristics of Included studies

2.1 Characteristics of Included studies (RCTs)

First author(year)	Diagnosis	Participants			Interventions		Outcome measures
		Number(male/female)	Age(mean ±SD or range)	Disease duration(mean ±SD or range	Experimental group(E)	Control group(C)	
Xiao (2014)	1994CDC	80 (39/41)	E: 40.2±8.2 years C: 40.6±8.5 years	E: 0.5~2years C: 0.6~2years	Mox: semi-fixed points; mainpoints(bilateral BL13、BL15、BL18、BL20、BL23) and additional points(according to TCM syndrome differentiation); 15min/session, 30 sessions for 5 weeks	WM: *Vitamin B1*、*Vitamin B6*、*Oryzanol*、*Paroxetine*; 5 weeks	FS-14；SF-36；adverse reaction；overall response rate
Li (2018)	1994CDC	60 (24/36)	E: 38.4±3.1 years C: 38.1±3.2 years	E: 6.10 ± 18.30 months C: 6.09 ± 18.17 months	Mox: fixed points(bilateral ST36)；15min/session, 20 sessions for 4 weeks	THM: *Liuwei Dihuang pill;* one dose daily for 4 weeks	FS-14;overall response rate;CFS clinical symptom score
Xing (2019)	1994 CDC	80 (28/52)	E: 32.23 ± 10.35 years C: 36.98	Not reported	MT: *Shenqi Zhencao decoction* combined with moxibustion (CV4);moxib	Mox: fixed point(CV4)；30min/session,20 sessions for 4 weeks	FASA;overall response rate

			± 11.38 years		ustion 30min/session, THM one dose daily; 20 sessions for 4 weeks		
Qi (2017)	1994 CDC	60 (36/24)	E: 38 ± 9 years C: 37 ± 11 years	E: 27.7 ± 5.8 months C: 26.5 ± 4.5 months	AT: *Chaihu Longgu Muli Decoction* combined with acupuncture (semi-fixed points; main points (bilateral BL13、BL15、BL18、BL20、BL23) and additional points(according to TCM syndrome differentiation); de qi required; acupuncture 30min/session, THM one dose daily; 36 sessions for 5 weeks	THM: *Chaihu Longgu Muli Decoction;* one dose daily for 5 weeks	FS-14、SAS
Huang (2017)	1994 CDC	60 (29/31)	E: 39.70 ±9.86 years C: 41.73 ±9.33 years	E: 14.13 ± 3.12 months C: 13.97 ± 3.23 months	Acu: fixed points(GV20、GV29; bilateral HT7、KI3、LR3、SP6、ST36); de qi required; 40min/session, 16 sessions for 2 months	WM: *Vitamin B1*、*Oryzanol, once daily for 2 months*	FAI;FS-14;SAS;SDS; overall response rate

Hu (2013)	1994 CDC	60 (25/35)	E: 37.36 ±8.55 years C: 37.20 ±6.93 years	E: 38.27 ± 8.73 months C: 39.30 ± 7.55 months	AT: acupuncture combined with THM; fixed points(GV20、CV4、CV6;bilateral BL15、BL17、BL18、BL20、PC6、LI4、ST36、LR3); de qi required; 30min/session, 12 sessions for 4 weeks; *Liuwei Dihuang pill*; three times daily for 4 weeks	THM: *Liuwei Dihuang pill*; three times daily for 4 weeks	FAI;WHOQOL-BREF;overall response rate; CFS clinical symptom score
Xiong (2005)	1994 CDC	87 (25/62)	E: 18~50years C: 18~50years	E: 6~12 months C: 6~12months	Acu: fixed points(bilateral BL13、BL15、BL18、BL20、BL23); de qi required; 30min/session, 20 session for 4 weeks	THM: *Liuwei Dihuang pill*; twice daily for 4 weeks	Overall response rate;FS-14;SCL-90; adverse reaction
Xu (2014)	1994 CDC	95 (51/44)	E: AT 20~50 years C：THM 20~50 years WM	E: AT 0.5~3 years C: THM 0.5~3 years WM 0.5~3 years	AT: fixed points(GV14、BL43、GV4、CV6、ST36); de qi required; 15min/session, 20 sessions for 4 weeks; *Qingshu Yiqi*	THM: *Qingshu Yiqi decoction*; once daily for 4 weeks WM: *oryzanol*、*diazepam*; symptomatic	Overall response rate

			20~50 years		*decoction*; once daily for 4 weeks	treatment	
Hou (2017)	1994 CDC	54 (30/24)	E: 43.04 ±9.31 years C: 45.62 ±9.92 years	E: 52.54 ± 15.23 days C: 48.35 ± 16.93 days	Mox: fixed points(GV14~GV2); 15min/session,6 sessions for 3 months	Acu: semi-fixed points(GV29、GV20; bilateral KI3、HT7、SP6、LR3) and additional points(according to TCM syndrome differentiation); 30min/session, 48 sessions for 3 months	FS-14; CFS clinical symptom score;overall response rate; adverse reaction
Xu (2019)	1994 CDC	94 (56/38)	E: 41.5±5.3 years C: 42.5±3.6 years	Not reported	Mox: fixed points(GV14~GV2); 15min/session,6 sessions for 3 months	Acu: semi-fixed points; main points(GV29; bilateral KI3、HT7、SP6、LR3) and additional points(according to TCM syndrome differentiation); 30min/session, 48 sessions for 3	Overall response rate;FS-14; CFS clinical symptom score

						months	
Liu (2018)	1994 CDC	88 (19/69)	E: AM 37.39 ±8.47 years C: Mox 37.10 ±7.36 years Acu 36.60 ±8.07 years	E: AM 18.29 ± 5.81 months C: Mox 15.93 ± 5.71 months Acu 16.73 ± 5.74 months	AM：Mox fixed points (GV14~GV2); 40min/session,8 sessions for 4 weeks；Acu fixed points(GV20、CV4、BL20、BL18、BL23、ST36、SP6)；de qi required 30min/session, 8 sessions for 4 weeks	Mox: fixed points (GV14~GV2); 40min/session,8 sessions for 4 weeks Acu: fixed points(GV20、CV4、BL20、BL18、BL23、ST36、SP6）;de qi required; 30min/session, 8 sessions for 4 weeks	Overall response rate;FS-14;FAI
Shi (2015)	1994 CDC	59 (17/42)	E: 39.00 ± 12.54 years C: 41.62 ± 11.70 years	E：13.00 ± 4.94 months C：13.42 ± 4.97 months	Mox：fixed points（GV14~GV2；BL11~BL26)；30min/session，12 sessions for 6 weeks	Acu：semi-fixed points; main points(GV20、PC6、CV4、ST36、KI3、BL20、BL23、GV4) and additional points(according to TCM syndrome differentiation)；de qi required; 30min/session, 18 sessions for 6 weeks	FS-14;SAS;overall response rate; adverse reaction

Luo Dai Hong (2019)	1994 CDC	60 (31/29)	E: 43 ± 4 years C: 42 ± 3 years	E: 11.9 ± 4.4 months C: 11.1 ± 3.7 months	Mox: fixed points（GV14~GV2；BL11~BL26）；30min/session, 30 sessions for 2 months	Acu: fixed points(GV20、CV6、CV4；bilateral BL20、BL23、ST36、PC6); de qi required;30min/session, 30 sessions for 2 months	FS-14;overall response rate; adverse reaction
Zhou (2018)	1994 CDC	96 (30/66)	E: 35.2±8.5 years C: 34.7±8.3 years	E: 1.0±0.4 years C: 1.1±0.4 years	AM: Acu fixed points(CV12、CV10、CV6、CV4; bilateral ST24、ST26、SP15、KI13); de qi required; 30min/session,20 sessions for 6 weeks; Mox fixed points(CV6、CV4); 30min/session,20 sessions for 6 weeks	Acu: fixed points(CV12、CV10、CV6、CV4; bilateral ST24、ST26、SP15、KI13); de qi required; 30min/session,20 sessions for 6 weeks	FS-14;overall response rate
Zhu (2012)	1994 CDC	60 (25/35)	E: 26.98 ±9.54 years C: 28.58 ± 12.67 years	E: 18.72 ± 14.00 months C: 20.08 ± 16.11 months	Acu: semi-fixed points; main points(CV12、CV10、CV6、CV4、ST25、ST26、ST24、CV9) and additional points(according to TCM	THM: Chinses herb decoctions according to syndrome differentiation; 10 days	FSS;HAMA;VAS;HAMD;overall response rate; adverse reaction

					syndrome differentiation); 30min/session, 10 sessions for 3 weeks		
Tian (2015)	1994 CDC	72 (40/32)	E: 42 ± 9 years C: 42 ± 10 years	Not reported	Mox: fixed points(BL43、CV6 、ST36);30min/session,30 sessions for 1 month	Acu: fixed points(BL43、CV6、ST36); de qi required;30min/session,30 sessions for 1 month	FAI;overall response rate; adverse reaction
Jiang (2015)	1994 CDC	60 (24/36)	E: 37 ± 9 years C: 39 ± 11 years	Not reported	MT: Mox fixed points(CV12、CV4、CV6、ST36、PC6)；15min/session, 18 sessions for 3 weeks THM *Bazhen decoction*; one dose daily for 3 weeks	Acu: fixed points(CV20、CV29、TF4、PC6、LR3、ST36、SP6)；de qi required; 30min/ session, 18 sessions for 3 weeks	FS-14;overall response rate
Guo (2016)	1994 CDC	60 (29/31)	E: 45.48 ±8.32 years C: 46.31 ±7.95 years	E: 58.32 ± 10.65 months C: 60.58 ± 11.57 months	MT: Mox fixed points(CV6; bilateral ST36、BL20)；15min/session, 20 sessions for 1 month THM *Bazhen decoction; one dose*	THM: *Bazhen decoction; one dose daily for 1 month*	Overall response rate;FS-14; adverse reaction

					daily for 1 month		
Zhao (2014)	1994 CDC	60 (19/41)	E: 40.80 ±6.60 years C: 41.07 ±5.78 years	E: 11.57 ± 3.58 months C: 11.13 ± 2.98 months	Mox: fixed points(CV4; bilateral BL20、BL23、ST36); 15min/session, 20 sessions for 4 weeks	Acu: fixed points(GV20、CV4、BL18、BL20、BL23、ST36、CV17); de qi required; 30min/session, 20 sessions for 4 weeks	FS-14;SDS;SAS; overall response rate
Luo Wen (2019)	1994 CDC	90 (32/58)	E: 37.58 ±6.48 years C: 38.25 ±6.26 years	E: 10.42 ± 3.22 months C: 11.06 ± 3.18 months	AM: Acu fixed points(CV13、CV12、CV10、CV4、CV6; bilateral ST25、ST36、SP6); de qi required; 30min/session, 28 sessions for 4 weeks; Mox fixed point(CV8); 6min/session, 28 sessions for 4 weeks	Acu: fixed points(CV13、CV12、CV10、CV4、CV6; bilateral ST25、ST36、SP6); de qi required; 30min/session, 28 sessions for 4 weeks;	FSS;SF-36;overall response rate
Zheng (2014)	1994 CDC	86 (44/42)	E: 43.5± 13.2 years C: 42.6± 12.9 years	E: 2.85 ± 0.89 years C: 2.82 ± 0.92 years	Mox: fixed points(bilateral ST36、GB39); de qi required; 5min/session, 21 sessions for 4 weeks	Acu: semi-fixed points; main points(GV20、GV29、TF4、KI3、LR3、ST36、SP6) and	Overall response rate; adverse reaction

						additional points(according to TCM syndrome differentiation); de qi required; 30min/sessions, 21 sessions for 4 weeks	
Wang (2013)	1994 CDC	80 (25/55)	E: 39 ± 6 years C: 38 ± 8 years	E: 23.6 ± 2.9 months C: 25.1 ± 3.3 months	Mox: fixed points(GV20 、 EX-HN1); 1min/session , 20 sessions for 3 weeks	WM: Fluoxetine hydrochloride; 20mg/d for 3 weeks	Overall response rate; adverse reaction
Sui (2015)	1994 CDC	60 (27/33)	E: 44.2±4.2 years C: 45.3±5.1 years	E: 9.7± 2.5 months C: 10.1 ± 3.1 months	Mox: fixed points(CV4、CV6、GV4；bilateral BL20、BL15、PC6 、 ST36); 30min/session, 56 sessions for 2 weeks	THM: *Guipi pill;* 10 pills/bid for 2 months	Overall response rate
Hao (2013)	1994 CDC	59 (24/35)	E: 35.07 ±7.77 years C: 37.07 ±8.00 years	E: 26.53 ± 14.29 months C: 23.79 ± 14.26 months	AM: Acu semi-fixed points;main points and additional points(according to TCM syndrome differentiation); de qi required; 20min/session, 24 sessions for 8 weeks	Acu: semi-fixed points;main points(GV20 、 GV29; bilateral TF4、KI3、LR3 、 SP6) and additional points(according to TCM syndrome differentiat	FS-14;overall response rate; adverse reaction

					Mox fixed point(CV8); 20min/session, 24 sessions for 8 weeks	ion); de qi required; 30min/session, 24 sessions for 8 weeks	
Li Wei Wei (2016)	1994 CDC	60 (34/26)	E: 32~68 years C: 30~60 years	Not reported	Acu: semi-fixed points; main points and additional points(according to TCM syndrome differentiation); de qi required; 30min/session, 30 sessions for 1 month;	THM: *Buzhong Yiqi decoction* combined with *Liuwei Dihuang pill*; one dose daliy for 1 month	FS-14;overall response rate;SDS
Lin (2010)	1994 CDC	60 (19/41)	E: 38.27 ±7.49 years C: 39.77 ±5.58 years	Not reported	AM: fixed points(bilateral BL13 、BL15、BL18、BL20 、BL23);de qi required; 30min/session, 15 sessions for 3 weeks	THM: Guipi pill; 6g/tid for 3 weeks	FS-14;FSS;overall response rate; adverse reaction
Song (2016)	1994 CDC	57 (20/37)	E: 35.06 ±1.35 years C: 35.50 ±1.40 years	E: 17.56 ± 0.48 months C: 17.30 ± 0.44 months	AM: fixed points(BL23、BL18、BL20、ST36、SP6、GV20); de qi required; 30min/session, 30 sessions for 1 month	THM: *Yougui pill*; 9g/tid for 1 month	FS-14;overall response rate; adverse reaction
Liu You	1994	70 (23/47)	E: 40.6±	E: 14.5 ± 6.7	Acu: fixed points(BL13、	AM: fixed points(BL1	Overall response

Bao (2017)	CDC		8.5 years C: 40.5±8.4 years	months C: 14.4 ± 6.7 months	BL15、BL18、BL20、BL23、ST36、SP9、SP6、CV4、CV6、CV12、CV10); de qi required; 30min/session	3、BL15、BL18 、BL20 、BL23 、ST36、SP9、SP6、CV4、CV6 、CV12 、CV10); de qi required; 30min/session	rate
Zhong (2014)	1994 CDC	60 (21/39)	E: 41 ± 9 years C: 41 ± 8 years	E: 14.32 ± 6.33 months C: 15.42 ± 6.05 months	AM: Acu fixed points(bilateral BL13 、BL15、BL18、BL20、BL23、ST36、SP6、SP9; CV12、CV10、CV6、CV4); de qi required; Mox fixed points; 30min/session, 20 sessions for 3 weeks	Acu: fixed points(bilateral BL13、BL15 、BL18 、BL20 、BL23 、ST36、SP6、SP9; CV12、CV10 、CV6、CV4); de qi required; 30min/session, 20 sessions for 3 weeks	Overall response rate
Zhou (2013)	1994 CDC	99 (46/53)	E: AM 40.83 ±9.13 years C: Acu 43.86 ±5.93 years THM 41.32 ±7.02 years	E: AM 13.57 ± 4.51 months C: Acu 12.31 ± 1.03 months THM 14.81 ± 2.78 months	AM: semi-fixed points; main points(BL15、BL20、ST36、SP6、GV20) and additional points(according to TCM syndrome differentiation); de qi	Acu: semi-fixed points: main points(BL15、BL20、ST36、SP6、GV20) and additional points(according to TCM syndrome	Overall response rate;FS-14;SAS;WHOQOL-BREF; adverse reaction

					required; 30min/session, 30 sessions for 6 weeks	differentiation); de qi required; 30min/session, 30 sessions for 6 weeks THM: *Guipi decoction*; one dose daliy for 6 weeks	
Liu Chun (2017)	1994 CDC	60 (26/34)	E: 37.58 ± 12.36 years C: 42.37 ± 14.45 years	E: 11.24 ± 4.07 months C: 10.78 ± 5.12 months	Mox: semi-fixed points; main points(GV29、GV20、GV24、GV14、GB20、BL23、KI3、EX-HN5) and additional points(according to TCM syndrome differentiation);30min/session, 48 sessions for 8 weeks	WH: *Oryzanol*; 20mg/tid for 8 weeks	FS-14;overall response rate;SAS;SDS;PSQI
Zheng (2012)	1994 CDC	77 (31/46)	E: 38.73 ±4.11 years C: 37.08 ±5.32 years	E: 18.41± 5.34 months C: 17.12± 6.03 months	Acu: semi-fixed points;main points and additional points(according to TCM syndrome differentiation); de qi required;30 min/session, 20 sessions for 4 weeks	NC: Streitberger's placebo Acupuncture*	FS-14;VAS;DSI

An (2014)	1994 CDC	80 (35/45)	E: 36.49 ±4.12 years C: 37.08 ±4.69 years	Not reported	Acu: semi-fixed points;main points and additional points(according to TCM syndrome differentiation); de qi required;30 min/session, 20 sessions for 4 weeks	NC: Streitberger's placebo Acupuncture*	FS-14;VAS;DSI
Sai (2018)	1994 CDC	62 (30/32)	E: 38.97 ±6.98 years C: 37.73 ±6.26 years	E: 15.72 ± 5.20 months C: 15.23 ± 5.40 months	Mox: fixed points(BL13 ~BL28);10 min/session, 24 seesions for 8 weeks	Acu: fixed points(BL12~BL28); de qi required; 30min/session, 24 sessions for 8 weeks	FS-14;overall response rate
Zheng (2013)	1994 CDC	59 (19/40)	E: 42 ± 6 years C: 43 ± 6 years	E: 21.72 ± 6.04 months C: 22.48 ± 5.64 months	AM: Acu fixed points(BL20、BL23 LR13、GB25); de qi required; 30min/session, 20 sessions for 4 weeks; Mox fixed points(GV14 ~GV2); 60min/session, 4 sessions for 4 weeks	Acu: fixed points(BL20、BL23 LR13 、GB25); de qi required; 30min/session, 20 sessions for 4 weeks	FS-14;VAS;DSI; overall response rate
Li Rong Zhen	1994 CDC	62 (30/32)	E: 30.77 ±6.98 years	E: 28.10 ± 9.71 months C:	Acu: semi-fixed points; main points(EX-	NC: Usual care	FAI;FS-14;SAS;SDS; overall response

(2016)			C: 28.94 ±6.26 years	26.75 ± 8.30 months	HN1、GV24、GV29; bilateral PC6、SP6); de qi required; 30min/session, 18 sessions for 6 weeks		rate
Yu (2013)	1994 CDC	60 (15/45)	E: 30~50 years C: 30~50 years	E: 0.5~20 years C: 0.5~20 years	Acu: fixed points(BL20、BL18; bilateral LI4、ST36、LR3、SP9); de qi required; 30min/session, 15 sessions for 3 weeks	THM: *Xiaoyao decoction*; one dose daily for 3 weeks	Overall response rate
Zhang (2007)	1994 CDC	50 (29/21)	E: 24~61 years C: 18~57 years	E: 1~6 years C: 0.8~5 years	Acu: semi-fixed points; main points(BL18、BL15、BL20、BL23) and additional points(according to TCM syndrome differentiation); de qi required;30 min/session, 20 sessions for 4 weeks	NC: Streitberger's placebo Acupuncture*	FS-14;overall response rate
Wang (2009)	1994 CDC	64 (32/32)	E: 35.8± 10.7 years C: 38.8± 8.8 years	Not reported	Acu: fixed points(GV20、CV17、CV12、CV4、CV6、LI4、ST36、SP6、LR3、KI3、BL20、BL18、	NC: Needling non-acupuncture points; de qi not required*	FS-14; adverse reaction

					BL23); de qi required; 30min/session, 14 sessions for 6 weeks		
Chen (2010)	1994 CDC	53 (23/30)	E:37.47 ± 12.21 years C: 39.85 ± 13.47 years	Not reported	Acu: fixed points(ST36、KI3); de qi required; 30min/session, 6 sessions for 2 weeks	NC: Streitberger's placebo Acupuncture*	WHOQOL-BREF;FS-14;overall response rate; adverse reaction
Xu (2016)	1994 CDC	62 (35/27)	E: 20~55 years C: 20~55 years	E: 21.31 ± 2.83 months C: 22.87 ± 2.37 months	Acu: semi-fixed points; main points(BL18、BL20、LR3、SP6、GV20、ST36) and additional points(according to TCM syndrome differentiation); de qi required;30 min/session, 20 sessions for 6 weeks	THM: *Xiaoyao decoction*; one dose daily for 3 weeks	FAI;SDS;SF-36;overall response rate
Lu (2014)	1994 CDC	133 (60/73)	E: Acu 20~55 years C: AM 19~53 years NC 18~54	E: Acu 1~5 years C: AM 0.8~6 years NC 1.2~5.3 years	Acu: fixed points(GV20、CV17、CV4、CV6; bilateral ST36、LI4、LR3、SP6); de qi required; 30min/session, 20 sessions for 3 weeks	AM: Acu fixed points(GV20、CV17、CV4、CV6; bilateral ST36、LI4、LR3、SP6); de qi required; Mox fixed points(GV2	FS-14;

			years			0 、 CV4 、 CV6 、 ST36); 30min/session, 20 sessions for 3 weeks NC: Needling non-acupuncture points; de qi not required*	
Wang (2018)	1994 CDC	48 (15/33)	E: 37.3±7.5 years C: 36.5±6.9 years	E: 18.5 ± 6.2 months C: 19.4 ± 7.6 months	AM: Acu fixed points(CV4、CV6; bilateral ST25、ST36、KI3、LR3); de qi required; Mox fixed point(CV4 、 CV6、CV8); 30min/session; 16 sessions for 2 months	Acu: Acu fixed points(CV4 、 CV6; bilateral ST25 、 ST36、KI3、LR3); de qi required; 30min/session; 16 sessions for 2 months	Overall response rate;FAI; adverse reaction
Chen (2018)	1994 CDC	60 (20/40)	E: 40.77 ± 11.61 years C: 41.47 ± 12.21 years	E: 18.70 ± 9.22 months C: 17.83 ± 9.01 months	Acu: fixed points(bilateral BL13 、 BL15、BL18、BL20、BL23); de qi required; 20min/session, 8 sessions for 4 weeks	NC: Needling non-acupuncture points; de qi not required*	FS-14;SCL-90;overall response rate; adverse reaction
Ding (2011)	1994 CDC	60 (35/25)	E: 18~65 years C:	E: 0.5~3 years C: 0.5~3	Acu: fixed points(bilateral BL13 、 BL15、BL18、BL20、BL23);	NC: Needling non-acupuncture points;	FS-14;SAS;SDS; overall response rate

			18~65 years	years	de qi required; 30min/session, 18 sessions for 6 weeks	de qi not required*	
Ye (2009)	1994 CDC	60 (17/43)	E: 20~50 years C: 18~50 years	E: 0.6~20 years E: 0.6~20 years	Acu: fixed points(BL20、BL18、SP9、ST36 ; bilateral LI4、LR3); de qi required; 30min/session, 15 sessions for 3 weeks	THM: *Xiaoyao decoction*; one dose daily for 3 weeks	Overall response rate
Yang (2019)	1994 CDC	57 (22/35)	E: 37.42 ±9.21 years C: 35.79 ± 10.03 years	E: 38.13 ± 14.04 months C: 37.29 ± 15.12 months	AT: Acu fixed points(CV12; bilateral BL18、BL20、ST36、SP9、SP6 、 LR3 、 HT7); de qi required;30 min/session, 20 sessions for 4 weeks THM *Xiaoyao pill*; 8 pills/bid for 4 weeks	THM: *Xiaoyao pill*; 8 pills/bid for 4 weeks	FS-14;SF-36;overall response rate; adverse reaction
Yang (2017)	1994 CDC	15 (0/15)	E: 24.75 ±3.77 years C: 23.57 ±3.10 years	Not reported	Acu: fixed points(BL18、BL20、BL23、ST36、GV20、HT7); de qi required; 30min/session, 16 sessions for 4 weeks	NC: Streitberger's placebo Acupuncture*	FS-14;SPHERE; adverse reaction
Liang	1994	59 (20/39)	E: 31.61	E: 27.39 ± 7.18	AM: Acu fixed	NC: Usual care	FAI;FS-14;SAS;PSQI

(2016)	CDC		±7.60 years C: 30.54 ±6.87 years	months C: 28.57 ± 7.45 months	points(CV4、CV6 、 CV12; bilateral ST25、ST36、LI4、SP6); de qi required; 30min/session Mox fixed points(CV4、CV6); 10min/session 16 sessions for 4 weeks		;overall response rate; adverse reaction
Guan (2017)	1994 CDC	66 (21/45)	E: 40.6± 9.2 years C: 41.4± 9.6 years	E: 14.2 ± 6.2 months C: 14.6 ± 5.8 months	AM: fixed points(CV4、CV6; bilateral BL13、BL15、BL20、BL23、CV12、CV10、GB34、ST36、SP6); de qi required; 30min/session, 10 sessions for 3 weeks	Acu: fixed points(CV4 、 CV6; bilateral BL13 、 BL15 、 BL20 、 BL23 、 CV12 、 CV10 、 GB34 、 ST36、SP6); de qi required; 30min/session, 10 sessions for 3 weeks	Overall response rate
Wu (2015)	1994 CDC	100 (38/62)	E: 40.7± 6.0 years C: 40.5± 5.0 years	E: 5.25 ± 1.34 years C: 5.75 ± 1.50 years	AM: fixed points(bilateral BL13 、BL15、BL18、BL20、BL23、ST36、SP6、SP9、CV12、CV10、CV6、CV4); de qi required; 30min/sessio	Acu: fixed points(bilateral BL13、BL15 、 BL18 、 BL20 、 BL23 、 ST36、SP6、SP9、CV12、CV10 、 CV6、CV4);	Overall response rate; adverse reaction

		n, 10 sessions for 3 weeks	de qi required; 30min/session, 10 sessions for 3 weeks

*The points and treatment course in the NC group were same as in the acupuncture group.
Acronyms: Acu, acupuncture; Mox, moxibustion; AM, acupuncture combined with moxibustion; AT, acupuncture combined with Chinses herbal medicine; MT, moxibustion combined with Chinses herbal medicine; THM, Traditional chinses herbal medicine; WM, western medicine; NC, no control; CDC, Centres for Disease Control and Prevention; FS-14, Fatigue Scale-14; DSI, Depression Status Inventory; FSS, Fatigue Severity Scale; SAS, Self Rating Anxiety Scale; SF-12, SF-20, SF-36: 12, 20, 36-item short-form health survey; VAS, Visual Analogue Scale; WHOQOL-BREF, WHO Quality of Life Assessment Instrument brief version; SDS, Self-Rating Depression Scale; FAI, fatigue assessment instrument; PSQI, Pittsburgh sleep quality index; SPHERE, Somatic and Psychological health report; SCL-90, SymptomChecklist-90; HAMD, Hamilton Depression Scale; HAMA, Hamilton Anxiety Scale.

2.2 General information of each therapy

	Average age	Average course of disease
Acupuncture + Moxibustion	37.94 years	22.34 months
Acupuncture	38.78 years	21.96 months
Moxibustion	40.22 years	14.14 months
Acupuncture + THM	36.95 years	31.28 months
Moxibustion + THM	40.36 years	58.32 months
Traditional Chinses herbal medicine	38.02 years	23.76 months
Western medicine	39.54 years	17.29 months
No control	35.67 years	26.44onths

2.3 Statistics of acupoint frequency

Acupoint	Frequency
BL 13	14
BL 15	16
BL 18	24
BL 20	30
BL 23	22
ST 36	34
CV 4	23
CV 6	17
SP 6	23

3 Risk of bias table of included studies

3.1 The risk of bias graph

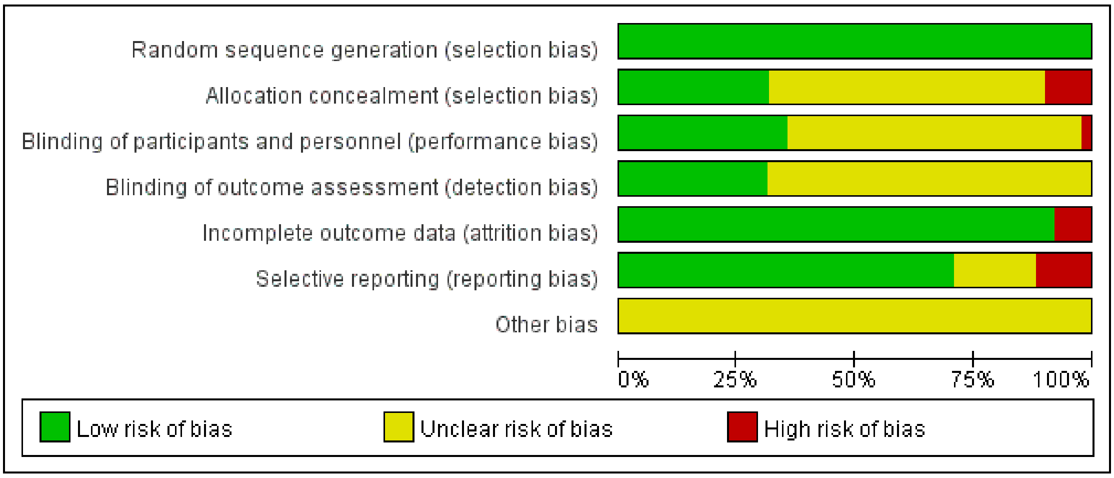

3.2 Risk of bias summary

Study ID	Random sequence generation	Allocation concealment	Blinding participant and personnel	Blinding of outcome assessment	Incomplete outcome data	Selective reporting	Other bias
1.Ding 2011	Low risk of bias	High risk of bias	Unclear risk of bias	Unclear risk of bias	Low risk of bias	Low risk of bias	Unclear risk of bias
2.Yu 2013	Low risk of bias	Unclear risk of bias	Unclear risk of bias	Unclear risk of bias	Low risk of bias	Low risk of bias	Unclear risk of bias
3.Hou 2017	Low risk of bias	Unclear risk of bias	Unclear risk of bias	Unclear risk of bias	High risk of bias	High risk of bias	Unclear risk of bias
4.Liu 2017	Low risk of bias	Unclear risk of bias	Unclear risk of bias	Unclear risk of bias	Low risk of bias	Unclear risk of bias	Unclear risk of bias
5.Liu	Low risk	Low risk	Low risk	Low risk	Low risk	Low risk	Unclear

2017	of bias	of bias	of bias	of bias	of bias	of bias	risk of bias
6.Liu 2018	Low risk of bias	Unclear risk of bias	Low risk of bias	Unclear risk of bias	Low risk of bias	Low risk of bias	Unclear risk of bias
7.Lu 2014	Low risk of bias	Unclear risk of bias	Unclear risk of bias	Unclear risk of bias	Low risk of bias	Unclear risk of bias	Unclear risk of bias
8.Ye 2009	Low risk of bias	Unclear risk of bias	Unclear risk of bias	Unclear risk of bias	Low risk of bias	Low risk of bias	Unclear risk of bias
9.Wu 2015	Low risk of bias	Low risk of bias	Low risk of bias	Unclear risk of bias	Low risk of bias	Low risk of bias	Unclear risk of bias
10.Zhou 2013	Low risk of bias	Low risk of bias	Low risk of bias	Low risk of bias	Low risk of bias	Low risk of bias	Unclear risk of bias
11.Zhou 2018	Low risk of bias	Unclear risk of bias	Unclear risk of bias	Unclear risk of bias	Low risk of bias	Low risk of bias	Unclear risk of bias
12.Jiang 2015	Low risk of bias	Unclear risk of bias	Unclear risk of bias	Unclear risk of bias	Low risk of bias	Low risk of bias	Unclear risk of bias
13.An 2014	Low risk of bias	Unclear risk of bias	Unclear risk of bias	Unclear risk of bias	High risk of bias	High risk of bias	Unclear risk of bias
14.Song 2016	Low risk of bias	Unclear risk of bias	Unclear risk of bias	Unclear risk of bias	Low risk of bias	Low risk of bias	Unclear risk of bias
15.Zhang 2007	Low risk of bias	Low risk of bias	Low risk of bias	Low risk of bias	Low risk of bias	Low risk of bias	Unclear risk of bias
16.Xu 2014	Low risk of bias	Unclear risk of bias	Unclear risk of bias	Unclear risk of bias	Low risk of bias	High risk of bias	Unclear risk of bias
17.Xu 2019	Low risk of bias	Unclear risk of bias	Unclear risk of bias	Unclear risk of bias	Low risk of bias	Unclear risk of bias	Unclear risk of bias
18.Xu 2010	Low risk of bias	Low risk of bias	Low risk of bias	Low risk of bias	Low risk of bias	Low risk of bias	Unclear risk of bias
19.Zhu 2012	Low risk of bias	Low risk of bias	Low risk of bias	Low risk of bias	Low risk of bias	Low risk of bias	Unclear risk of bias

20.Li 2016	Low risk of bias	Unclear risk of bias	Unclear risk of bias	Unclear risk of bias	Low risk of bias	Low risk of bias	Unclear risk of bias
21.Li 2018	Low risk of bias	Unclear risk of bias	Unclear risk of bias	Unclear risk of bias	Low risk of bias	Low risk of bias	Unclear risk of bias
22.Li 2016	Low risk of bias	Low risk of bias	Unclear risk of bias	Unclear risk of bias	High risk of bias	High risk of bias	Unclear risk of bias
23.Yang 2019	Low risk of bias	Unclear risk of bias	Unclear risk of bias	Unclear risk of bias	Low risk of bias	Low risk of bias	Unclear risk of bias
24.Yang 2017	Low risk of bias	Low risk of bias	Low risk of bias	Low risk of bias	Low risk of bias	Low risk of bias	Unclear risk of bias
25.Lin 2010	Low risk of bias	Unclear risk of bias	Unclear risk of bias	Unclear risk of bias	Low risk of bias	Low risk of bias	Unclear risk of bias
26.Liang 2016	Low risk of bias	Low risk of bias	Low risk of bias	Low risk of bias	Low risk of bias	Low risk of bias	Unclear risk of bias
27.Wang 2013	Low risk of bias	Unclear risk of bias	Unclear risk of bias	Unclear risk of bias	Low risk of bias	Unclear risk of bias	Unclear risk of bias
28.Xiong 2005	Low risk of bias	Unclear risk of bias	Unclear risk of bias	Unclear risk of bias	Low risk of bias	Low risk of bias	Unclear risk of bias
29.Wang 2009	Low risk of bias	Low risk of bias	Low risk of bias	Low risk of bias	Low risk of bias	Low risk of bias	Unclear risk of bias
30.Wang 2018	Low risk of bias	High risk of bias	Unclear risk of bias	Unclear risk of bias	Low risk of bias	Unclear risk of bias	Unclear risk of bias
31.Tian 2015	Low risk of bias	Low risk of bias	Low risk of bias	Low risk of bias	Low risk of bias	Low risk of bias	Unclear risk of bias
32.Shi 2015	Low risk of bias	High risk of bias	Low risk of bias	Unclear risk of bias	High risk of bias	High risk of bias	Unclear risk of bias
33.Qi 2017	Low risk of bias	Unclear risk of bias	Unclear risk of bias	Unclear risk of bias	Low risk of bias	Low risk of bias	Unclear risk of bias
34.Luo 2019	Low risk of bias	Unclear risk of	Unclear risk of	Unclear risk of	Low risk of bias	Unclear risk of	Unclear risk of

		bias	bias	bias		bias	bias
35.Luo 2019	Low risk of bias	Low risk of bias	Low risk of bias	Low risk of bias	Low risk of bias	Low risk of bias	Unclear risk of bias
36.Xiao 2014	Low risk of bias	Low risk of bias	Low risk of bias	Low risk of bias	Low risk of bias	Low risk of bias	Unclear risk of bias
37.Hu 2013	Low risk of bias	Unclear risk of bias	Unclear risk of bias	Unclear risk of bias	Low risk of bias	Low risk of bias	Unclear risk of bias
38.Guan 2107	Low risk of bias	Unclear risk of bias	Unclear risk of bias	Unclear risk of bias	Low risk of bias	High risk of bias	Unclear risk of bias
39.Sai 2018	Low risk of bias	High risk of bias	High risk of bias	Unclear risk of bias	Low risk of bias	Low risk of bias	Unclear risk of bias
40.Zhao 2014	Low risk of bias	Unclear risk of bias	Unclear risk of bias	Unclear risk of bias	Low risk of bias	Low risk of bias	Unclear risk of bias
41.Xing 2019	Low risk of bias	Unclear risk of bias	Unclear risk of bias	Unclear risk of bias	Low risk of bias	Low risk of bias	Unclear risk of bias
42.Zheng 2014	Low risk of bias	Unclear risk of bias	Unclear risk of bias	Unclear risk of bias	Low risk of bias	Unclear risk of bias	Unclear risk of bias
43.Zheng 2012	Low risk of bias	Unclear risk of bias	Unclear risk of bias	Unclear risk of bias	Low risk of bias	Low risk of bias	Unclear risk of bias
44.Zheng 2013	Low risk of bias	Unclear risk of bias	Unclear risk of bias	Unclear risk of bias	Low risk of bias	Unclear risk of bias	Unclear risk of bias
45.Hao 2013	Low risk of bias	Low risk of bias	Low risk of bias	Low risk of bias	Low risk of bias	Low risk of bias	Unclear risk of bias
46.Guo 2016	Low risk of bias	Low risk of bias	Low risk of bias	Low risk of bias	Low risk of bias	Low risk of bias	Unclear risk of bias
47.Zhong 2014	Low risk of bias	High risk of bias	Unclear risk of bias	Unclear risk of bias	Low risk of bias	Unclear risk of bias	Unclear risk of bias
48.Chen 2018	Low risk of bias	Low risk of bias	Low risk of bias	Low risk of bias	Low risk of bias	Low risk of bias	Unclear risk of bias
49.Chen	Low risk	Unclear	Unclear	Low risk	Low risk	Low risk	Unclear

2010	of bias	risk of bias	risk of bias	of bias	of bias	of bias	risk of bias
50.Sui 2015	Low risk of bias	Unclear risk of bias	Unclear risk of bias	Unclear risk of bias	Low risk of bias	Low risk of bias	Unclear risk of bias
51.Huang 2017	Low risk of bias	Low risk of bias	Low risk of bias	Low risk of bias	Low risk of bias	Low risk of bias	Unclear risk of bias

4 Analyses of the all trials network

4.1 Estimation of inconsistency: summary of results

Outcome and Data	Number of studies	Number of inconsistent loops out of total (loop-specific method)	Percentage of the inconsistent loops	Number of inconsistent comparisons out of total (SIDE splitting)	Percentage of the inconsistent comparisons	p-value of the DesignbyTreatment test
Overall response rate	46	0/11	0%	1/15	7%	0.3267
FS-14 total score	31	0/5	0%	0/12	0%	0.5026
FS-14 physical score	13	2/3	67%	2/7	29%	0.0004
FS-14 metal score	13	0/3	0%	2/7	29%	0.1187

4.2 Overall response rate of CFS patients

A.Network map

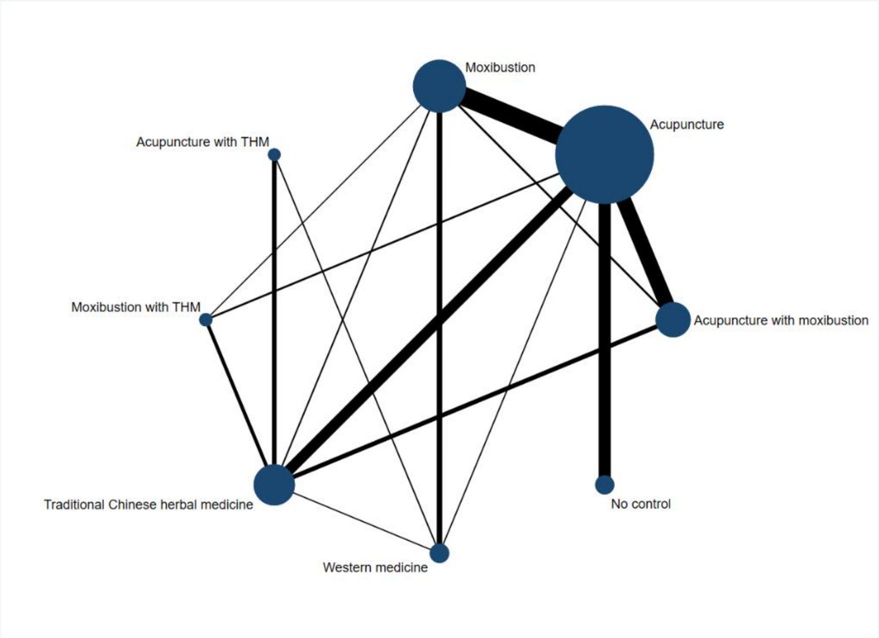

B.Contribution plot

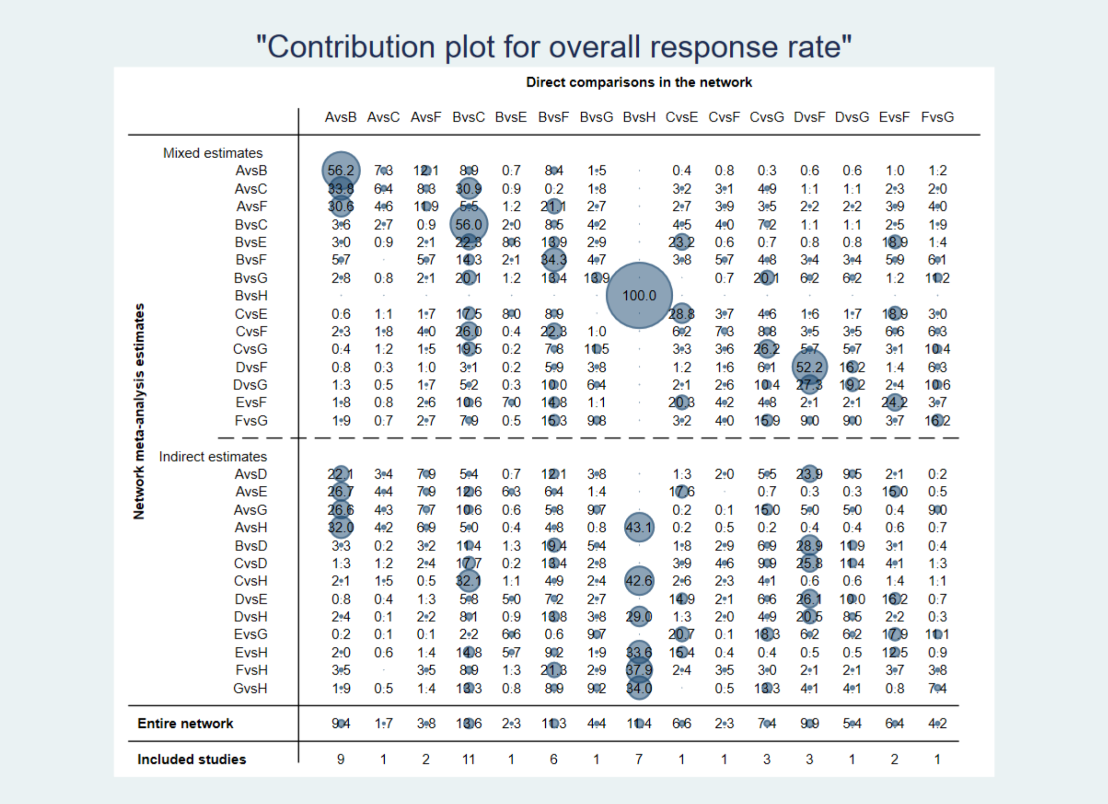

A- Acupuncture with moxibustion; B- Acupuncture; C- Moxibustion; D- Acupuncture with THM; E- Moxibustion with THM; F- Traditional Chinses herbal medicine(THM) G- Western medicine; H- No control

C.Convergence diagraph

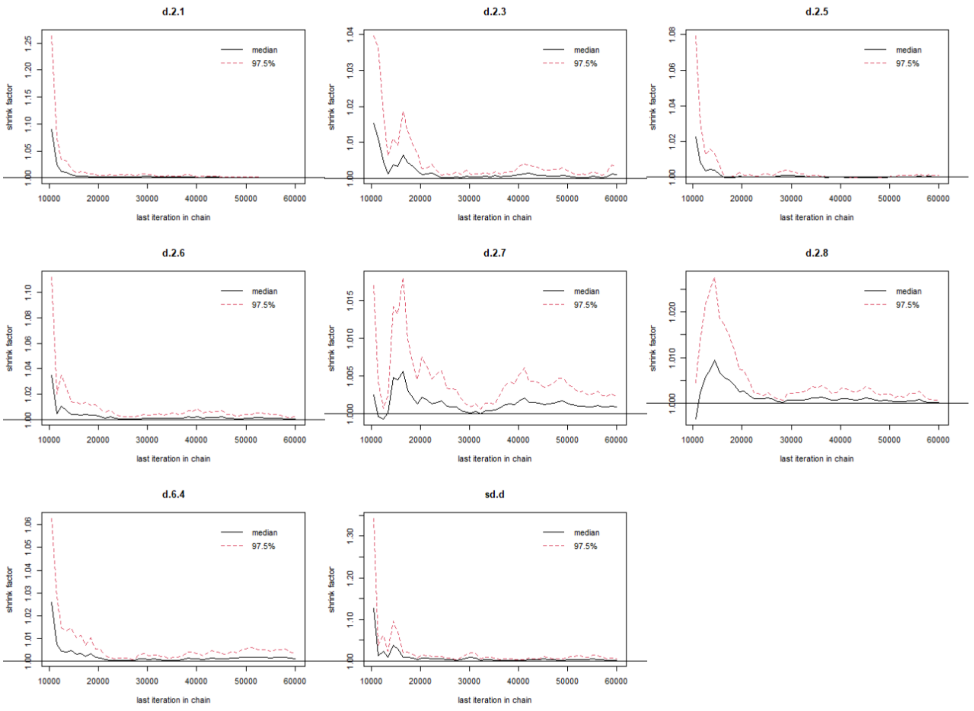

Potential scale reduction factors:

	Point est.	Upper C.I.
**d.2.1**	1	1
**d.2.3**	1	1
**d.2.5**	1	1
**d.2.6**	1	1
**d.2.7**	1	1
**d.2.8**	1	1
**d.6.4**	1	1
**sd.d**	1	1

1- Acupuncture with moxibustion; 2- Acupuncture; 3- Moxibustion; 4- Acupuncture with THM; 5- Moxibustion with THM; 6- Traditional Chinses herbal medicine(THM) 7- Western medicine; 8- No control

D.Trajectories and density diagram

Results on the Log Odds Ratio scale

Iterations = 10010:60000

Thinning interval = 10

Number of chains = 4

Sample size per chain = 5000

1. **Empirical mean and standard deviation for each variable,plus standard error of the mean:**

	Mean	SD	NaiveSE	Time-seriesSE
**d.2.1**	1.1375	0.2641	0.001867	0.002901
**d.2.3**	1.2402	0.2346	0.001659	0.002350
**d.2.5**	1.5955	0.4778	0.003378	0.004990
**d.2.6**	-0.3063	0.2761	0.001952	0.002952
**d.2.7**	-1.2059	0.3912	0.002766	0.004152
**d.2.8**	-2.2683	0.3345	0.002366	0.003430
**d.6.4**	1.1750	0.4729	0.003344	0.005340
**sd.d**	0.4424	0.1952	0.001380	0.005340

2. **Quantiles for each variable:**

	2.5%	25%	50%	75%	97.5%
**d.2.1**	0.62007	0.9627	1.1340	1.3085	1.6642
**d.2.3**	0.78366	1.0830	1.2393	1.3939	1.7094
**d.2.5**	0.64705	1.2817	1.5991	1.9104	2.5382
**d.2.6**	-0.86249	-0.4857	-0.3022	-0.1216	0.2227
**d.2.7**	-1.98612	-1.4614	-1.2038	-0.9470	-0.4376
**d.2.8**	-2.94538	-2.4843	-2.2649	-2.0475	-1.6148
**d.6.4**	0.26413	0.8598	1.1608	1.4841	2.1239
**sd.d**	0.05566	0.3113	0.4492	0.5744	0.8241

3. **Model fit (residual deviance):**
   Dbar: 95.39520 pD: 66.08622 DIC: 161.48142
   94 ata points, ratio 1.015, I²= 3%

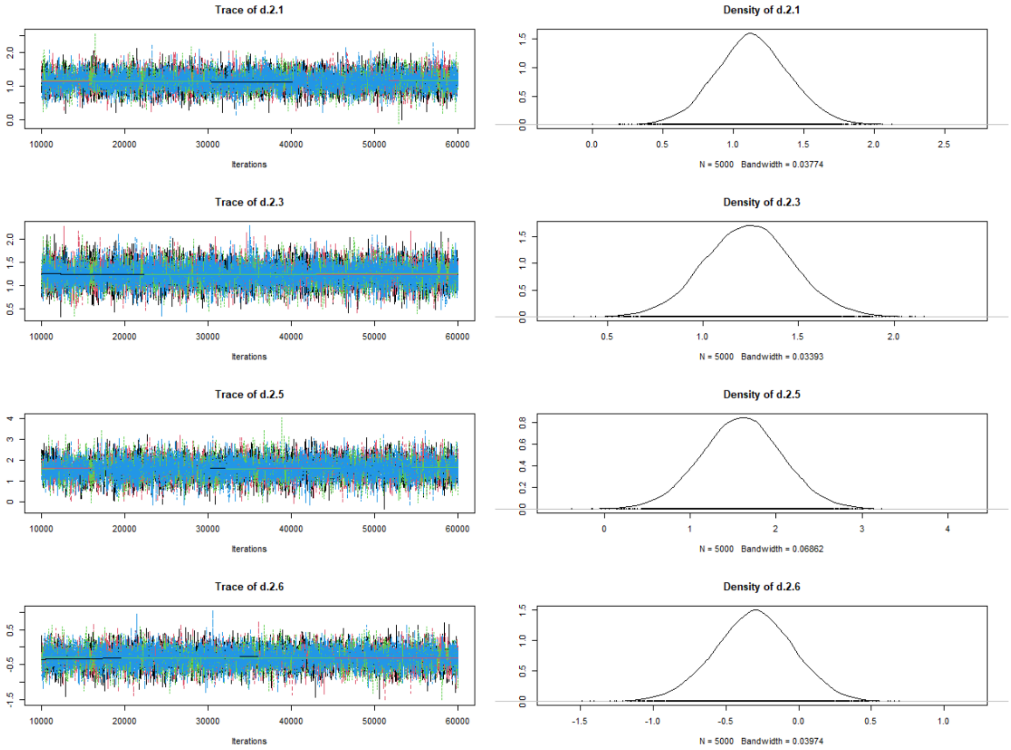

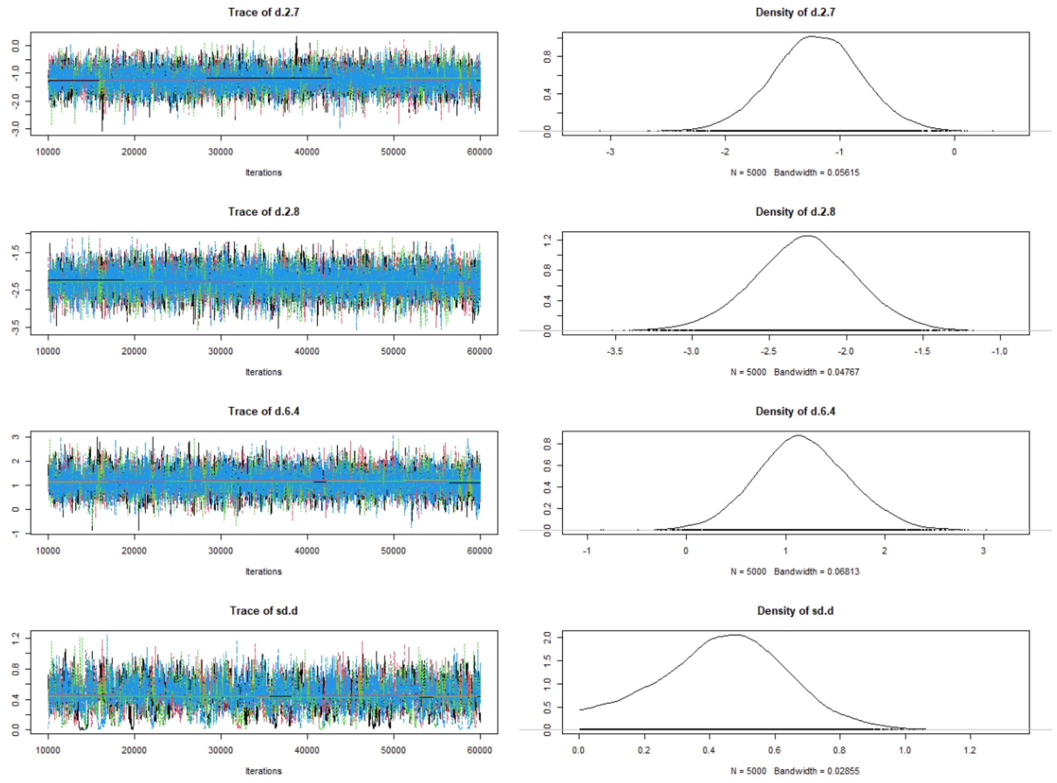

E.Forest plot

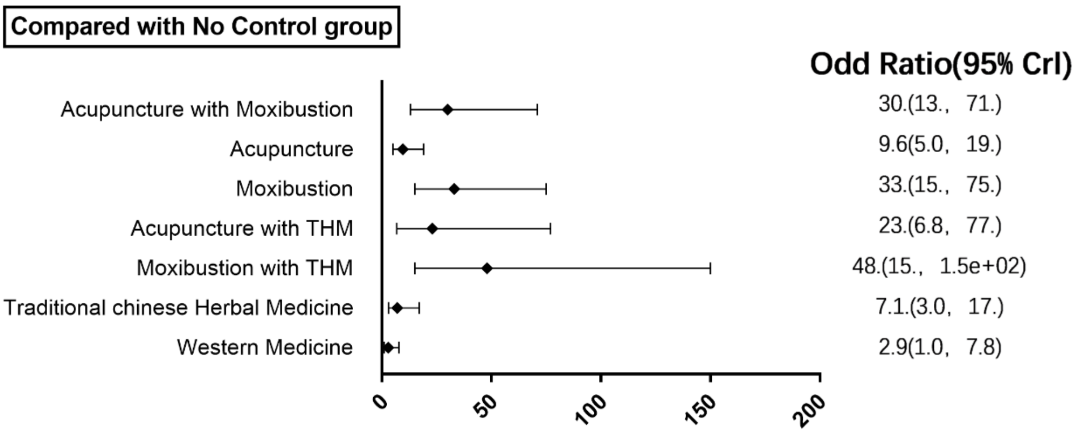

F.League table

Acupuncture with moxibustion			
**3.11 (1.86, 5.28)**	Acupuncture		
0.9 (0.46, 1.77)	0.29 (0.18, 0.46)	Moxibustion	
1.31 (0.42,	0.42 (0.15,	1.46 (0.48,	Acupuncture

4.11)	1.18)	4.31)	with THM				
0.63 (0.22, 1.88)	0.2 (0.08, 0.52)	0.7 (0.27, 1.83)	0.48 (0.13, 1.81)	Moxibustion with THM			
**4.2 (2.15, 8.68)**	1.35 (0.8, 2.37)	**4.68 (2.5, 9.05)**	**3.19 (1.3, 8.36)**	**6.68 (2.64, 17.21)**	Traditional Chinses herbal medicine		
**10.38 (4.25, 26.26)**	**3.33 (1.55, 7.29)**	**11.47 (5.48, 24.89)**	**7.91 (2.65, 25.2)**	**16.58 (5.3, 50.45)**	**2.47 (1.09, 5.51)**	Western medicine	
**29.89 (13.09, 71.88)**	**9.63 (5.03, 19.02)**	**33.19 (15.17, 75.48)**	**22.76 (6.71, 79.94)**	**47.67 (14.93, 147.62)**	**7.12 (3.02, 16.66)**	**2.89 (1.03, 7.91)**	No control

G.Inconsistency and Hetergenneity

a.Loops of Statistical Inconsistency:

Loop	IF	seIF	z_value	p_value	CI_95	Loop_Heterog_tau2
Acu-Mox-MT	1.626	1.033	1.575	0.115	(0.00,3.65)	0.000
Mox-MT-THM	1.171	0.993	1.179	0.238	(0.00,3.12)	0.000
AM-Acu-Mox	1.097	1.056	1.039	0.299	(0.00,3.19)	0.123
Acu-THM-WM	1.015	0.923	1.100	0.271	(0.00,2.82)	0.066
Acu-Mox-THM	0.969	0.767	1.262	0.207	(0.00,2.47)	0.000
Acu-MT-THM	0.513	1.069	0.480	0.631	(0.00,2.61)	0.000
AT-THM-WM	0.495	0.947	0.523	0.601	(0.00,2.35)	0.000
Acu-Mox-WM	0.203	0.741	0.274	0.784	(0.00,1.66)	0.000
Mox-THM-WM	0.182	0.961	0.189	0.850	(0.00,2.07)	0.000
AM-Acu-THM	0.103	0.932	0.111	0.912	(0.00,1.93)	0.287
AM-Mox-THM	0.047	1.324	0.035	0.972	(0.00,2.64)	0.000

b.Side-splitting

**Side**	**Direct**		**Indirect**		**Difference**		**P>|z|**	**tau**
	Coef.	Std. Err.	Coef.	Std. Err.	Coef.	Std. Err.		
**A B**	-1.021869	.2556342	-1.655396	.6791747	.6335276	.7194262	0.379	.2592191
**A C**	-.8185231	.9314647	.1314689	.3259562	-.9499919	.984253	0.334	.2642273
**A F**	-1.51364	.7129481	-1.251295	.3646051	-.2623453	.8001277	0.743	.2784302
**B C**	1.322742	.2254018	.4418885	.4133724	.8808536	.4704145	0.061	.1865741
**B E**	1.029619	.9190925	1.671367	.4468437	-.6417478	1.021959	0.530	.2627105
**B F**	-.5250864	.3049666	.3042393	.3741198	-.8293257	.4782195	0.083	.1964546
**B G**	-.7841189	.6390239	-1.282205	.3985133	.4980865	.7531032	0.508	.288318
**B H***	-2.092675	.2931997	2.445298	533.7928	-4.537973	533.7929	0.993	.2637588
**C E**	1.336846	.4935786	-.4138533	.5124342	1.750699	.7114834	0.014	5.20e-07
**C F**	-.8602012	.7383239	-1.421878	.314402	.5616767	.8024779	0.484	.2936087

**C G**	-2.322688	.460161	-2.214042	.4892641	-.1086459	.6706003	0.871	.2978023
**D F***	-1.059243	.4231174	-1.733843	1.427139	.6745994	1.467598	0.646	.2973091
**D G**	-2.069122	.6728811	-2.002707	.7057275	-.0664147	.9704452	0.945	.2986808
**E F**	-1.026387	.5379574	-2.466222	.5095211	1.439835	.7409602	0.052	.1034935
**F G**	-1.259592	.6100866	-.7585859	.444602	-.5010062	.7571358	0.508	.2920758

* All the evidence about these contrasts comes from the trials which directly compare them.

c.Design-by-treatment test

chi2(9) = 10.30

Prob > chi2 = 0.3267

H.Comparison-adjusted funnel plot

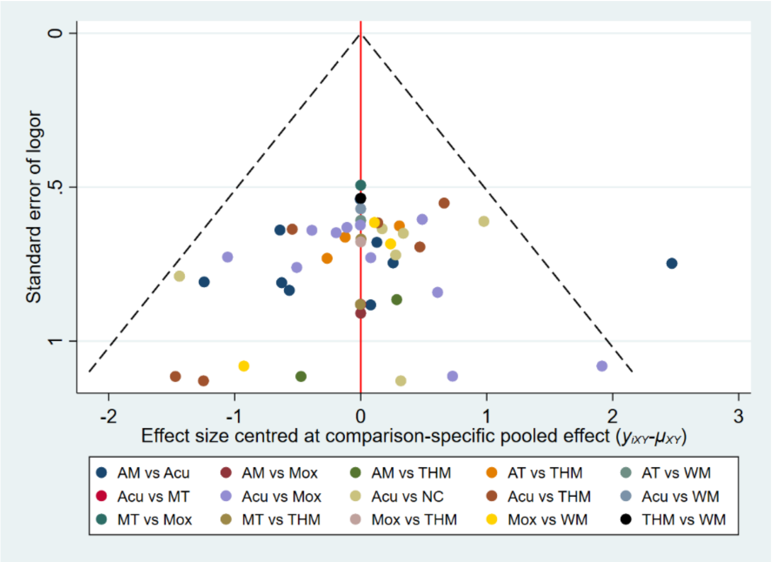

I.SUCRA diagram

a.Ranking probability diagram

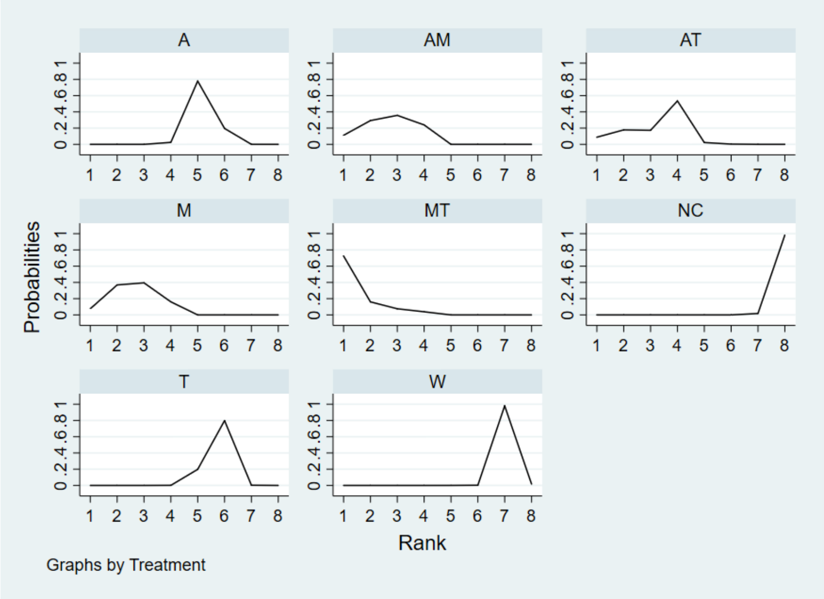

b.Cumulative probability ranking diagram

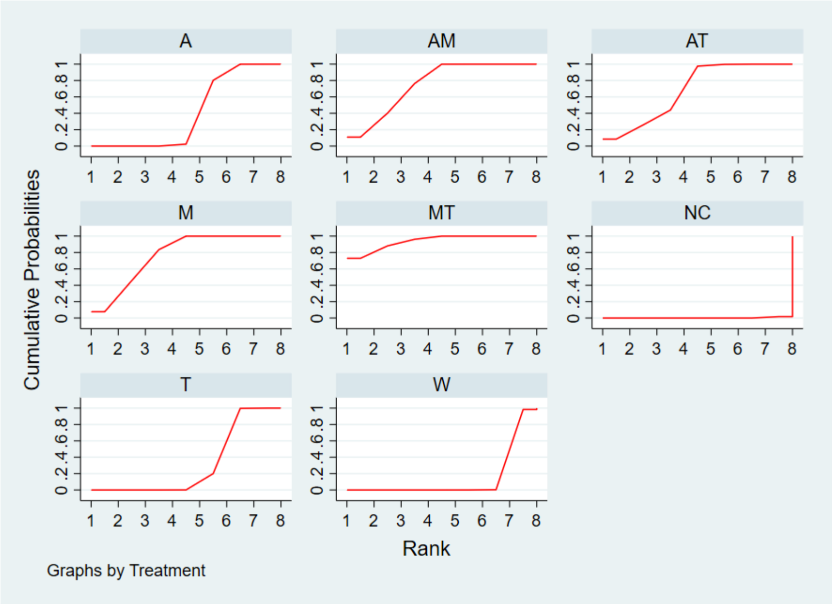

AM-Acupuncture with moxibustion; A-Acupuncture; M-Moxibustion; AT-Acupuncture with THM; MT-Moxibustion with THM; T-Traditional Chinses herbal medicine; W-Western medicine; NC-No control.

J.Subgroup analysis

a. age

	mean	sd	MC_error	val2.5pc	median	val97.5pc
**B**	-0.1776	0.5057	0.009056	-1.16	-0.1808	0.8243
**SUCRA[1]**	0.2049	0.1418	0.002662	0	0.1429	0.4286
**SUCRA[2]**	0.5863	0.05562	5.20E-04	0.4286	0.5714	0.7143
**SUCRA[3]**	0.2367	0.1146	0.001594	0	0.2857	0.4286

**SUCRA[4]**	0.3519	0.133	0.001572	0	0.4286	0.5714
**SUCRA[5]**	0.06816	0.1137	0.001316	0	0	0.4286
**SUCRA[6]**	0.6959	0.05103	4.93E-04	0.5714	0.7143	0.7143
**SUCRA[7]**	0.8575	0.01952	1.24E-04	0.8571	0.8571	0.8571
**SUCRA[8]**	0.9985	0.0147	7.30E-05	1	1	1
**SUCRAz[1]**	0.1515	0.1305	9.75E-04	0	0.1429	0.4286
**SUCRAz[2]**	0.5864	0.05553	5.19E-04	0.4286	0.5714	0.7143
**SUCRAz[3]**	0.2625	0.1067	9.08E-04	0	0.2857	0.4286
**SUCRAz[4]**	0.3645	0.1262	0.001353	0	0.4286	0.5714
**SUCRAz[5]**	0.08316	0.1198	0.00117	0	0	0.4286
**SUCRAz[6]**	0.696	0.05098	4.93E-04	0.5714	0.7143	0.7143
**SUCRAz[7]**	0.8575	0.0195	1.24E-04	0.8571	0.8571	0.8571
**SUCRAz[8]**	0.9985	0.01471	7.31E-05	1	1	1
**or[1,2]**	0.2944	0.1191	0.002492	0.1224	0.275	0.5726
**or[1,3]**	1.025	0.4621	0.009107	0.3994	0.9336	2.153
**or[1,4]**	0.7837	0.5091	0.008041	0.2096	0.6614	2.067
**or[1,5]**	1.714	1.013	0.01678	0.4671	1.487	4.281
**or[1,6]**	0.2267	0.1093	0.00192	0.08617	0.2102	0.4606
**or[1,7]**	0.09516	0.05516	9.16E-04	0.03008	0.08486	0.2215
**or[1,8]**	0.03226	0.05057	3.44E-04	0.01038	0.02878	0.07384
**or[2,3]**	3.506	0.7393	0.007745	2.299	3.424	5.182
**or[2,4]**	2.714	1.436	0.01639	0.975	2.407	6.188
**or[2,5]**	5.918	2.705	0.03387	2.266	5.372	12.64
**or[2,6]**	0.7849	0.2038	0.002312	0.4647	0.7619	1.23
**or[2,7]**	0.3257	0.1161	0.001323	0.1541	0.3077	0.6003
**or[2,8]**	0.1094	0.05706	4.10E-04	0.05647	0.105	0.1879
**or[3,4]**	0.7965	0.4307	0.00496	0.2719	0.7038	1.835
**or[3,5]**	1.717	0.7624	0.009286	0.664	1.571	3.598
**or[3,6]**	0.2325	0.7771	0.001634	0.1242	0.2218	0.3862
**or[3,7]**	0.09483	0.09208	4.08E-04	0.04505	0.09029	0.1704
**or[3,8]**	0.0337	0.6257	0.001192	0.01443	0.03067	0.06175
**or[4,5]**	2.666	1.759	0.02209	0.6728	2.236	7.251
**or[4,6]**	0.3438	0.4602	0.00174	0.1353	0.3165	0.7023
**or[4,7]**	0.1436	0.09081	8.02E-04	0.0468	0.1272	0.3333
**or[4,8]**	0.0511	0.3544	7.88E-04	0.01422	0.04297	0.1282
**or[5,6]**	0.1693	7.413	0.01357	0.05921	0.1415	0.3336
**or[5,7]**	0.06671	0.8261	0.00156	0.02048	0.05693	0.1563
**or[5,8]**	0.03332	5.986	0.01095	0.006811	0.01936	0.05605
**or[6,7]**	0.4304	0.1624	0.001651	0.1967	0.404	0.8221
**or[6,8]**	0.1479	0.06014	6.59E-04	0.0631	0.1378	0.2954
**or[7,8]**	0.3778	0.1882	0.001944	0.138	0.3398	0.8432
**orz[1,1,2]**	0.2401	0.07909	3.94E-04	0.1206	0.2296	0.4195
**orz[1,1,3]**	0.842	0.3329	0.002369	0.3692	0.785	1.642
**orz[1,1,4]**	0.6521	0.4486	0.004235	0.1808	0.5516	1.702

**orz[1,1,5]**	1.422	0.8246	0.008581	0.4161	1.236	3.511
**orz[1,1,6]**	0.1886	0.08641	6.70E-04	0.07734	0.1746	0.3796
**orz[1,1,7]**	0.07828	0.04163	3.51E-04	0.0275	0.07038	0.1747
**orz[1,1,8]**	0.0264	0.04752	1.47E-04	0.009776	0.02394	0.05612
**orz[1,2,3]**	3.506	0.7393	0.007745	2.299	3.424	5.182
**orz[1,2,4]**	2.714	1.436	0.01639	0.975	2.407	6.188
**orz[1,2,5]**	5.918	2.705	0.03387	2.266	5.372	12.64
**orz[1,2,6]**	0.7849	0.2038	0.002312	0.4647	0.7619	1.23
**orz[1,2,7]**	0.3257	0.1161	0.001323	0.1541	0.3077	0.6003
**orz[1,2,8]**	0.1094	0.05706	4.10E-04	0.05647	0.105	0.1879
**orz[1,3,4]**	0.7965	0.4307	0.00496	0.2719	0.7038	1.835
**orz[1,3,5]**	1.717	0.7624	0.009286	0.664	1.571	3.598
**orz[1,3,6]**	0.2325	0.7771	0.001634	0.1242	0.2218	0.3862
**orz[1,3,7]**	0.09483	0.09208	4.08E-04	0.04505	0.09029	0.1704
**orz[1,3,8]**	0.0337	0.6257	0.001192	0.01443	0.03067	0.06175
**orz[1,4,5]**	2.666	1.759	0.02209	0.6728	2.236	7.251
**orz[1,4,6]**	0.3438	0.4602	0.00174	0.1353	0.3165	0.7023
**orz[1,4,7]**	0.1436	0.09081	8.02E-04	0.0468	0.1272	0.3333
**orz[1,4,8]**	0.0511	0.3544	7.88E-04	0.01422	0.04297	0.1282
**orz[1,5,6]**	0.1693	7.413	0.01357	0.05921	0.1415	0.3336
**orz[1,5,7]**	0.06671	0.8261	0.00156	0.02048	0.05693	0.1563
**orz[1,5,8]**	0.03332	5.986	0.01095	0.006811	0.01936	0.05605
**orz[1,6,7]**	0.4304	0.1624	0.001651	0.1967	0.404	0.8221
**orz[1,6,8]**	0.1479	0.06014	6.59E-04	0.0631	0.1378	0.2954
**orz[1,7,8]**	0.3778	0.1882	0.001944	0.138	0.3398	0.8432
**sd**	0.2676	0.1682	0.003084	0.01381	0.2523	0.6262
**totresdev**	91.72	13.08	0.08468	69.53	91.33	116.2

or: Average age≤40 years orz: Average age>40 years

b. course of disease

	**mean**	**sd**	**MC_error**	**val2.5pc**	**median**	**val97.5pc**
**B**	-0.00362	0.5349	0.006154	-1.067	-0.00158	1.036
**SUCRA[1]**	0.1721	0.13	0.002063	0	0.1429	0.4286
**SUCRA[2]**	0.589	0.05691	5.27E-04	0.4286	0.5714	0.7143
**SUCRA[3]**	0.2549	0.1124	0.001385	0	0.2857	0.4286
**SUCRA[4]**	0.3591	0.1295	0.001412	0	0.4286	0.5714
**SUCRA[5]**	0.07504	0.1198	0.00133	0	0	0.4286
**SUCRA[6]**	0.6937	0.05301	4.96E-04	0.5714	0.7143	0.7143
**SUCRA[7]**	0.8575	0.01882	7.82E-05	0.8571	0.8571	0.8571
**SUCRA[8]**	0.9987	0.01413	6.64E-05	1	1	1
**SUCRAz[1]**	0.1806	0.1488	9.21E-04	0	0.1429	0.4286
**SUCRAz[2]**	0.5889	0.05708	5.28E-04	0.4286	0.5714	0.7143
**SUCRAz[3]**	0.2482	0.1111	9.55E-04	0	0.2857	0.4286
**SUCRAz[4]**	0.3526	0.1302	0.001311	0	0.4286	0.5714

**SUCRAz[5]**	0.07996	0.1171	0.001074	0	0	0.4286
**SUCRAz[6]**	0.6936	0.05328	4.97E-04	0.5714	0.7143	0.7143
**SUCRAz[7]**	0.8575	0.01882	7.83E-05	0.8571	0.8571	0.8571
**SUCRAz[8]**	0.9987	0.01411	6.63E-05	1	1	1
**or[1,2]**	0.2551	0.07716	0.001388	0.1337	0.2462	0.4274
**or[1,3]**	0.9	0.3228	0.00546	0.4274	0.8479	1.664
**or[1,4]**	0.7021	0.4068	0.005535	0.2087	0.6135	1.726
**or[1,5]**	1.506	0.841	0.01146	0.4818	1.342	3.549
**or[1,6]**	0.2024	0.07818	0.001217	0.09252	0.1922	0.3736
**or[1,7]**	0.08269	0.03805	5.50E-04	0.03166	0.07561	0.1741
**or[1,8]**	0.02771	0.03973	2.35E-04	0.01103	0.02529	0.05727
**or[2,3]**	3.542	0.7373	0.007898	2.342	3.457	5.23
**or[2,4]**	2.785	1.42	0.01574	0.9975	2.49	6.319
**or[2,5]**	5.944	2.683	0.02988	2.303	5.435	12.62
**or[2,6]**	0.8018	0.2044	0.002285	0.4792	0.7804	1.255
**or[2,7]**	0.3267	0.1155	0.001217	0.1564	0.3096	0.5989
**or[2,8]**	0.1083	0.0534	4.17E-04	0.05555	0.1034	0.1861
**or[3,4]**	0.8093	0.4249	0.004693	0.2798	0.721	1.875
**or[3,5]**	1.707	0.7532	0.008248	0.6706	1.574	3.56
**or[3,6]**	0.235	0.7855	0.001595	0.1262	0.2254	0.3898
**or[3,7]**	0.09414	0.09218	3.84E-04	0.04508	0.08943	0.1685
**or[3,8]**	0.03297	0.6073	0.001131	0.01412	0.02985	0.06033
**or[4,5]**	2.598	1.851	0.01877	0.6587	2.173	6.966
**or[4,6]**	0.3419	0.443	0.001818	0.1334	0.3139	0.7061
**or[4,7]**	0.1405	0.08946	8.32E-04	0.04493	0.1241	0.3284
**or[4,8]**	0.04946	0.3369	8.83E-04	0.01361	0.04157	0.1242
**or[5,6]**	0.1714	7.345	0.01343	0.06112	0.1439	0.343
**or[5,7]**	0.0665	0.8118	0.001532	0.02034	0.05687	0.1579
**or[5,8]**	0.03237	5.695	0.01041	0.006755	0.01906	0.05464
**or[6,7]**	0.4233	0.1614	0.001687	0.192	0.3964	0.8067
**or[6,8]**	0.1434	0.05896	6.84E-04	0.06083	0.1326	0.2839
**or[7,8]**	0.3735	0.1866	0.002132	0.1344	0.3349	0.8373
**orz[1,1,2]**	0.2687	0.1282	5.79E-04	0.09932	0.2452	0.5732
**orz[1,1,3]**	0.9515	0.4948	0.003003	0.3176	0.848	2.183
**orz[1,1,4]**	0.7481	0.5489	0.004547	0.1684	0.6085	2.152
**orz[1,1,5]**	1.594	1.1	0.008701	0.3834	1.327	4.37
**orz[1,1,6]**	0.2156	0.1232	7.94E-04	0.06807	0.1908	0.5053
**orz[1,1,7]**	0.08781	0.05686	3.86E-04	0.02435	0.07543	0.2229
**orz[1,1,8]**	0.0293	0.06699	1.95E-04	0.008461	0.02535	0.07188
**orz[1,2,3]**	3.542	0.7373	0.007898	2.342	3.457	5.23
**orz[1,2,4]**	2.785	1.42	0.01574	0.9975	2.49	6.319
**orz[1,2,5]**	5.944	2.683	0.02988	2.303	5.435	12.62
**orz[1,2,6]**	0.8018	0.2044	0.002285	0.4792	0.7804	1.255
**orz[1,2,7]**	0.3267	0.1155	0.001217	0.1564	0.3096	0.5989

**orz[1,2,8]**	0.1083	0.0534	4.17E-04	0.05555	0.1034	0.1861
**orz[1,3,4]**	0.8093	0.4249	0.004693	0.2798	0.721	1.875
**orz[1,3,5]**	1.707	0.7532	0.008248	0.6706	1.574	3.56
**orz[1,3,6]**	0.235	0.7855	0.001595	0.1262	0.2254	0.3898
**orz[1,3,7]**	0.09414	0.09218	3.84E-04	0.04508	0.08943	0.1685
**orz[1,3,8]**	0.03297	0.6073	0.001131	0.01412	0.02985	0.06033
**orz[1,4,5]**	2.598	1.851	0.01877	0.6587	2.173	6.966
**orz[1,4,6]**	0.3419	0.443	0.001818	0.1334	0.3139	0.7061
**orz[1,4,7]**	0.1405	0.08946	8.32E-04	0.04493	0.1241	0.3284
**orz[1,4,8]**	0.04946	0.3369	8.83E-04	0.01361	0.04157	0.1242
**orz[1,5,6]**	0.1714	7.345	0.01343	0.06112	0.1439	0.343
**orz[1,5,7]**	0.0665	0.8118	0.001532	0.02034	0.05687	0.1579
**orz[1,5,8]**	0.03237	5.695	0.01041	0.006755	0.01906	0.05464
**orz[1,6,7]**	0.4233	0.1614	0.001687	0.192	0.3964	0.8067
**orz[1,6,8]**	0.1434	0.05896	6.84E-04	0.06083	0.1326	0.2839
**orz[1,7,8]**	0.3735	0.1866	0.002132	0.1344	0.3349	0.8373
**sd**	0.2687	0.171	0.003137	0.01302	0.2532	0.6349
**totresdev**	91.78	12.7	0.08098	69.47	91.41	116.1

or: Average course of disease≤1 year orz: Average course of disease>1 year

c. Duration of treatment

**mean**	**sd**	**MC_error**	**val2.5pc**	**median**	**val97.5pc**
**B**	-3.47	11.14	0.3615	-17.92	-6.031
**SUCRA[1]**	0.6208	0.4668	0.01511	0	1
**SUCRA[2]**	0.4956	0.1027	0.002473	0.2857	0.4286
**SUCRA[3]**	0.1115	0.1117	0.002515	0	0.1429
**SUCRA[4]**	0.2285	0.127	0.002645	0	0.2857
**SUCRA[5]**	0.2557	0.1553	0.003063	0	0.2857
**SUCRA[6]**	0.5975	0.09265	0.002378	0.4286	0.5714
**SUCRA[7]**	0.7882	0.08417	0.002303	0.7143	0.7143
**SUCRA[8]**	0.9021	0.08482	0.002351	0.7143	0.8571
**SUCRAz[1]**	0.1187	0.1258	0.001347	0	0.1429
**SUCRAz[2]**	0.5838	0.07525	0.00103	0.4286	0.5714
**SUCRAz[3]**	0.1448	0.1228	0.001664	0	0.1429
**SUCRAz[4]**	0.2935	0.142	0.002071	0	0.2857
**SUCRAz[5]**	0.3177	0.176	0.00266	0	0.4286
**SUCRAz[6]**	0.6852	0.0609	7.96E-04	0.5714	0.7143
**SUCRAz[7]**	0.8722	0.04683	5.48E-04	0.8571	0.8571
**SUCRAz[8]**	0.984	0.04531	5.43E-04	0.8571	1
**or[1,2]**	3.65E+06	3.22E+07	1.01E+06	1.45E-08	101.3
**or[1,3]**	1.39E+07	1.27E+08	3.93E+06	5.22E-08	374.4
**or[1,4]**	1.08E+07	1.16E+08	3.53E+06	3.17E-08	320.8
**or[1,5]**	1.01E+07	1.01E+08	2.82E+06	2.64E-08	352.5
**or[1,6]**	3.06E+06	2.88E+07	901800	1.08E-08	99.22

**or[1,7]**	1.17E+06	1.02E+07	315700	3.97E-09	36.9
**or[1,8]**	6.38E+05	5.36E+06	1.64E+05	2.33E-09	21.41
**or[2,3]**	3.842	0.8908	0.01332	2.424	3.725
**or[2,4]**	2.852	1.87	0.02274	1.075	2.555
**or[2,5]**	2.873	18.98	0.04744	0.7207	2.294
**or[2,6]**	0.8218	0.2169	0.003509	0.4791	0.7962
**or[2,7]**	0.3382	0.1126	0.001582	0.1686	0.3222
**or[2,8]**	0.1939	0.3103	0.001117	0.09551	0.1842
**or[3,4]**	0.7717	0.7792	0.006783	0.2701	0.6789
**or[3,5]**	0.7871	10.17	0.02064	0.1832	0.614
**or[3,6]**	0.2228	0.1079	0.001146	0.1141	0.2121
**or[3,7]**	0.09028	0.0477	4.23E-04	0.04461	0.08646
**or[3,8]**	0.05507	1.149	0.002129	0.02221	0.04899
**or[4,5]**	1.165	6.767	0.01777	0.2391	0.9069
**or[4,6]**	0.3389	1.425	0.003339	0.1395	0.3123
**or[4,7]**	0.1405	0.2899	0.001165	0.0481	0.1266
**or[4,8]**	0.1007	9.213	0.01685	0.02352	0.07172
**or[5,6]**	0.3996	0.471	0.003481	0.1085	0.3488
**or[5,7]**	0.1717	0.2158	0.001845	0.03627	0.1395
**or[5,8]**	0.1114	5.761	0.01056	0.01921	0.07909
**or[6,7]**	0.4285	0.1541	0.002083	0.2062	0.4039
**or[6,8]**	0.2519	0.4052	0.001816	0.1016	0.2311
**or[7,8]**	0.6541	9.663	0.01954	0.2304	0.5696
**orz[1,1,2]**	0.2598	0.06602	2.96E-04	0.1563	0.2531
**orz[1,1,3]**	0.9968	0.4116	0.003638	0.5024	0.943
**orz[1,1,4]**	0.7397	1.029	0.006403	0.2423	0.6467
**orz[1,1,5]**	0.7497	8.081	0.01742	0.1664	0.5791
**orz[1,1,6]**	0.2128	0.1033	9.11E-04	0.1021	0.2012
**orz[1,1,7]**	0.08767	0.04142	4.15E-04	0.03674	0.0812
**orz[1,1,8]**	0.05366	1.789	0.003364	0.02068	0.04639
**orz[1,2,3]**	3.842	0.8908	0.01332	2.424	3.725
**orz[1,2,4]**	2.852	1.87	0.02274	1.075	2.555
**orz[1,2,5]**	2.873	18.98	0.04744	0.7207	2.294
**orz[1,2,6]**	0.8218	0.2169	0.003509	0.4791	0.7962
**orz[1,2,7]**	0.3382	0.1126	0.001582	0.1686	0.3222
**orz[1,2,8]**	0.1939	0.3103	0.001117	0.09551	0.1842
**orz[1,3,4]**	0.7717	0.7792	0.006783	0.2701	0.6789
**orz[1,3,5]**	0.7871	10.17	0.02064	0.1832	0.614
**orz[1,3,6]**	0.2228	0.1079	0.001146	0.1141	0.2121
**orz[1,3,7]**	0.09028	0.0477	4.23E-04	0.04461	0.08646
**orz[1,3,8]**	0.05507	1.149	0.002129	0.02221	0.04899
**orz[1,4,5]**	1.165	6.767	0.01777	0.2391	0.9069
**orz[1,4,6]**	0.3389	1.425	0.003339	0.1395	0.3123
**orz[1,4,7]**	0.1405	0.2899	0.001165	0.0481	0.1266

**orz[1,4,8]**	0.1007	9.213	0.01685	0.02352	0.07172
**orz[1,5,6]**	0.3996	0.471	0.003481	0.1085	0.3488
**orz[1,5,7]**	0.1717	0.2158	0.001845	0.03627	0.1395
**orz[1,5,8]**	0.1114	5.761	0.01056	0.01921	0.07909
**orz[1,6,7]**	0.4285	0.1541	0.002083	0.2062	0.4039
**orz[1,6,8]**	0.2519	0.4052	0.001816	0.1016	0.2311
**orz[1,7,8]**	0.6541	9.663	0.01954	0.2304	0.5696
**sd**	0.1618	0.123	0.002106	0.007746	0.1367
**totresdev**	66.83	10.84	0.05295	49.31	66.17

or: Duration of treatment≤4 weeks orz: Duration of treatment>4 weeks

d. Principle of acupoint selection

	**mean**	**sd**	**MC_error**	**val2.5pc**	**median**	**val97.5pc**
**B**	0.03398	0.6015	0.006248	-1.168	0.03928	1.201
**SUCRA[1]**	0.1684	0.129	0.002071	0	0.1429	0.4286
**SUCRA[2]**	0.59	0.05747	6.03E-04	0.5714	0.5714	0.7143
**SUCRA[3]**	0.2567	0.1106	0.001326	0	0.2857	0.4286
**SUCRA[4]**	0.3613	0.1282	0.001391	0	0.4286	0.4286
**SUCRA[5]**	0.07463	0.1186	0.001249	0	0	0.4286
**SUCRA[6]**	0.6928	0.05377	5.77E-04	0.5714	0.7143	0.7143
**SUCRA[7]**	0.8576	0.01927	7.89E-05	0.8571	0.8571	0.8571
**SUCRA[8]**	0.9985	0.0147	6.75E-05	1	1	1
**SUCRAz[1]**	0.1913	0.1575	9.49E-04	0	0.1429	0.4286
**SUCRAz[2]**	0.5894	0.05818	6.04E-04	0.4286	0.5714	0.7143
**SUCRAz[3]**	0.2421	0.1104	9.33E-04	0	0.2857	0.4286
**SUCRAz[4]**	0.3498	0.1295	0.001291	0	0.4286	0.4286
**SUCRAz[5]**	0.07876	0.1146	9.56E-04	0	0	0.4286
**SUCRAz[6]**	0.6925	0.05464	5.80E-04	0.5714	0.7143	0.7143
**SUCRAz[7]**	0.8576	0.01929	7.90E-05	0.8571	0.8571	0.8571
**SUCRAz[8]**	0.9985	0.01469	6.74E-05	1	1	1
**or[1,2]**	0.2513	0.0743	0.001339	0.1364	0.2425	0.4204
**or[1,3]**	0.889	0.3204	0.005348	0.4287	0.8433	1.641
**or[1,4]**	0.6901	0.407	0.00575	0.2131	0.5967	1.707
**or[1,5]**	1.493	0.7982	0.01178	0.4774	1.329	3.485
**or[1,6]**	0.1999	0.08491	0.001237	0.09374	0.1888	0.3689
**or[1,7]**	0.08207	0.04213	5.89E-04	0.03097	0.07517	0.1741
**or[1,8]**	0.02747	0.04339	2.18E-04	0.01094	0.02523	0.05609
**or[2,3]**	3.551	0.7407	0.008502	2.33	3.462	5.233
**or[2,4]**	2.775	1.437	0.01672	1.007	2.475	6.332
**or[2,5]**	5.979	2.699	0.03351	2.313	5.463	12.65
**or[2,6]**	0.8028	0.208	0.002513	0.4784	0.7789	1.262
**or[2,7]**	0.3276	0.1156	0.001253	0.1544	0.3109	0.5989
**or[2,8]**	0.109	0.05713	4.18E-04	0.05611	0.1044	0.1886
**or[3,4]**	0.8041	0.4324	0.005088	0.2781	0.7103	1.889

**or[3,5]**	1.713	0.7541	0.008587	0.6707	1.581	3.596
**or[3,6]**	0.2349	0.7887	0.001689	0.1263	0.2242	0.3942
**or[3,7]**	0.09423	0.09234	3.77E-04	0.04463	0.0894	0.1701
**or[3,8]**	0.03311	0.6017	0.001145	0.0143	0.03011	0.06078
**or[4,5]**	2.625	1.71	0.02052	0.6644	2.194	7.041
**or[4,6]**	0.3431	0.4367	0.001749	0.1351	0.316	0.6993
**or[4,7]**	0.141	0.08781	8.01E-04	0.04561	0.1251	0.3261
**or[4,8]**	0.04962	0.3171	7.03E-04	0.01362	0.04201	0.1241
**or[5,6]**	0.1706	7.322	0.01344	0.05996	0.1433	0.3357
**or[5,7]**	0.06627	0.8071	0.001517	0.02069	0.05651	0.1578
**or[5,8]**	0.03222	5.603	0.01025	0.006701	0.01908	0.05439
**or[6,7]**	0.4239	0.1611	0.001658	0.1921	0.3977	0.8113
**or[6,8]**	0.1442	0.0593	6.76E-04	0.06088	0.1338	0.2889
**or[7,8]**	0.3745	0.1882	0.001956	0.1364	0.3362	0.8375
**orz[1,1,2]**	0.2864	0.1615	7.63E-04	0.08576	0.2518	0.6888
**orz[1,1,3]**	1.017	0.6175	0.003647	0.2791	0.8739	2.587
**orz[1,1,4]**	0.7946	0.6595	0.005175	0.1531	0.6214	2.461
**orz[1,1,5]**	1.71	1.297	0.0104	0.3455	1.378	5.013
**orz[1,1,6]**	0.2298	0.1483	9.25E-04	0.0604	0.196	0.5969
**orz[1,1,7]**	0.09383	0.06673	4.38E-04	0.02175	0.07771	0.2594
**orz[1,1,8]**	0.03131	0.06027	1.89E-04	0.007635	0.02611	0.0839
**orz[1,2,3]**	3.551	0.7407	0.008502	2.33	3.462	5.233
**orz[1,2,4]**	2.775	1.437	0.01672	1.007	2.475	6.332
**orz[1,2,5]**	5.979	2.699	0.03351	2.313	5.463	12.65
**orz[1,2,6]**	0.8028	0.208	0.002513	0.4784	0.7789	1.262
**orz[1,2,7]**	0.3276	0.1156	0.001253	0.1544	0.3109	0.5989
**orz[1,2,8]**	0.109	0.05713	4.18E-04	0.05611	0.1044	0.1886
**orz[1,3,4]**	0.8041	0.4324	0.005088	0.2781	0.7103	1.889
**orz[1,3,5]**	1.713	0.7541	0.008587	0.6707	1.581	3.596
**orz[1,3,6]**	0.2349	0.7887	0.001689	0.1263	0.2242	0.3942
**orz[1,3,7]**	0.09423	0.09234	3.77E-04	0.04463	0.0894	0.1701
**orz[1,3,8]**	0.03311	0.6017	0.001145	0.0143	0.03011	0.06078
**orz[1,4,5]**	2.625	1.71	0.02052	0.6644	2.194	7.041
**orz[1,4,6]**	0.3431	0.4367	0.001749	0.1351	0.316	0.6993
**orz[1,4,7]**	0.141	0.08781	8.01E-04	0.04561	0.1251	0.3261
**orz[1,4,8]**	0.04962	0.3171	7.03E-04	0.01362	0.04201	0.1241
**orz[1,5,6]**	0.1706	7.322	0.01344	0.05996	0.1433	0.3357
**orz[1,5,7]**	0.06627	0.8071	0.001517	0.02069	0.05651	0.1578
**orz[1,5,8]**	0.03222	5.603	0.01025	0.006701	0.01908	0.05439
**orz[1,6,7]**	0.4239	0.1611	0.001658	0.1921	0.3977	0.8113
**orz[1,6,8]**	0.1442	0.0593	6.76E-04	0.06088	0.1338	0.2889
**orz[1,7,8]**	0.3745	0.1882	0.001956	0.1364	0.3362	0.8375
**sd**	0.2694	0.1708	0.003203	0.01371	0.2561	0.6327

or: fixed points orz: semi-fixed points

e.gender

	mean	sd	MC_error	val2.5pc	median	val97.5pc
**B**	-12.54	10.77	0.3495	-33.26	-8.802	1.822
**SUCRA[1]**	0.8475	0.3124	0.01001	0	1	1
**SUCRA[2]**	0.4688	0.07754	0.001726	0.4286	0.4286	0.7143
**SUCRA[3]**	0.1733	0.09208	0.001542	0	0.1429	0.2857
**SUCRA[4]**	0.2574	0.1047	0.001729	0	0.2857	0.4286
**SUCRA[5]**	0.04337	0.08379	0.001298	0	0	0.2857
**SUCRA[6]**	0.5748	0.07572	0.001734	0.4286	0.5714	0.7143
**SUCRA[7]**	0.7437	0.06053	0.001821	0.7143	0.7143	0.8571
**SUCRA[8]**	0.891	0.06314	0.00194	0.8571	0.8571	1
**SUCRAz[1]**	0.1746	0.1261	0.001006	0	0.1429	0.4286
**SUCRAz[2]**	0.5894	0.05682	5.47E-04	0.5714	0.5714	0.7143
**SUCRAz[3]**	0.2578	0.1117	0.001043	0	0.2857	0.4286
**SUCRAz[4]**	0.3591	0.1297	0.001456	0	0.4286	0.4286
**SUCRAz[5]**	0.06943	0.1171	0.001119	0	0	0.4286
**SUCRAz[6]**	0.6934	0.05305	5.09E-04	0.5714	0.7143	0.7143
**SUCRAz[7]**	0.8576	0.01829	1.05E-04	0.8571	0.8571	0.8571
**SUCRAz[8]**	0.9986	0.01408	9.64E-05	1	1	1
**or[1,2]**	5.54E+12	3.14E+13	9.52E+11	0.04145	1591	6.75E+13
**or[1,3]**	1.91E+13	1.08E+14	3.26E+12	0.1449	5374	2.40E+14
**or[1,4]**	1.57E+13	9.82E+13	2.73E+12	0.09924	4259	1.71E+14
**or[1,5]**	3.27E+13	1.97E+14	5.50E+12	0.2138	9288	3.60E+14
**or[1,6]**	4.42E+12	2.55E+13	7.61E+11	0.0325	1220	5.32E+13
**or[1,7]**	1.82E+12	1.09E+13	3.14E+11	0.01276	488.1	2.11E+13
**or[1,8]**	6.12E+11	3.60E+12	1.06E+11	0.0042	169.5	7.25E+12
**or[2,3]**	3.532	0.7362	0.008387	2.312	3.458	5.197
**or[2,4]**	2.786	1.412	0.01813	1.008	2.488	6.283
**or[2,5]**	5.983	2.665	0.03439	2.35	5.455	12.62
**or[2,6]**	0.8015	0.2029	0.0024	0.4775	0.781	1.246
**or[2,7]**	0.3297	0.1144	0.001279	0.1565	0.3127	0.5968
**or[2,8]**	0.1095	0.05039	4.42E-04	0.0571	0.1045	0.1894
**or[3,4]**	0.8133	0.4292	0.005553	0.2818	0.7209	1.885
**or[3,5]**	1.726	0.7557	0.009537	0.6812	1.59	3.577
**or[3,6]**	0.236	0.8484	0.001762	0.1267	0.226	0.3941
**or[3,7]**	0.09551	0.09196	4.38E-04	0.04512	0.09058	0.1719
**or[3,8]**	0.0335	0.617	0.00117	0.01468	0.03031	0.06186
**or[4,5]**	2.602	1.662	0.01973	0.6807	2.199	6.867
**or[4,6]**	0.3411	0.5388	0.001955	0.1354	0.3138	0.6979
**or[4,7]**	0.141	0.09074	8.26E-04	0.04629	0.1257	0.324
**or[4,8]**	0.04944	0.3795	7.77E-04	0.01386	0.04216	0.1222
**or[5,6]**	0.1719	8.49	0.01555	0.05987	0.1435	0.3315
**or[5,7]**	0.0663	0.8639	0.001639	0.02116	0.05685	0.1551

**or[5,8]**	0.03322	6.19	0.01132	0.006954	0.01921	0.05293
**or[6,7]**	0.4271	0.1601	0.001702	0.1954	0.4	0.8144
**or[6,8]**	0.1449	0.05885	7.23E-04	0.06273	0.1345	0.2869
**or[7,8]**	0.3726	0.1828	0.002078	0.1391	0.3354	0.8257
**orz[1,1,2]**	0.2551	0.06534	3.09E-04	0.1512	0.2484	0.3967
**orz[1,1,3]**	0.8991	0.289	0.002354	0.4593	0.8577	1.578
**orz[1,1,4]**	0.7066	0.3985	0.004629	0.2251	0.6167	1.705
**orz[1,1,5]**	1.521	0.7801	0.008931	0.5107	1.358	3.477
**orz[1,1,6]**	0.2031	0.07266	6.06E-04	0.1002	0.1936	0.3595
**orz[1,1,7]**	0.08396	0.03875	3.35E-04	0.03339	0.07753	0.1713
**orz[1,1,8]**	0.02802	0.03959	1.45E-04	0.01191	0.02596	0.05513
**orz[1,2,3]**	3.532	0.7362	0.008387	2.312	3.458	5.197
**orz[1,2,4]**	2.786	1.412	0.01813	1.008	2.488	6.283
**orz[1,2,5]**	5.983	2.665	0.03439	2.35	5.455	12.62
**orz[1,2,6]**	0.8015	0.2029	0.0024	0.4775	0.781	1.246
**orz[1,2,7]**	0.3297	0.1144	0.001279	0.1565	0.3127	0.5968
**orz[1,2,8]**	0.1095	0.05039	4.42E-04	0.0571	0.1045	0.1894
**orz[1,3,4]**	0.8133	0.4292	0.005553	0.2818	0.7209	1.885
**orz[1,3,5]**	1.726	0.7557	0.009537	0.6812	1.59	3.577
**orz[1,3,6]**	0.236	0.8484	0.001762	0.1267	0.226	0.3941
**orz[1,3,7]**	0.09551	0.09196	4.38E-04	0.04512	0.09058	0.1719
**orz[1,3,8]**	0.0335	0.617	0.00117	0.01468	0.03031	0.06186
**orz[1,4,5]**	2.602	1.662	0.01973	0.6807	2.199	6.867
**orz[1,4,6]**	0.3411	0.5388	0.001955	0.1354	0.3138	0.6979
**orz[1,4,7]**	0.141	0.09074	8.26E-04	0.04629	0.1257	0.324
**orz[1,4,8]**	0.04944	0.3795	7.77E-04	0.01386	0.04216	0.1222
**orz[1,5,6]**	0.1719	8.49	0.01555	0.05987	0.1435	0.3315
**orz[1,5,7]**	0.0663	0.8639	0.001639	0.02116	0.05685	0.1551
**orz[1,5,8]**	0.03322	6.19	0.01132	0.006954	0.01921	0.05293
**orz[1,6,7]**	0.4271	0.1601	0.001702	0.1954	0.4	0.8144
**orz[1,6,8]**	0.1449	0.05885	7.23E-04	0.06273	0.1345	0.2869
**orz[1,7,8]**	0.3726	0.1828	0.002078	0.1391	0.3354	0.8257
**sd**	0.2533	0.1645	0.003102	0.0134	0.2381	0.6073
**totresdev**	91.28	12.58	0.07884	69.31	90.89	115.4

Gender ratio=(n[female] x 2+ n[male] x1)/n[total]

or: Gender ratio≤1.5   orz: Gender ratio>1.5

4.3 FS-14 total score of CFS patients

A.Network map

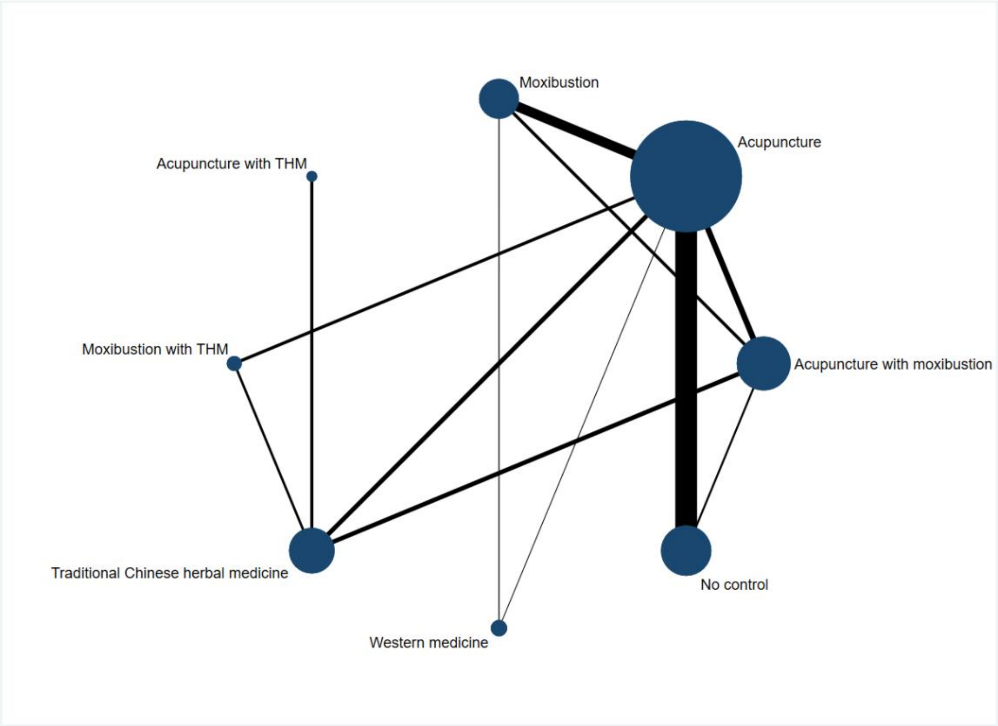

B.Contribution plot

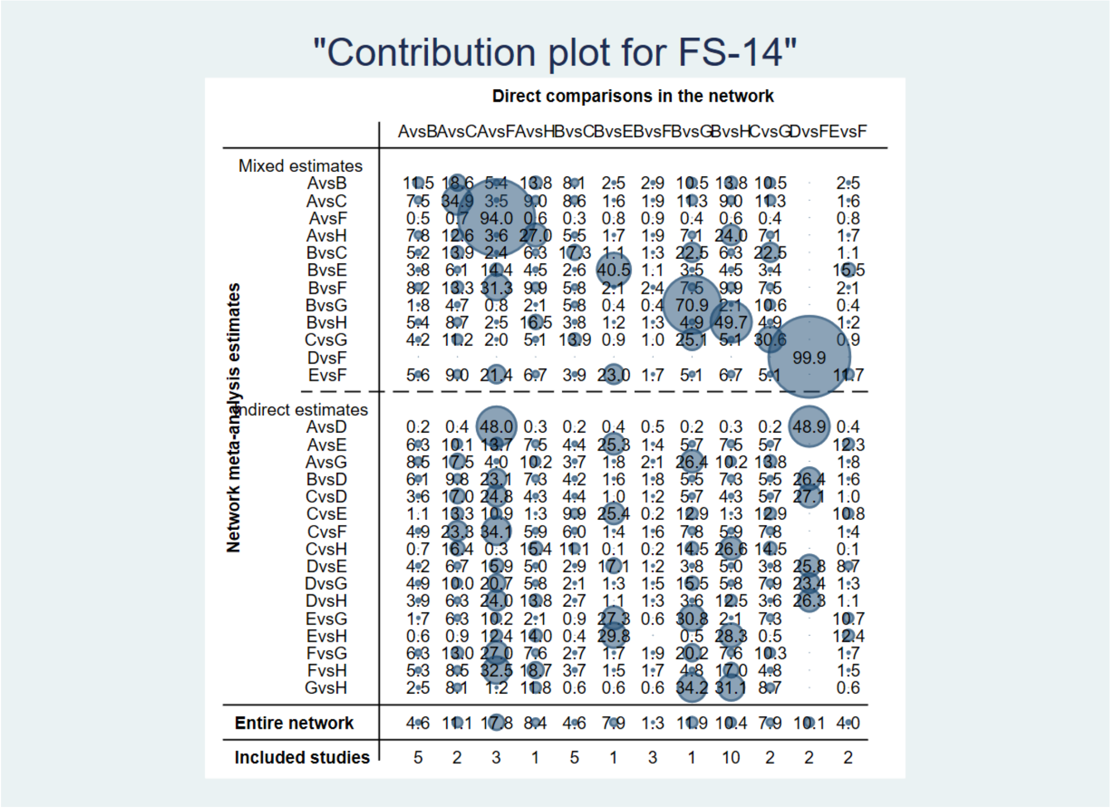

A- Acupuncture with moxibustion; B- Acupuncture; C- Moxibustion; D- Acupuncture with THM; E- Moxibustion with THM; F- Traditional Chinses herbal medicine(THM) G- Western medicine; H- No control

C.Convergence diagraph

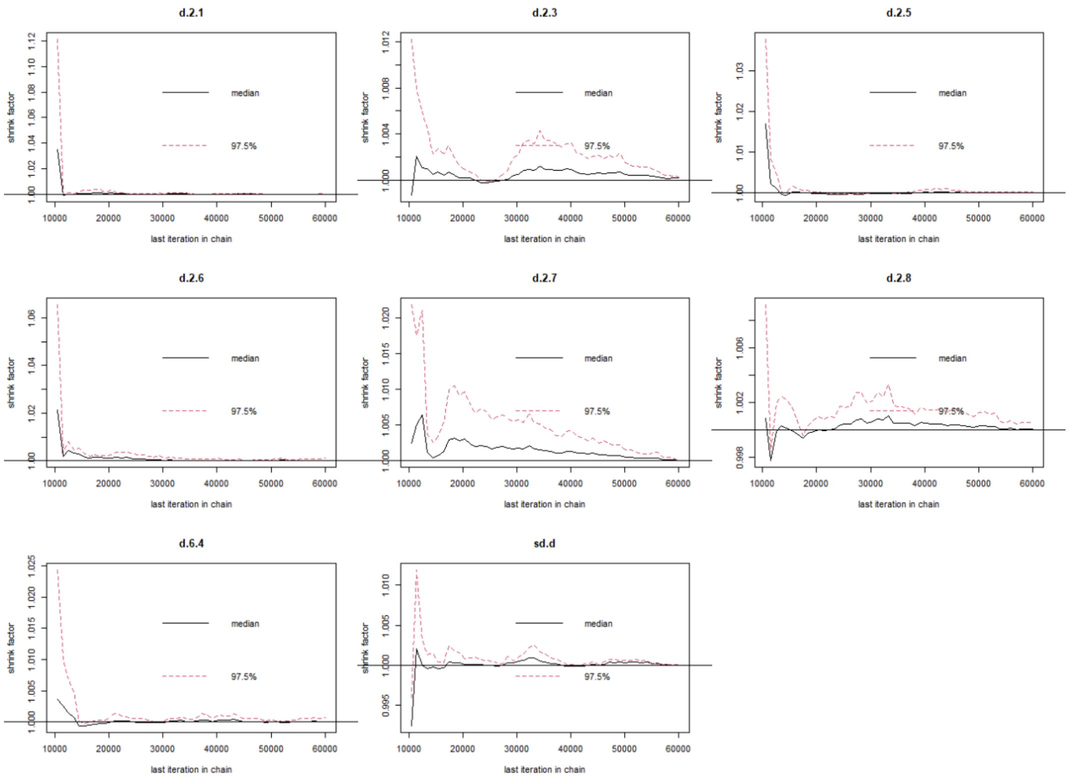

Potential scale reduction factors:

	Point est.	Upper C.I.
**d.2.1**	1	1
**d.2.3**	1	1
**d.2.5**	1	1
**d.2.6**	1	1
**d.2.7**	1	1
**d.2.8**	1	1
**d.6.4**	1	1
**sd.d**	1	1

1- Acupuncture with moxibustion; 2- Acupuncture; 3- Moxibustion; 4- Acupuncture with THM; 5- Moxibustion with THM; 6- Traditional Chinses herbal medicine(THM) 7- Western medicine; 8- No control

D.Trajectories and density diagram

Results on the Log Odds Ratio scale
Iterations = 10010:60000
Thinning interval = 10
Number of chains = 4
Sample size per chain = 5000

1. **Empirical mean and standard deviation for each variable,plus standard error of the mean:**

	Mean	SD	NaiveSE	Time-seriesSE
**d.2.1**	2.1982	0.5710	0.004038	0.004071
**d.2.3**	1.4150	0.6139	0.004341	0.004341
**d.2.5**	1.5191	1.0460	0.007396	0.007273
**d.2.6**	-0.8948	0.6969	0.004928	0.005005
**d.2.7**	-1.7984	0.9720	0.006873	0.006941
**d.2.8**	-2.2637	0.5062	0.003579	0.003458
**d.6.4**	1.4716	1.0965	0.007753	0.007632
**sd.d**	1.4713	0.2515	0.001779	0.001815

2. **Quantiles for each variable:**

	2.5%	25%	50%	75%	97.5%
**d.2.1**	1.0576	1.8269	2.2013	2.5741	3.3223
**d.2.3**	0.2008	1.0155	1.4152	1.8106	2.6369
**d.2.5**	-0.5408	0.8280	1.5180	2.1994	3.6033
**d.2.6**	-2.2805	-1.3491	-0.8959	-0.4428	0.4921
**d.2.7**	-3.7187	-2.4343	-1.8050	-1.1640	0.1164
**d.2.8**	-3.2670	-2.5937	-2.2598	-1.9270	-1.2856
**d.6.4**	-0.7006	0.7556	1.4717	-2.1788	3.6355
**sd.d**	1.0649	1.2929	1.4401	1.6172	2.0552

3. **Model fit (residual deviance):**
   Dbar: 68.54744   pD: 61.98776   DIC:130.53520
   65 data points, ratio 1.055, I²= 7%

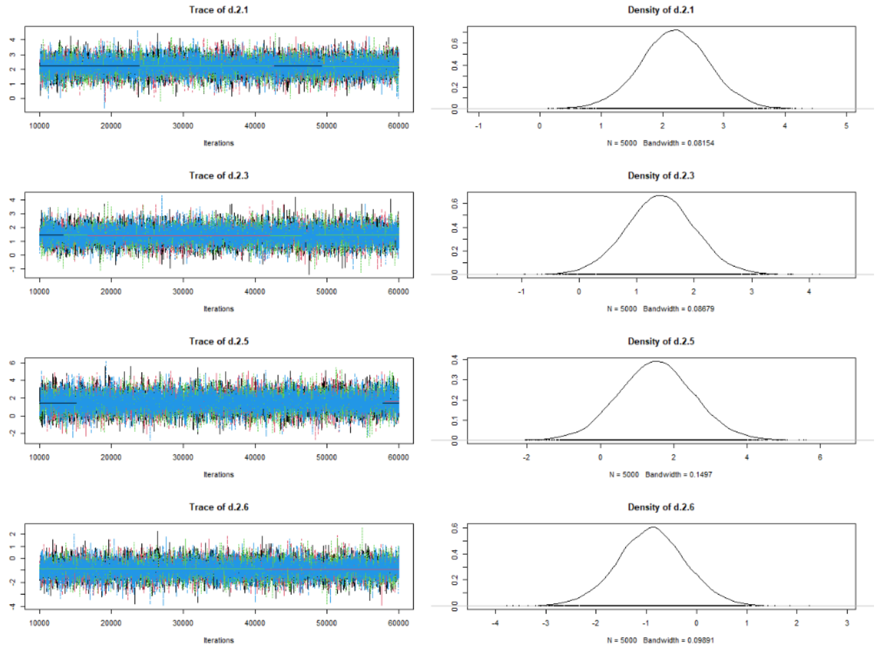

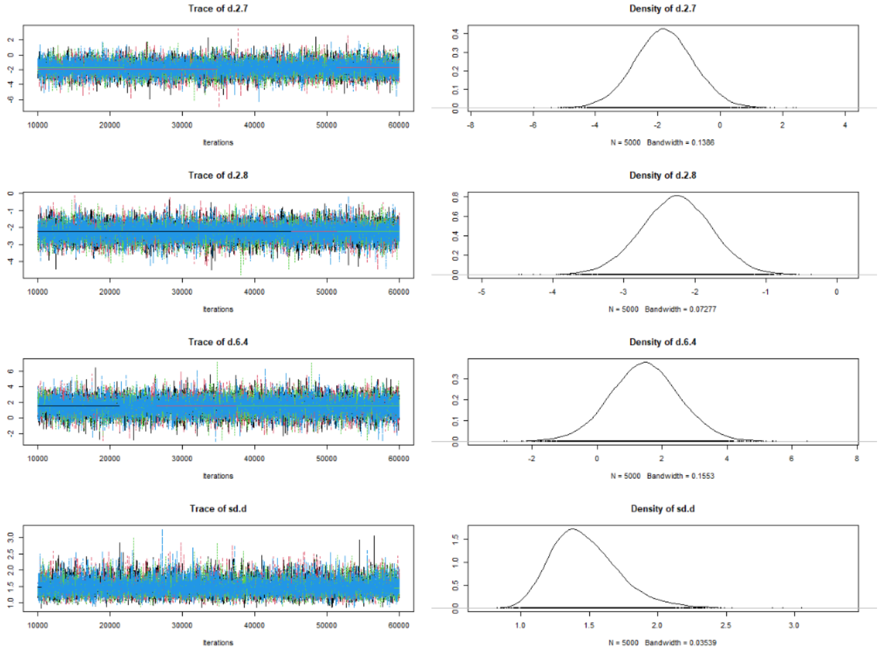

E. Forest plot

Compared with No Control group

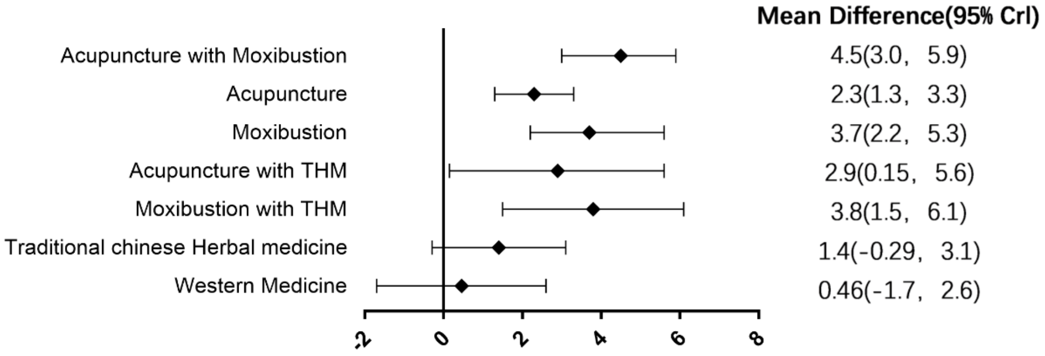

F．League table

**Acupuncture with moxibustion**					
**9.04 (2.88, 27.73)**	**Acupuncture**				
2.2 (0.5, 9.5)	0.24 (0.07, 0.82)	**Moxibustion**			
5 (0.38, 65.46)	0.56 (0.04, 7.35)	2.3 (0.14, 38.67)	**Acupuncture with THM**		
1.98 (0.22, 17.3)	0.22 (0.03, 1.72)	0.9 (0.09, 9.59)	0.39 (0.02, 7.03)	**Moxibustion with THM**	
**22.08 (5.28,**	2.45 (0.61,	**9.96 (1.76,**	4.36 (0.5,	**11 (1.77,**	**Traditional**

**91.55)**	9.78)	**59.3)**	37.92)	**71.79)**	**Chinses herbal medicine**		
**54.61 (6.33, 445.72)**	6.08 (0.89, 41.21)	**24.88 (4.12, 151.54)**	10.91 (0.43, 256.78)	**27.79 (1.61, 456.6)**	2.49 (0.24, 24.35)	**Western medicine**	
**86.56 (20.2, 364.67)**	**9.58 (3.62, 26.23)**	**39.42 (8.57, 189.2)**	**16.96 (1.11, 265.34)**	**43.5 (4.61, 441.68)**	3.91 (0.76, 21.41)	1.57 (0.19, 13.94)	**No control**

G. Inconsistency and Heterogeneity

a. Loops of Statistical Inconsistency:

Loop	IF	seIF	z_value	p_value	CI_95	Loop_Heterog_tau2
AM-Acu-Mox	2.065	1.619	1.279	0.202	(0.00,5.24)	2.523
AM-Acu-NC	2.054	2.096	0.980	0.327	(0.00,6.16)	2.309
AM-Acu-THM	1.077	2.110	0.510	0.610	(0.00,5.21)	4.659
Acu-MT-THM	0.433	3.406	0.127	0.899	(0.00,7.11)	5.810
Acu-Mox-WM	0.304	1.335	0.228	0.820	(0.00,2.92)	0.694

b. Side-splitting

**Side**	**Direct**		**Indirect**		**Difference**		**P>|z|**	**tau**
	Coef.	Std. Err.	Coef.	Std. Err.	Coef.	Std. Err.		
**A B**	-2.305951	.65426	-1.97746	.9845388	-.3284915	1.18225	0.781	1.418279
**A C**	-2.126021	.993292	.2812497	.8944823	-2.407271	1.336896	0.072	1.329197
**A F**	-2.357591	.8187938	-4.386167	1.079052	2.028576	1.354578	0.134	1.344323
**A H**	-2.667148	1.482368	-4.928846	.7640266	2.261697	1.668503	0.175	1.366883
**B C**	1.83649	.6686422	.2622648	1.111815	1.574225	1.297659	0.225	1.380465
**B E**	1.5	1.678761	1.527036	1.250325	-.0270358	2.093216	0.990	1.416971
**B F**	-1.349406	.8468539	-.2186542	1.027484	-1.130752	1.331299	0.396	1.395591
**B G**	-1.77	1.445186	-1.833633	1.189895	.063633	1.872008	0.973	1.423544
**B H***	-2.351019	.4745794	1.534789	2.766528	-3.885808	2.803832	0.166	1.361341
**C G**	-3.24678	1.01286	-3.182878	1.574323	-.0639014	1.872002	0.973	1.423544
**D F***	-1.478334	1.029831	-6.201754	745.4444	4.723421	745.4451	0.995	1.387736
**E F**	-2.417179	1.034031	-2.390207	1.820086	-.0269724	2.093213	0.990	1.416972

*** All the evidence about these contrasts comes from the trials which directly compare them.**

c.Design-by-treatment test

chi2(10) = 9.31

Prob > chi2 = 0.5026

H. Comparison-adjusted funnel plot

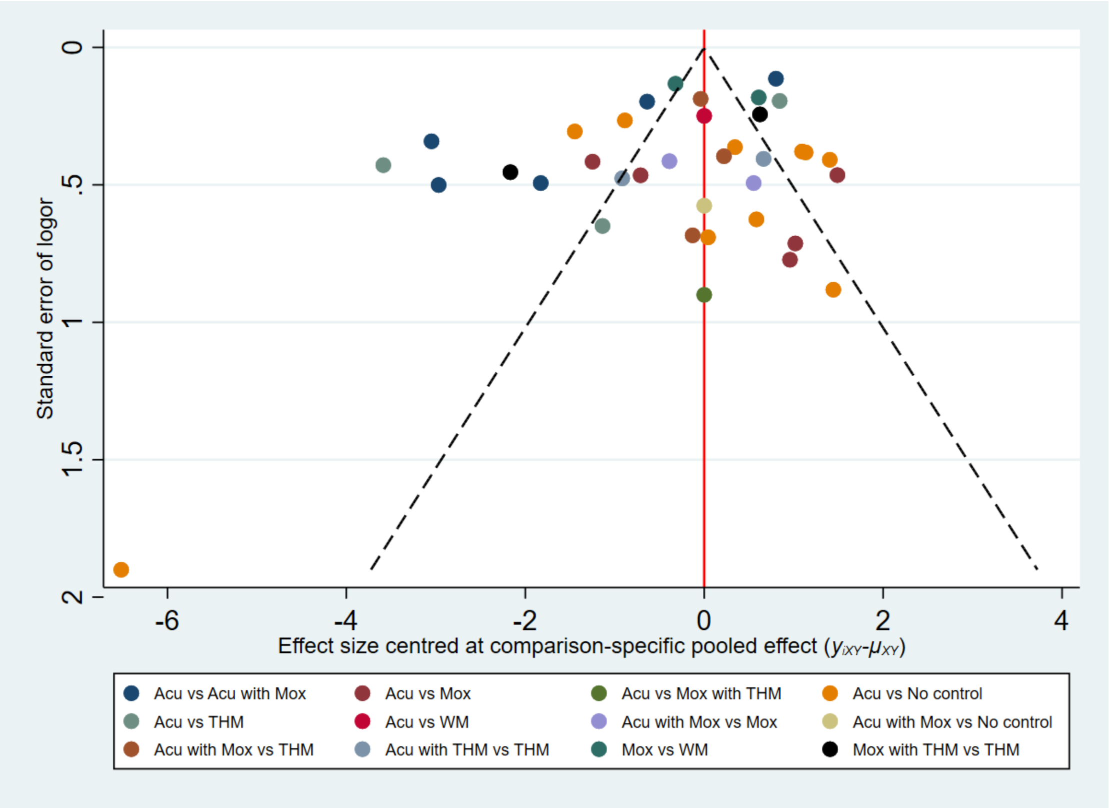

I.SUCRA diagram

a. Ranking probability diagram

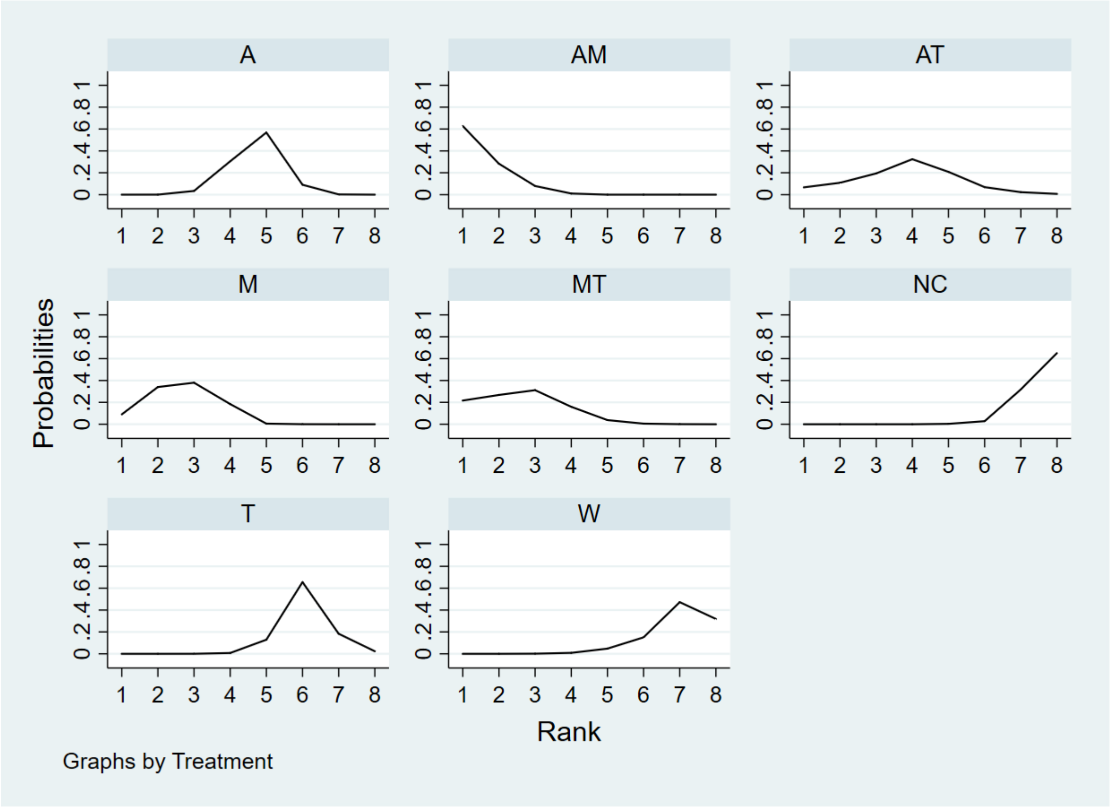

b. **Cumulative probability ranking diagram**

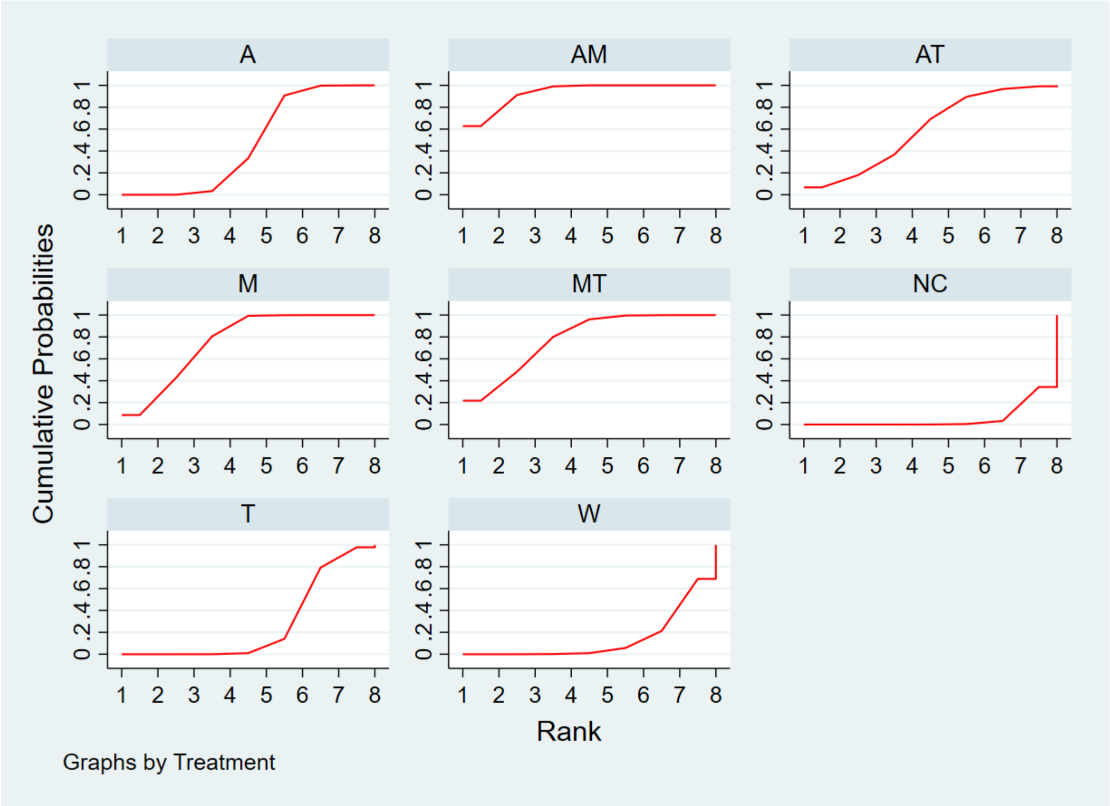

AM-Acupuncture with moxibustion; A-Acupuncture; M-Moxibustion; AT-Acupuncture with THM; MT-Moxibustion with THM; T-Traditional Chinses herbal medicine; W-Western medicine; NC-No control.

J.Subgroup analysis

a. age

	mean	sd	MC_error	val2.5pc	median	val97.5pc
**B**	-1.167	1.191	0.02507	-3.54	-1.163	1.181
**MD[1,2]**	-1.911	0.6481	0.006934	-3.182	-1.915	-0.6184
**MD[1,3]**	-0.3874	0.8442	0.009023	-2.039	-0.3915	1.299
**MD[1,4]**	-1.315	1.356	0.007634	-3.996	-1.314	1.368
**MD[1,5]**	-0.3865	1.15	0.007442	-2.648	-0.3907	1.896
**MD[1,6]**	-2.793	0.7845	0.007178	-4.343	-2.794	-1.237
**MD[1,7]**	-3.65	1.136	0.008533	-5.881	-3.657	-1.385
**MD[1,8]**	-4.194	0.7896	0.006656	-5.763	-4.191	-2.642
**MD[2,3]**	1.524	0.6249	0.002443	0.2937	1.522	2.764
**MD[2,4]**	0.5966	1.307	0.002718	-1.995	0.5992	3.176
**MD[2,5]**	1.525	1.053	0.002283	-0.5592	1.527	3.608
**MD[2,6]**	-0.8821	0.699	0.001606	-2.273	-0.8772	0.491
**MD[2,7]**	-1.739	0.9677	0.002267	-3.652	-1.741	0.1794
**MD[2,8]**	-2.282	0.5101	0.001063	-3.306	-2.276	-1.29
**MD[3,4]**	-0.9272	1.423	0.003515	-3.749	-0.9238	1.879

**MD[3,5]**	9.63E-04	1.204	0.003262	-2.384	0.002439	2.375
**MD[3,6]**	-2.406	0.8976	0.002759	-4.201	-2.402	-0.6418
**MD[3,7]**	-3.263	0.897	0.00151	-5.042	-3.262	-1.494
**MD[3,8]**	-3.806	0.8025	0.003063	-5.411	-3.797	-2.239
**MD[4,5]**	0.9282	1.454	0.002435	-1.935	0.9236	3.808
**MD[4,6]**	-1.479	1.104	0.001836	-3.661	-1.479	0.7025
**MD[4,7]**	-2.336	1.612	0.00352	-5.514	-2.338	0.8516
**MD[4,8]**	-2.879	1.395	0.003042	-5.657	-2.875	-0.1359
**MD[5,6]**	-2.407	0.9455	0.001563	-4.289	-2.405	-0.5464
**MD[5,7]**	-3.264	1.419	0.003213	-6.057	-3.267	-0.4441
**MD[5,8]**	-3.807	1.163	0.00262	-6.127	-3.801	-1.528
**MD[6,7]**	-0.857	1.173	0.00274	-3.167	-0.8621	1.478
**MD[6,8]**	-1.4	0.8522	0.00207	-3.1	-1.397	0.2735
**MD[7,8]**	-0.5433	1.093	0.002805	-2.732	-0.5353	1.599
**MDz[1,1,2]**	-3.078	1.061	0.01867	-5.187	-3.08	-0.971
**MDz[1,1,3]**	-1.555	1.083	0.01656	-3.695	-1.555	0.604
**MDz[1,1,4]**	-2.482	1.586	0.01847	-5.623	-2.481	0.6535
**MDz[1,1,5]**	-1.554	1.417	0.01857	-4.361	-1.551	1.247
**MDz[1,1,6]**	-3.961	1.139	0.01841	-6.227	-3.958	-1.71
**MDz[1,1,7]**	-4.818	1.355	0.01736	-7.497	-4.819	-2.124
**MDz[1,1,8]**	-5.361	1.173	0.01914	-7.703	-5.354	-3.055
**MDz[1,2,3]**	1.524	0.6249	0.002443	0.2937	1.522	2.764
**MDz[1,2,4]**	0.5966	1.307	0.002718	-1.995	0.5992	3.176
**MDz[1,2,5]**	1.525	1.053	0.002283	-0.5592	1.527	3.608
**MDz[1,2,6]**	-0.8821	0.699	0.001606	-2.273	-0.8772	0.491
**MDz[1,2,7]**	-1.739	0.9677	0.002267	-3.652	-1.741	0.1794
**MDz[1,2,8]**	-2.282	0.5101	0.001063	-3.306	-2.276	-1.29
**MDz[1,3,4]**	-0.9272	1.423	0.003515	-3.749	-0.9238	1.879
**MDz[1,3,5]**	9.63E-04	1.204	0.003262	-2.384	0.002439	2.375
**MDz[1,3,6]**	-2.406	0.8976	0.002759	-4.201	-2.402	-0.6418
**MDz[1,3,7]**	-3.263	0.897	0.00151	-5.042	-3.262	-1.494
**MDz[1,3,8]**	-3.806	0.8025	0.003063	-5.411	-3.797	-2.239
**MDz[1,4,5]**	0.9282	1.454	0.002435	-1.935	0.9236	3.808
**MDz[1,4,6]**	-1.479	1.104	0.001836	-3.661	-1.479	0.7025
**MDz[1,4,7]**	-2.336	1.612	0.00352	-5.514	-2.338	0.8516
**MDz[1,4,8]**	-2.879	1.395	0.003042	-5.657	-2.875	-0.1359
**MDz[1,5,6]**	-2.407	0.9455	0.001563	-4.289	-2.405	-0.5464
**MDz[1,5,7]**	-3.264	1.419	0.003213	-6.057	-3.267	-0.4441
**MDz[1,5,8]**	-3.807	1.163	0.00262	-6.127	-3.801	-1.528
**MDz[1,6,7]**	-0.857	1.173	0.00274	-3.167	-0.8621	1.478
**MDz[1,6,8]**	-1.4	0.8522	0.00207	-3.1	-1.397	0.2735
**MDz[1,7,8]**	-0.5433	1.093	0.002805	-2.732	-0.5353	1.599
**SUCRA[1]**	0.1206	0.1277	0.001116	0	0.1429	0.4286
**SUCRA[2]**	0.5333	0.1029	2.10E-04	0.2857	0.5714	0.7143

**SUCRA[3]**	0.2072	0.1431	7.49E-04	0	0.1429	0.4286
**SUCRA[4]**	0.4043	0.2187	4.39E-04	0	0.4286	0.8571
**SUCRA[5]**	0.212	0.1745	4.15E-04	0	0.1429	0.5714
**SUCRA[6]**	0.7252	0.101	1.92E-04	0.5714	0.7143	1
**SUCRA[7]**	0.851	0.1363	3.20E-04	0.5714	0.8571	1
**SUCRA[8]**	0.9464	0.08167	1.77E-04	0.7143	1	1
**SUCRAz[1]**	0.03786	0.0885	0.001098	0	0	0.2857
**SUCRAz[2]**	0.5333	0.1029	2.28E-04	0.2857	0.5714	0.7143
**SUCRAz[3]**	0.2418	0.1188	3.46E-04	0	0.2857	0.4286
**SUCRAz[4]**	0.4191	0.2007	4.59E-04	0	0.4286	0.8571
**SUCRAz[5]**	0.2452	0.1516	5.83E-04	0	0.2857	0.5714
**SUCRAz[6]**	0.7251	0.101	1.94E-04	0.5714	0.7143	1
**SUCRAz[7]**	0.8512	0.1357	3.14E-04	0.5714	0.8571	1
**SUCRAz[8]**	0.9464	0.08169	1.78E-04	0.7143	1	1
**sd**	1.478	0.2588	0.001345	1.063	1.446	2.073
**totresdev**	68.36	11.55	0.02525	47.55	67.72	92.7

or: Average age≤40 years **orz: Average age>40 years**

b. course of disease

	**mean**	**sd**	**MC_error**	**val2.5pc**	**median**	**val97.5pc**
**B**	-0.3858	1.087	0.02681	-2.48	-0.4104	1.829
**MD[1,2]**	-2.008	0.7975	0.01373	-3.595	-2.006	-0.434
**MD[1,3]**	-0.6369	0.8588	0.01054	-2.328	-0.6374	1.069
**MD[1,4]**	-1.403	1.472	0.01541	-4.308	-1.402	1.502
**MD[1,5]**	-0.4763	1.268	0.01501	-2.987	-0.4754	2.03
**MD[1,6]**	-2.882	0.9483	0.01519	-4.768	-2.879	-1.014
**MD[1,7]**	-3.849	1.186	0.01177	-6.189	-3.849	-1.494
**MD[1,8]**	-4.287	0.9054	0.01295	-6.092	-4.281	-2.511
**MD[2,3]**	1.371	0.6402	0.00347	0.1097	1.369	2.641
**MD[2,4]**	0.6048	1.333	0.003202	-2.026	0.6067	3.238
**MD[2,5]**	1.532	1.071	0.002567	-0.593	1.532	3.644
**MD[2,6]**	-0.8738	0.7129	0.00218	-2.287	-0.8719	0.5299
**MD[2,7]**	-1.841	0.9887	0.002752	-3.801	-1.843	0.1227
**MD[2,8]**	-2.279	0.5193	0.00127	-3.321	-2.274	-1.269
**MD[3,4]**	-0.7662	1.458	0.005702	-3.651	-0.765	2.116
**MD[3,5]**	0.1605	1.23	0.005159	-2.278	0.1632	2.587
**MD[3,6]**	-2.245	0.9278	0.005158	-4.086	-2.24	-0.4254
**MD[3,7]**	-3.212	0.9141	0.001743	-5.026	-3.212	-1.399
**MD[3,8]**	-3.65	0.8125	0.002876	-5.281	-3.642	-2.065
**MD[4,5]**	0.9268	1.48	0.002659	-1.989	0.9241	3.859
**MD[4,6]**	-1.479	1.125	0.001933	-3.709	-1.478	0.742
**MD[4,7]**	-2.446	1.646	0.004998	-5.699	-2.448	0.8181
**MD[4,8]**	-2.884	1.423	0.003905	-5.712	-2.88	-0.09264
**MD[5,6]**	-2.405	0.9615	0.001737	-4.307	-2.405	-0.5066

**MD[5,7]**	-3.373	1.445	0.004427	-6.231	-3.372	-0.5063
**MD[5,8]**	-3.811	1.185	0.003278	-6.165	-3.805	-1.481
**MD[6,7]**	-0.9677	1.203	0.004383	-3.339	-0.9697	1.42
**MD[6,8]**	-1.405	0.8715	0.003089	-3.14	-1.402	0.3025
**MD[7,8]**	-0.4377	1.111	0.002364	-2.661	-0.4309	1.741
**MDz[1,1,2]**	-2.394	0.799	0.01344	-3.959	-2.401	-0.7854
**MDz[1,1,3]**	-1.023	1.013	0.0167	-3.003	-1.031	1.01
**MDz[1,1,4]**	-1.789	1.429	0.01223	-4.611	-1.792	1.047
**MDz[1,1,5]**	-0.8621	1.231	0.01252	-3.29	-0.8639	1.579
**MDz[1,1,6]**	-3.267	0.8796	0.01201	-4.997	-3.271	-1.518
**MDz[1,1,7]**	-4.235	1.266	0.01568	-6.731	-4.239	-1.708
**MDz[1,1,8]**	-4.673	0.948	0.01434	-6.548	-4.674	-2.794
**MDz[1,2,3]**	1.371	0.6402	0.00347	0.1097	1.369	2.641
**MDz[1,2,4]**	0.6048	1.333	0.003202	-2.026	0.6067	3.238
**MDz[1,2,5]**	1.532	1.071	0.002567	-0.593	1.532	3.644
**MDz[1,2,6]**	-0.8738	0.7129	0.00218	-2.287	-0.8719	0.5299
**MDz[1,2,7]**	-1.841	0.9887	0.002752	-3.801	-1.843	0.1227
**MDz[1,2,8]**	-2.279	0.5193	0.00127	-3.321	-2.274	-1.269
**MDz[1,3,4]**	-0.7662	1.458	0.005702	-3.651	-0.765	2.116
**MDz[1,3,5]**	0.1605	1.23	0.005159	-2.278	0.1632	2.587
**MDz[1,3,6]**	-2.245	0.9278	0.005158	-4.086	-2.24	-0.4254
**MDz[1,3,7]**	-3.212	0.9141	0.001743	-5.026	-3.212	-1.399
**MDz[1,3,8]**	-3.65	0.8125	0.002876	-5.281	-3.642	-2.065
**MDz[1,4,5]**	0.9268	1.48	0.002659	-1.989	0.9241	3.859
**MDz[1,4,6]**	-1.479	1.125	0.001933	-3.709	-1.478	0.742
**MDz[1,4,7]**	-2.446	1.646	0.004998	-5.699	-2.448	0.8181
**MDz[1,4,8]**	-2.884	1.423	0.003905	-5.712	-2.88	-0.09264
**MDz[1,5,6]**	-2.405	0.9615	0.001737	-4.307	-2.405	-0.5066
**MDz[1,5,7]**	-3.373	1.445	0.004427	-6.231	-3.372	-0.5063
**MDz[1,5,8]**	-3.811	1.185	0.003278	-6.165	-3.805	-1.481
**MDz[1,6,7]**	-0.9677	1.203	0.004383	-3.339	-0.9697	1.42
**MDz[1,6,8]**	-1.405	0.8715	0.003089	-3.14	-1.402	0.3025
**MDz[1,7,8]**	-0.4377	1.111	0.002364	-2.661	-0.4309	1.741
**SUCRA[1]**	0.1066	0.1291	0.001599	0	0	0.4286
**SUCRA[2]**	0.5313	0.1055	2.37E-04	0.2857	0.5714	0.7143
**SUCRA[3]**	0.2362	0.1395	3.28E-04	0	0.2857	0.4286
**SUCRA[4]**	0.3974	0.2227	7.71E-04	0	0.4286	0.8571
**SUCRA[5]**	0.2072	0.174	8.90E-04	0	0.1429	0.5714
**SUCRA[6]**	0.72	0.103	3.21E-04	0.5714	0.7143	1
**SUCRA[7]**	0.861	0.1345	3.68E-04	0.5714	0.8571	1
**SUCRA[8]**	0.9403	0.08427	1.51E-04	0.7143	1	1
**SUCRAz[1]**	0.07005	0.1089	0.001371	0	0	0.2857
**SUCRAz[2]**	0.532	0.1047	2.38E-04	0.2857	0.5714	0.7143
**SUCRAz[3]**	0.2466	0.1361	0.001027	0	0.2857	0.4286

**SUCRAz[4]**	0.4063	0.2128	4.27E-04	0	0.4286	0.8571
**SUCRAz[5]**	0.2235	0.1638	3.96E-04	0	0.1429	0.5714
**SUCRAz[6]**	0.7202	0.1025	3.04E-04	0.5714	0.7143	1
**SUCRAz[7]**	0.861	0.1344	3.79E-04	0.5714	0.8571	1
**SUCRAz[8]**	0.9403	0.08428	1.51E-04	0.7143	1	1
**sd**	1.505	0.2631	0.001504	1.081	1.474	2.106
**totresdev**	68.49	11.6	0.02511	47.6	67.84	92.98

or: Average course of disease≤1 year orz: Average course of disease>1 year

c. Duration of treatment

	**mean**	**sd**	**MC_error**	**val2.5pc**	**median**	**val97.5pc**
**B**	11.88	30.43	0.8308	-32.55	2.596	61.34
**MD[1,2]**	-14.41	30.43	0.8307	-63.93	-5.129	30.11
**MD[1,3]**	-12.92	30.44	0.8307	-62.48	-3.645	31.64
**MD[1,4]**	-13.07	30.45	0.8306	-62.79	-3.863	31.72
**MD[1,5]**	-12.14	30.45	0.8305	-61.86	-2.921	32.66
**MD[1,6]**	-14.55	30.44	0.8307	-64.12	-5.294	30.07
**MD[1,7]**	-16.17	30.44	0.8306	-65.82	-6.925	28.5
**MD[1,8]**	-16.18	30.44	0.8307	-65.74	-6.917	28.41
**MD[2,3]**	1.491	0.5819	9.68E-04	0.382	1.49	2.607
**MD[2,4]**	1.336	1.333	0.003067	-1.262	1.341	3.912
**MD[2,5]**	2.267	1.379	0.003125	-0.2931	2.268	4.826
**MD[2,6]**	-0.1407	0.8493	0.002111	-1.807	-0.1372	1.504
**MD[2,7]**	-1.759	0.8793	0.001383	-3.487	-1.76	-0.02611
**MD[2,8]**	-1.769	0.5684	8.22E-04	-2.89	-1.771	-0.6431
**MD[3,4]**	-0.155	1.447	0.003329	-2.934	-0.1496	2.595
**MD[3,5]**	0.7757	1.499	0.003404	-1.97	0.7786	3.511
**MD[3,6]**	-1.632	1.01	0.002437	-3.567	-1.628	0.2737
**MD[3,7]**	-3.25	0.8255	0.00117	-4.861	-3.25	-1.646
**MD[3,8]**	-3.261	0.8081	0.001372	-4.822	-3.261	-1.7
**MD[4,5]**	0.9307	1.431	0.002151	-1.856	0.9313	3.725
**MD[4,6]**	-1.477	1.013	0.00155	-3.468	-1.475	0.512
**MD[4,7]**	-3.095	1.583	0.003428	-6.158	-3.101	-0.00696
**MD[4,8]**	-3.106	1.436	0.003248	-5.883	-3.11	-0.2996
**MD[5,6]**	-2.408	1.046	0.001638	-4.373	-2.406	-0.4678
**MD[5,7]**	-4.026	1.627	0.003511	-7.071	-4.028	-0.9657
**MD[5,8]**	-4.036	1.477	0.003285	-6.812	-4.038	-1.253
**MD[6,7]**	-1.618	1.201	0.002623	-3.948	-1.624	0.7424
**MD[6,8]**	-1.629	1.004	0.002343	-3.585	-1.634	0.3581
**MD[7,8]**	-0.01046	1.042	0.001663	-2.063	-0.01063	2.04
**MDz[1,1,2]**	-2.529	0.5765	0.001794	-3.604	-2.532	-1.442
**MDz[1,1,3]**	-1.038	0.7317	0.002063	-2.385	-1.044	0.3296
**MDz[1,1,4]**	-1.193	1.338	0.003392	-3.783	-1.192	1.387
**MDz[1,1,5]**	-0.2623	1.364	0.003362	-2.815	-0.2631	2.301

**MDz[1,1,6]**	-2.67	0.8579	0.002494	-4.331	-2.669	-1.022
**MDz[1,1,7]**	-4.288	1.005	0.002449	-6.224	-4.291	-2.337
**MDz[1,1,8]**	-4.299	0.77	0.002049	-5.77	-4.303	-2.809
**MDz[1,2,3]**	1.491	0.5819	9.68E-04	0.382	1.49	2.607
**MDz[1,2,4]**	1.336	1.333	0.003067	-1.262	1.341	3.912
**MDz[1,2,5]**	2.267	1.379	0.003125	-0.2931	2.268	4.826
**MDz[1,2,6]**	-0.1407	0.8493	0.002111	-1.807	-0.1372	1.504
**MDz[1,2,7]**	-1.759	0.8793	0.001383	-3.487	-1.76	-0.02611
**MDz[1,2,8]**	-1.769	0.5684	8.22E-04	-2.89	-1.771	-0.6431
**MDz[1,3,4]**	-0.155	1.447	0.003329	-2.934	-0.1496	2.595
**MDz[1,3,5]**	0.7757	1.499	0.003404	-1.97	0.7786	3.511
**MDz[1,3,6]**	-1.632	1.01	0.002437	-3.567	-1.628	0.2737
**MDz[1,3,7]**	-3.25	0.8255	0.00117	-4.861	-3.25	-1.646
**MDz[1,3,8]**	-3.261	0.8081	0.001372	-4.822	-3.261	-1.7
**MDz[1,4,5]**	0.9307	1.431	0.002151	-1.856	0.9313	3.725
**MDz[1,4,6]**	-1.477	1.013	0.00155	-3.468	-1.475	0.512
**MDz[1,4,7]**	-3.095	1.583	0.003428	-6.158	-3.101	-0.00696
**MDz[1,4,8]**	-3.106	1.436	0.003248	-5.883	-3.11	-0.2996
**MDz[1,5,6]**	-2.408	1.046	0.001638	-4.373	-2.406	-0.4678
**MDz[1,5,7]**	-4.026	1.627	0.003511	-7.071	-4.028	-0.9657
**MDz[1,5,8]**	-4.036	1.477	0.003285	-6.812	-4.038	-1.253
**MDz[1,6,7]**	-1.618	1.201	0.002623	-3.948	-1.624	0.7424
**MDz[1,6,8]**	-1.629	1.004	0.002343	-3.585	-1.634	0.3581
**MDz[1,7,8]**	-0.01046	1.042	0.001663	-2.063	-0.01063	2.04
**SUCRA[1]**	0.3825	0.4658	0.01262	0	0	1
**SUCRA[2]**	0.5554	0.1312	0.001881	0.2857	0.5714	0.7143
**SUCRA[3]**	0.2597	0.1404	0.001903	0	0.2857	0.5714
**SUCRA[4]**	0.3069	0.1826	0.00186	0	0.2857	0.7143
**SUCRA[5]**	0.1652	0.1445	0.001848	0	0.1429	0.4286
**SUCRA[6]**	0.6007	0.1385	0.001858	0.2857	0.5714	0.8571
**SUCRA[7]**	0.8595	0.1302	0.001805	0.5714	0.8571	1
**SUCRA[8]**	0.8701	0.1121	0.001812	0.7143	0.8571	1
**SUCRAz[1]**	0.09359	0.1074	2.71E-04	0	0	0.2857
**SUCRAz[2]**	0.6093	0.1108	2.48E-04	0.4286	0.5714	0.7143
**SUCRAz[3]**	0.3089	0.1315	2.92E-04	0	0.2857	0.5714
**SUCRAz[4]**	0.3402	0.1949	3.85E-04	0	0.2857	0.7143
**SUCRAz[5]**	0.167	0.1681	3.25E-04	0	0.1429	0.5714
**SUCRAz[6]**	0.6541	0.119	2.50E-04	0.4286	0.7143	0.8571
**SUCRAz[7]**	0.9082	0.1094	1.83E-04	0.5714	0.8571	1
**SUCRAz[8]**	0.9188	0.08837	1.41E-04	0.7143	0.8571	1
**sd**	1.327	0.2505	5.86E-04	0.9329	1.295	1.904
**totresdev**	69.81	5843	11.21	32.92	50.03	72.16

or: Duration of treatment≤4 weeks orz: Duration of treatment>4 weeks

d. Principle of acupoint selection

	mean	sd	MC_error	val2.5pc	median	val97.5pc
B	0.775	1.196	0.02359	-1.579	0.7619	3.158
MD[1,2]	-2.425	0.6717	0.007172	-3.748	-2.428	-1.092
MD[1,3]	-0.9488	0.7904	0.00563	-2.499	-0.9525	0.6224
MD[1,4]	-1.839	1.373	0.007323	-4.549	-1.84	0.8804
MD[1,5]	-0.9034	1.167	0.007286	-3.202	-0.9052	1.407
MD[1,6]	-3.314	0.7993	0.006995	-4.894	-3.315	-1.735
MD[1,7]	-4.194	1.122	0.006392	-6.398	-4.197	-1.961
MD[1,8]	-4.678	0.8085	0.006863	-6.291	-4.673	-3.093
MD[2,3]	1.476	0.6291	0.002018	0.2376	1.475	2.729
MD[2,4]	0.5866	1.32	0.002638	-2.026	0.5892	3.195
MD[2,5]	1.522	1.063	0.002026	-0.5754	1.523	3.62
MD[2,6]	-0.8893	0.7033	0.001501	-2.283	-0.8883	0.4976
MD[2,7]	-1.769	0.9775	0.001965	-3.706	-1.769	0.171
MD[2,8]	-2.253	0.5143	9.57E-04	-3.288	-2.247	-1.255
MD[3,4]	-0.8898	1.436	0.00324	-3.733	-0.8885	1.948
MD[3,5]	0.0454	1.213	0.002846	-2.363	0.04816	2.442
MD[3,6]	-2.366	0.9028	0.002414	-4.159	-2.364	-0.5853
MD[3,7]	-3.245	0.9071	0.001357	-5.05	-3.244	-1.448
MD[3,8]	-3.729	0.8036	0.001965	-5.338	-3.721	-2.16
MD[4,5]	0.9352	1.469	0.002454	-1.967	0.9352	3.843
MD[4,6]	-1.476	1.116	0.00178	-3.69	-1.475	0.7287
MD[4,7]	-2.355	1.629	0.00323	-5.572	-2.356	0.8755
MD[4,8]	-2.839	1.409	0.002844	-5.646	-2.835	-0.06485
MD[5,6]	-2.411	0.9549	0.001477	-4.306	-2.409	-0.5301
MD[5,7]	-3.29	1.434	0.002816	-6.125	-3.292	-0.4504
MD[5,8]	-3.775	1.175	0.002267	-6.114	-3.769	-1.468
MD[6,7]	-0.8793	1.183	0.002446	-3.215	-0.8811	1.466
MD[6,8]	-1.363	0.8593	0.00183	-3.077	-1.359	0.3242
MD[7,8]	-0.4842	1.099	0.002015	-2.687	-0.4754	1.671
MDz[1,1,2]	-1.65	1.035	0.01685	-3.684	-1.657	0.4117
MDz[1,1,3]	-0.1737	1.203	0.01855	-2.539	-0.1824	2.229
MDz[1,1,4]	-1.064	1.59	0.01732	-4.203	-1.064	2.081
MDz[1,1,5]	-0.1283	1.412	0.01711	-2.911	-0.1316	2.676
MDz[1,1,6]	-2.539	1.13	0.01708	-4.773	-2.54	-0.2947
MDz[1,1,7]	-3.419	1.419	0.01803	-6.212	-3.427	-0.5852
MDz[1,1,8]	-3.903	1.151	0.01731	-6.19	-3.9	-1.627
MDz[1,2,3]	1.476	0.6291	0.002018	0.2376	1.475	2.729
MDz[1,2,4]	0.5866	1.32	0.002638	-2.026	0.5892	3.195
MDz[1,2,5]	1.522	1.063	0.002026	-0.5754	1.523	3.62
MDz[1,2,6]	-0.8893	0.7033	0.001501	-2.283	-0.8883	0.4976
MDz[1,2,7]	-1.769	0.9775	0.001965	-3.706	-1.769	0.171
MDz[1,2,8]	-2.253	0.5143	9.57E-04	-3.288	-2.247	-1.255

**MDz[1,3,4]**	-0.8898	1.436	0.00324	-3.733	-0.8885	1.948
**MDz[1,3,5]**	0.0454	1.213	0.002846	-2.363	0.04816	2.442
**MDz[1,3,6]**	-2.366	0.9028	0.002414	-4.159	-2.364	-0.5853
**MDz[1,3,7]**	-3.245	0.9071	0.001357	-5.05	-3.244	-1.448
**MDz[1,3,8]**	-3.729	0.8036	0.001965	-5.338	-3.721	-2.16
**MDz[1,4,5]**	0.9352	1.469	0.002454	-1.967	0.9352	3.843
**MDz[1,4,6]**	-1.476	1.116	0.00178	-3.69	-1.475	0.7287
**MDz[1,4,7]**	-2.355	1.629	0.00323	-5.572	-2.356	0.8755
**MDz[1,4,8]**	-2.839	1.409	0.002844	-5.646	-2.835	-0.06485
**MDz[1,5,6]**	-2.411	0.9549	0.001477	-4.306	-2.409	-0.5301
**MDz[1,5,7]**	-3.29	1.434	0.002816	-6.125	-3.292	-0.4504
**MDz[1,5,8]**	-3.775	1.175	0.002267	-6.114	-3.769	-1.468
**MDz[1,6,7]**	-0.8793	1.183	0.002446	-3.215	-0.8811	1.466
**MDz[1,6,8]**	-1.363	0.8593	0.00183	-3.077	-1.359	0.3242
**MDz[1,7,8]**	-0.4842	1.099	0.002015	-2.687	-0.4754	1.671
**SUCRA[1]**	0.05898	0.09521	5.91E-04	0	0	0.2857
**SUCRA[2]**	0.5324	0.1028	1.98E-04	0.2857	0.5714	0.7143
**SUCRA[3]**	0.2405	0.1271	2.60E-04	0	0.2857	0.4286
**SUCRA[4]**	0.4146	0.2097	4.09E-04	0	0.4286	0.8571
**SUCRA[5]**	0.2316	0.164	4.32E-04	0	0.2857	0.5714
**SUCRA[6]**	0.7249	0.1022	1.82E-04	0.5714	0.7143	1
**SUCRA[7]**	0.8548	0.1366	2.62E-04	0.5714	0.8571	1
**SUCRA[8]**	0.9422	0.08416	1.47E-04	0.7143	1	1
**SUCRAz[1]**	0.1749	0.1754	0.002494	0	0.1429	0.5714
**SUCRAz[2]**	0.5245	0.1097	3.86E-04	0.2857	0.5714	0.7143
**SUCRAz[3]**	0.1939	0.1448	0.001035	0	0.1429	0.4286
**SUCRAz[4]**	0.3921	0.2263	6.11E-04	0	0.4286	0.8571
**SUCRAz[5]**	0.1961	0.1739	6.67E-04	0	0.1429	0.5714
**SUCRAz[6]**	0.7229	0.1052	2.18E-04	0.5714	0.7143	1
**SUCRAz[7]**	0.8534	0.1402	3.05E-04	0.5714	0.8571	1
**SUCRAz[8]**	0.9421	0.08442	1.49E-04	0.7143	1	1
**sd**	1.494	0.2614	0.001189	1.072	1.463	2.091
**totresdev**	68.52	11.59	0.02262	47.67	67.88	93

or: fixed points   orz: semi-fixed points

e.gender

	**mean**	**sd**	**MC_error**	**val2.5pc**	**median**	**val97.5pc**
**B**	2.742	1.534	0.03951	-0.3032	2.712	5.906
**MD[1,2]**	-4.545	1.417	0.03423	-7.435	-4.528	-1.749
**MD[1,3]**	-3.23	1.534	0.03568	-6.341	-3.213	-0.2061
**MD[1,4]**	-4.138	1.878	0.03688	-7.921	-4.124	-0.454
**MD[1,5]**	-3.162	1.732	0.03616	-6.644	-3.149	0.2504
**MD[1,6]**	-5.617	1.565	0.03677	-8.809	-5.597	-2.539
**MD[1,7]**	-6.425	1.679	0.0352	-9.81	-6.413	-3.111

**MD[1,8]**	-6.818	1.497	0.03474	-9.875	-6.795	-3.886
**MD[2,3]**	1.315	0.583	0.001822	0.1687	1.313	2.473
**MD[2,4]**	0.4069	1.231	0.003639	-2.031	0.4083	2.84
**MD[2,5]**	1.383	0.9931	0.002836	-0.5771	1.382	3.352
**MD[2,6]**	-1.073	0.6619	0.002951	-2.388	-1.071	0.2312
**MD[2,7]**	-1.88	0.9049	0.00188	-3.669	-1.881	-0.08246
**MD[2,8]**	-2.273	0.479	0.001047	-3.24	-2.268	-1.343
**MD[3,4]**	-0.9077	1.333	0.003101	-3.556	-0.9041	1.721
**MD[3,5]**	0.06801	1.127	0.0025	-2.162	0.06875	2.299
**MD[3,6]**	-2.387	0.8373	0.002216	-4.05	-2.383	-0.7433
**MD[3,7]**	-3.195	0.8376	0.001359	-4.855	-3.195	-1.534
**MD[3,8]**	-3.588	0.7473	0.00184	-5.087	-3.581	-2.131
**MD[4,5]**	0.9757	1.367	0.002466	-1.727	0.9708	3.687
**MD[4,6]**	-1.48	1.037	0.001704	-3.534	-1.48	0.5715
**MD[4,7]**	-2.287	1.509	0.003545	-5.268	-2.287	0.7023
**MD[4,8]**	-2.68	1.314	0.003484	-5.296	-2.674	-0.09592
**MD[5,6]**	-2.455	0.8902	0.001644	-4.226	-2.453	-0.7074
**MD[5,7]**	-3.263	1.329	0.002909	-5.891	-3.263	-0.634
**MD[5,8]**	-3.656	1.095	0.002772	-5.844	-3.648	-1.51
**MD[6,7]**	-0.8073	1.095	0.002839	-2.965	-0.8093	1.369
**MD[6,8]**	-1.201	0.8028	0.002779	-2.8	-1.197	0.3744
**MD[7,8]**	-0.3934	1.02	0.002021	-2.437	-0.3847	1.603
**MDz[1,1,2]**	-1.802	0.5805	0.005676	-2.95	-1.803	-0.6515
**MDz[1,1,3]**	-0.4878	0.7151	0.004446	-1.892	-0.49	0.9301
**MDz[1,1,4]**	-1.396	1.245	0.004069	-3.857	-1.395	1.064
**MDz[1,1,5]**	-0.4198	1.051	0.004483	-2.493	-0.422	1.67
**MDz[1,1,6]**	-2.875	0.6879	0.003466	-4.236	-2.874	-1.518
**MDz[1,1,7]**	-3.682	1.022	0.005171	-5.702	-3.684	-1.647
**MDz[1,1,8]**	-4.076	0.7199	0.005364	-5.516	-4.07	-2.672
**MDz[1,2,3]**	1.315	0.583	0.001822	0.1687	1.313	2.473
**MDz[1,2,4]**	0.4069	1.231	0.003639	-2.031	0.4083	2.84
**MDz[1,2,5]**	1.383	0.9931	0.002836	-0.5771	1.382	3.352
**MDz[1,2,6]**	-1.073	0.6619	0.002951	-2.388	-1.071	0.2312
**MDz[1,2,7]**	-1.88	0.9049	0.00188	-3.669	-1.881	-0.08246
**MDz[1,2,8]**	-2.273	0.479	0.001047	-3.24	-2.268	-1.343
**MDz[1,3,4]**	-0.9077	1.333	0.003101	-3.556	-0.9041	1.721
**MDz[1,3,5]**	0.06801	1.127	0.0025	-2.162	0.06875	2.299
**MDz[1,3,6]**	-2.387	0.8373	0.002216	-4.05	-2.383	-0.7433
**MDz[1,3,7]**	-3.195	0.8376	0.001359	-4.855	-3.195	-1.534
**MDz[1,3,8]**	-3.588	0.7473	0.00184	-5.087	-3.581	-2.131
**MDz[1,4,5]**	0.9757	1.367	0.002466	-1.727	0.9708	3.687
**MDz[1,4,6]**	-1.48	1.037	0.001704	-3.534	-1.48	0.5715
**MDz[1,4,7]**	-2.287	1.509	0.003545	-5.268	-2.287	0.7023
**MDz[1,4,8]**	-2.68	1.314	0.003484	-5.296	-2.674	-0.09592

**MDz[1,5,6]**	-2.455	0.8902	0.001644	-4.226	-2.453	-0.7074
**MDz[1,5,7]**	-3.263	1.329	0.002909	-5.891	-3.263	-0.634
**MDz[1,5,8]**	-3.656	1.095	0.002772	-5.844	-3.648	-1.51
**MDz[1,6,7]**	-0.8073	1.095	0.002839	-2.965	-0.8093	1.369
**MDz[1,6,8]**	-1.201	0.8028	0.002779	-2.8	-1.197	0.3744
**MDz[1,7,8]**	-0.3934	1.02	0.002021	-2.437	-0.3847	1.603
**SUCRA[1]**	0.009941	0.05011	8.64E-04	0	0	0.1429
**SUCRA[2]**	0.5161	0.09714	2.98E-04	0.2857	0.5714	0.7143
**SUCRA[3]**	0.252	0.1112	3.52E-04	0.1429	0.2857	0.4286
**SUCRA[4]**	0.434	0.1872	4.85E-04	0.1429	0.4286	0.8571
**SUCRA[5]**	0.2519	0.1343	4.24E-04	0	0.2857	0.5714
**SUCRA[6]**	0.7364	0.09431	2.47E-04	0.5714	0.7143	1
**SUCRA[7]**	0.8617	0.1304	2.75E-04	0.5714	0.8571	1
**SUCRA[8]**	0.938	0.0864	1.90E-04	0.7143	1	1
**SUCRAz[1]**	0.101	0.1159	6.01E-04	0	0.1429	0.4286
**SUCRAz[2]**	0.516	0.0974	3.10E-04	0.2857	0.5714	0.7143
**SUCRAz[3]**	0.2204	0.1383	4.22E-04	0	0.2857	0.4286
**SUCRAz[4]**	0.4181	0.2104	4.30E-04	0	0.4286	0.8571
**SUCRAz[5]**	0.2084	0.1717	3.67E-04	0	0.1429	0.5714
**SUCRAz[6]**	0.7364	0.09425	2.43E-04	0.5714	0.7143	1
**SUCRAz[7]**	0.8617	0.1307	2.76E-04	0.5714	0.8571	1
**SUCRAz[8]**	0.938	0.08639	1.90E-04	0.7143	1	1
**sd**	1.377	0.2482	0.001656	0.9778	1.347	1.947
**totresdev**	69.08	11.59	0.02489	48.23	68.46	93.51

Gender ratio=(n[female] x 2+ n[male] x1)/n[total]

or: Gender ratio≤1.5   orz: Gender ratio>1.5

4.4 FS-14 physical score of CFS patients

A. network map

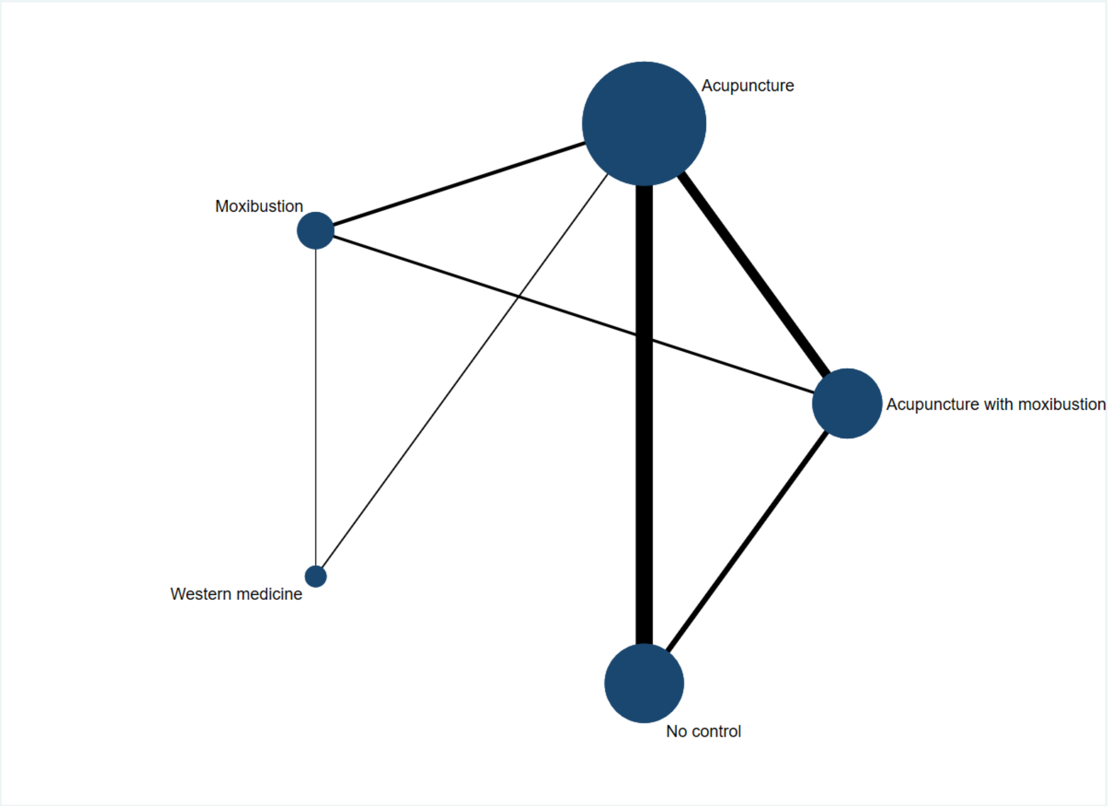

B.Contribution plot

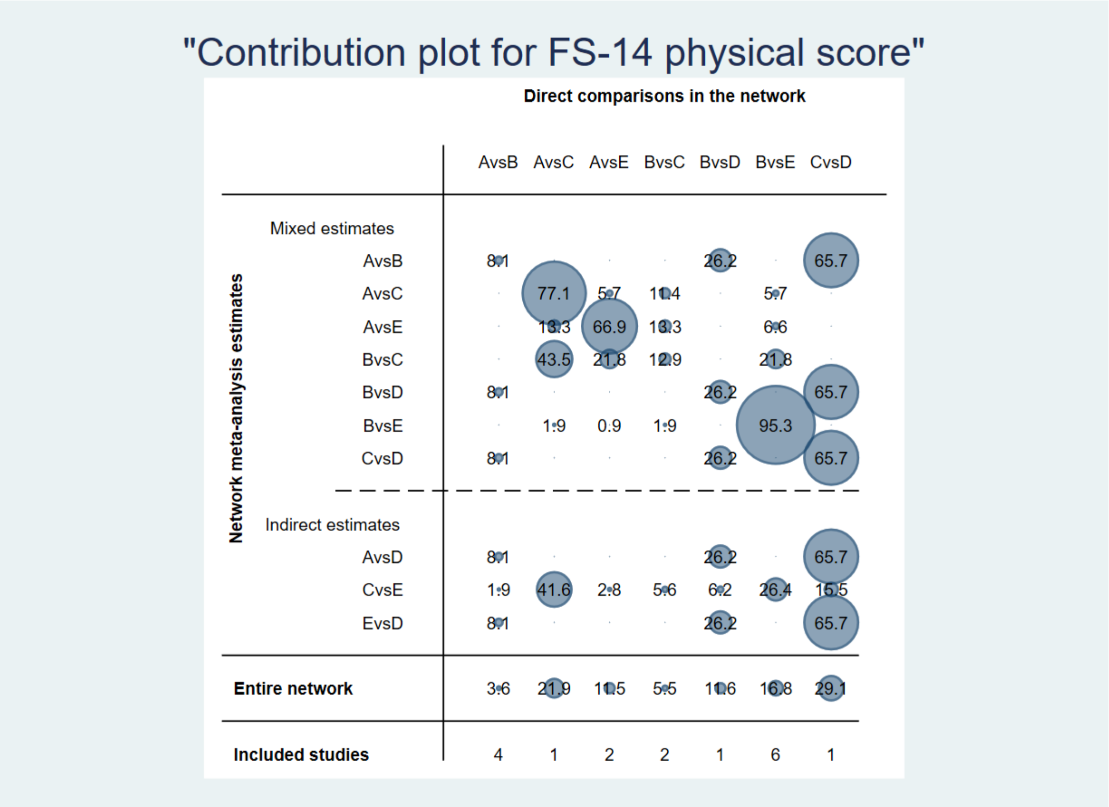

A- Acupuncture with moxibustion; B- Acupuncture; C- Moxibustion; E- Western medicine; F- No control

C.Convergence diagraph

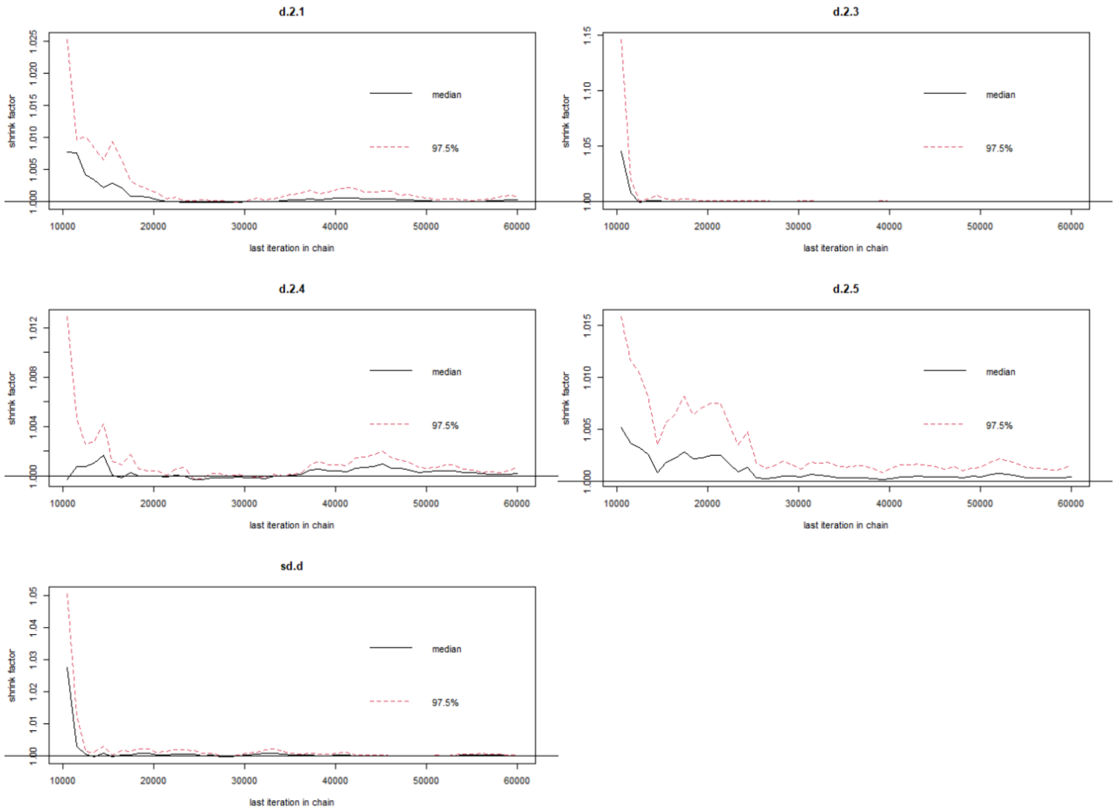

Potential scale reduction factors:

	Point est.	Upper C.I.
**d.2.1**	1	1
**d.2.3**	1	1
**d.2.4**	1	1
**d.2.5**	1	1
**sd.d**	1	1

1- Acupuncture with moxibustion; 2- Acupuncture; 3- Moxibustion; 4- Western medicine; 5- No control

D.Trajectories and density diagram

Results on the Log Odds Ratio scale

Iterations = 10010:60000

Thinning interval = 10

Number of chains = 4

Sample size per chain = 5000

1. **Empirical mean and standard deviation for each variable,plus standard error**

of the mean:

	Mean	SD	NaiveSE	Time-seriesSE
**d.2.1**	1.2119	0.4372	0.003092	0.003060
**d.2.3**	1.0951	0.5808	0.004107	0.004063
**d.2.4**	-1.0786	0.7334	0.005186	0.005175
**d.2.5**	-0.8530	0.3808	0.002692	0.002691
**sd.d**	0.9035	0.2427	0.001716	0.001742

2. **Quantiles for each variable:**

	2.5%	25%	50%	75%	97.5%
**d.2.1**	0.34393	0.9367	1.2162	1.4861	2.0766
**d.2.3**	-0.07591	0.7336	1.1007	1.4569	2.2597
**d.2.4**	-2.54393	-1.5381	-1.0774	-0.6191	0.3775
**d.2.5**	-1.60533	-1.0937	-0.8555	-0.6128	-0.0871
**sd.d**	0.55527	0.7335	0.8639	1.0263	1.5014

3. **Model fit (residual deviance):**
Dbar: 27.52494 pD: 26.74782 DIC:54.27276
27data points, ratio 0.983, I^2= 2%

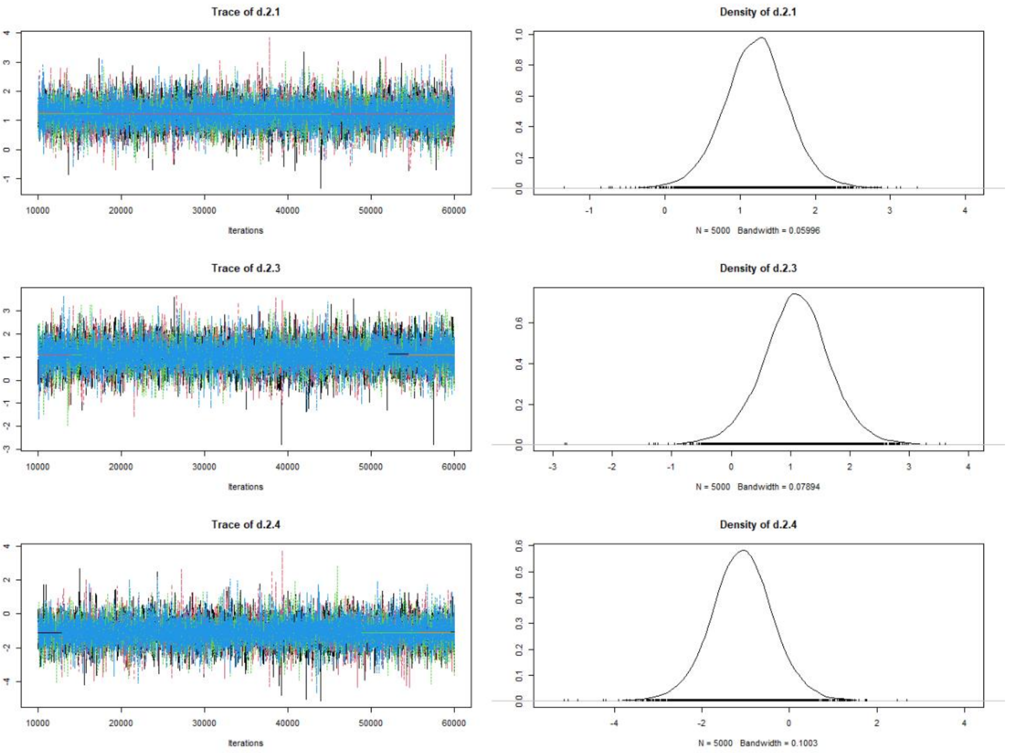

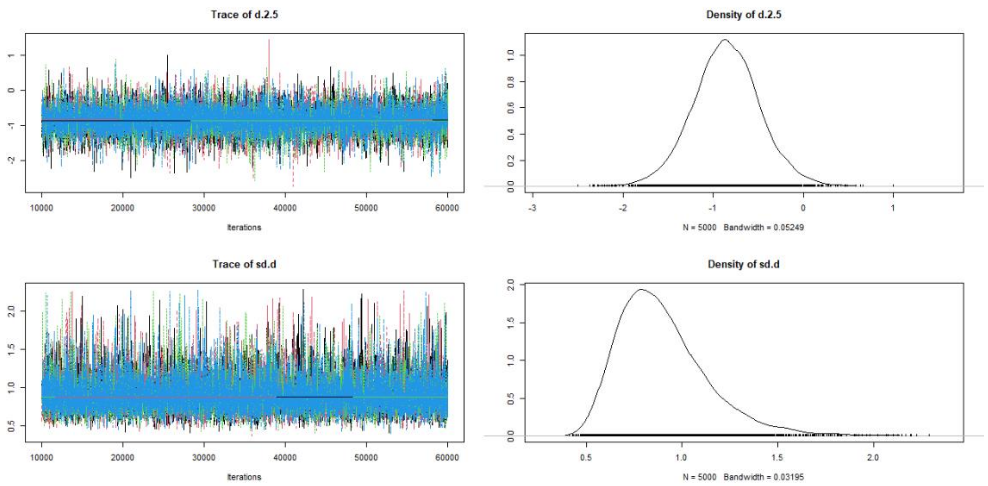

E.Forest plot

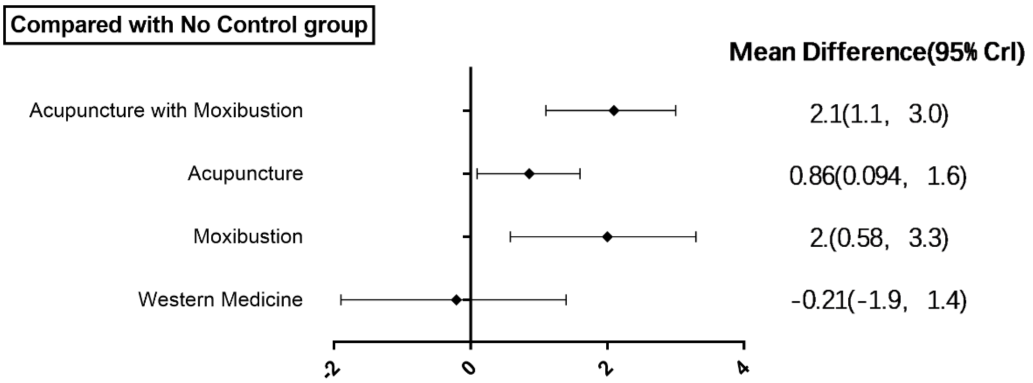

F.League table

**Acupuncture with moxibustion**				
**3.37 (1.41, 7.98)**	**Acupuncture**			
1.12 (0.3, 4.31)	0.33 (0.1, 1.08)	**Moxibustion**		
**9.87 (1.85, 51.66)**	2.95(0.69, 12.73)	**8.84 (2, 37.9)**	**Western medicine**	
**7.91 (2.92, 20.94)**	**2.35 (1.09, 4.98)**	**7.05 (1.79, 27.04)**	0.8 (0.15, 4.06)	**No control**

G.Inconsistency and Heterogeneity

a. Loops of Statistical Inconsistency:

Loop	IF	seIF	z_value	p_value	CI_95	Loop_Heterog_tau

						2
AM-Acu-NC	1.655	0.655	2.526	0.013	(0.37,2.94)	0.341
Acu-Mox-WM	0.897	0.272	3.291	0.001	(0.36,1.43)	0.000
AM-Acu-Mox	0.253	1.313	0.193	0.847	(0.00,2.83)	0.838

b. Side-splitting

**Side**	**Direct**		**Indirect**		**Difference**		**P>|z|**	**tau**
	Coef.	Std. Err.	Coef.	Std. Err.	Coef.	Std. Err.		
**A B**	-1.509609	.3989677	-1.890671	.7420173	-1.320542	.8413748	0.117	.7314626
**A C**	-.7203902	.8712461	.368167	.7723588	-1.088557	1.167275	0.351	.7946764
**A E**	-1.112339	.4726905	-3.046441	.4700077	1.934102	.6696152	0.004	.5736396
**B C**	1.220922	.6089278	.7705572	.9953524	.4503651	1.167956	0.700	.8263071
**B D**	-1.2	.8550631	-.8945828	1.023501	-.3054172	1.333674	0.819	.8322056
**B E**	-1.181286	.2602647	1.197459	.6590426	-2.378745	.7091628	0.001	.5304401
**C D**	-2.05	.8432037	-2.355364	1.0333	.3053638	1.33368	0.819	.832206

*** All the evidence about these contrasts comes from the trials which directly compare them.**

c. Design-by-treatment test

chi2(6) = 24.36

Prob > chi2 = 0.0004

H.Comparison-adjusted funnel plot

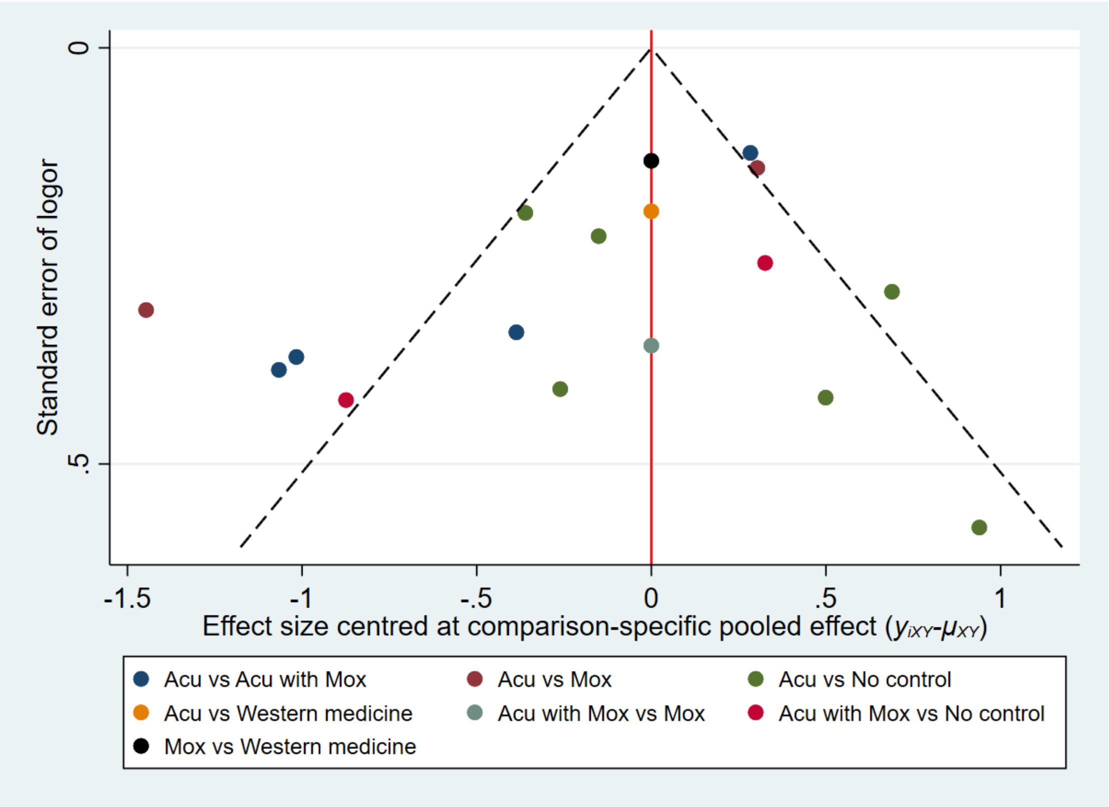

I.SUCRA diagram

a. Ranking probability diagram

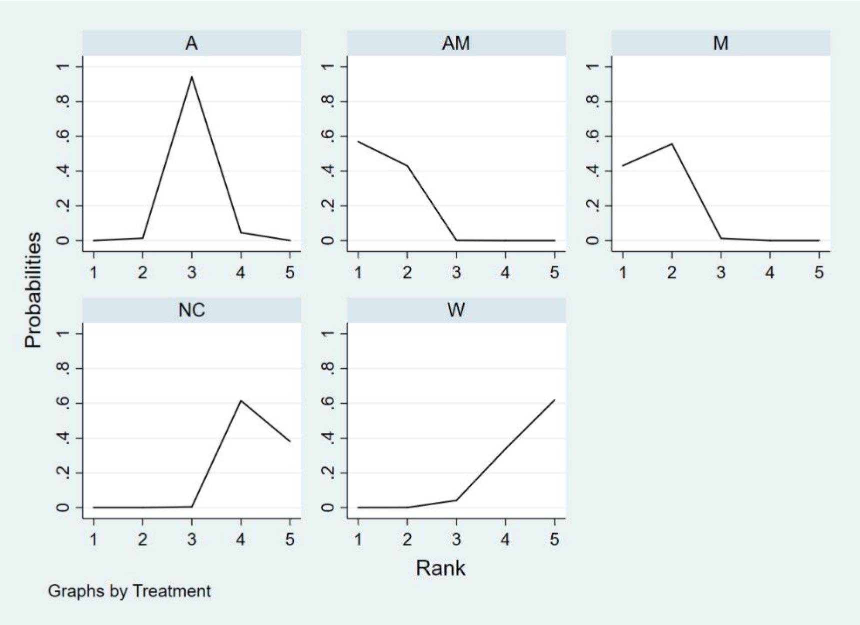

b. Cumulative probability ranking diagram

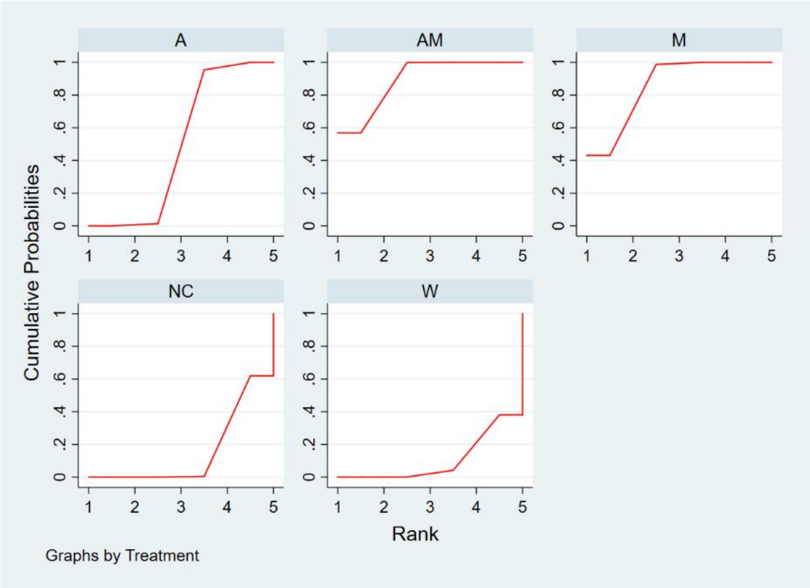

AM-Acupuncture with moxibustion; A-Acupuncture; M-Moxibustion; W-Western medicine; NC-No control.

J.Subgroup analysis

a. age

	mean	sd	MC_error	val2.5pc	median	val97.5pc
B	-0.5121	1.156	0.0107	-2.811	-0.5133	1.786
MD[1,2]	-1.112	0.5115	0.002848	-2.124	-1.114	-0.08831
MD[1,3]	-0.03747	0.725	0.002725	-1.484	-0.03596	1.399
MD[1,4]	-2.198	0.8973	0.003184	-3.987	-2.199	-0.4069

**MD[1,5]**	-1.991	0.5532	0.002465	-3.083	-1.995	-0.8842
**MD[2,3]**	1.075	0.6158	0.001153	-0.1611	1.078	2.292
**MD[2,4]**	-1.086	0.7757	0.001354	-2.641	-1.085	0.4565
**MD[2,5]**	-0.8786	0.4048	8.35E-04	-1.679	-0.8794	-0.0702
**MD[3,4]**	-2.16	0.7736	0.001144	-3.699	-2.162	-0.6067
**MD[3,5]**	-1.953	0.7191	0.001347	-3.372	-1.958	-0.5096
**MD[4,5]**	0.207	0.8682	0.001631	-1.514	0.2049	1.95
**MDz[1,1,2]**	-1.624	1.038	0.008389	-3.682	-1.629	0.4508
**MDz[1,1,3]**	-0.5496	1.206	0.008954	-2.956	-0.5499	1.855
**MDz[1,1,4]**	-2.71	1.294	0.008782	-5.286	-2.711	-0.1228
**MDz[1,1,5]**	-2.503	1.114	0.008906	-4.71	-2.51	-0.2777
**MDz[1,2,3]**	1.075	0.6158	0.001153	-0.1611	1.078	2.292
**MDz[1,2,4]**	-1.086	0.7757	0.001354	-2.641	-1.085	0.4565
**MDz[1,2,5]**	-0.8786	0.4048	8.35E-04	-1.679	-0.8794	-0.0702
**MDz[1,3,4]**	-2.16	0.7736	0.001144	-3.699	-2.162	-0.6067
**MDz[1,3,5]**	-1.953	0.7191	0.001347	-3.372	-1.958	-0.5096
**MDz[1,4,5]**	0.207	0.8682	0.001631	-1.514	0.2049	1.95
**SUCRA[1]**	0.1272	0.1398	4.94E-04	0	0	0.25
**SUCRA[2]**	0.5082	0.09711	1.89E-04	0.25	0.5	0.75
**SUCRA[3]**	0.1436	0.1507	4.10E-04	0	0.25	0.5
**SUCRA[4]**	0.8783	0.1694	3.15E-04	0.5	1	1
**SUCRA[5]**	0.8427	0.1339	2.38E-04	0.75	0.75	1
**SUCRAz[1]**	0.1008	0.1759	0.001199	0	0	0.5
**SUCRAz[2]**	0.4987	0.1061	3.15E-04	0.25	0.5	0.75
**SUCRAz[3]**	0.1856	0.1384	6.85E-04	0	0.25	0.5
**SUCRAz[4]**	0.8758	0.1728	3.53E-04	0.5	1	1
**SUCRAz[5]**	0.8392	0.1388	3.14E-04	0.5	0.75	1
**sd**	0.9502	0.2745	0.001115	0.569	0.9007	1.619
**totresdev**	27.56	7.375	0.0154	15.05	26.9	43.75

or: Average age≤40 years orz: Average age>40 years

b. course of disease

	**mean**	**sd**	**MC_error**	**val2.5pc**	**median**	**val97.5pc**
**B**	-1.267	13.63	0.3717	-4.171	-1.387	11.28
**MD[1,2]**	-0.9293	2.732	0.07256	-2.734	-0.9056	0.3072
**MD[1,3]**	0.1128	2.239	0.05542	-1.866	0.1372	1.762
**MD[1,4]**	-2.034	2.609	0.0639	-4.421	-2.009	0.01093
**MD[1,5]**	-1.853	2.163	0.05587	-3.464	-1.838	-0.5558
**MD[2,3]**	1.042	1.025	0.01722	-0.3378	1.045	2.438
**MD[2,4]**	-1.104	1.075	0.008847	-2.798	-1.103	0.584
**MD[2,5]**	-0.924	0.8146	0.01672	-1.823	-0.9319	0.05945
**MD[3,4]**	-2.146	1.072	0.008572	-3.832	-2.147	-0.46
**MD[3,5]**	-1.966	0.9496	0.001652	-3.483	-1.976	-0.3849
**MD[4,5]**	0.1804	1.172	0.008212	-1.67	0.1707	2.081

**MDz[1,1,2]**	-2.196	10.99	0.2991	-4.773	-2.292	6.751
**MDz[1,1,3]**	-1.154	11.64	0.3163	-4.157	-1.247	7.771
**MDz[1,1,4]**	-3.3	11.35	0.3078	-6.525	-3.393	4.958
**MDz[1,1,5]**	-3.12	11.6	0.3158	-5.886	-3.226	6.272
**MDz[1,2,3]**	1.042	1.025	0.01722	-0.3378	1.045	2.438
**MDz[1,2,4]**	-1.104	1.075	0.008847	-2.798	-1.103	0.584
**MDz[1,2,5]**	-0.924	0.8146	0.01672	-1.823	-0.9319	0.05945
**MDz[1,3,4]**	-2.146	1.072	0.008572	-3.832	-2.147	-0.46
**MDz[1,3,5]**	-1.966	0.9496	0.001652	-3.483	-1.976	-0.3849
**MDz[1,4,5]**	0.1804	1.172	0.008212	-1.67	0.1707	2.081
**SUCRA[1]**	0.1684	0.1734	0.002909	0	0.25	0.5
**SUCRA[2]**	0.5006	0.1134	0.001753	0.25	0.5	0.75
**SUCRA[3]**	0.1228	0.1602	0.001604	0	0	0.5
**SUCRA[4]**	0.8717	0.1775	0.001673	0.5	1	1
**SUCRA[5]**	0.8365	0.1459	0.001437	0.5	0.75	1
**SUCRAz[1]**	0.05416	0.1827	0.004482	0	0	1
**SUCRAz[2]**	0.5026	0.08937	6.74E-04	0.25	0.5	0.75
**SUCRAz[3]**	0.2395	0.1162	0.001616	0	0.25	0.5
**SUCRAz[4]**	0.8704	0.1745	0.001909	0.5	1	1
**SUCRAz[5]**	0.8334	0.1573	0.002195	0.5	0.75	1
**sd**	1.007	0.8305	0.02165	0.4864	0.8047	4.89
**totresdev**	45.13	5433	10.27	15.15	27	43.98

or: Average course of disease≤1 year orz: Average course of disease>1 year

c. Duration of treatment

	**mean**	**sd**	**MC_error**	**val2.5pc**	**median**	**val97.5pc**
**B**	23.84	135.4	3.698	-113.1	-46.76	239.8
**MD[1,2]**	-24.72	135.4	3.698	-240.6	45.81	112.2
**MD[1,3]**	-23.91	135.4	3.698	-239.6	46.54	112.9
**MD[1,4]**	-25.42	135.4	3.697	-241.1	44.96	111.4
**MD[1,5]**	-25.55	135.4	3.698	-241.4	44.96	111.3
**MD[2,3]**	0.8096	0.7615	0.00124	-0.5874	0.8144	2.183
**MD[2,4]**	-0.7042	0.8794	0.001366	-2.455	-0.7014	1.033
**MD[2,5]**	-0.8329	0.5423	7.90E-04	-1.889	-0.8318	0.2147
**MD[3,4]**	-1.514	0.9086	0.00117	-3.252	-1.517	0.2308
**MD[3,5]**	-1.643	0.941	0.001662	-3.332	-1.647	0.05976
**MD[4,5]**	-0.1287	1.023	0.00171	-2.142	-0.1306	1.894
**MDz[1,1,2]**	-0.8751	0.5552	0.001393	-1.9	-0.8762	0.1585
**MDz[1,1,3]**	-0.06547	0.9131	0.002156	-1.652	-0.06134	1.512
**MDz[1,1,4]**	-1.579	1.01	0.00226	-3.543	-1.578	0.3872
**MDz[1,1,5]**	-1.708	0.6195	0.001322	-2.926	-1.709	-0.4852
**MDz[1,2,3]**	0.8096	0.7615	0.00124	-0.5874	0.8144	2.183
**MDz[1,2,4]**	-0.7042	0.8794	0.001366	-2.455	-0.7014	1.033
**MDz[1,2,5]**	-0.8329	0.5423	7.90E-04	-1.889	-0.8318	0.2147

**MDz[1,3,4]**	-1.514	0.9086	0.00117	-3.252	-1.517	0.2308
**MDz[1,3,5]**	-1.643	0.941	0.001662	-3.332	-1.647	0.05976
**MDz[1,4,5]**	-0.1287	1.023	0.00171	-2.142	-0.1306	1.894
**SUCRA[1]**	0.6666	0.4714	0.01287	0	1	1
**SUCRA[2]**	0.3664	0.1861	0.003253	0	0.25	0.75
**SUCRA[3]**	0.1275	0.169	0.003213	0	0	0.5
**SUCRA[4]**	0.6369	0.2358	0.003185	0.25	0.75	1
**SUCRA[5]**	0.7026	0.1995	0.003254	0.25	0.75	1
**SUCRAz[1]**	0.1414	0.1677	3.72E-04	0	0	0.5
**SUCRAz[2]**	0.5223	0.1545	1.97E-04	0.25	0.5	0.75
**SUCRAz[3]**	0.1778	0.197	3.52E-04	0	0.25	0.75
**SUCRAz[4]**	0.7909	0.2333	3.70E-04	0.25	0.75	1
**SUCRAz[5]**	0.8677	0.1644	2.54E-04	0.5	1	1
**sd**	1.072	0.3292	8.32E-04	0.631	1.009	1.884
**totresdev**	48.37	9146	15.38	12.32	23.23	39.18

or: Duration of treatment≤4 weeks orz: Duration of treatment>4 weeks

d. Principle of acupoint selection

	**mean**	**sd**	**MC_error**	**val2.5pc**	**median**	**val97.5pc**
**B**	-0.0897	100	0.1485	-196.4	-0.2775	196
**MD[1,2]**	-1.211	0.4375	0.001396	-2.078	-1.212	-0.3352
**MD[1,3]**	-0.1137	0.672	0.001864	-1.457	-0.1112	1.219
**MD[1,4]**	-2.287	0.8316	0.002187	-3.945	-2.286	-0.627
**MD[1,5]**	-2.067	0.5	0.001473	-3.056	-2.069	-1.065
**MD[2,3]**	1.097	0.585	0.001023	-0.07957	1.101	2.259
**MD[2,4]**	-1.076	0.7356	0.001316	-2.547	-1.074	0.3893
**MD[2,5]**	-0.8562	0.383	6.26E-04	-1.615	-0.8575	-0.09361
**MD[3,4]**	-2.173	0.7334	0.001045	-3.632	-2.175	-0.7051
**MD[3,5]**	-1.953	0.6854	0.001316	-3.31	-1.958	-0.575
**MD[4,5]**	0.2199	0.8231	0.00156	-1.415	0.2184	1.861
**MDz[1,1,2]**	-1.3	100	0.1485	-197.6	-1.48	194.8
**MDz[1,1,3]**	-0.2034	100	0.1485	-196.4	-0.4027	195.9
**MDz[1,1,4]**	-2.377	100	0.1485	-198.7	-2.586	193.7
**MDz[1,1,5]**	-2.157	100	0.1485	-198.4	-2.334	193.9
**MDz[1,2,3]**	1.097	0.585	0.001023	-0.07957	1.101	2.259
**MDz[1,2,4]**	-1.076	0.7356	0.001316	-2.547	-1.074	0.3893
**MDz[1,2,5]**	-0.8562	0.383	6.26E-04	-1.615	-0.8575	-0.09361
**MDz[1,3,4]**	-2.173	0.7334	0.001045	-3.632	-2.175	-0.7051
**MDz[1,3,5]**	-1.953	0.6854	0.001316	-3.31	-1.958	-0.575
**MDz[1,4,5]**	0.2199	0.8231	0.00156	-1.415	0.2184	1.861
**SUCRA[1]**	0.1101	0.1301	3.23E-04	0	0	0.25
**SUCRA[2]**	0.5108	0.08605	1.29E-04	0.25	0.5	0.75
**SUCRA[3]**	0.1534	0.1438	3.24E-04	0	0.25	0.5
**SUCRA[4]**	0.8843	0.162	2.98E-04	0.5	1	1

**SUCRA[5]**	0.8414	0.131	2.33E-04	0.75	0.75	1
**SUCRAz[1]**	0.4934	0.498	7.51E-04	0	0	1
**SUCRAz[2]**	0.3887	0.1504	2.32E-04	0.25	0.5	0.75
**SUCRAz[3]**	0.1358	0.1375	2.11E-04	0	0.25	0.25
**SUCRAz[4]**	0.7634	0.201	3.39E-04	0.25	0.75	1
**SUCRAz[5]**	0.7188	0.1809	3.01E-04	0.5	0.75	1
**sd**	0.905	0.2472	7.18E-04	0.5527	0.8628	1.509
**totresdev**	27.5	7.351	0.01557	15.05	26.84	43.71

or: fixed points orz: semi-fixed points

e.gender

	**mean**	**sd**	**MC_error**	**val2.5pc**	**median**	**val97.5pc**
**B**	-10.97	83.5	2.45	-150.5	29.04	97.68
**MD[1,2]**	9.76	83.5	2.45	-98.62	-30.25	149.3
**MD[1,3]**	10.86	83.5	2.45	-97.42	-29.13	150.4
**MD[1,4]**	8.685	83.5	2.45	-99.4	-31.28	148.3
**MD[1,5]**	8.904	83.5	2.45	-99.38	-31.1	148.5
**MD[2,3]**	1.097	0.5817	0.001022	-0.07314	1.101	2.249
**MD[2,4]**	-1.075	0.7336	0.001369	-2.544	-1.073	0.3842
**MD[2,5]**	-0.8562	0.3819	6.72E-04	-1.615	-0.8566	-0.09591
**MD[3,4]**	-2.172	0.7305	0.001041	-3.631	-2.173	-0.7083
**MD[3,5]**	-1.953	0.6831	0.001335	-3.303	-1.958	-0.5796
**MD[4,5]**	0.219	0.8206	0.001642	-1.414	0.2166	1.865
**MDz[1,1,2]**	-1.212	0.4363	0.001331	-2.079	-1.213	-0.3396
**MDz[1,1,3]**	-0.1146	0.6682	0.001805	-1.452	-0.1136	1.211
**MDz[1,1,4]**	-2.287	0.8291	0.002152	-3.941	-2.287	-0.6323
**MDz[1,1,5]**	-2.068	0.5	0.001428	-3.058	-2.07	-1.069
**MDz[1,2,3]**	1.097	0.5817	0.001022	-0.07314	1.101	2.249
**MDz[1,2,4]**	-1.075	0.7336	0.001369	-2.544	-1.073	0.3842
**MDz[1,2,5]**	-0.8562	0.3819	6.72E-04	-1.615	-0.8566	-0.09591
**MDz[1,3,4]**	-2.172	0.7305	0.001041	-3.631	-2.173	-0.7083
**MDz[1,3,5]**	-1.953	0.6831	0.001335	-3.303	-1.958	-0.5796
**MDz[1,4,5]**	0.219	0.8206	0.001642	-1.414	0.2166	1.865
**SUCRA[1]**	0.3478	0.4723	0.01381	0	0	1
**SUCRA[2]**	0.4251	0.1456	0.003478	0.25	0.5	0.75
**SUCRA[3]**	0.1706	0.1327	0.00348	0	0.25	0.25
**SUCRA[4]**	0.8006	0.1968	0.003457	0.5	0.75	1
**SUCRA[5]**	0.7558	0.1768	0.003476	0.5	0.75	1
**SUCRAz[1]**	0.1097	0.13	3.13E-04	0	0	0.25
**SUCRAz[2]**	0.5109	0.08565	1.34E-04	0.25	0.5	0.75
**SUCRAz[3]**	0.1535	0.1434	3.16E-04	0	0.25	0.5
**SUCRAz[4]**	0.8846	0.1616	3.05E-04	0.5	1	1
**SUCRAz[5]**	0.8414	0.131	2.43E-04	0.75	0.75	1
**sd**	0.9028	0.2456	7.24E-04	0.5521	0.8609	1.499

totresdev	27.5	7.353	0.01491	15.03	26.86	43.71

Gender ratio=(n[female] x 2+ n[male] x1)/n[total]

or: Gender ratio≤1.5   orz: Gender ratio>1.5

4.5 FS-14 mental score of CFS patients

A.network map

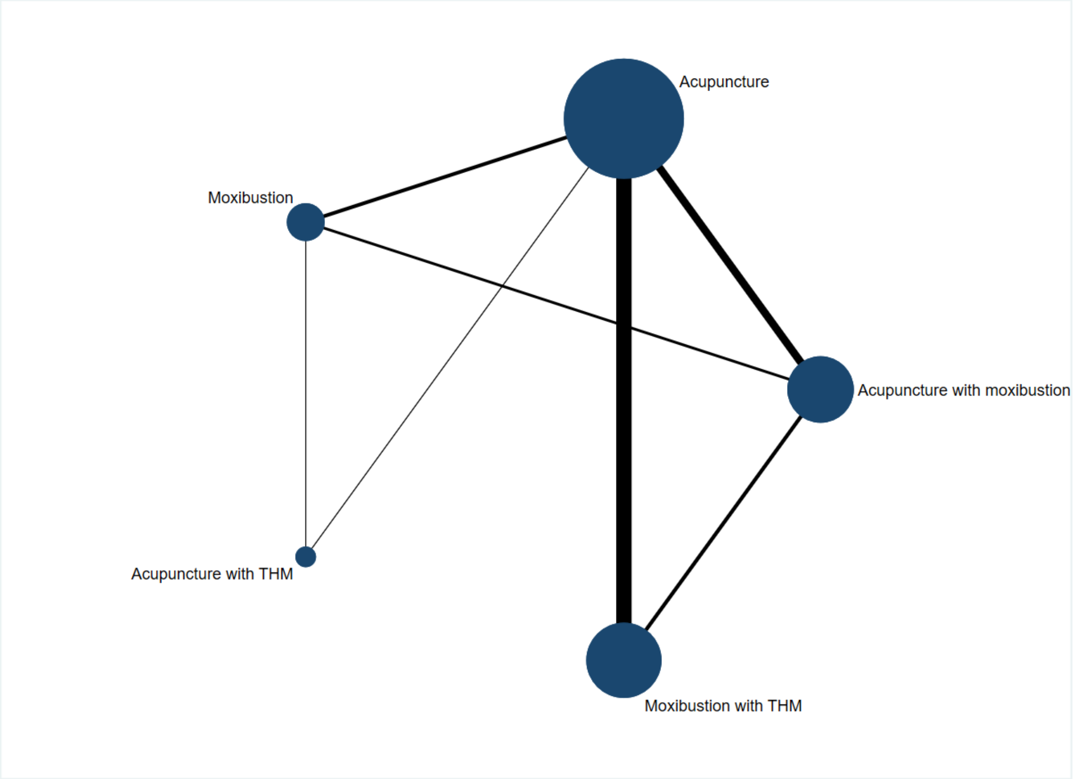

B.Contribution plot

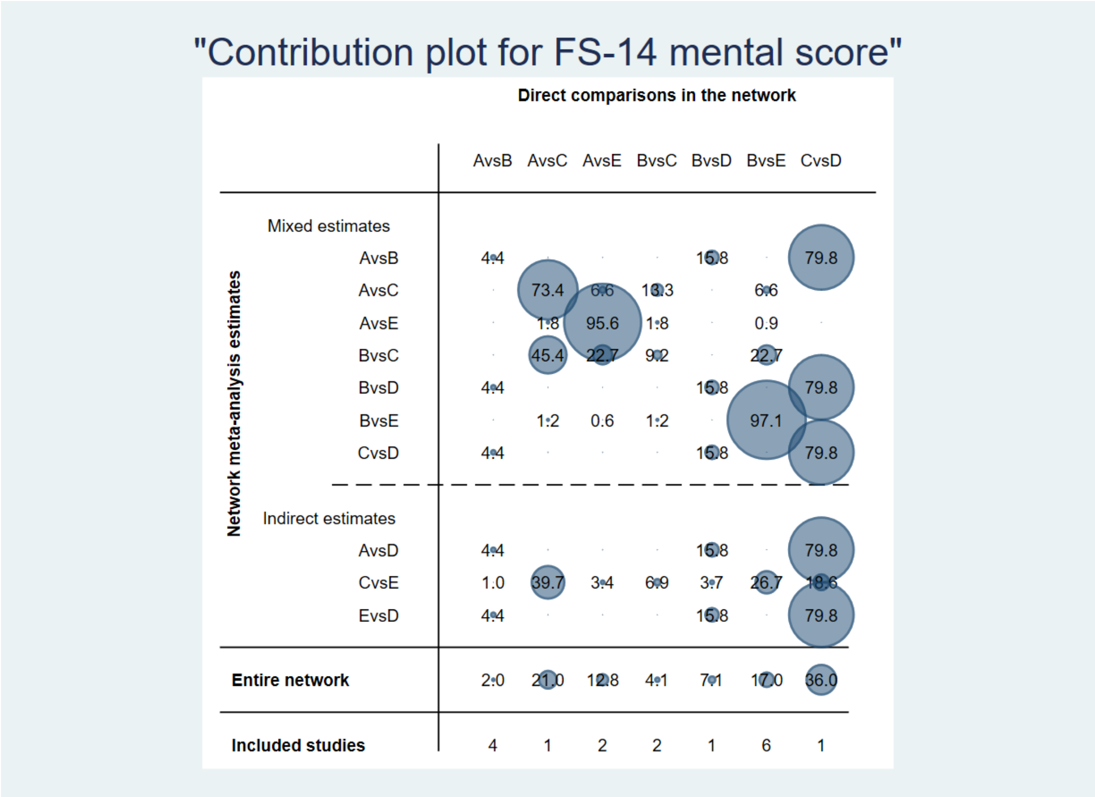

A- Acupuncture with moxibustion; B- Acupuncture; C- Moxibustion; E- Western medicine; F- No control

C.Convergence diagraph

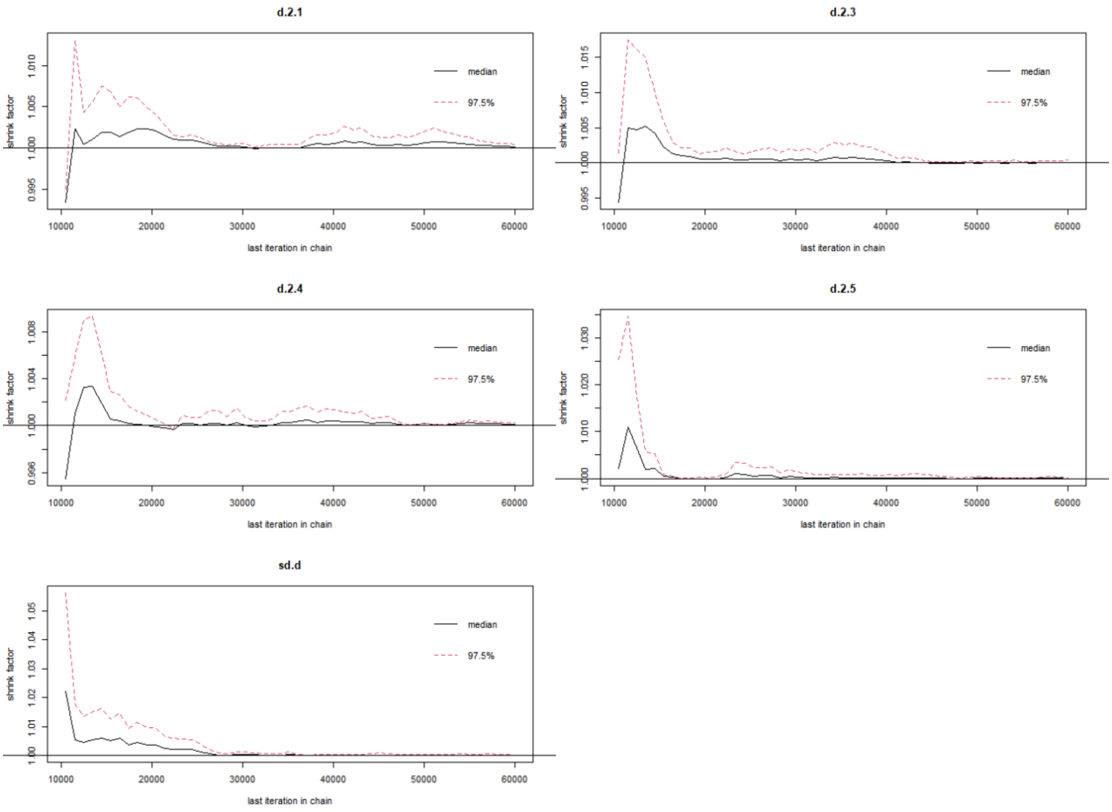

Potential scale reduction factors:

	Point est.	Upper C.I.
**d.2.1**	1	1
**d.2.3**	1	1
**d.2.4**	1	1
**d.2.5**	1	1
**sd.d**	1	1

1- Acupuncture with moxibustion; 2- Acupuncture; 3- Moxibustion; 4- Western medicine; 5- No control

D.Trajectories and density diagram

Results on the Log Odds Ratio scale

Iterations = 10010:60000

Thinning interval = 10

Number of chains = 4

Sample size per chain = 5000

1. **Empirical mean and standard deviation for each variable,plus standard error of the mean:**

	Mean	SD	NaiveSE	Time-seriesSE
**d.2.1**	0.8415	0.4661	0.003296	0.003329
**d.2.3**	0.8173	0.6300	0.004455	0.004384
**d.2.4**	-0.7017	0.7871	0.005566	0.005566

**d.2.5**	-0.9343	0.4096	0.002896	0.002897
**sd.d**	0.9828	0.2590	0.001831	0.001879

2. **Quantiles for each variable:**

	2.5%	25%	50%	75%	97.5%
**d.2.1**	-0.08802	0.5496	0.8435	1.1323	1.7593
**d.2.3**	-0.45380	0.4254	0.8198	1.2072	2.0794
**d.2.4**	-2.30161	-1.1918	-0.6961	-0.2080	0.8723
**d.2.5**	-1.75546	-1.1912	-0.9332	-0.6772	-0.1181
**sd.d**	0.60651	0.8016	0.9373	1.1131	1.6296

3. **Model fit (residual deviance):**

Dbar: 27.70335 pD: 27.10428 DIC:54.80762

28 data points, ratio 0.9894, I^2= 3%

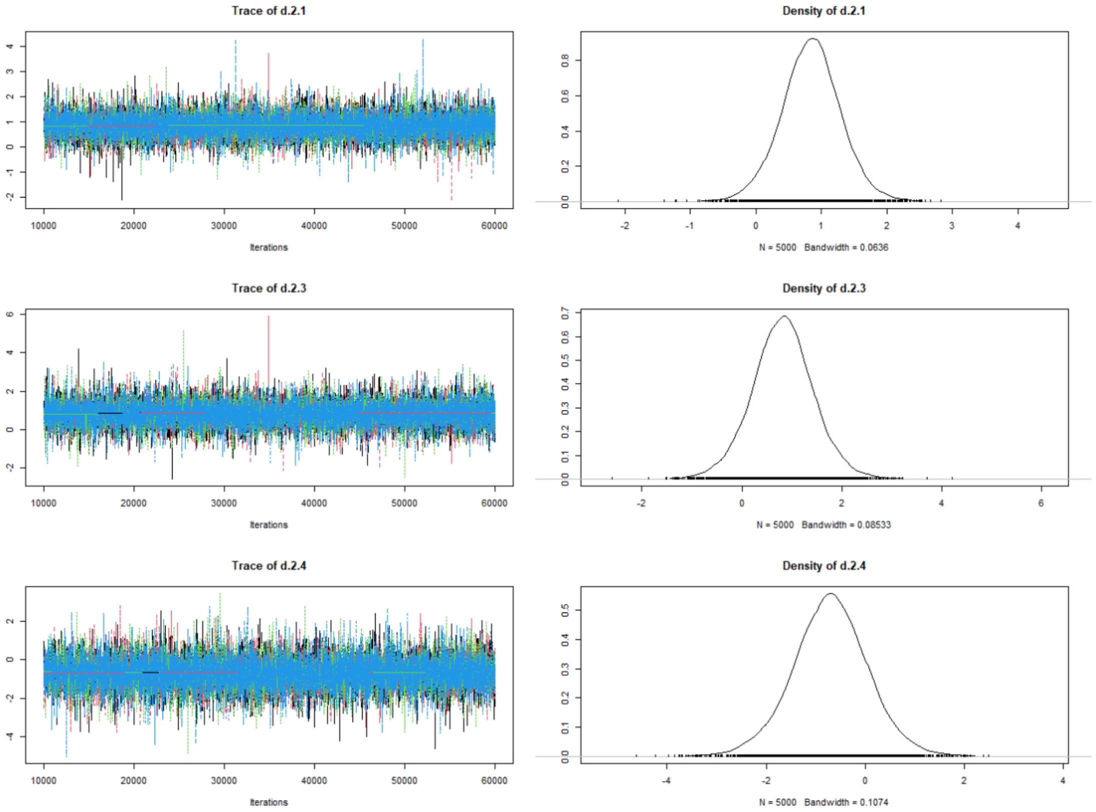

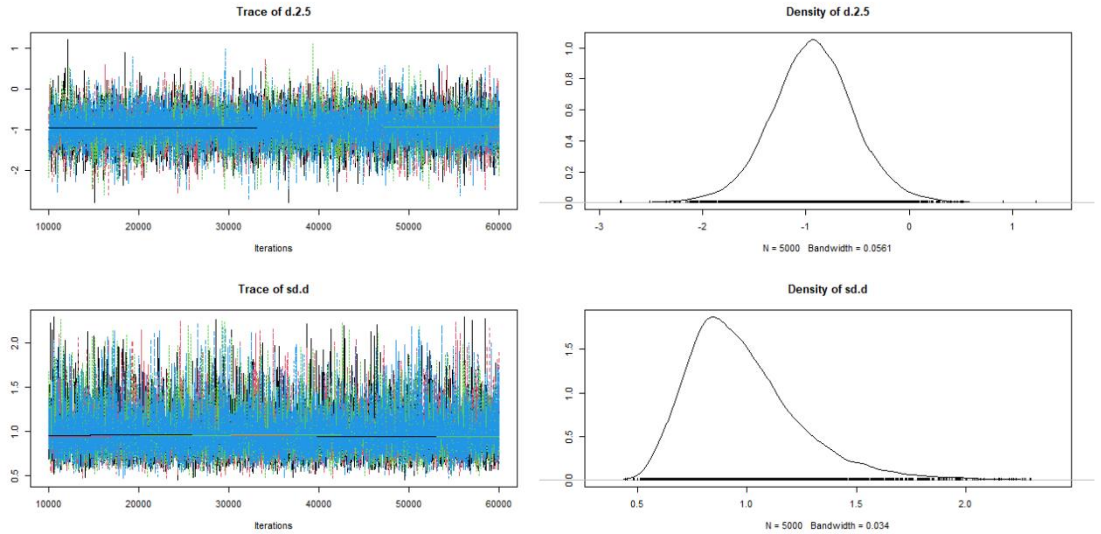

E.Forest plot

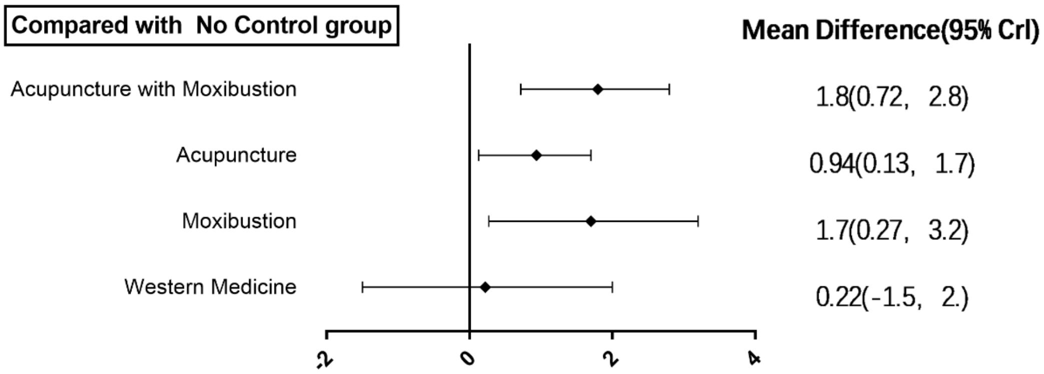

F.League table

Acupuncture with moxibustion			
2.32 (0.92, 5.81)	Acupuncture		
1.03 (0.25, 4.26)	0.44 (0.13, 1.57)	Moxibustion	
4.67 (0.8,	2.01 (0.42,	4.6 (0.97,	Western

27.85)	9.99)	21.55)	**medicine**	
**5.88 (2.03, 17.24)**	**2.54 (1.13, 5.79)**	**5.77 (1.32, 25.25)**	1.26 (0.21, 7.36)	**No control**

G.Inconsistency and Heterogeneity

a. Loops of Statistical Inconsistency:

Loop	IF	seIF	z_value	p_value	CI_95	Loop_Heterog_tau2
AM-Acu-WM	1.528	0.889	1.718	0.886	(0.00,3.27)	0.761
AM-Acu-Mox	0.745	1.657	0.450	0.653	(0.00,3.99)	1.403
Acu-Mo-NC	0.335	0.228	1.466	0.143	(0.00,0.78)	0.000

b. Side-splitting

Side	Direct		Indirect		Difference		P>|z|	tau
	Coef.	Std. Err.	Coef.	Std. Err.	Coef.	Std. Err.		
**A B**	-1.053246	.4494383	-.104083	.8483489	-.949163	.9617843	0.324	.8607026
**A C**	-.9358651	.9060654	.6794506	.8012152	-1.615316	1.20907	0.182	.8361518
**A E**	-.8042756	.4846723	-2.839311	.5019041	2.035035	.6980684	0.004	.641162
**B C**	.8918981	.6663418	.5902405	1.076576	.3016576	1.264029	0.811	.9079177
**B D**	-.5600001	.9198388	-.9203103	1.115159	.3603102	1.445574	0.803	.9093625
**B E**	-1.246478	.2995874	1.090854	.7647471	-2.337332	.8226802	0.004	.6541502
**C D**	-1.66	.9189004	-1.29962	1.115943	-.3603803	1.445582	0.803	.909363

*** All the evidence about these contrasts comes from the trials which directly compare them.**

c. Design-by-treatment test

chi2(6)=10.15

Prob>chi2=0.1187

H.Comparison-adjusted funnel plot

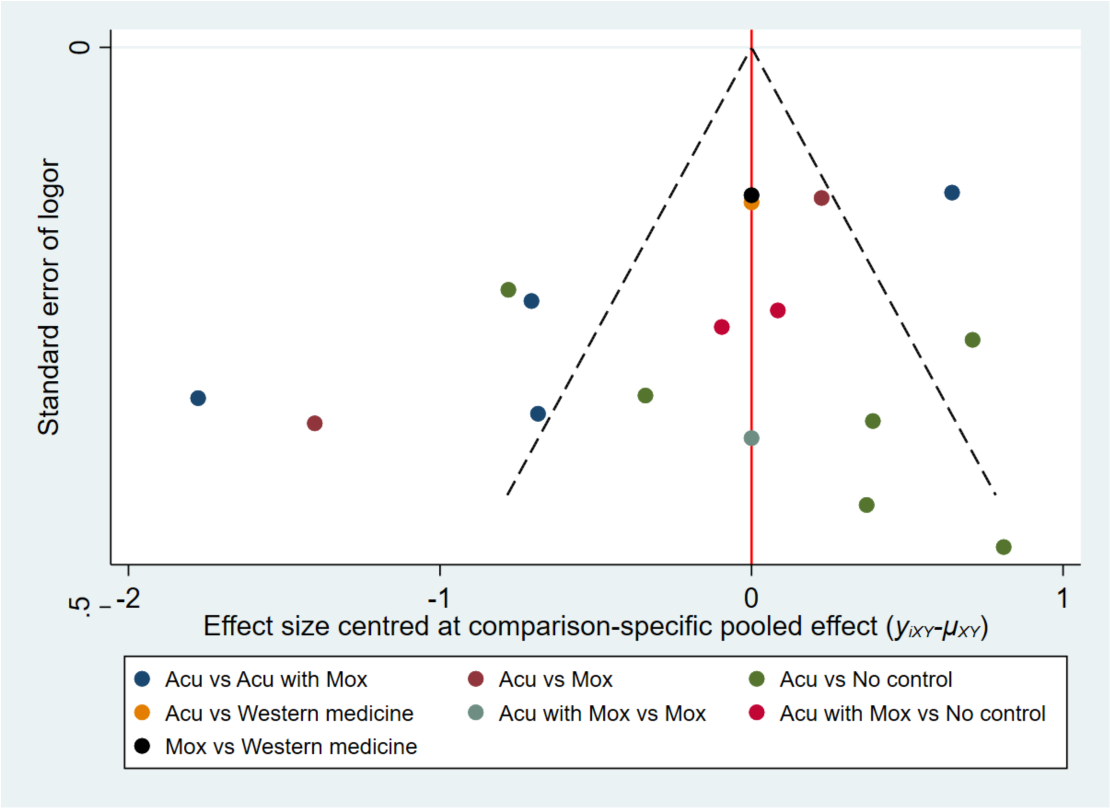

I.SUCRA diagram

a. **Ranking probability diagram**

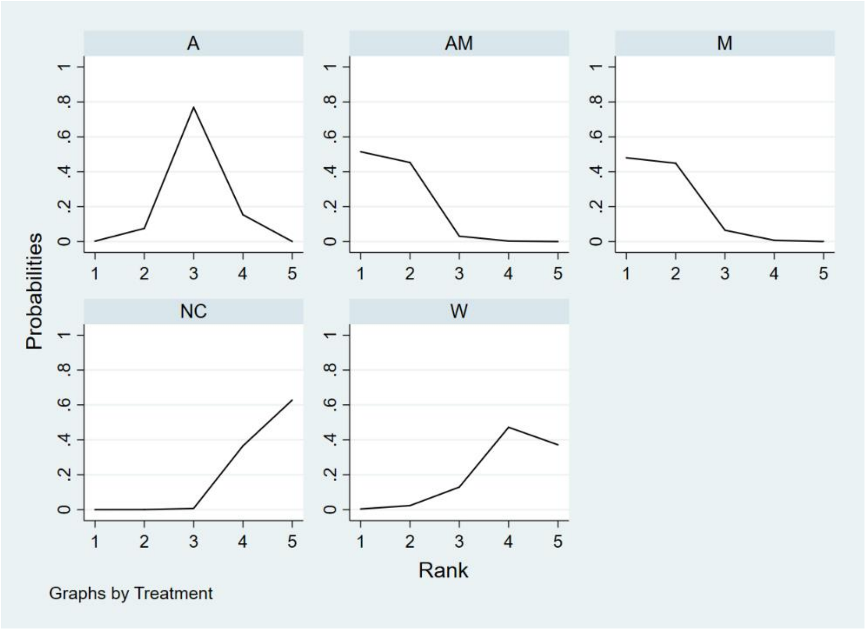

b. **Cumulative probability ranking diagram**

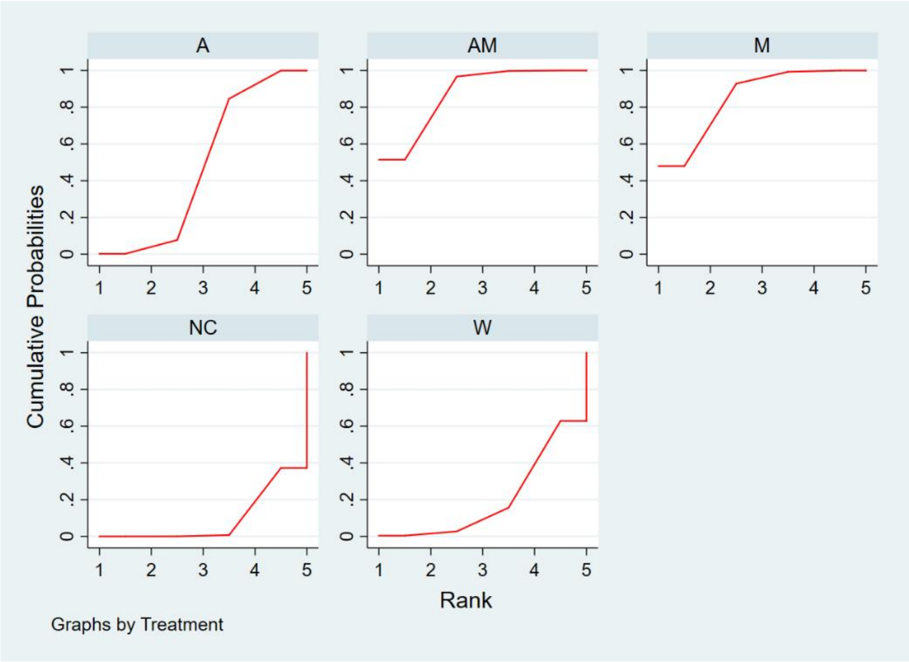

J.Subgroup analysis

a. age

	mean	sd	MC_error	val2.5pc	median	val97.5pc
**B**	-0.1317	1.224	0.01792	-2.544	-0.1394	2.333
**MD[1,2]**	-0.8154	0.5489	0.004006	-1.91	-0.8162	0.2829
**MD[1,3]**	-0.01827	0.7899	0.003541	-1.603	-0.01347	1.547
**MD[1,4]**	-1.526	0.9774	0.004062	-3.476	-1.523	0.426
**MD[1,5]**	-1.759	0.5926	0.003244	-2.942	-1.76	-0.5732
**MD[2,3]**	0.7972	0.6787	0.001381	-0.5689	0.8024	2.138
**MD[2,4]**	-0.7102	0.8508	0.001532	-2.415	-0.7084	0.9832
**MD[2,5]**	-0.944	0.4381	0.001072	-1.818	-0.9429	-0.07145
**MD[3,4]**	-1.507	0.85	0.001205	-3.199	-1.51	0.1972
**MD[3,5]**	-1.741	0.7871	0.001389	-3.299	-1.745	-0.159
**MD[4,5]**	-0.2337	0.9484	0.00177	-2.124	-0.2349	1.664
**MDz[1,1,2]**	-0.9471	1.1	0.01426	-3.125	-0.953	1.276
**MDz[1,1,3]**	-0.1499	1.289	0.0151	-2.712	-0.1503	2.437
**MDz[1,1,4]**	-1.657	1.388	0.01476	-4.41	-1.66	1.123
**MDz[1,1,5]**	-1.891	1.181	0.01512	-4.225	-1.897	0.4908
**MDz[1,2,3]**	0.7972	0.6787	0.001381	-0.5689	0.8024	2.138
**MDz[1,2,4]**	-0.7102	0.8508	0.001532	-2.415	-0.7084	0.9832
**MDz[1,2,5]**	-0.944	0.4381	0.001072	-1.818	-0.9429	-0.07145
**MDz[1,3,4]**	-1.507	0.85	0.001205	-3.199	-1.51	0.1972
**MDz[1,3,5]**	-1.741	0.7871	0.001389	-3.299	-1.745	-0.159
**MDz[1,4,5]**	-0.2337	0.9484	0.00177	-2.124	-0.2349	1.664
**SUCRA[1]**	0.1539	0.1758	8.55E-04	0	0.25	0.5

**SUCRA[2]**	0.5074	0.1517	4.28E-04	0.25	0.5	0.75
**SUCRA[3]**	0.1679	0.1915	5.00E-04	0	0.25	0.75
**SUCRA[4]**	0.7789	0.2294	4.58E-04	0.25	0.75	1
**SUCRA[5]**	0.892	0.1422	2.67E-04	0.5	1	1
**SUCRAz[1]**	0.1966	0.2682	0.002994	0	0	1
**SUCRAz[2]**	0.4787	0.1652	8.66E-04	0.25	0.5	0.75
**SUCRAz[3]**	0.1787	0.1846	0.001216	0	0.25	0.5
**SUCRAz[4]**	0.7663	0.2374	7.09E-04	0.25	0.75	1
**SUCRAz[5]**	0.8797	0.1565	6.67E-04	0.5	1	1
**sd**	1.05	0.296	0.0015	0.6381	0.9962	1.775
**totresdev**	27.74	7.414	0.01515	15.14	27.09	44.05

or: Average age≤40 years orz: Average age>40 years

b. course of disease

	**mean**	**sd**	**MC_error**	**val2.5pc**	**median**	**val97.5pc**
**B**	-1.863	0.9779	0.01834	-3.808	-1.871	0.1089
**MD[1,2]**	-0.4551	0.452	0.004129	-1.358	-0.4549	0.4471
**MD[1,3]**	0.2849	0.6505	0.003557	-1.038	0.2923	1.567
**MD[1,4]**	-1.195	0.7957	0.004089	-2.8	-1.191	0.3793
**MD[1,5]**	-1.487	0.4864	0.003293	-2.459	-1.487	-0.5193
**MD[2,3]**	0.7399	0.5526	0.001296	-0.3797	0.7468	1.825
**MD[2,4]**	-0.7399	0.6871	0.001242	-2.122	-0.7359	0.6262
**MD[2,5]**	-1.032	0.3613	0.001088	-1.751	-1.032	-0.31
**MD[3,4]**	-1.48	0.6867	0.001061	-2.848	-1.483	-0.09809
**MD[3,5]**	-1.772	0.644	0.00126	-3.035	-1.78	-0.4649
**MD[4,5]**	-0.2923	0.7691	0.001539	-1.822	-0.297	1.256
**MDz[1,1,2]**	-2.318	0.8734	0.01448	-4.059	-2.321	-0.5601
**MDz[1,1,3]**	-1.578	1.033	0.01536	-3.655	-1.572	0.4736
**MDz[1,1,4]**	-3.058	1.11	0.01491	-5.279	-3.054	-0.8391
**MDz[1,1,5]**	-3.351	0.9427	0.01539	-5.227	-3.354	-1.45
**MDz[1,2,3]**	0.7399	0.5526	0.001296	-0.3797	0.7468	1.825
**MDz[1,2,4]**	-0.7399	0.6871	0.001242	-2.122	-0.7359	0.6262
**MDz[1,2,5]**	-1.032	0.3613	0.001088	-1.751	-1.032	-0.31
**MDz[1,3,4]**	-1.48	0.6867	0.001061	-2.848	-1.483	-0.09809
**MDz[1,3,5]**	-1.772	0.644	0.00126	-3.035	-1.78	-0.4649
**MDz[1,4,5]**	-0.2923	0.7691	0.001539	-1.822	-0.297	1.256
**SUCRA[1]**	0.2233	0.1848	0.001171	0	0.25	0.75
**SUCRA[2]**	0.4761	0.1475	6.58E-04	0.25	0.5	0.75
**SUCRA[3]**	0.1056	0.1695	5.55E-04	0	0	0.5
**SUCRA[4]**	0.7828	0.2042	4.31E-04	0.25	0.75	1
**SUCRA[5]**	0.9122	0.1266	2.66E-04	0.75	1	1
**SUCRAz[1]**	0.01865	0.08393	0.001066	0	0	0.25
**SUCRAz[2]**	0.5095	0.1155	2.99E-04	0.25	0.5	0.75
**SUCRAz[3]**	0.263	0.1096	6.07E-04	0	0.25	0.5

**SUCRAz[4]**	0.7963	0.1756	3.53E-04	0.5	0.75	1
**SUCRAz[5]**	0.9126	0.1257	3.12E-04	0.75	1	1
**sd**	0.8429	0.2487	0.001563	0.4913	0.7997	1.449
**totresdev**	27.87	7.454	0.01486	15.22	27.21	44.24

or: Average course of disease≤1 year orz: Average course of disease>1 year

c. Duration of treatment

	**mean**	**sd**	**MC_error**	**val2.5pc**	**median**	**val97.5pc**
**B**	19.16	107.6	3.158	-75.51	-47.74	203.1
**MD[1,2]**	-20.39	107.6	3.158	-204.4	46.49	74.31
**MD[1,3]**	-19.29	107.6	3.157	-203.3	47.59	75.44
**MD[1,4]**	-21.46	107.6	3.157	-205.5	45.42	73.31
**MD[1,5]**	-21.18	107.6	3.158	-205.2	45.7	73.55
**MD[2,3]**	1.096	0.6408	0.001105	-0.1988	1.102	2.36
**MD[2,4]**	-1.076	0.8078	0.001435	-2.698	-1.073	0.5239
**MD[2,5]**	-0.7915	0.4929	7.99E-04	-1.78	-0.7902	0.1894
**MD[3,4]**	-2.173	0.8065	0.001142	-3.777	-2.175	-0.557
**MD[3,5]**	-1.888	0.7876	0.001474	-3.446	-1.893	-0.3067
**MD[4,5]**	0.2848	0.9374	0.001791	-1.579	0.2809	2.166
**MDz[1,1,2]**	-1.226	0.4847	0.001387	-2.182	-1.228	-0.2482
**MDz[1,1,3]**	-0.1294	0.7351	0.001949	-1.601	-0.1272	1.33
**MDz[1,1,4]**	-2.302	0.9132	0.002284	-4.125	-2.301	-0.4835
**MDz[1,1,5]**	-2.017	0.5768	0.00145	-3.167	-2.019	-0.8612
**MDz[1,2,3]**	1.096	0.6408	0.001105	-0.1988	1.102	2.36
**MDz[1,2,4]**	-1.076	0.8078	0.001435	-2.698	-1.073	0.5239
**MDz[1,2,5]**	-0.7915	0.4929	7.99E-04	-1.78	-0.7902	0.1894
**MDz[1,3,4]**	-2.173	0.8065	0.001142	-3.777	-2.175	-0.557
**MDz[1,3,5]**	-1.888	0.7876	0.001474	-3.446	-1.893	-0.3067
**MDz[1,4,5]**	0.2848	0.9374	0.001791	-1.579	0.2809	2.166
**SUCRA[1]**	0.6667	0.4714	0.01383	0	1	1
**SUCRA[2]**	0.3551	0.1569	0.003476	0	0.25	0.75
**SUCRA[3]**	0.09918	0.1386	0.003453	0	0	0.25
**SUCRA[4]**	0.7194	0.2011	0.00343	0.25	0.75	1
**SUCRA[5]**	0.6596	0.1883	0.0035	0.25	0.75	1
**SUCRAz[1]**	0.1114	0.1347	3.28E-04	0	0	0.25
**SUCRAz[2]**	0.5193	0.1066	1.63E-04	0.25	0.5	0.75
**SUCRAz[3]**	0.1598	0.1539	3.38E-04	0	0.25	0.5
**SUCRAz[4]**	0.8837	0.1721	3.22E-04	0.5	1	1
**SUCRAz[5]**	0.8258	0.1474	2.68E-04	0.5	0.75	1
**sd**	0.9874	0.3071	8.66E-04	0.5731	0.9284	1.748
**totresdev**	23.73	6.837	0.01386	12.29	23.08	38.85

or: Duration of treatment≤4 weeks orz: Duration of treatment>4 weeks

d. Principle of acupoint selection

	mean	sd	MC_error	val2.5pc	median	val97.5pc
**B**	-0.01789	100	0.1247	-195.9	-0.252	196.3
**MD[1,2]**	-0.8414	0.4912	0.001267	-1.766	-0.8433	0.09157
**MD[1,3]**	-0.03355	0.7493	0.001698	-1.482	-0.03107	1.401
**MD[1,4]**	-1.548	0.9	0.001909	-3.335	-1.547	0.2374
**MD[1,5]**	-1.779	0.5469	0.001346	-2.835	-1.78	-0.7163
**MD[2,3]**	0.8079	0.6411	9.84E-04	-0.468	0.8124	2.058
**MD[2,4]**	-0.7062	0.8117	0.001242	-2.299	-0.7025	0.8748
**MD[2,5]**	-0.9373	0.4346	6.59E-04	-1.756	-0.9361	-0.1236
**MD[3,4]**	-1.514	0.8133	0.001044	-3.093	-1.516	0.07922
**MD[3,5]**	-1.745	0.7537	0.00125	-3.207	-1.749	-0.2612
**MD[4,5]**	-0.2311	0.8982	0.001429	-1.993	-0.2338	1.546
**MDz[1,1,2]**	-0.8593	100	0.1248	-196.7	-1.09	195.5
**MDz[1,1,3]**	-0.05144	100	0.1248	-195.9	-0.283	196.4
**MDz[1,1,4]**	-1.565	100	0.1248	-197.4	-1.78	194.8
**MDz[1,1,5]**	-1.797	100	0.1248	-197.7	-2.007	194.6
**MDz[1,2,3]**	0.8079	0.6411	9.84E-04	-0.468	0.8124	2.058
**MDz[1,2,4]**	-0.7062	0.8117	0.001242	-2.299	-0.7025	0.8748
**MDz[1,2,5]**	-0.9373	0.4346	6.59E-04	-1.756	-0.9361	-0.1236
**MDz[1,3,4]**	-1.514	0.8133	0.001044	-3.093	-1.516	0.07922
**MDz[1,3,5]**	-1.745	0.7537	0.00125	-3.207	-1.749	-0.2612
**MDz[1,4,5]**	-0.2311	0.8982	0.001429	-1.993	-0.2338	1.546
**SUCRA[1]**	0.14	0.1591	3.46E-04	0	0	0.5
**SUCRA[2]**	0.514	0.1385	1.98E-04	0.25	0.5	0.75
**SUCRA[3]**	0.1637	0.182	3.19E-04	0	0.25	0.5
**SUCRA[4]**	0.786	0.2169	3.28E-04	0.25	0.75	1
**SUCRA[5]**	0.8962	0.1367	2.06E-04	0.75	1	1
**SUCRAz[1]**	0.495	0.4984	6.17E-04	0	0	1
**SUCRAz[2]**	0.3991	0.179	2.35E-04	0	0.5	0.75
**SUCRAz[3]**	0.1593	0.1621	2.24E-04	0	0.25	0.5
**SUCRAz[4]**	0.673	0.2307	3.27E-04	0.25	0.75	1
**SUCRAz[5]**	0.7737	0.1844	2.50E-04	0.5	0.75	1
**sd**	0.9875	0.2659	6.42E-04	0.6127	0.9412	1.633
**totresdev**	45.56	5899	10.42	15.15	27.06	44.02

or: fixed points   orz: semi-fixed points

e.gender

	mean	sd	MC_error	val2.5pc	median	val97.5pc
**B**	3.133	103.7	3.042	-155.8	40.01	116.1
**MD[1,2]**	-3.975	103.7	3.042	-117	-40.89	154.9
**MD[1,3]**	-3.169	103.7	3.042	-116.2	-40.15	155.8
**MD[1,4]**	-4.68	103.7	3.042	-117.8	-41.71	154.3
**MD[1,5]**	-4.912	103.7	3.042	-118	-41.83	154
**MD[2,3]**	0.8066	0.6342	0.001117	-0.4649	0.8106	2.059

**MD[2,4]**	-0.7052	0.7948	0.001426	-2.293	-0.7031	0.8734
**MD[2,5]**	-0.9365	0.4086	6.55E-04	-1.751	-0.9365	-0.1248
**MD[3,4]**	-1.512	0.795	0.001084	-3.091	-1.513	0.07633
**MD[3,5]**	-1.743	0.7392	0.001394	-3.199	-1.749	-0.262
**MD[4,5]**	-0.2313	0.8877	0.001684	-1.995	-0.2328	1.546
**MDz[1,1,2]**	-0.8427	0.4645	0.001383	-1.764	-0.8443	0.08812
**MDz[1,1,3]**	-0.03602	0.7226	0.001892	-1.48	-0.03268	1.396
**MDz[1,1,4]**	-1.548	0.8951	0.002237	-3.337	-1.545	0.2364
**MDz[1,1,5]**	-1.779	0.529	0.001442	-2.83	-1.78	-0.7242
**MDz[1,2,3]**	0.8066	0.6342	0.001117	-0.4649	0.8106	2.059
**MDz[1,2,4]**	-0.7052	0.7948	0.001426	-2.293	-0.7031	0.8734
**MDz[1,2,5]**	-0.9365	0.4086	6.55E-04	-1.751	-0.9365	-0.1248
**MDz[1,3,4]**	-1.512	0.795	0.001084	-3.091	-1.513	0.07633
**MDz[1,3,5]**	-1.743	0.7392	0.001394	-3.199	-1.749	-0.262
**MDz[1,4,5]**	-0.2313	0.8877	0.001684	-1.995	-0.2328	1.546
**SUCRA[1]**	0.3333	0.4714	0.01383	0	0	1
**SUCRA[2]**	0.4396	0.1744	0.003484	0	0.5	0.75
**SUCRA[3]**	0.2007	0.1565	0.003454	0	0.25	0.5
**SUCRA[4]**	0.713	0.2259	0.003448	0.25	0.75	1
**SUCRA[5]**	0.8133	0.1797	0.003482	0.5	0.75	1
**SUCRAz[1]**	0.1395	0.1589	4.06E-04	0	0	0.5
**SUCRAz[2]**	0.514	0.138	2.29E-04	0.25	0.5	0.75
**SUCRAz[3]**	0.164	0.1823	3.78E-04	0	0.25	0.5
**SUCRAz[4]**	0.7863	0.2166	4.00E-04	0.25	0.75	1
**SUCRAz[5]**	0.8961	0.1365	2.47E-04	0.75	1	1
**sd**	0.9868	0.2642	7.20E-04	0.6137	0.9409	1.629
**totresdev**	27.71	7.4	0.01479	15.19	27.05	44.04

Gender ratio=(n[female] x 2+ n[male] x1)/n[total]

or: Gender ratio≤1.5 orz: Gender ratio>1.5

5 GRADE for the primary outcomes

5.1 Overall response rate

5.1.1 Contribution of low or moderate RoB comparisons to each network estimate

Based on the assessment of RoB for each comparison and the contribution matrix detailing contribution of each direct comparison to all network estimates, the following bar graphs show the percentage of low or moderate RoB contributions for each network estimate.

Each bar corresponds to a NMA relative treatment effect and shows how much

information comes from comparisons at low risk of bias [green] or moderate risk of bias [yellow].

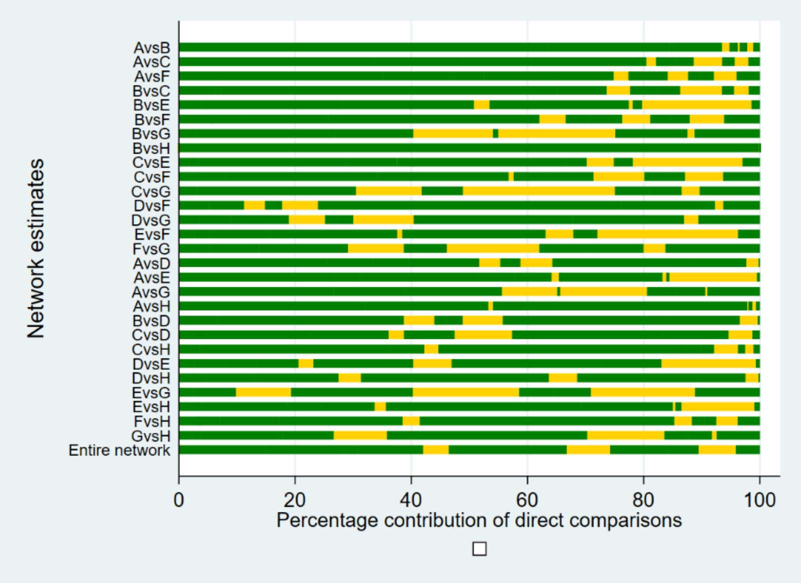

6	P1	P2	P3	P4	P5	P6	P7	P8	P9	P10	P11	P12	P13	P14	P15
AvsB	56.2	7.3	12.1	8.9	0.7	8.4	1.5	0.0	0.4	0.8	0.3	0.6	0.6	1.0	1.2
AvsC	33.8	6.4	8.3	30.9	0.9	0.2	1.8	0.0	3.2	3.1	4.9	1.1	1.1	2.3	2.0
AvsF	30.6	4.6	11.9	5.5	1.2	21.1	2.7	0.0	2.7	3.9	3.5	2.2	2.2	3.9	4.0
BvsC	3.6	2.7	0.9	56.0	2.0	8.5	4.2	0.0	4.5	4.0	7.2	1.1	1.1	2.5	1.9
BvsE	3.0	0.9	2.1	22.3	8.6	13.9	2.9	0.0	23.2	0.6	0.7	0.8	0.8	18.9	1.4
BvsF	5.7	0.0	5.7	14.3	2.1	34.3	4.7	0.0	3.8	5.7	4.8	3.4	3.4	5.9	6.1
BvsG	2.8	0.8	2.1	20.1	1.2	13.4	13.9	0.0	0.0	0.7	20.1	6.2	6.2	1.2	11.2
BvsH	0.0	0.0	0.0	0.0	0.0	0.0	0.0	100.0	0.0	0.0	0.0	0.0	0.0	0.0	0.0
CvsE	0.6	1.1	1.7	17.5	8.0	8.9	0.0	0.0	28.8	3.7	4.6	1.6	1.7	18.9	3.0
CvsF	2.3	1.8	4.0	26.0	0.4	22.3	1.0	0.0	6.2	7.3	8.8	3.5	3.5	6.6	6.3
CvsG	0.4	1.2	1.5	19.5	0.2	7.8	11.5	0.0	3.3	3.6	26.2	5.7	5.7	3.1	10.4
DvsF	0.8	0.3	1.0	3.1	0.2	5.9	3.8	0.0	1.2	1.6	6.1	52.2	16.2	1.4	6.3
DvsG	1.3	0.5	1.7	5.2	0.3	10.0	6.4	0.0	2.1	2.6	10.4	27.3	19.2	2.4	10.6
EvsF	1.8	0.8	2.6	10.6	7.0	14.8	1.1	0.0	20.3	4.2	4.8	2.1	2.1	24.2	3.7
FvsG	1.9	0.7	2.7	7.9	0.5	15.3	9.8	0.0	3.2	4.0	15.9	9.0	9.0	3.7	16.2
AvsD	22.1	3.4	7.9	5.4	0.7	12.1	3.8	0.0	1.3	2.0	5.5	23.9	9.5	2.1	0.2
AvsE	26.7	4.4	7.9	12.6	6.3	6.4	1.4	0.0	17.6	0.0	0.7	0.3	0.3	15.0	0.5
AvsG	26.6	4.3	7.7	10.6	0.6	5.8	9.7	0.0	0.2	0.1	15.0	5.0	5.0	0.4	9.0
AvsH	32.0	4.2	6.9	5.0	0.4	4.8	0.8	43.1	0.2	0.5	0.2	0.4	0.4	0.6	0.7
BvsD	3.3	0.2	3.2	11.4	1.3	19.4	5.4	0.0	1.8	2.9	6.9	28.9	11.9	3.1	0.4
CvsD	1.3	1.2	2.4	17.7	0.2	13.4	2.8	0.0	3.9	4.6	9.9	25.8	11.4	4.1	1.3
CvsH	2.1	1.5	0.5	32.1	1.1	4.9	2.4	42.6	2.6	2.3	4.1	0.6	0.6	1.4	1.1
DvsE	0.8	0.4	1.3	5.8	5.0	7.2	2.7	0.0	14.9	2.1	6.6	26.1	10.0	16.2	0.7
DvsH	2.4	0.1	2.2	8.1	0.9	13.8	3.8	29.0	1.3	2.0	4.9	20.5	8.5	2.2	0.3
EvsG	0.2	0.1	0.1	2.2	6.6	0.6	9.7	0.0	20.7	0.1	18.3	6.2	6.2	17.9	11.1

EvsH	2.0	0.6	1.4	14.8	5.7	9.2	1.9	33.6	15.4	0.4	0.4	0.5	0.5	12.5	0.9
FvsH	3.5	0.0	3.5	8.9	1.3	21.3	2.9	37.9	2.4	3.5	3.0	2.1	2.1	3.7	3.8
GvsH	1.9	0.5	1.4	13.3	0.8	8.9	9.2	34.0	0.0	0.5	13.3	4.1	4.1	0.8	7.4

Compaison	AvsB	AvsC	AvsF	BvsC	BvsE	BvsF	BvsG	BvsH	CvsE
Moderate percent	2.8%	9%	10.1%	13.9%	22.5%	15.4%	35.2%	0%	23.5%
CvsF	CvsG	DvsF	DvsG	EvsF	FvsG	AvsD	AvsE	AvsG	AvsH
16.4%	40.8%	11.3%	19.2%	30.1%	29.4%	11.4%	17.1%	25.1%	1.6%
BvsD	CvsD	CvsH	DvsE	DvsH	EvsG	EvsH	FvsH	GvsH	Entire network
15.4%	16.8%	7.9%	25.5%	10.9%	45.9%	14.8%	9.6%	23.3%	18.2

5.1.2 Table of reasons for downgrading

Based on all the above information, we GRADEd each network estimate according to the following criteria.

(1) Study limitations: We downgraded by one level when the contributions from low RoB comparisons were less than 30% and contributions from moderate RoB comparisons were 70% or greater.

(2) Imprecision: We considered a clinically meaningful threshold for OR to be 0.80 or 1.25 and downgraded the estimate if the OR point estimate is 1 or more and the lower limit of its CrI is below 0.80; or if the OR point estimate is less than 1 and the upper limit of its CrI is above 1.25.

(3) Inconsistency: We rated two concepts, heterogeneity and incoherence (inconsistency), in this domain.

For heterogeneity, we looked at the common tau and found that it is low compared to the expected value as reported in the literature (Turner RM et al (2012) Int J Epidemiol, 41, 818-827) , so we did not downgrade any network estimate for heterogeneity. For inconsistency, we looked at the results of side splitting and we downgraded the comparisons with important inconsistency ($p<0.05$), where we have not downgraded for imprecision (we did not downgrade the same network estimate for both imprecision and inconsistency).

(4) Indirectness: We have assured transitivity in our network by limiting the included studies to chronic fatigue syndrome with the diagnostic criteria of "1994 CDC" .We further ran various subgroup analysis, and assured that they did not violate transitivity of the network.

(5) Publication bias: The comparison-adjusted funnel plot did not suggest obvious publication bias. However, we cannot completely rule out the possibility that some studies are still missing. Considering that the field of CFS trials in the past has been prone to publication bias, the review team decided by default to downgrade all the included studies for potential publication bias by one level.

	Study limitations	Imprecision	Inconsistency	Indirectness	Publication bias	GRADE
AvsB	No	No	No	No	Downgrade	MODERATE

	downgrade	downgrade	downgrade	downgrade		
AvsC	No downgrade	Downgrade Because point estimate >1.0 but lower limit<0.80	No downgrade	No downgrade	Downgrade	LOW
AvsF	No downgrade	No downgrade	No downgrade	No downgrade	Downgrade	MODERATE
BvsC	No downgrade	No downgrade	No downgrade	No downgrade	Downgrade	MODERATE
BvsE	No downgrade	No downgrade	No downgrade	No downgrade	Downgrade	MODERATE
BvsF	No downgrade	No downgrade	No downgrade	No downgrade	Downgrade	MODERATE
BvsG	No downgrade	No downgrade	No downgrade	No downgrade	Downgrade	MODERATE
BvsH	No downgrade	No downgrade	No downgrade	No downgrade	Downgrade	MODERATE
CvsE	No downgrade	Downgrade Because point estimate >1.0 but lower limit<0.80	Downgrade because sidesplitting p=0.014	No downgrade	Downgrade	VERY LOW
CvsF	No downgrade	No downgrade	No downgrade	No downgrade	Downgrade	MODERATE
CvsG	No downgrade	No downgrade	No downgrade	No downgrade	Downgrade	MODERATE
DvsF	No downgrade	No downgrade	No downgrade	No downgrade	Downgrade	MODERATE
DvsG	No downgrade	No downgrade	No downgrade	No downgrade	Downgrade	MODERATE
EvsF	No downgrade	No downgrade	No downgrade	No downgrade	Downgrade	MODERATE
FvsG	No downgrade	No downgrade	No downgrade	No downgrade	Downgrade	MODERATE

A， acupuncture with moxibustion； B， acupuncture； C， moxibutsion； D， acupuncture with THM； E， moxibustion with THM； F， traditional chinses herbal medicine(THM)；G，western medicine；H，no control group.

5.2 FS-14 total score of CFS patients

5.2.1 Contribution of low or moderate RoB comparisons to each network estimate

Based on the assessment of RoB for each comparison and the contribution matrix detailing contribution of each direct comparison to all network estimates, the following bar graphs show the percentage of low or moderate RoB contributions for each network estimate.

Each bar corresponds to a NMA relative treatment effect and shows how much information comes from comparisons at low risk of bias [green] or moderate risk of bias [yellow].

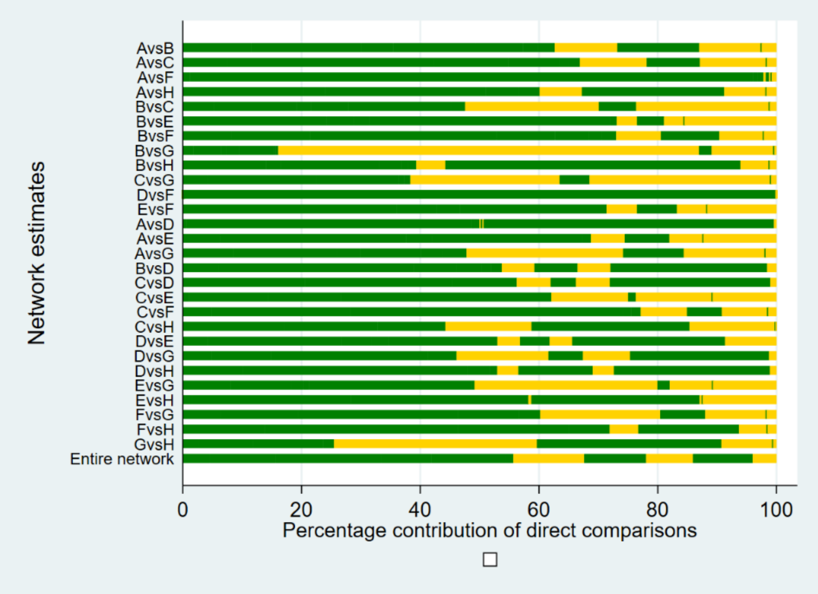

6	P1	P2	P3	P4	P5	P6	P7	P8	P9	P10	P11	P12
AvsB	11.5	18.6	5.4	13.8	8.1	2.5	2.9	10.5	13.8	10.5	0..0	2.5
AvsC	7.5	34.9	3.5	9.0	8.6	1.6	1.9	11.3	9.0	11.3	0.0	1.6
AvsF	0.5	0.7	94.0	0.6	0.3	0.8	0.9	0.4	0.6	0.4	0.0	0.8
AvsH	7.8	12.6	3.6	27.0	5.5	1.7	1.9	7.1	24.0	7.1	0.0	1.7.
BvsC	5.2	13.9	2.4	6.3	17.3	1.1	1.3	22.5	6.3	22.5	0.0	1.1
BvsE	3.8	6.1	14.4	4.5	2.6	40.5	1.1	3.5	4.5	3.4	0.0	15.5
BvsF	8.2	13.3	31.3	9.9	5.8	2.1	2.4	7.5	9.9	7.5	0.0	2.1
BvsG	1.8	4.7	0.8	2.1	5.8	0.4	0.4	70.9	2.1	10.6	0.0	0.4
BvsH	5.4	8.7	2.5	16.5	3.8	1.2	1.3	4.9	49.7	4.9	0.0	1.2
CvsG	4.2	11.2	2.0	5.1	13.9	0.9	1.0	25.1	5.1	30.6	0.0	0.9
DvsF	0.0	0.0	0.0	0.0	0.0	0.0	0.0	0.0	0.0	0.0	99.9	0.0
EvsF	5.6	9.0	21.4	6.7	3.9	23.0	1.7	5.1	6.7	5.1	0.0	11.7
AvsD	0.2	0.4	48.0	0.3	0.2	0.4	0.5	0.2	0.3	0.2	48.9	0.4
AvsE	6.3	10.1	13.7	7.5	4.4	25.3	1.4	5.7	7.5	5.7	0.0	12.3
AvsG	8.5	17.5	4.0	10.2	3.7	1.8	2.1	26.4	10.2	13.8	0.0	1.8
BvsD	6.2	9.8	23.1	7.3	4.2	1.6	1.8	5.5	7.3	5.5	26.4	1.6
CvsD	3.6	17.0	24.8	4.3	4.4	1.0	1.2	5.7	4.3	5.7	27.1	1.2
CvsE	1.1	13.3	10.9	1.3	9.9	25.4	0.2	12.9	1.3	12.9	0.0	10.8

CvsF	4.9	23.3	34.1	5.9	6.0	1.4	1.6	7.8	5.9	7.8	0.0	1.4
CvsH	0.7	16.4	0.3	15.4	11.1	0.1	0.2	14.5	26.6	14.5	0.0	0.1
DvsE	4.2	6.7	15.9	5.0	2.9	17.1	1.2	3.8	5.0	3.8	25.8	8.7
DvsG	4.9	10.0	20.7	5.8	2.1	1.3	1.5	15.5	5.8	7.9	23.4	1.3
DvsH	3.9	6.3	24.0	13.8	2.7	1.1	1.3	3.6	12.5	3.6	26.3	1.1
EvsG	1.7	6.3	10.2	2.1	0.9	27.3	0.6	30.8	2.1	7.3	0.0	10.7
EvsH	0.6	0.6	12.4	14.0	0.4	29.8	0.0	0.5	28.3	0.5	0.0	12.4
FvsG	6.3	13.0	27.0	7.6	2.7	1.7	1.9	20.2	7.6	10.3	0.0	1.7
FvsH	5.3	8.5	32.5	18.7	3.7	1.5	1.7	4.8	17.0	4.8	0.0	1.5
GvsH	2.5	8.1	1.2	11.8	0.6	0.6	0.6	34.2	31.1	8.7	0.0	0.6

Compaison	AvsB	AvsC	AvsF	AvsH	BvsC	BvsE	BvsF	BvsG	BvsH
Moderate percent	23.5 %	24.2%	1.6%	15.9%	46.1%	22.4%	17.1%	81.9%	11%
CvsG	DvsF	EvsF	AvsD	AvsE	AvsG	BvsD	CvsD	CvsE	CvsF
56.6%	0.0%	21.9%	0.8%	23.7%	42%	12.6%	12.6%	36.6%	17%
CvsH	DvsE	DvsG	DvsH	EvsG	EvsH	FvsG	FvsH	GvsH	Entire network
29.1%	16.3%	24.7%	8.3%	48.8%	13.4%	32.2%	11.1%	43.5%	23.8%

5.2.2 Table of reasons for downgrading

Based on all the above information, we GRADEd each network estimate according to the following criteria.

(1) Study limitations: We downgraded by one level when the contributions from low RoB comparisons were less than 30% and contributions from moderate RoB comparisons were 70% or greater.

(2) Imprecision: We considered a clinically meaningful threshold for OR to be 0.80 or 1.25 and downgraded the estimate if the OR point estimate is 1 or more and the lower limit of its CrI is below 0.80; or if the OR point estimate is less than 1 and the upper limit of its CrI is above 1.25.

(3) Inconsistency: We rated two concepts, heterogeneity and incoherence (inconsistency), in this domain.

For heterogeneity, we looked at the common tau and found that it is low compared to the expected value as reported in the literature (Turner RM et al (2012) Int J Epidemiol, 41, 818-827) , so we did not downgrade any network estimate for heterogeneity. For inconsistency, we looked at the results of side splitting and we downgraded the comparisons with important inconsistency ($p<0.05$), where we have not downgraded for imprecision (we did not downgrade the same network estimate for both imprecision and inconsistency).

(4) Indirectness: We have assured transitivity in our network by limiting the included studies to chronic fatigue syndrome with the diagnostic criteria of "1994 CDC" .We further ran various subgroup analysis, and assured that they did not violate transitivity of the network.

(5) Publication bias: The comparison-adjusted funnel plot did not suggest obvious

publication bias. However, we cannot completely rule out the possibility that some studies are still missing. Considering that the field of CFS trials in the past has been prone to publication bias, the review team decided by default to downgrade all the included studies for potential publication bias by one level.

	Study limitations	Imprecision	Inconsistency	Indirectness	Publication bias	GRADE
AvsB	No downgrade	No downgrade	No downgrade	No downgrade	Downgrade	MODERATE
AvsC	No downgrade	No downgrade	No downgrade	No downgrade	Downgrade	MODERATE
AvsF	No downgrade	No downgrade	No downgrade	No downgrade	Downgrade	MODERATE
AvsH	No downgrade	No downgrade	No downgrade	No downgrade	Downgrade	MODERATE
BvsC	No downgrade	Downgrade Because point estimate >1.0 but lower limit<0.80	No downgrade	No downgrade	Downgrade	LOW
BvsE	No downgrade	Downgrade Because point estimate >1.0 but lower limit<0.80	No downgrade	No downgrade	Downgrade	LOW
BvsF	No downgrade	No downgrade	No downgrade	No downgrade	Downgrade	MODERATE
BvsG	Downgrade because >70% contribution from moderate RoB comparisons	No downgrade	No downgrade	No downgrade	Downgrade	LOW
BvsH	No downgrade	No downgrade	No downgrade	No downgrade	Downgrade	MODERATE
CvsG	No downgrade	No downgrade	No downgrade	No downgrade	Downgrade	MODERATE
DvsF	No downgrade	No downgrade	No downgrade	No downgrade	Downgrade	MODERATE
EvsF	No downgrade	No downgrade	No downgrade	No downgrade	Downgrade	MODERATE

A， acupuncture with moxibustion； B， acupuncture； C， moxibutsion； D，

acupuncture with THM； E， moxibustion with THM； F， traditional chinses herbal medicine(THM)；G，western medicine；H，no control group.

5.3 FS-14 physical score of CFS patients

5.3.1 Contribution of low or moderate RoB comparisons to each network estimate

Based on the assessment of RoB for each comparison and the contribution matrix detailing contribution of each direct comparison to all network estimates, the following bar graphs show the percentage of low or moderate RoB contributions for each network estimate.

Each bar corresponds to a NMA relative treatment effect and shows how much information comes from comparisons at low risk of bias [green] or moderate risk of bias [yellow].

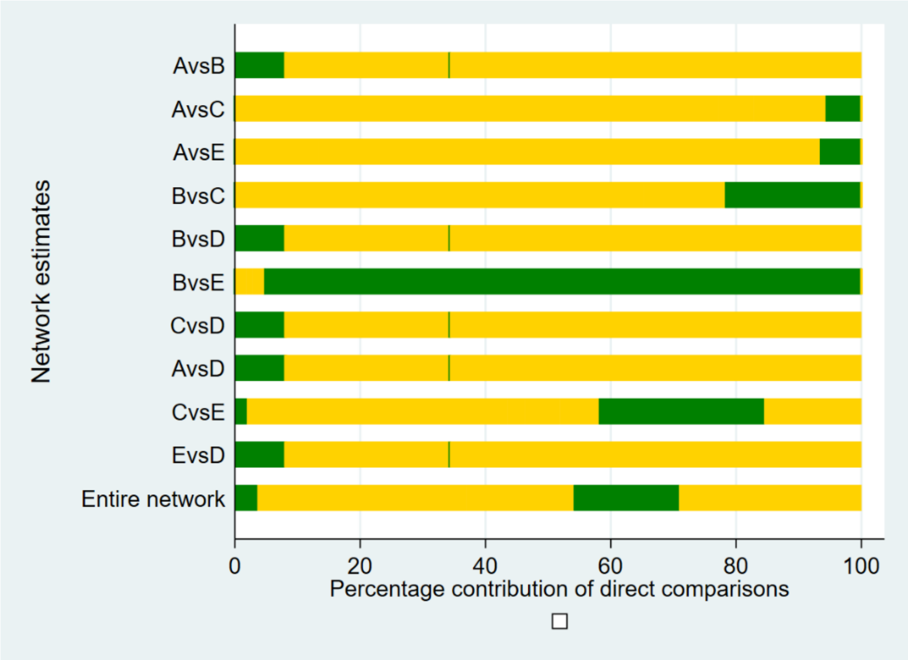

7	P1	P2	P3	P4	P5	P6	P7
AvsB	8.1	0.0	0.0	0.0	26.2	0.0	65.7
AvsC	0.0	77.1	5.7	11.4	0.0	5.7	0.0
AvsE	0.0	13.3	66.9	13.3	0.0	6.6	0.0
BvsC	0.0	43.5	21.8	12.9	0.0	21.8	0.0
BvsD	8.1	0.0	0.0	0.0	26.2	0.0	65.7
BvsE	0.0	1.9	0.9	1.9	0.0	95.3	0.0
CvsD	8.1	0.0	0.0	0.0	26.2	0.0	65.7
AvsD	8.1	0.0	0.0	0.0	26.2	0.0	65.7
CvsE	1.9	41.6	2.8	5.6	6.2	26.4	15.5
EvsD	8.1	0.0	0.0	0.0	26.2	0.0	65.7

Compaison	AvsB	AvsC	AvsE	BvsC	BvsD	BvsE	CvsD	AvsD	CvsE
Moderate	91.9%	94.3%	93.4%	78.2%	91.9%	4.7%	91.9%	91.9%	71.7%

percent									
EvsD	Entire network								
91.9%	79.6%								

5.3.2 Table of reasons for downgrading

Based on all the above information, we GRADEd each network estimate according to the following criteria.

(1) Study limitations: We downgraded by one level when the contributions from low RoB comparisons were less than 30% and contributions from moderate RoB comparisons were 70% or greater.

(2) Imprecision: We considered a clinically meaningful threshold for OR to be 0.80 or 1.25 and downgraded the estimate if the OR point estimate is 1 or more and the lower limit of its CrI is below 0.80; or if the OR point estimate is less than 1 and the upper limit of its CrI is above 1.25.

(3) Inconsistency: We rated two concepts, heterogeneity and incoherence (inconsistency), in this domain.

For heterogeneity, we looked at the common tau and found that it is low compared to the expected value as reported in the literature (Turner RM et al (2012) Int J Epidemiol, 41, 818-827) , so we did not downgrade any network estimate for heterogeneity. For inconsistency, we looked at the results of side splitting and we downgraded the comparisons with important inconsistency ($p<0.05$), where we have not downgraded for imprecision (we did not downgrade the same network estimate for both imprecision and inconsistency).

(4) Indirectness: We have assured transitivity in our network by limiting the included studies to chronic fatigue syndrome with the diagnostic criteria of "1994 CDC" .We further ran various subgroup analysis, and assured that they did not violate transitivity of the network.

(5) Publication bias: The comparison-adjusted funnel plot did not suggest obvious publication bias. However, we cannot completely rule out the possibility that some studies are still missing. Considering that the field of CFS trials in the past has been prone to publication bias, the review team decided by default to downgrade all the included studies for potential publication bias by one level.

	Study limitations	Imprecision	Inconsistency	Indirectness	Publication bias	GRADE
AvsB	Downgrade because >70% contribution from moderate RoB comparisons	No downgrade	No downgrade	No downgrade	Downgrade	LOW
AvsC	Downgrade because >70%	No downgrade	No downgrade	No downgrade	Downgrade	LOW

	contribution from moderate RoB comparisons					
AvsE	Downgrade because >70% contribution from moderate RoB comparisons	No downgrade	Downgrade because sidesplitting p=0.004	No downgrade	Downgrade	VERY LOW
BvsC	Downgrade because >70% contribution from moderate RoB comparisons	Downgrade Because point estimate >1.0 but lower limit<0.80	No downgrade	No downgrade	Downgrade	VERY LOW
BvsD	Downgrade because >70% contribution from moderate RoB comparisons	No downgrade	No downgrade	No downgrade	Downgrade	LOW
BvsE	No downgrade	No downgrade	Downgrade because sidesplitting p=0.001	No downgrade	Downgrade	LOW
CvsD	Downgrade because >70% contribution from moderate RoB comparisons	No downgrade	No downgrade	No downgrade	Downgrade	LOW

A，acupuncture with moxibustion；B，acupuncture；C，moxibutsion； D，western medicine；E，no control group.

5.4 FS-14 mental score of CFS patients

5.4.1 Contribution of low or moderate RoB comparisons to each network estimate

Based on the assessment of RoB for each comparison and the contribution

matrix detailing contribution of each direct comparison to all network estimates, the following bar graphs show the percentage of low or moderate RoB contributions for each network estimate.

Each bar corresponds to a NMA relative treatment effect and shows how much information comes from comparisons at low risk of bias [green] or moderate risk of bias [yellow].

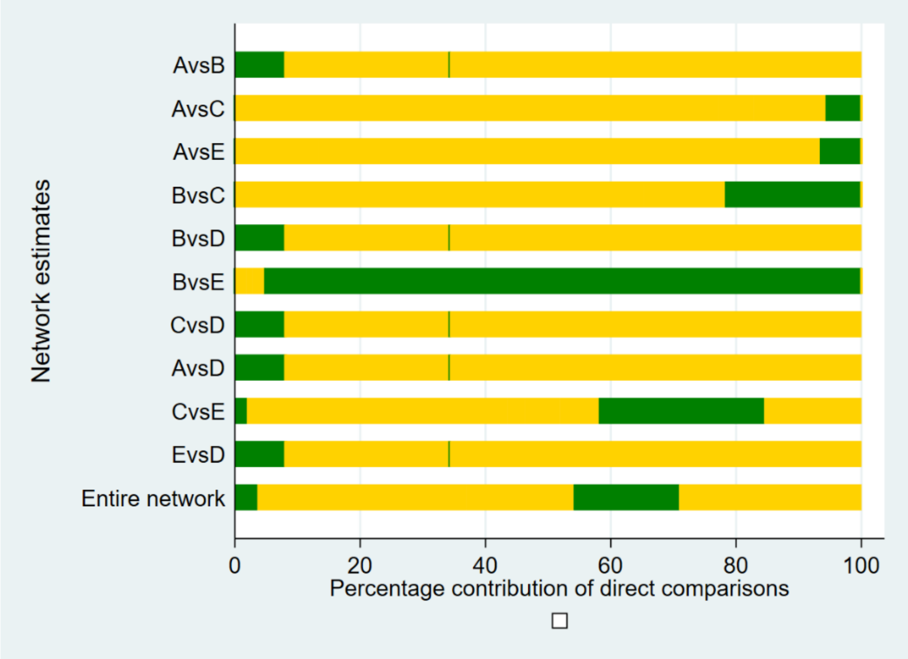

8	P1	P2	P3	P4	P5	P6	P7
AvsB	8.1	0.0	0.0	0.0	26.2	0.0	65.7
AvsC	0.0	77.1	5.7	11.4	0.0	5.7	0.0
AvsE	0.0	13.3	66.9	13.3	0.0	6.6	0.0
BvsC	0.0	43.5	21.8	12.9	0.0	21.8	0.0
BvsD	8.1	0.0	0.0	0.0	26.2	0.0	65.7
BvsE	0.0	1.9	0.9	1.9	0.0	95.3	0.0
CvsD	8.1	0.0	0.0	0.0	26.2	0.0	65.7
AvsD	8.1	0.0	0.0	0.0	26.2	0.0	65.7
CvsE	1.9	41.6	2.8	5.6	6.2	26.4	15.5
EvsD	8.1	0.0	0.0	0.0	26.2	0.0	65.7

Compaison	AvsB	AvsC	AvsE	BvsC	BvsD	BvsE	CvsD	AvsD	CvsE
Moderate percent	91.9%	94.3%	93.4%	78.2%	91.9%	4.7%	91.9%	91.9%	71.7%
EvsD	Entire network								
91.9%	79.6%								

5.4.2 Table of reasons for downgrading

Based on all the above information, we GRADEd each network estimate according to the

following criteria.

(1) Study limitations: We downgraded by one level when the contributions from low RoB comparisons were less than 30% and contributions from moderate RoB comparisons were 70% or greater.

(2) Imprecision: We considered a clinically meaningful threshold for OR to be 0.80 or 1.25 and downgraded the estimate if the OR point estimate is 1 or more and the lower limit of its CrI is below 0.80; or if the OR point estimate is less than 1 and the upper limit of its CrI is above 1.25.

(3) Inconsistency: We rated two concepts, heterogeneity and incoherence (inconsistency), in this domain.

For heterogeneity, we looked at the common tau and found that it is low compared to the expected value as reported in the literature (Turner RM et al (2012) Int J Epidemiol, 41, 818-827) , so we did not downgrade any network estimate for heterogeneity. For inconsistency, we looked at the results of side splitting and we downgraded the comparisons with important inconsistency ($p<0.05$), where we have not downgraded for imprecision (we did not downgrade the same network estimate for both imprecision and inconsistency).

(4) Indirectness: We have assured transitivity in our network by limiting the included studies to chronic fatigue syndrome with the diagnostic criteria of "1994 CDC" .We further ran various subgroup analysis, and assured that they did not violate transitivity of the network.

(5) Publication bias: The comparison-adjusted funnel plot did not suggest obvious publication bias. However, we cannot completely rule out the possibility that some studies are still missing. Considering that the field of CFS trials in the past has been prone to publication bias, the review team decided by default to downgrade all the included studies for potential publication bias by one level.

	Study limitations	Imprecision	Inconsistency	Indirectness	Publication bias	GRADE
AvsB	Downgrade because >70% contribution from moderate RoB comparisons	No downgrade	No downgrade	No downgrade	Downgrade	LOW
AvsC	Downgrade because >70% contribution from moderate RoB comparisons	Downgrade because point estimate <1.0 but upper limit >1.25	No downgrade	No downgrade	Downgrade	VERY LOW
AvsE	Downgrade because >70%	No downgrade	Downgrade because	No downgrade	Downgrade	VERY LOW

	contribution from moderate RoB comparisons		sidesplitting p=0.004			
BvsC	Downgrade because >70% contribution from moderate RoB comparisons	Downgrade Because point estimate >1.0 but lower limit<0.80	No downgrade	No downgrade	Downgrade	VERY LOW
BvsD	Downgrade because >70% contribution from moderate RoB comparisons	No downgrade	No downgrade	No downgrade	Downgrade	LOW
BvsE	No downgrade	No downgrade	Downgrade because sidesplitting p=0.004	No downgrade	Downgrade	LOW
CvsD	Downgrade because >70% contribution from moderate RoB comparisons	No downgrade	No downgrade	No downgrade	Downgrade	LOW

A，acupuncture with moxibustion；B，acupuncture；C，moxibutsion； D，western medicine；E，no control group.

Search strategy

Pubmed

History and Search Details

Download Delete

Search	Actions	Details	Query	Results	Time
#13	•••	>	Search: (((((((("Acupuncture"[Mesh]) OR "Acupuncture Points"[Mesh]) OR "Acupuncture Therapy"[Mesh]) OR "Acupuncture, Ear"[Mesh]) OR "Dry Needling"[Mesh]) OR "Moxibustion"[Mesh]) OR "Trigger Points"[Mesh]) OR "Meridians"[Mesh]) OR ((((((((((((((((((((((((acupoint injection[Title/Abstract]) OR (acupuncture[Title/Abstract])) OR (acupuncture[Title/Abstract] AND moxibustion[Title/Abstract])) OR (acupuncture points[Title/Abstract])) OR (acupuncture therapy[Title/Abstract])) OR (auricular acupuncture[Title/Abstract])) OR (auricular plaster[Title/Abstract])) OR (body acupuncture[Title/Abstract])) OR (coiling dragon needling[Title/Abstract])) OR (dermal needle[Title/Abstract])) OR (dry needling[Title/Abstract])) OR (ear acupuncture[Title/Abstract])) OR (ear seed pressure[Title/Abstract])) OR (Electro-acupuncture[Title/Abstract])) OR (Embedding[Title/Abstract])) OR (embedding therapy[Title/Abstract])) OR (fire needle[Title/Abstract])) OR (moxibustion[Title/Abstract])) OR (panlongci[Title/Abstract])) OR (percussopuncture[Title/Abstract])) OR (point injection[Title/Abstract])) OR (pricking blood[Title/Abstract])) OR (scalp acupuncture[Title/Abstract])) OR (trigger points[Title/Abstract])) OR (meridians[Title/Abstract]))) AND (("Fatigue Syndrome, Chronic"[Mesh]) OR ((((chronic fatigue syndrome[Title/Abstract]) OR (chronic fatigue[Title/Abstract])) OR (fatigue syndrome[Title/Abstract])) OR (Myalgic Encephalopathy[Title/Abstract])))	85	06:07:07
#12	•••	>	Search: ("Fatigue Syndrome, Chronic"[Mesh]) OR ((((chronic fatigue syndrome[Title/Abstract]) OR (chronic fatigue[Title/Abstract])) OR (fatigue syndrome[Title/Abstract])) OR (Myalgic Encephalopathy[Title/Abstract]))	8,371	06:06:57
#11	•••	>	Search: (((((((("Acupuncture"[Mesh]) OR "Acupuncture Points"[Mesh]) OR "Acupuncture Therapy"[Mesh]) OR "Acupuncture, Ear"[Mesh]) OR "Dry Needling"[Mesh]) OR "Moxibustion"[Mesh]) OR "Trigger Points"[Mesh]) OR "Meridians"[Mesh]) OR ((((((((((((((((((((((((acupoint injection[Title/Abstract]) OR (acupuncture[Title/Abstract])) OR (acupuncture[Title/Abstract] AND moxibustion[Title/Abstract])) OR (acupuncture points[Title/Abstract])) OR (acupuncture therapy[Title/Abstract])) OR (auricular acupuncture[Title/Abstract])) OR (auricular plaster[Title/Abstract])) OR (body acupuncture[Title/Abstract])) OR (coiling dragon needling[Title/Abstract])) OR (dermal needle[Title/Abstract])) OR (dry needling[Title/Abstract])) OR (ear acupuncture[Title/Abstract])) OR (ear seed pressure[Title/Abstract])) OR (Electro-acupuncture[Title/Abstract])) OR (Embedding[Title/Abstract])) OR (embedding therapy[Title/Abstract])) OR (fire needle[Title/Abstract])) OR (moxibustion[Title/Abstract])) OR (panlongci[Title/Abstract])) OR (percussopuncture[Title/Abstract])) OR (point injection[Title/Abstract])) OR (pricking blood[Title/Abstract])) OR (scalp acupuncture[Title/Abstract])) OR (trigger points[Title/Abstract])) OR (meridians[Title/Abstract]))	51,497	06:06:46
#7	•••	>	Search: (((chronic fatigue syndrome[Title/Abstract]) OR (chronic fatigue[Title/Abstract])) OR (fatigue syndrome[Title/Abstract])) OR (Myalgic Encephalopathy[Title/Abstract])	7,481	05:47:53

#6	•••	>	Search: **"Fatigue Syndrome, Chronic"[Mesh]** Sort by: **Most Recent**	5,504	05:47:08
#5	•••	>	Search: **((((((((((((((((((((((acupoint injection[Title/Abstract]) OR (acupuncture[Title/Abstract])) OR (acupuncture[Title/Abstract] AND moxibustion[Title/Abstract])) OR (acupuncture points[Title/Abstract])) OR (acupuncture therapy[Title/Abstract])) OR (auricular acupuncture[Title/Abstract])) OR (auricular plaster[Title/Abstract])) OR (body acupuncture[Title/Abstract])) OR (coiling dragon needling[Title/Abstract])) OR (dermal needle[Title/Abstract])) OR (dry needling[Title/Abstract])) OR (ear acupuncture[Title/Abstract])) OR (ear seed pressure[Title/Abstract])) OR (Electro-acupuncture[Title/Abstract])) OR (Embedding[Title/Abstract])) OR (embedding therapy[Title/Abstract])) OR (fire needle[Title/Abstract])) OR (moxibustion[Title/Abstract])) OR (panlongci[Title/Abstract])) OR (percussopuncture[Title/Abstract])) OR (point injection[Title/Abstract])) OR (pricking blood[Title/Abstract])) OR (scalp acupuncture[Title/Abstract])) OR (trigger points[Title/Abstract])) OR (meridians[Title/Abstract])**	44,442	05:45:36
#4	•••	>	Search: **((((((("Acupuncture"[Mesh]) OR "Acupuncture Points"[Mesh]) OR "Acupuncture Therapy"[Mesh]) OR "Acupuncture, Ear"[Mesh]) OR "Dry Needling"[Mesh]) OR "Moxibustion"[Mesh]) OR "Trigger Points"[Mesh]) OR "Meridians"[Mesh])** Sort by: **Most Recent**	25,866	05:41:33

Showing 1 to 7 of 7 entries

EMBASE

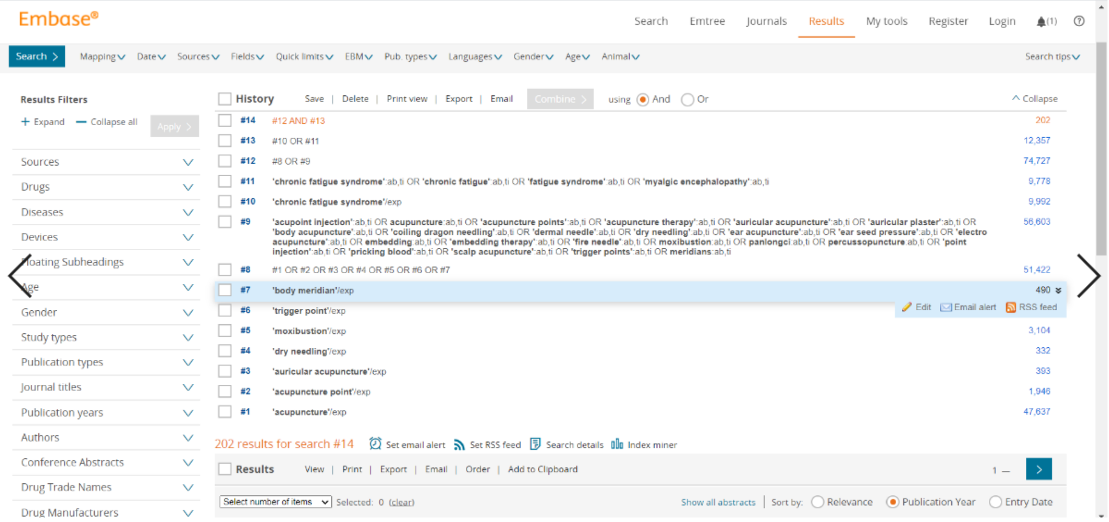

Cochrane Library

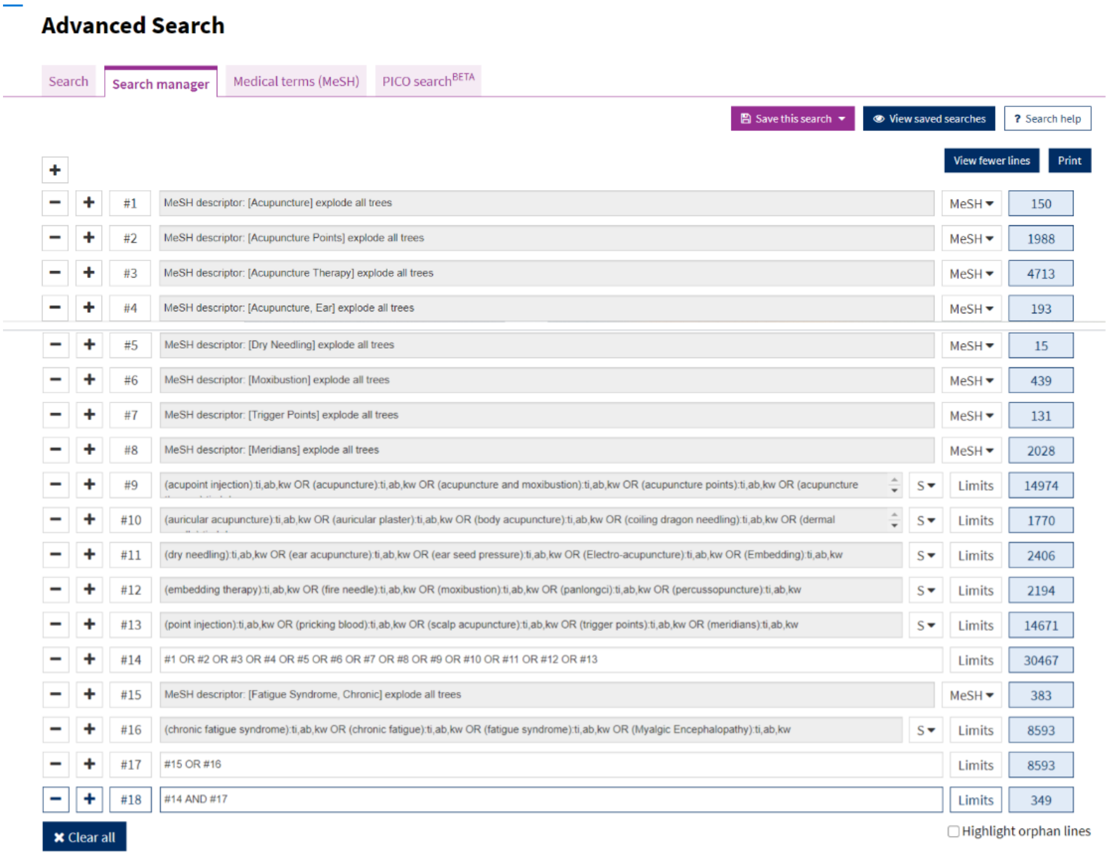

Web of Science

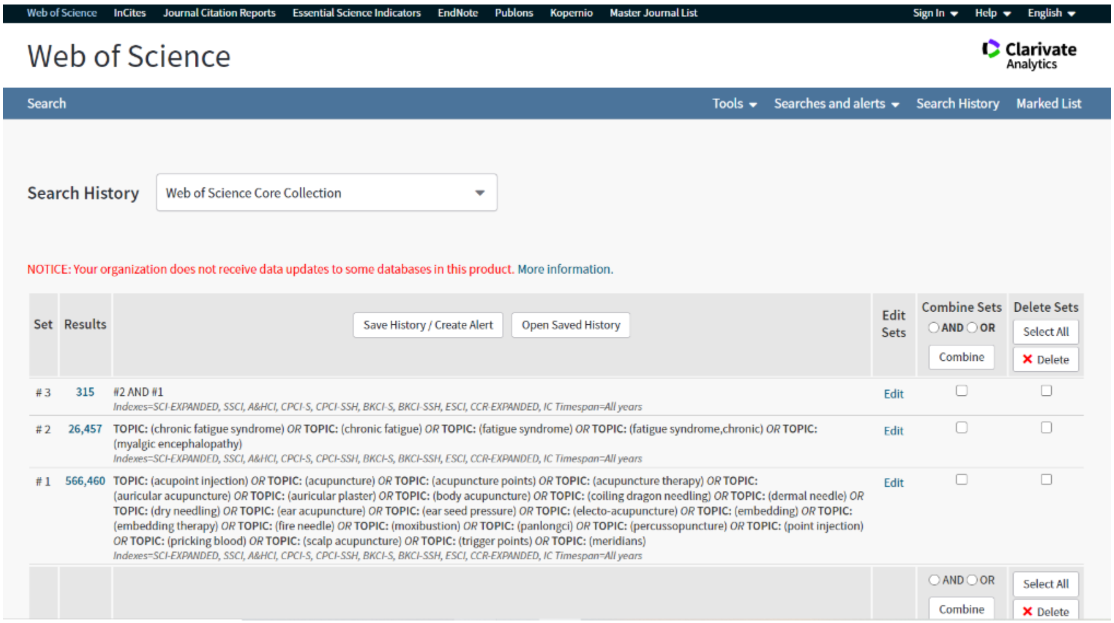

CNKI

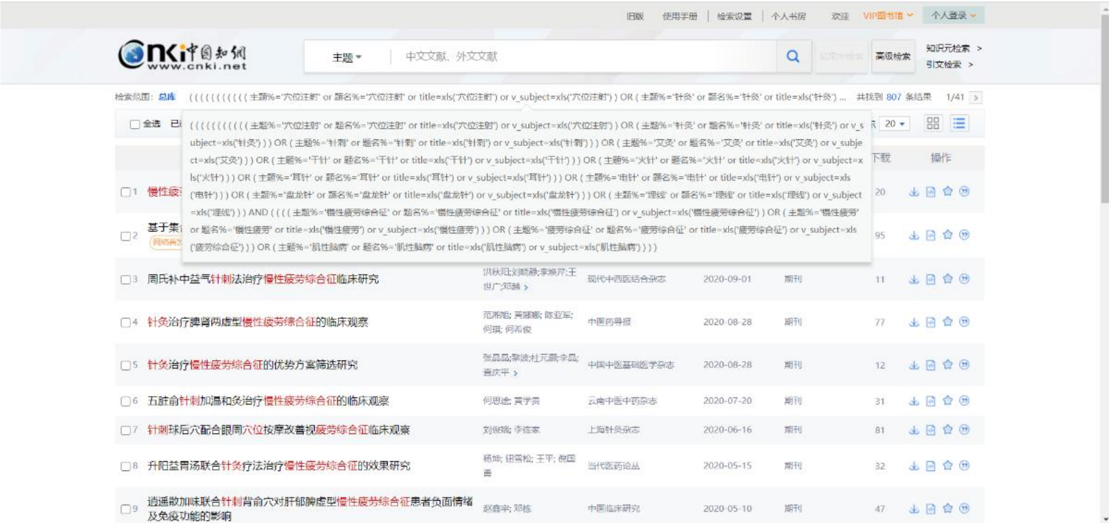

Wan Fang

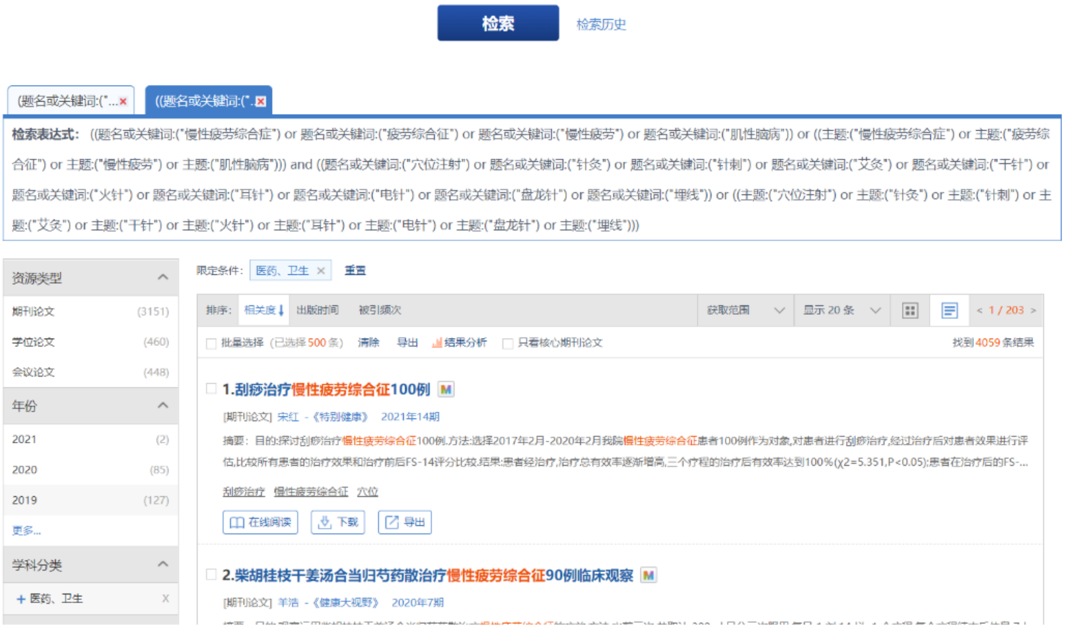

CQVIP

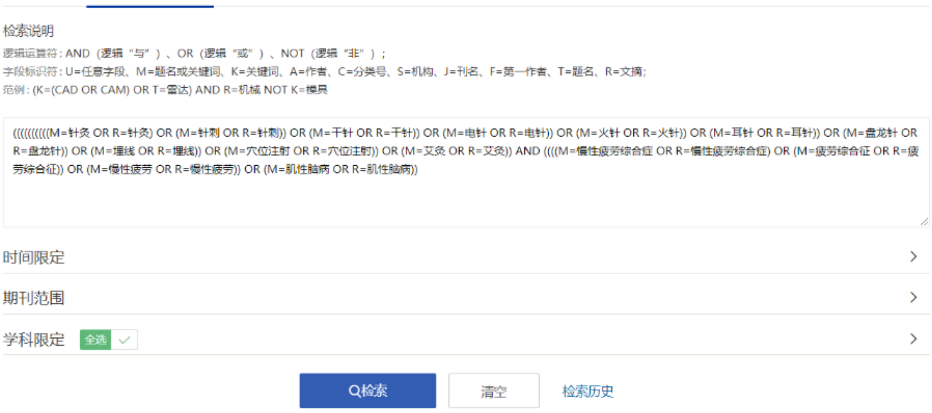
检索说明
逻辑运算符：AND（逻辑"与"）、OR（逻辑"或"）、NOT（逻辑"非"）；
字段标识符：U=任意字段、M=题名或关键词、K=关键词、A=作者、C=分类号、S=机构、J=刊名、F=第一作者、T=题名、R=文摘；
范例：(K=(CAD OR CAM) OR T=雷达) AND R=机械 NOT K=模具
(((((((((M=针灸 OR R=针灸) OR (M=针刺 OR R=针刺)) OR (M=干针 OR R=干针)) OR (M=电针 OR R=电针)) OR (M=火针 OR R=火针)) OR (M=耳针 OR R=耳针)) OR (M=盘龙针 OR R=盘龙针)) OR (M=埋线 OR R=埋线)) OR (M=穴位注射 OR R=穴位注射)) OR (M=艾灸 OR R=艾灸)) AND ((((M=慢性疲劳综合症 OR R=慢性疲劳综合症) OR (M=疲劳综合征 OR R=疲劳综合征)) OR (M=慢性疲劳 OR R=慢性疲劳)) OR (M=肌性脑病 OR R=肌性脑病))
时间限定
期刊范围
学科限定
全选
Q检索
清空
检索历史

SionMed

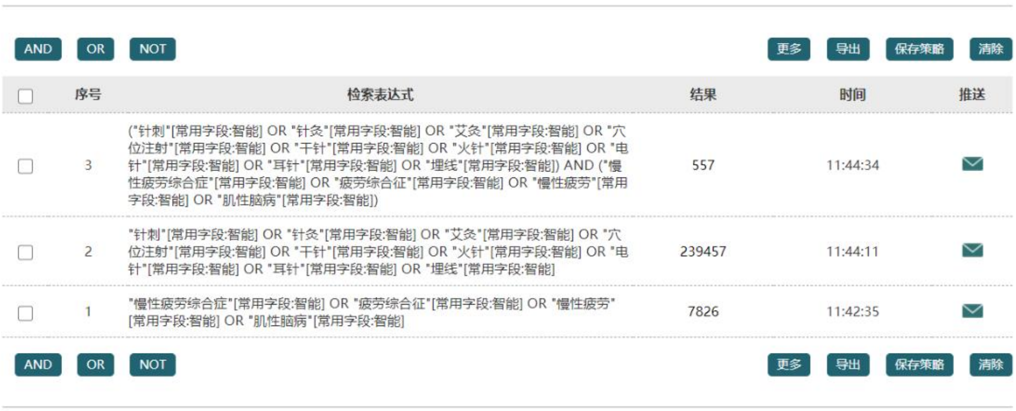
AND
OR
NOT
更多
导出
保存策略
清除
序号
检索表达式
结果
时间
推送
3
("针刺"[常用字段:智能] OR "针灸"[常用字段:智能] OR "艾灸"[常用字段:智能] OR "穴位注射"[常用字段:智能] OR "干针"[常用字段:智能] OR "火针"[常用字段:智能] OR "电针"[常用字段:智能] OR "耳针"[常用字段:智能] OR "埋线"[常用字段:智能]) AND ("慢性疲劳综合症"[常用字段:智能] OR "疲劳综合征"[常用字段:智能] OR "慢性疲劳"[常用字段:智能] OR "肌性脑病"[常用字段:智能])
557
11:44:34
2
"针刺"[常用字段:智能] OR "针灸"[常用字段:智能] OR "艾灸"[常用字段:智能] OR "穴位注射"[常用字段:智能] OR "干针"[常用字段:智能] OR "火针"[常用字段:智能] OR "电针"[常用字段:智能] OR "耳针"[常用字段:智能] OR "埋线"[常用字段:智能]
239457
11:44:11
1
"慢性疲劳综合症"[常用字段:智能] OR "疲劳综合征"[常用字段:智能] OR "慢性疲劳"[常用字段:智能] OR "肌性脑病"[常用字段:智能]
7826
11:42:35
AND
OR
NOT
更多
导出
保存策略
清除
